# Supplementary material for: Three distinct strategies lead to programmable aliphatic C−H oxidation in bicyclomycin biosynthesis
Source: Nat Commun. 2025 May 19;16:4651. doi: 10.1038/s41467-025-58997-8 (PMC12089406; doi:10.1038/s41467-025-58997-8)
Supplement: Supplementary file 1 — Supplementary Information [file 41467_2025_58997_MOESM1_ESM.pdf]

Supplementary Information

Three distinct strategies lead to programmable aliphatic C−H oxidation in bicyclomycin biosynthesis

Lian Wu[1,8], Jun-Bin He[2,8], Wanqing Wei[3,8], Hai-Xue Pan[2,4], Xin Wang[5], Sheng Yang[1]*, Yong Liang[5,6]*, Gong-Li Tang[2,4]*, Jiahai Zhou[7]*

[1]Key Laboratory of Synthetic Biology, CAS Center for Excellence in Molecular Plant Sciences, University of Chinese Academy of Sciences (CAS), CAS; Shanghai, 200032, China

[2]State Key Laboratory of Chemical Biology, Shanghai Institute of Organic Chemistry, CAS; Shanghai, 200032, China

[3]School of Biotechnology and Key Laboratory of Industrial Biotechnology of Ministry of Education, Jiangnan University; Wuxi, 214122, China

[4]School of Chemistry and Material Sciences, Hangzhou Institute for Advanced Study, University of CAS; Hangzhou, 310024, China

[5]Henan-Macquarie University Joint Centre for Biomedical Innovation, School of Life Sciences, Henan University; Kaifeng, 475004, China

[6]State Key Laboratory of Coordination Chemistry, Jiangsu Key Laboratory of Advanced Organic Materials, Chemistry and Biomedicine Innovation Center, School of Chemistry and Chemical Engineering, Nanjing University; Nanjing, 210023, China

[7]State Key Laboratory of Microbial Technology, Nanjing Normal University; Nanjing 210009, China

[8]These authors contributed equally: Lian Wu, Jun-Bin He, Wanqing Wei.

*Correspondence: Sheng Yang, Email: syang@sibs.ac.cn; Yong Liang, Email: yongliang@nju.edu.cn

Gong-Li Tang, Email: gltang@sioc.ac.cn; Jiahai Zhou, Email: jiahai@nnu.edu.cn

Table of contents

Supplementary Text.. 4-7
Supplementary Methods 7
Enzymatic syntheses 7
Quantum chemical model constructions..16
Molecular dynamics initial structural preparations...................................16
Supplementary Tables..18
Supplementary Table 1. Data collection and refinement statistics. 18
Supplementary Table 2. Primers used in the study of BcmE. 19
Supplementary Table 3. Primers used in the study of BcmC. 20
Supplementary Table 4. Primers used in the study of BcmG. 21
Supplementary Figures 22
Supplementary Fig. 1. The bond dissociation energy of different types of C-H. 22
Supplementary Fig. 2. The intrinsic site selectivity of **1** hydroxylation. 23
Supplementary Fig. 3. The intrinsic site selectivity of **2** hydroxylation. 24
Supplementary Fig. 4. The intrinsic site selectivity of **3** hydroxylation. 25
Supplementary Fig. 5. HPLC and LC-MS analyses of in vitro enzyme-catalyzed hydroxylation reactions...26
Supplementary Fig. 6. The structure of BcmE, BcmC and BcmG without substrates.27-28
Supplementary Fig. 7. The sequence alignment of SsBcmE, SoBcmC, SsBcmC and PaBcmG. 29
Supplementary Fig. 8. The omit map of substrate, αKG, Fe(II) and coordinating water in complex structure of SsBcmE^{T307A}, SoBcmC and PaBcmG. 30
Supplementary Fig. 9. HPLC analysis of in vitro reaction of SsBcmE and its mutants. 31
Supplementary Fig. 10. The stereo- and regioselectivity of C–H activation by SsBcmE, SoBcmC and PaBcmG. 32
Supplementary Fig. 11. The alignment of apo and complex structures of SsBcmE, SoBcmC and PaBcmG. 34

Supplementary Fig. 12. Origin of regio-selectivity in SsBcmE-catalysed hydroxylation reactions. 35
Supplementary Fig. 13. HPLC analysis of in vitro reaction of SoBcmC and its mutants. 37
Supplementary Fig. 14. HPLC analysis of in vitro reaction of PaBcmG and its mutants. 38-39
Supplementary Fig. 15. HPLC and LC-MS analyses of in vitro enzyme-catalyzed hydroxylation reactions..40
Supplementary Fig. 16. HPLC analysis of in vitro SsBcmE-catalyzed hydroxylation reactions using compounds **5**–**7** as substrates. 41
Supplementary Fig. 17. HPLC analysis of in vitro SoBcmC-catalyzed hydroxylation reactions using compounds **8**–**10** as substrates. 42
Supplementary Fig. 18. HPLC analysis of in vitro PaBcmG-catalyzed hydroxylation reactions using compounds **8a**–**10a** as substrates. 43
Supplementary Fig. 19. NMR spectrum. 44-172
References..173

Supplementary Text

Nucleotide and amino acid sequences

Codon-optimized nucleotide sequence of SsbcmE

ATGGCGTCACCCGATTCCGCCACCCTCCGGGAACCGGTCGTCCTGCCTCCCAT
GCCCGGTGAGCACGAGGCGCGGGCGGCGTATCCGCCGATCGGGCTGGAGCG
CTCCCGCGTCACCGGTGGCCGGCTCGTCTTCGACCGCGACGAGGGCTTCGAC
CGTGCCCTCGCGCAGGGGTTCTTCCTCGTACGGATCCCCGAGGGCACGGACC
CCGCCGCCGGCGACCGCTTCGCGGCCCACTTCCACGAGGAGCGGGCCGGCGG
GGACCCGCTGGACGCCTACCGCGGCTACCGCCACGTGCGCGTGCCCGGCGAC
TACCAGGGCTACTTCGACCGCGAGCACGACCAGTGGGAGAACTTCTACGTCG
AGAGGGACAACTGGGACGTGCTGCCATCCGAGGTCGCCCGGGTGGGCCGGG
GCATGGCCGGTCTCGGGGTCACGATCCTGCGCGGCGTCCTGGAGCACCTGCG
GCTGCCCCGGGAGCACTGGGCGCGCGTCACGGGCGGGCTCACCGAGGACCG
CGGCCACCAGATGCTCGCCTTCAACCACTTCCGGTCGCACAAGGGCGTGCGC
GGCTCGAAGTTCCACCGGGACTCCGGCTGGGTGACGGTCCTGCGGTCCGTGG
ACCCGGGTCTGCTCGCCCTCGTCGACGGGCGCCTGTGGGCCGTCGACCCGGA
GCCCGGCCACTTCATCGTCAACTTCGGCAGCTCCCTCGAAGTGCTGACCGAA
CGCCTCGACCGACCGGTGCGGGCCAATGTGCACGGCGTCGTCTCCACGGAAC
GGGCGCCGGGACAACCGGACCGGACCTCCTACGTCACCTTCCTCGACTCCGA
CCTCACCGGCACCGTCTACCGGTTCGAGAACGGCACGCCCCGGCCCCTCCAG
TCGGTGGCCGAGTTCGCCGGCCAGGAAGTCGGCCGGACCTACGACGACAGCG
GTGCGCTCTGA

Amino acid sequence of SsBcmE

MASPDSATLREPVVLPPMPGEHEARAAYPPIGLERSRVTGGRLVFDRDEGFDRAL
AQGFFLVRIPEGTDPAAGDRFAAHFHEERAGGDPLDAYRGYRHVRVPGDYQGY
FDREHDQWENFYVERDNWDVLPSEVARVGRGMAGLGVTILRGVLEHLRLPREH
WARVTGGLTEDRGHQMLAFNHFRSHKGVRGSKFHRDSGWVTVLRSVDPGLLA
LVDGRLWAVDPEPGHFIVNFGSSLEVLTERLDRPVRANVHGVVSTERAPGQPDR
TSYVTFLDSDLTGTVYRFENGTPRPLQSVAEFAGQEVGRTYDDSGAL

Codon-optimized Nucleotide sequence of SsbcmC

ATGACCACCAAAGAAATGACCCTGCAGCGCGCCCGTACCGCAAGCGGAGAAC
TGGTGTTTGAAACCGGTGGGGGTCTGAGCCAGGCCCTGCAGGACGGTTGTTT
CTACCTGGCCATTCCGGAAGACATCGATCTGGAACCGGGTAAACTGCTGTGCC
GTCAGTTTTATCGTCCGGCACATCCAGGTAGTCCGGAACTGCGTCCGTATCGTG
GTTTTCGTCGTAATGATGGTATTTATTTTGACCGTGAATACTATCAGACAGAACA
TATTCTGGCCGATGGCCCGGCACGTGAAAAATATCTGCCACCTGATGTTGTTGC
ACTGTGTGAACGTATGACCAGCCTGGCGCTGCTGGTTCTGACCAGTACCCTGA
CCGGACTGGGCATTGATGAAGCAGTTTGGGAAAAAGTTACAGGTGGTGCAGT
TGGAGGTGGTGGTACTCAGTGGTTTGCGGCAAGCCATTATCGTCCGGAGCGTC
ACCAGCTGGGTTGTGCACCTCATAAAGATACAGGATTTGTTACCGTTCTGTACA
TTGAACAAGATGGGCTGGAAAGTAGCGTTGGGGGGGAATGGATTCCGATTGC
ACCTCTGCCTGGCTATTTTCTGGTTAATTTCGGTGGTGCAACCGAACTGCTGAC
CGCACGTATGGGCCGTCCGGTTCAAGCAATTCTGCACCGTGTTCGTAGTTGTG
TTACTGAGCCGGCACGTGAGGATCGTTTTTCTTTTGCCGTTTTTGCGAATCCGC
CTGCCACCGGCGATCTGTACCAGATGTCAGAATCCGGAGAGCCGGTTGCTGTT
CGTGGGGTTGAGGAATTTCTGCGTGATTTTAATAATGAGACCTGGAGCGATCG
TCATACCGACTTTGGTATTACAACCACCGCCCCTGGTGAGGTTCATGAT

Amino acid sequence of SsBcmC

MTTKEMTLQRARTASGELVFETGGGLSQALQDGCFYLAIPEDIDLEPGKLLCRQF
YRPAHPGSPELRPYRGFRRNDGIYFDREYYQTEHILADGPAREKYLPPDVVALCE
RMTSLALLVLTSTLTGLGIDEAVWEKVTGGAVGGGGTQWFAASHYRPERHQLG
CAPHKDTGFVTVLYIEQDGLESSVGGEWIPIAPLPGYFLVNFGGATELLTARMGR
PVQAILHRVRSCVTEPAREDRFSFAVFANPPATGDLYQMSESGEPVAVRGVEEFL
RDFNNETWSDRHTDFGITTTAPGEVHD

Codon-optimized Nucleotide sequence of SobcmC

ATGACCACCAAAGAAATGACCCTGCAGCGCGCCCGTACCGCAAGCGGAGAAC
TGGTGTTTGAAACCGGTGGGGGTCTGAGCCAGGCCCTGCAGGACGGTTGTTT
CTACCTGGCCATTCCGGAAGACATCGATCTGGAACCGGGTAAACTGCTGTGCC
GTCAGTTTTATCGTCCGGCACATCCAGGTAGTCCGGAACTGCGTCCGTATCGTG
GTTTTCGTCGTAATGATGGTATTTATTTTGACCGTGAATACTATCAGACAGAACA
TATTCTGGCCGATGGCCCGGCACGTGAAAAATATCTGCCACCTGATGTTGTTGC

ACTGTGTGAACGTATGACCAGCCTGGCGCTGCTGGTTCTGACCAGTACCCTGA
CCGGACTGGGCATTGATGAAGCAGTTTGGGAAAAAGTTACAGGTGGTGCAGT
TGGAGGTGGTGGTACTCAGTGGTTTGCGGCAAGCCATTATCGTCCGGAGCGTC
ACCAGCTGGGTTGTGCACCTCATAAAGATACAGGATTTGTTACCGTTCTGTACA
TTGAACAAGATGGGCTGGAAAGTAGCGTTGGGGGGGAATGGATTCCGATTGC
ACCTCTGCCTGGCTATTTTCTGGTTAATTTCGGTGGTGCAACCGAACTGCTGAC
CGCACGTATGGGCCGTCCGGTTCAAGCAATTCTGCACCGTGTTCGTAGTTGTG
TTACTGAGCCGGCACGTGAGGATCGTTTTTCTTTTGCCGTTTTTGCGAATCCGC
CTGCCACCGGCGATCTGTACCAGATGTCAGAATCCGGAGAGCCGGTTGCTGTT
CGTGGGGTTGAGGAATTTCTGCGTGATTTTAATAATGAGACCTGGAGCGATCG
TCATACCGACTTTGGTATTACAACCACCGCCCCTGGTGAGGTTCATGAT

Amino acid sequence of SoBcmC

HMTTKEMTLQRARTASGELVFETGGGLSQALQDGCFYLAIPEDIDLEPGKLLCRQ
FYRPAHPGSPELRPYRGFRRNDGIYFDREYYQTEHILADGPAREKYLPPDVVALC
ERMTSLALLVLTSTLTGLGIDEAVWEKVTGGAVGGGGTQWFAASHYRPERHQL
GCAPHKDTGFVTVLYIEQDGLESSVGGEWIPIAPLPGYFLVNFGGATELLTARMG
RPVQAILHRVRSCVTEPAREDRFSFAVFANPPATGDLYQMSESGEPVAVRGVEEF
LRDFNNETWSDRHTDFGITTTAPGEVHDLE

Codon-optimized Nucleotide sequence of PabcmG

AACCCGAACGCAACCTATGCACTGGCAAGCGCAGAACTGATCGATGGCAAAC
TGCGCTTTGATAGCAGCGATGGCTTTGCACGCGCCATCGCAGATGGCTTTTTTT
TTGTGAAAAGCCCGAGCCTGGATCTGGCAGCAGGTGATACATTTGCACGTAAC
TTTTATCTGCCGCGTCAGGAAGGTCTGGGTGCTCCGTATCAGGGTTTTAGTCAG
TGGACCGAAGATCGTCTGGCACGTCGTGAAGGTTATTTTAGTCGTGATGTTGAT
CAGGTTGAACAGTTTTTTCTGGAAAGCCGTTTTTGGCAGACAGTTTTTCCGGG
TCCGCTGCTGCGTCAGGCAGAACGTATGCGTTCATTTAGTCTGGAAGTTCTGC
GTGCAGTTCTGGCTGAACTGGATCTGCCGGTTGAACTGTGGGATGAAGCAACC
GGTCGTTGTCTGAGCATGCAGGGTACATATCATCTGACCTTTAACCATTTTCGT
AGTCATGTTCGTGCACGTGGTCTGAATGTTCATAAAGATAGCGGTTGGGTTACC
ATTCTGCGTAGCCTGGAACCGGGTCTGGAAGTTCTGCGCGAAGGTGATTGGCT

GCCGGTTAGCCCGCGTCCGGGTGAATTTATTGTTAATTTTGGTTGTGCGATGGA
AATTCTGACCCGTCATTCAGCAACTCCTGTTGCAGCAGTGGCACATCGTGTTC
AGGAACAGCTGCCTGGTCAGGCCGATCGCTTTAGTTATGCCCTGTTTGTTGATA
GCTCACTGGATCCTCGTACCTGTCCTGGTCTGTTTCGTTATCTGCCGGGTCATG
GCCTGGTTCTGGAAGCAGATTTTGAAATGTTTCTGAATGAAATCCTGCATAATA
CCTATCAGGAAAATACTCAGGGTCTGTAT

Amino acid sequence of PaBcmG

MNPNATYALASAELIDGKLRFDSSDGFARAIADGFFFVKSPSLDLAAGDTFARNF
YLPRQEGLGAPYQGFSQWTEDRLARREGYFSRDVDQVEQFFLESRFWQTVFPGP
LLRQAERMRSFSLEVLRAVLAELDLPVELWDEATGRCLSMQGTYHLTFNHFRSH
VRARGLNVHKDSGWVTILRSLEPGLEVLREGDWLPVSPRPGEFIVNFGCAMEILT
RHSATPVAAVAHRVQEQLPGQADRFSYALFVDSSLDPRTCPGLFRYLPGHGLVL
EADFEMFLNEILHNTYQENTQGLY

Supplementary Methods

Enzymatic syntheses

Enzymatic synthesis of compound 1a

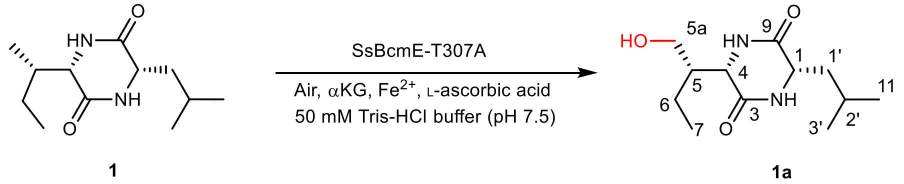

The enzymatic reaction contained 50 mM Tris-HCl buffer (pH 7.5), 1.2 mM **1** (14 mg dissolved in 1.5 mL DMSO, 62 μmol), 3 mM αKG, 3 mM L-ascorbic acid, 0.1 mM $FeSO_4 \cdot 7H_2O$, and 14 μM mutant SsBcmE-T307A in a total volume of 50 mL. The reaction mixtures were incubated at 16 °C for 12 h. The resulting mixture was then extracted with ethyl acetate (100 mL×3). The combined organic phases were concentrated under reduced pressure, and purified by reverse-phase semi-preparative HPLC to give **1a** (3.7 mg, 25%). **1a** ^{1}**H NMR** (600 MHz, CD_3OD): δ 4.28 (1H, dd, *J* = 3.0, 1.7 Hz, H-4), 4.01 (1H, ddd, *J* = 8.4, 4.6, 1.6 Hz, H-1), 3.73 (1H, dd, *J* = 11.2, 4.3 Hz, H5a), 3.67 (1H, dd, *J* = 11.2, 6.5 Hz, H5a), 2.14 – 2.04 (1H, m, H-5), 1.93 – 1.82 (1H, m, H-2'), 1.77 (1H, ddd, *J* = 13.5, 8.7, 4.6 Hz, H-1'), 1.63 (1H, ddd, *J* = 13.8, 8.4, 5.4 Hz, H-1'), 1.48 – 1.29 (2H, m, H-6), 0.99 – 0.93 (9H, m, H-7, 11, 3'). ^{13}**C NMR** (150 MHz, CD_3OD): δ 171.0 (C-9), 170.0 (C-3), 62.6

(C-5a), 58.3 (C-4), 54.1 (C-1), 45.8 (C-5), 44.8 (C-1'), 25.2 (C-2'), 23.5 (C-3'), 22.1 (C-11), 20.0 (C-6), 12.2 (C-7). **HR-ESI-MS** (positive): *m*/*z* calculated for $C_{12}H_{23}N_2O_3$ $[M+H]^+$ 243.1703, found 243.1706.

Enzymatic synthesis of compound 1c

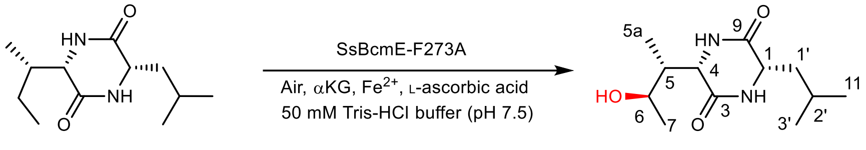

1 **1c**

The enzymatic reaction contained 50 mM Tris-HCl buffer (pH 7.5), 1 mM **1** (11.3 mg dissolved in 1.25 mL DMSO, 50 µmol), 3 mM αKG, 3 mM L-ascorbic acid, 0.1 mM $FeSO_4 \cdot 7H_2O$, and 14 µM mutant SsBcmE-F273A in a total volume of 50 mL. The reaction mixtures were incubated at 16 °C for 12 h. The resulting mixture was then extracted with ethyl acetate (100 mL×3). The combined organic phases were concentrated under reduced pressure, and purified by reverse-phase semi-preparative HPLC to give **1c** (5.5 mg, 46%). **1c 1H NMR** (600 MHz, CD_3OD): δ 4.06 (1H, dd, *J* = 4.7, 1.4 Hz, H-4), 4.05 – 4.00 (1H, m, H-6), 3.96 (1H, ddd, *J* = 8.9, 4.5, 1.4 Hz, H-1), 1.90 – 1.83 (1H, m, H-2'), 1.82 – 1.78 (1H, m, H-5), 1.75 (1H, td, *J* = 9.0, 4.4 Hz, H-1'), 1.62 (1H, ddd, *J* = 13.9, 8.9, 5.3 Hz, H-1'), 1.22 (3H, d, *J* = 6.4 Hz, H-7), 1.01 (3H, d, *J* = 7.1 Hz, H5a), 0.97 (3H, d, *J* = 6.6 Hz, H-3'), 0.96 (3H, d, *J* = 6.6 Hz, H-11). **^{13}C NMR** (150 MHz, CD_3OD): δ 171.1 (C-9), 170.0 (C-3), 68.9 (C-6), 60.0 (C-4), 54.3 (C-1), 45.7 (C-5), 45.1 (C-1'), 25.3 (C-2'), 23.6 (C-3'), 22.0 (C-11), 21.5 (C-7), 8.9 (C-5a). **HR-ESI-MS** (positive): *m*/*z* calculated for $C_{12}H_{22}N_2O_3Na$ $[M+H]^+$ 265.1523, found 265.1522.

Enzymatic synthesis of compound 2a

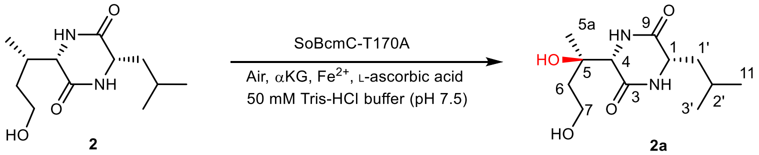

The enzymatic reaction contained 50 mM Tris-HCl buffer (pH 7.5), 0.9 mM **2** (9 mg dissolved in 0.12 mL DMSO, 37 µmol), 3 mM αKG, 3 mM L-ascorbic acid, 0.1 mM $FeSO_4 \cdot 7H_2O$, and 38 µM mutant SoBcmC-T170A in a total volume of 40 mL. The reaction mixtures were incubated at 37 °C overnight and terminated by the addition of 80 mL of pre-cooled methanol. After centrifugation at 13,800 g for 30 min, the supernatant was

concentrated under reduced pressure, and purified by reverse-phase semi-preparative HPLC to give **2a** (1.7 mg, 18 %).

2a ^{1}H NMR (600 MHz, CD_3OD): δ 3.92 (1H, ddd, *J* = 9.5, 4.2, 1.1 Hz, H-1), 3.88 (1H, s, H-4), 3.79 (1H, dt, *J* = 11.4, 5.8 Hz, H-7), 3.73 (1H, ddd, *J* = 10.9, 7.9, 5.3 Hz, H-7), 1.95 (1H, ddd, *J* = 14.0, 7.9, 5.8 Hz, H-6), 1.89 – 1.85 (1H, m, H-1'), 1.85 – 1.83 (1H, m, H-2'), 1.83 – 1.78 (1H, m, H-6), 1.77 – 1.69 (1H, m, H-1'), 1.33 (3H, s, H-5a), 0.96 (3H, d, *J* = 6.4 Hz, H-3'), 0.94 (3H, d, *J* = 6.4 Hz, H-11). **^{13}C NMR** (150 MHz, CD_3OD): δ 171.6 (C-9), 168.3 (C-3), 74.6 (C-5), 63.4 (C-4), 58.9 (C-7), 54.7 (C-1), 45.5 (C-1'), 42.4 (C-6), 25.2 (C-2'), 24.1 (C-5a), 23.7 (C-3'), 21.7 (C-11). **HR-ESI-MS** (positive): *m*/*z* calculated for $C_{12}H_{22}N_2O_4Na$ $[M+H]^+$ 281.1472, found 281.1473.

Enzymatic synthesis of compound 2b

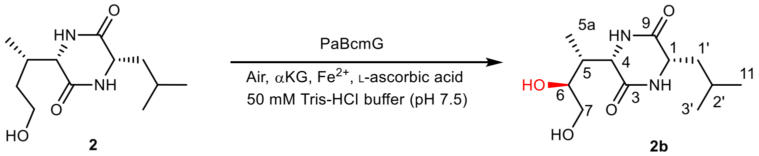

The enzymatic reaction contained 50 mM Tris-HCl buffer (pH 7.5), 0.6 mM **2** (8.7 mg dissolved in 0.12 mL DMSO, 36 μmol), 2 mM αKG, 2 mM L-ascorbic acid, 50 μM $FeSO_4 \cdot 7H_2O$, and 14.9 μM PaBcmG in a total volume of 60 mL. The reaction mixtures were incubated at 37 °C for 12 h. The reaction mixtures were incubated at 37 °C for 12 h and terminated by the addition of 120 mL of pre-cooled methanol. After centrifugation at 13,800 g for 30 min, the supernatant was concentrated under reduced pressure, and purified by reverse-phase semi-preparative HPLC to give **2b** (5.9 mg, 64%).

2b ^{1}H NMR (600 MHz, CD_3OD): δ 4.29 (1H, dd, *J* = 3.3, 1.5 Hz, H-4), 3.97 (1H, ddd, *J* = 8.9, 4.5, 1.6 Hz, H-1), 3.73 (1H, ddd, *J* = 8.7, 5.5, 3.7 Hz, H-6), 3.66 (1H, dd, *J* = 11.5, 3.7 Hz, H-7), 3.54 (1H, dd, *J* = 11.5, 5.5 Hz, H-7), 2.19 – 2.09 (1H, m, H-5), 1.87 (1H, dtd, *J* = 8.9, 6.6, 5.2 Hz, H-2'), 1.77 (1H, ddd, *J* = 13.6, 9.0, 4.5 Hz, H-1'), 1.69 (1H, ddd, *J* = 13.9, 8.9, 5.2 Hz, H-1'), 1.01 (3H, d, *J* = 7.1 Hz, H5a), 0.97 (3H, d, *J* = 6.6 Hz, H-3'), 0.95 (3H, d, *J* = 6.5 Hz, H-11). **^{13}C NMR** (150 MHz, CD_3OD): δ 171.2 (C-9), 169.7 (C-3), 74.3 (C-6), 65.3 (C-7), 57.5 (C-4), 54.2 (C-1), 45.0 (C-1'), 41.4 (C-5), 25.3 (C-2'), 23.6 (C-3'), 21.9 (C-11), 12.9 (C-5a). **HR-ESI-MS** (positive): *m*/*z* calculated for $C_{12}H_{23}N_2O_4$ $[M+H]^+$ 259.1652, found 259.1655.

Enzymatic synthesis of compounds 3a–3c

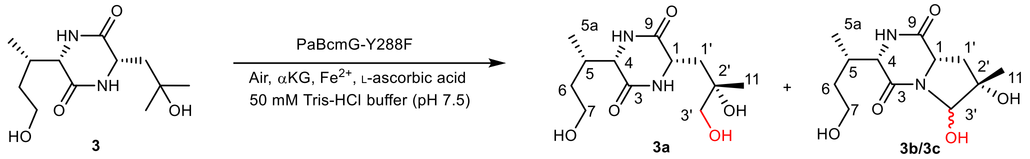

The enzymatic reaction contained 50 mM Tris-HCl buffer (pH 7.5), 0.8 mM **3** (7.2 mg dissolved in 0.1 mL DMSO, 28 μmol), 2 mM αKG, 2 mM L-ascorbic acid, 0.1 mM $FeSO_4 \cdot 7H_2O$, and 10 μM mutant PaBcmG-Y288F in a total volume of 35 mL. The reaction mixtures were incubated at 37 °C overnight and terminated by the addition of 70 mL of pre-cooled methanol. After centrifugation at 13,800 g for 30 min, the supernatant was concentrated under reduced pressure, and purified by reverse-phase semi-preparative HPLC to give **3a** (3.2 mg, 42 %) and gave **3b**/**3c** (2.4 mg, 32%). It should be noted that acid was not included in the mobile phases to prevent the rearrangement of **3b** and **3c**. Nevertheless, we found that the purified compound contains both diastereomers **3b** and **3c**.

3a ^{1}H NMR (600 MHz, CD_3OD): δ 4.31 (1H, ddd, J = 9.1, 3.5, 1.6 Hz, H-1), 3.95 (1H, dd, *J* = 3.4, 1.6 Hz, H-4), 3.66 (1H, ddd, *J* = 10.9, 7.3, 5.4 Hz, H-7), 3.58 (1H, dt, *J* = 10.9, 7.1 Hz, H-7), 3.48 (1H, d, *J* = 11.2 Hz, H-3'), 3.41 (1H, d, *J* = 11.3 Hz, H-3'), 2.33 (1H, dd, *J* = 14.6, 3.5 Hz, H-1'), 2.29 – 2.14 (1H, m, H-5), 1.80 – 1.68 (2H, m, H-6, 1'), 1.56 – 1.41 (1H, m, H-6), 1.24 (3H, s, H-11), 1.05 (3H, d, *J* = 7.1 Hz, H-5a). **^{13}C NMR** (150 MHz, CD_3OD): δ 171.3 (C-9), 168.9 (C-3), 73.2 (C-2'), 69.3 (C-3'), 60.7 (C-7), 60.5 (C-4), 53.1 (C-1), 43.7 (C-1'), 35.7 (C-6), 35.0 (C-5), 26.1 (C-11), 15.8 (C-5a). **HR-ESI-MS** (positive): *m*/*z* calculated for $C_{12}H_{22}N_2O_5Na$ $[M+H]^+$ 297.1421, found 297.1424.

3b/3c ^{13}C NMR (150 MHz, CD_3OD): δ 173.5, 173.3, 170.0, 169.0, 87.9, 84.0, 78.4, 75.5, 61.2, 61.2, 60.8, 60.7, 57.9, 57.1, 38.5, 38.4, 35.6, 35.5, 31.2, 31.0, 24.2, 21.9, 16.3, 16.2. **3b HR-ESI-MS** (positive): *m*/*z* calculated for $C_{12}H_{20}N_2O_5Na$ $[M+H]^+$ 295.1264, found 295.1264. **3c HR-ESI-MS** (positive): *m*/*z* calculated for $C_{12}H_{20}N_2O_5Na$ $[M+H]^+$ 295.1264, found 295.1267.

Enzymatic synthesis of compound 5a

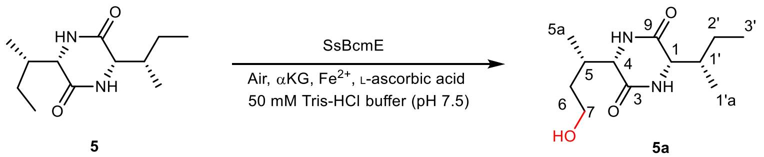

5 5a

The enzymatic reaction contained 50 mM Tris-HCl buffer (pH 7.5), 1 mM **5** (18 mg dissolved in 2.0 mL DMSO, 79.6 µmol), 3 mM αKG, 3 mM L-ascorbic acid, 0.1 mM $FeSO_4 \cdot 7H_2O$, and 12 µM SsBcmE in a total volume of 80 mL. The reaction mixtures were incubated at 16 °C for 12 h and terminated by the addition of 160 mL of pre-cooled methanol. After centrifugation at 13,800 g for 30 min, the supernatant was concentrated under reduced pressure, and purified by reverse-phase semi-preparative HPLC to give **5a** (6.9 mg, 36 %).

5a ^{1}H NMR (600 MHz, CD_3OD): δ 3.93 (1H, dd, *J* = 3.9, 1.9 Hz, H-4), 3.91 (1H, dd, *J* = 3.8, 1.9 Hz, H-1), 3.65 (1H, ddd, *J* = 10.8, 7.2, 5.4 Hz, H-7), 3.56 (1H, ddd, *J* = 10.9, 7.6, 6.6 Hz, H-7), 2.28 – 2.18 (1H, m, H-5), 2.06 – 1.95 (1H, m, H-1'), 1.80 – 1.67 (1H, m, H-6), 1.58 – 1.44 (2H, m, H-6, 2'), 1.37 – 1.18 (1H, m, H-2'), 1.06 (3H, d, *J* = 7.1 Hz, H-5a), 1.04 (3H, d, *J* = 7.1 Hz, H-1'a), 0.94 (3H, t, *J* = 7.4 Hz, H-3'). **^{13}C NMR** (150 MHz, CD_3OD): δ 170.2 (C-9), 170.0 (C-3), 60.7 (C-7), 60.6 (C-4), 60.6 (C-1), 39.9 (C-1'), 36.1 (C-6), 35.0 (C-5), 26.0 (C-2'), 16.0 (C-5a), 15.6 (C-1'a), 12.2 (C-3'). **HR-ESI-MS** (positive): *m/z* calculated for $C_{12}H_{22}N_2O_3Na$ $[M+H]^+$ 265.1523, found 265.1528.

Enzymatic synthesis of compound 6a

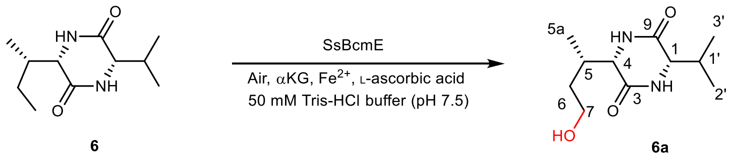

6 6a

The enzymatic reaction contained 50 mM Tris-HCl buffer (pH 7.5), 1 mM **6** (15 mg dissolved in 1.2 mL DMSO, 70.8 µmol), 3 mM αKG, 3 mM L-ascorbic acid, 0.1 mM $FeSO_4 \cdot 7H_2O$, and 10 µM SsBcmE in a total volume of 70 mL. The reaction mixtures were incubated at 16 °C for 12 h and terminated by the addition of 140 mL of pre-cooled methanol. After centrifugation at 13,800 g for 30 min, the supernatant was concentrated under reduced pressure, and purified by reverse-phase semi-preparative HPLC to give **6a** (1.6 mg, 10%).

6a ^{1}H NMR (600 MHz, CD_3OD): δ 3.93 (1H, dd, *J* = 4.0, 1.8 Hz, H-4), 3.84 (1H, dd, *J* = 4.1, 1.8 Hz, H-1), 3.65 (1H, ddd, *J* = 11.0, 7.0, 5.5 Hz, H-7), 3.57 (1H, dt, *J* = 10.9, 7.1 Hz, H-7), 2.41 – 2.27 (1H, m, H-1'), 2.26 – 2.17 (1H, m, H-5), 1.75 (1H, ddt, *J* = 14.4, 7.4, 3.7 Hz, H-6), 1.50 (1H, ddt, *J* = 13.6, 9.6, 6.4 Hz, H-6), 1.06 (6H, dd, *J* = 7.1, 2.8 Hz, H-5a, 2'), 0.96 (3H, d, *J* = 6.8 Hz, H-3'). **^{13}C NMR** (150 MHz, CD_3OD): δ 170.1 (C-9), 170.1 (C-3), 61.1 (C-1), 60.7 (C-7), 60.7 (C-4), 36.1 (C-6), 35.1 (C-5), 33.1 (C-1'), 19.3 (C-2'), 17.8 (C-3'), 16.0 (C-5a). **HR-ESI-MS** (positive): *m*/*z* calculated for $C_{11}H_{20}N_2O_3Na$ $[M+H]^+$ 251.1366, found 251.1370.

Enzymatic synthesis of compound 7a

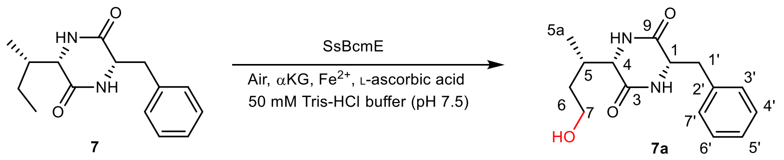

The enzymatic reaction contained 50 mM Tris-HCl buffer (pH 7.5), 1 mM **7** (17 mg dissolved in 1.6 mL DMSO, 65.4 µmol), 3 mM αKG, 3 mM L-ascorbic acid, 0.1 mM $FeSO_4·7H_2O$, and 19 µM SsBcmE in a total volume of 65 mL. The reaction mixtures were incubated at 16 °C for 16 h and terminated by the addition of 130 mL of pre-cooled methanol. After centrifugation at 13,800 g for 30 min, the supernatant was concentrated under reduced pressure, and purified by reverse-phase semi-preparative HPLC to give **7a** (2.1 mg, 12%).

7a ^{1}H NMR (500 MHz, CD_3OD): δ 7.31 – 7.26 (2H, m, H-4', 6'), 7.25 – 7.20 (3H, m, H-3', 5', 7'), 4.32 (1H, td, *J* = 5.0, 1.7 Hz, H-1), 3.79 (1H, dd, *J* = 4.1, 1.7 Hz, H-4), 3.46 – 3.40 (1H, m, H-7), 3.40 – 3.33 (1H, m, H-7), 3.25 (1H, dd, *J* = 13.8, 5.3 Hz, H-1'), 3.03 (1H, dd, *J* = 13.8, 4.7 Hz, H-1'), 1.79 – 1.70 (1H, m, H-5), 1.21 – 1.11 (1H, m, H-6), 1.04 – 0.94 (1H, m, H-6), 0.71 (1H, d, *J* = 7.1 Hz, H-5a). **^{13}C NMR** (125 MHz, CD_3OD): δ 169.2 (C-9), 169.2 (C-3), 137.1 (C-2'), 131.5 (C-3'), 131.5 (C-7'), 129.6 (C-4'), 129.6 (C-6'), 128.2 (C-5'), 60.7 (C-4), 60.5 (C-7), 57.2 (C-1), 39.9 (C-1'), 35.1 (C-5), 35.0 (C-6), 15.4 (C-5a). **HR-ESI-MS** (positive): *m*/*z* calculated for $C_{15}H_{20}N_2O_3Na$ $[M+H]^+$ 299.1366, found 299.1369.

Enzymatic synthesis of compounds 8a and 8b

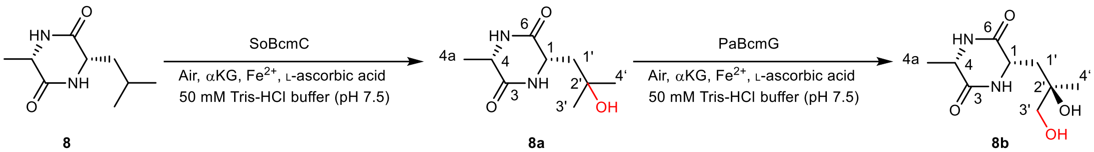

8 **8a** **8b**

The enzymatic reaction contained 50 mM Tris-HCl buffer (pH 7.5), 1 mM **8** (7.3 mg dissolved in 0.7 mL DMSO, 39.7 μmol), 4 mM αKG, 4 mM L-ascorbic acid, 0.1 mM $FeSO_4 \cdot 7H_2O$, and 5.9 μM SoBcmC in a total volume of 40 mL. The reaction mixtures were incubated at 37 °C for 12 h. Then, 2.9 μM PaBcmG was added to the resulting mixtures and the reaction continued at 37 °C for 2 h. The reaction was terminated by the addition of 80 mL of pre-cooled methanol. After centrifugation at 13,800 g for 30 min, the supernatant was concentrated under reduced pressure, and purified by reverse-phase semi-preparative HPLC to give **8a** (1.8 mg, 23%) and **8b** (1.6 mg, 19%).

8a **^{1}H NMR** (600 MHz, CD_3OD): δ 4.28 (1H, ddd, J = 9.9, 3.0, 1.6 Hz, H-1), 4.08 (1H, qd, J = 7.0, 1.6 Hz, H-4), 2.16 (1H, dd, J = 14.5, 3.0 Hz, H-1'), 1.77 (1H, dd, J = 14.5, 10.0 Hz, H-1'), 1.41 (3H, d, J = 7.0 Hz, H-4a), 1.29 (3H s, H-3'), 1.28 (3H s, H-4'). **^{13}C NMR** (150 MHz, CD3OD): δ 171.4 (C-6), 171.4 (C-3), 71.3 (C-2'), 53.7 (C-1), 51.3 (C-4), 45.5 (C-1'), 31.6 (C-3'), 27.9 (C-4'), 18.8 (C-4a). **HR-ESI-MS** (positive): m/z calculated for $C_{15}H_{20}N_2O_3Na$ $[M+H]^+$ 299.1366, found 299.1369.

8b **^{1}H NMR** (600 MHz, CD_3OD): δ 4.31 (1H, ddd, J = 10.1, 2.7, 1.6 Hz, H-1), 4.08 (1H, qd, J = 7.0, 1.6 Hz, H-4), 3.42 – 3.36 (2H, m, H-3'), 2.17 (1H, dd, J = 14.6, 2.8 Hz, H-1'), 1.82 (1H, dd, J = 14.6, 10.1 Hz, H-1'), 1.42 (3H, d, J = 7.0 Hz, H-4a), 1.23 (3H s, H-4'). **^{13}C NMR** (150 MHz, CD_3OD): δ 171.5 (C-6), 171.5 (C-3), 73.5 (C-2'), 71.2 (C-3'), 53.2 (C-1), 51.3 (C-4), 41.3 (C-1'), 23.7 (C-4'), 18.9 (C-4a). **HR-ESI-MS** (positive): m/z calculated for $C_9H_{16}N_2O_4Na$ $[M+H]^+$ 239.1002, found 239.1004.

Enzymatic synthesis of compound 9a

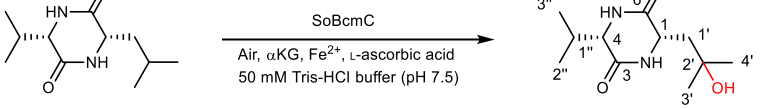

9 **9a**

The enzymatic reaction contained 50 mM Tris-HCl buffer (pH 7.5), 1 mM **9** (16 mg dissolved in 1.2 mL DMSO, 75.5 μmol), 3 mM αKG, 3 mM L-ascorbic acid, 0.1 mM

$FeSO_4 \cdot 7H_2O$, and 5.8 μM SoBcmC in a total volume of 70 mL. The reaction mixtures were incubated at 37 °C for 12 h and terminated by the addition of 140 mL of pre-cooled methanol. After centrifugation at 13,800 g for 30 min, the supernatant was concentrated under reduced pressure, and purified by reverse-phase semi-preparative HPLC to give **9a** (12.9 mg, 75%).

9a **^{1}H NMR** (600 MHz, CD_3OD): δ 4.27 (1H, ddd, J = 9.7, 2.9, 1.6 Hz, H-1), 3.87 (1H, dd, J = 3.4, 1.6 Hz, H-4), 2.39 – 2.24 (1H, m, H-1''), 2.15 (1H, dd, J = 14.3, 2.9 Hz, H-1'), 1.77 (1H, dd, J = 14.3, 9.7 Hz, H-1'), 1.29 (3H, s, H-3'), 1.28 (3H, s, H-4'), 1.05 (3H, d, J = 7.1 Hz, H-2''), 0.95 (3H, d, J = 6.8 Hz, H-3''). **^{13}C NMR** (150 MHz, CD_3OD): δ 171.2 (C-6), 169.1 (C-3), 71.3 (C-2'), 60.9 (C-4), 53.6 (C-1), 47.4 (C-1'), 33.0 (C-1''), 31.5 (C-3'), 28.1 (C-4'), 19.0 (C-2''), 17.1 (C-3''). **HR-ESI-MS** (positive): m/z calculated for $C_{11}H_{20}N_2O_3Na$ $[M+H]^+$ 251.1366, found 251.1372.

Enzymatic synthesis of compound 9b

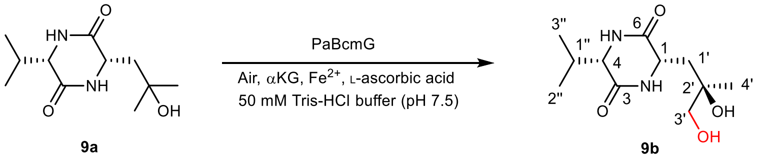

9a 9b

The enzymatic reaction contained 50 mM Tris-HCl buffer (pH 7.5), 1 mM **9a** (9.1 mg dissolved in 0.2 mL DMSO, 39.9 μmol), 3 mM αKG, 3 mM L-ascorbic acid, 0.1 mM $FeSO_4 \cdot 7H_2O$, and 5.8 μM PaBcmG in a total volume of 40 mL. The reaction mixtures were incubated at 37 °C for 6 h and terminated by the addition of 80 mL of pre-cooled methanol. After centrifugation at 13,800 g for 30 min, the supernatant was concentrated under reduced pressure, and purified by reverse-phase semi-preparative HPLC to give **9b** (7.6 mg, 78%).

9b **^{1}H NMR** (600 MHz, CD_3OD): δ 4.34 – 4.28 (1H, m, H-1), 3.87 (1H, dd, J = 3.3, 1.5 Hz, H-4), 3.44 – 3.35 (2H, m, H-3'), 2.36 – 2.24 (1H, m, H-1''), 2.15 (1H, dd, J = 14.4, 2.6 Hz, H-1'), 1.84 (1H, dd, J = 14.4, 9.7 Hz, H-1'), 1.22 (3H, s, H-4'), 1.05 (3H, d, J = 7.1 Hz, H-2''), 0.95 (3H, d, J = 6.8 Hz, H-3''). **^{13}C NMR** (150 MHz, CD_3OD): δ 171.3 (C-6), 169.1 (C-3), 73.5 (C-2'), 71.2 (C-3'), 60.9 (C-4), 53.0 (C-1), 43.4 (C-1'), 33.1 (C-1''), 23.9 (C-4'), 19.0 (C-2''), 17.1 (C-3''). **HR-ESI-MS** (positive): m/z calculated for $C_{11}H_{20}N_2O_4Na$ $[M+H]^+$ 267.1315, found 267.1320.

Enzymatic synthesis of compound 10a

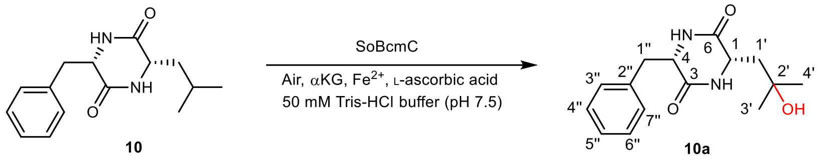

The enzymatic reaction contained 50 mM Tris-HCl buffer (pH 7.5), 1 mM **10** (17 mg dissolved in 1.6 mL DMSO, 65.4 μmol), 3 mM αKG, 3 mM L-ascorbic acid, 0.1 mM $FeSO_4·7H_2O$, and 21.4 μM SoBcmC in a total volume of 65 mL. The reaction mixtures were incubated at 37 °C for 16 h and terminated by the addition of 130 mL of pre-cooled methanol. After centrifugation at 13,800 g for 30 min, the supernatant was concentrated under reduced pressure, and purified by reverse-phase semi-preparative HPLC to give **10a** (4.3 mg, 24 %).

10a ^{1}H NMR (600 MHz, CD_3OD): δ 7.36 – 7.30 (2H, m, H-4", 6"), 7.29 – 7.25 (1H, m, H-5"), 7.22 – 7.19 (2H, m, H-3", 7"), 4.32 (1H, ddd, *J* = 4.9, 3.9, 1.4 Hz, H-4), 3.99 (1H, ddd, *J* = 9.4, 2.9, 1.4 Hz, H-1), 3.28 (1H, dd, *J* = 13.8, 3.8 Hz, H-1"), 2.97 (1H, dd, *J* = 13.8, 4.7 Hz, H-1"), 1.32 (1H, dd, *J* = 14.4, 2.9 Hz, H-1'), 1.08 (3H, s, H-4'), 1.04 (3H, s, H-3'), 0.36 (1H, dd, *J* = 14.4, 9.4 Hz, H-1'). **^{13}C NMR** (150 MHz, CD_3OD): δ 170.6 (C-6), 168.3 (C-3), 136.5 (C-2"), 131.9 (C-3"), 131.9 (C-7"), 129.7 (C-4"), 129.7 (C-6"), 128.4 (C-5"), 70.9 (C-2'), 57.0 (C-4), 53.6 (C-1), 47.0 (C-1'), 40.2 (C-1"), 31.0 (C-3'), 28.4 (C-4'). **HR-ESI-MS** (positive): *m/z* calculated for $C_{15}H_{20}N_2O_3Na$ $[M+H]^+$ 299.1366, found 299.1364.

Enzymatic synthesis of compound 10b

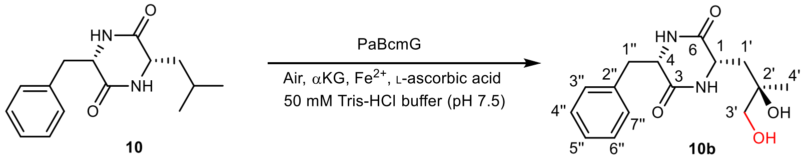

The enzymatic reaction contained 50 mM Tris-HCl buffer (pH 7.5), 1 mM **10** (13 mg dissolved in 1.0 mL DMSO, 50 μmol), 3 mM αKG, 3 mM L-ascorbic acid, 0.1 mM $FeSO_4·7H_2O$, and 32.7 μM PaBcmG in a total volume of 50 mL. The reaction mixtures were incubated at 37 °C for 12 h and terminated by the addition of 100 mL of pre-cooled methanol. After centrifugation at 13,800 g for 30 min, the supernatant was concentrated under reduced pressure, and purified by reverse-phase semi-preparative HPLC to give **10b**

(1.8 mg, 12%).

^{1}**H NMR** (600 MHz, CD_3OD): δ 7.31 (2H, dd, *J* = 8.0, 6.6 Hz, H-4'', 6''), 7.29 – 7.23 (1H, m, H-5''), 7.20 (2H, dd, *J* = 7.0, 1.8 Hz, H-3'', 7''), 4.32 (1H, td, *J* = 4.4, 4.0, 1.4 Hz, H-4), 4.03 (1H, ddd, *J* = 9.5, 2.8, 1.4 Hz, H-1), 3.27 (1H, dd, *J* = 13.7, 3.9 Hz, H-1''), 3.20 (1H, d, *J* = 11.1 Hz, H-3'), 3.11 (1H, d, *J* = 11.0 Hz, H-3'), 2.97 (1H, dd, *J* = 13.7, 4.7 Hz, H-1''), 1.38 (1H, dd, *J* = 14.6, 2.7 Hz, H-1'), 1.00 (3H, s, H-4'), 0.32 (1H, dd, *J* = 14.6, 9.5 Hz, H-1'). ^{13}**C NMR** (150 MHz, CD_3OD): δ 170.7 (C-6), 168.4 (C-3), 136.6 (C-2''), 131.9 (C-3''), 131.9 (C-7''), 129.7 (C-4''), 129.7 (C-6''), 128.4 (C-5''), 72.7 (C-2'), 70.6 (C-3'), 57.0 (C-4), 52.9 (C-1), 43.7 (C-1'), 40.2 (C-1''), 24.5 (C-4'). **HR-ESI-MS** (positive): *m*/*z* calculated for $C_{15}H_{20}N_2O_4Na$ $[M+H]^+$ 315.1315, found 315.1318.

Computational details

Quantum chemical model constructions

To reveal the inherent site selectivity of hydroxylation, we conducted density functional theory (DFT) calculations on the cognate substrates (**1**–**3**) catalyzed by a truncated catalytic-residue theozyme model. For SsBcmE, the model contains Fe^{IV}-oxo, two methylimidazoles for H195 and H253, two acetate anions for D197 and succinate, and a ligand water molecule. For SoBcmC, the model contains Fe^{IV}-oxo, two methylimidazoles for H167 and H225, two acetate anions for D169 and succinate, and a ligand water molecule. For PaBcmG, the model contains Fe^{IV}-oxo, two methylimidazoles for H172 and H230, two acetate anions for D174 and succinate, and a ligand water molecule. These models were set at the quintet spin state according to previous studies of Fe^{II}/αKG-dependent dioxygenases[1-3].

Molecular dynamics initial structural preparations

On the basis of the crystal structures of $SsBcmE^{T307A}$•Fe^{II}•αKG•**1**, SoBcmC•Fe^{II}•αKG•**2** and PaBcmG•Fe^{II}•αKG•**3** (PDB code: 8XHY, 8XHQ and 8XHX, respectively), Fe(II) was replaced by Fe^{IV}-oxo and αKG with succinate, as well as one water molecule was added to create SsBcmE•Fe^{IV}-oxo•succinate•**1**, SoBcmC•Fe^{IV}-oxo•succinate•**2** and PaBcmG•Fe^{IV}-oxo•succinate•**3** complex structures, which are similar to the proposed hexa-coordinate high-valent oxidant reported in the previous studies[2,4]. A representative MD pre-equilibrated SsBcmE•Fe^{IV}-oxo•succinate•**1** complex structure was used as the template for

creating the starting coordinates for three mutants F273A, T307L and T307A complexes with changing into the corresponding mutant residues. The protonation states of charged residues were determined at constant pH 7.5 based on pKa calculations via the PROPKA program[5] and the consideration of the local hydrogen bonding network. In SsBcmE wild type and mutants' systems, H22, H81, H98, H113, H154, H161, H182 and H186 were assigned as HIE; H79, H195, H228 and H253 were assigned as HID; and H175 was assigned as HIP. In SoBcmC wild-type system, H60, H160, and H285 were assigned as HIE; H89, H167 and H225 were assigned as HID; and H154 was assigned as HIP. In PaBcmG wild type system, H163, H220, H268 and H285 were assigned as HIE; H172 and H230 were assigned as HID; as well as H154 and H159 were assigned as HIP. In all the above systems, all D and E residues were deprotonated, while K and R were protonated.

Supplementary Tables

Supplementary Table 1. Data collection and refinement statistics.

	**SsBcmE^{T307A}•Fe^{II}•1**	**SsBcmC•Fe^{II}•αKG**	**SoBcmC•Fe^{II}•αKG•2**	**PaBcmG•Fe^{II}•αKG**	**PaBcmG•Fe^{II}•αKG•3**
**PDB code**	**8XHY**	**8XHP**	**8XHQ**	**8XHT**	**8XHX**
**Data collection**					
Space group	$P2_12_12_1$	$C2_1$	$P2_1$	$P2_1$	$P2_12_12_1$
Cell dimensions					
a, b, c (Å)	47.32, 81.97, 82.15	128.97, 74.23, 98.05	51.19, 142.99, 85.44	72.33, 103.40, 91.07	43.83, 77.51, 86.64
α, β, γ (◦)	90.00, 90.00, 90.00	90.00, 104.13, 90.00	90.00, 92.90, 90.00	90.00, 89.97, 90.00	90.00, 90.00, 90.00
Resolution (Å)	23.70–1.71 (1.77–1.71)	19.77–1.86 (1.90–1.86)	47.66–1.90 (1.93–1.90)	50.00–1.82 (1.85–1.82)	19.57-1.61 (1.64-1.61)
R_{pim}	0.045 (0.406)	0.072 (0.420)	0.054 (0.324)	0.047 (0.205)	0.026 (0.463)
R_{merge}	0.155 (1.414)	0.163 (0.973)	0.124 (0.767)	0.113 (0.469)	0.090 (1.634)
CC1/2 [a]	0.999 (0.851)	0.987 (0.682)	0.995 (0.820)	0.989 (0.940)	0.999 (0.629)
No. of unique reflections	35306	75346	96038	118876	39138
$I/\sigma I$	12.9 (2.2)	6.3 (1.6)	10.8 (3.0)	14.4 (3.0)	20.3 (1.7)
Completeness (%) [a]	100.0 (100.0)	99.9 (100.0)	99.8 (99.7)	99.1 (92.0)	99.9 (100.0)
Redundancy	13.0 (12.9)	6.1 (6.3)	6.3 (6.5)	6.6 (5.6)	12.9 (13.3)
**Refinement**					
Resolution (Å)	23.71–1.71 (1.76–1.71)	19.77–1.86 (1.93–1.86)	42.91–1.90 (1.97–1.90)	42.05–1.82 (1.84–1.82)	19.57-1.61 (1.69-1.61)
No. reflections	35232 (1727)	75346 (7482)	96012 (9553)	102881 (5133)	39078 (3846)
R_{work} / R_{free}	0.1605/0.2050	0.2054/0.2297	0.1975/ 0.2247	0.1992/0.2356	0.1561/0.1824
No. atoms					
Protein	2436	6398	9071	9280	2377
Ligand	39	45	114	62	34
Water	387	471	582	961	363
Wilson B factors (Å^2)	17.79	25.92	23.25	18.44	19.36
Average B factors (Å^2)	22.74	31.19	24.78	24.77	22.41
Protein	21.49	30.66	24.78	24.28	20.68
Ligand/ion	21.55	27.47	13.69	32.89	24.35
Water	30.73	36.69	27.02	28.99	33.54
R.m.s. deviations					
bond lengths (Å)	0.012	0.004	0.007	0.006	0.006
bond angles (˚)	1.140	0.730	0.920	0.890	0.860
Ramachandran Outliers (%)	0.40	0.00	0.26	0.26	0.00
Ramachandran Favored (%)	98.03	98.15	98.95	96.05	97.60

[a] Statistics for the highest-resolution shell are shown in parentheses.

Supplementary Table 2. Primers used in the study of BcmE.

Name	Sequence (5'→3')
SsBcmE-Y105A-f	GCGTGCCCGGCGACGCCCAGGGCTAC
SsBcmE-Y105A-r	TCGAAGTAGCCCTGGGCGTCGCCGGG
SsBcmE-Y120F-f	TGGGAGAACTTCTTCGTCGAGAGG
SsBcmE-Y120F-r	CCAGTTGTCCCTCTCGACGAAGAAGTTCTC
SsBcmE-Y120A-f	GTGGGAGAACTTCGCCGTCGAGAGG
SsBcmE-Y120A-r	CAGTTGTCCCTCTCGACGGCGAAGTTCTCC
SsBcmE-M177A-f	GGACCGCGGCCACCAGGCGCTCGCCTT
SsBcmE-M177A-r	GAAGTGGTTGAAGGCGAGCGCCTGGTGGCCGCG
SsBcmE-V304A-f	CGCCGGCCAGGAAGCCGGCCGGACCT
SsBcmE-V304A-r	GTCGTAGGTCCGGCCGGCTTCCTGGC
SsBcmE-T307A-f	GCCAGGAAGTCGGCCGGGCCTACGACGACA
SsBcmE-T307A-r	GCACCGCTGTCGTCGTAGGCCCGGCCGACT
SsBcmE-Y308A-f	AGGAAGTCGGCCGGACCGCCGACGACAGCGG
SsBcmE-Y308A-r	GAGCGCACCGCTGTCGTCGGCGGTCCGGCC
SsBcmE-F273A-f	GACCTCCTACGTCACCGCCCTCGACTCCG
SsBcmE-F273A-r	GGTGAGGTCGGAGTCGAGGGCGGTGACGTA
SsBcmE-T307L-f	GGCCAGGAAGTCGGCCGGCTCTACGACGACAGC
SsBcmE-T307L-r	GTAGAGCCGGCCGACTTCCTGGCCGGCGAACTCGGC
SsBcmE-Y308M-f	CAGGAAGTCGGCCGGACCATGGACGACAGCGGT
SsBcmE-Y308M-r	GTCCATGGTCCGGCCGACTTCCTGGCCGGCGAACTC
SsBcmE-Y308F-f	CAGGAAGTCGGCCGGACCTTTGACGACAGCGGT
SsBcmE-Y308F-r	AAAGGTCCGGCCGACTTCCTGGCCGGCGAACT
SsBcmE-V304T-f	GAGTTCGCCGGCCAGGAAACCGGCCGGACCTACG
SsBcmE-V304T-r	GGTTTCCTGGCCGGCGAACTCGGCCACCGACTG

Supplementary Table 3. Primers used in the study of BcmC.

Name	Sequence (5'→3')
SoBcmC-W149A-f	GGAGGTGGTGGTACTCAGgcGTTTGCGGCAAGC
SoBcmC-W149A-r	AAACgcCTGAGTACCACCACCTCCAACTGCACCACC
SoBcmC-T170A-f	TGTGCACCTCATAAAGATgCAGGATTTGTTACC
SoBcmC-T170A-r	TCCTGcATCTTTATGAGGTGCACAACCCAGCTGGTG
SoBcmC-F245A-f	TCTCCTTCGCCGCCGCCGTCAACCCA
SoBcmC-F245A-r	CGGCGGTGGGTTGACGGCGGCGGCGAAG
SoBcmC-N277A-f	GAATTTCTGCGTGATTTTgcTAATGAGACCTGG
SoBcmC-N277A-r	TAgcAAAATCACGCAGAAATTCCTCAACCCCACG
SoBcmC-W281A-f	GATTTTAATAATGAGACCgcGAGCGATCGTCAT
SoBcmC-W281A-r	CTCgcGGTCTCATTATTAAAATCACGCAGAAATTC
SoBcmC-F276A-f	GAGGAATTTCTGCGTGATgcTAATAATGAGACC
SoBcmC-F276A-r	ATTAgcATCACGCAGAAATTCCTCAACCCCACG
SoBcmC-F288A-f	AGCGATCGTCATACCGACgcTGGTATTACAACC
SoBcmC-F288A-r	AgcGTCGGTATGACGATCGCTCCAGGTCTCATT

Supplementary Table 4. Primers used in the study of BcmG.

Name	Sequence (5'→3')
PaBcmG-R81A-f	GAAGATCGTCTGGCACGTgcTGAAGGTTATTTTA
PaBcmG-R81A-r	gcACGTGCCAGACGATCTTCGGTCCACTGACT
PaBcmG-Q94A-f	GATGTTGATCAGGTTGAAgcGTTTTTTCTGGA
PaBcmG-Q94A-r	gcTTCAACCTGATCAACATCACGACTAAAATAAC
PaBcmG-F96A-f	GATCAGGTTGAACAGTTTgcTCTGGAAAGCCG
PaBcmG-F96A-r	gcAAACTGTTCAACCTGATCAACATCACGAC
PaBcmG-H154A-f	GAGCATGCAGGGTACATATgcTCTGACCTTTAA
PaBcmG-H154A-r	gcATATGTACCCTGCATGCTCAGACAACGACC
PaBcmG-T156A-f	GCAGGGTACATATCATCTGgCCTTTAACCAT
PaBcmG-T156A-r	cCAGATGATATGTACCCTGCATGCTCAGACAAC
PaBcmG-S175A-f	CTGAATGTTCATAAAGATgcCGGTTGGGTTAC
PaBcmG-S175A-r	gcATCTTTATGAACATTCAGACCACGTGCACG
PaBcmG-F248A-f	GCTTTAGTTATGCCCTGgcTGTTGATAGCTC
PaBcmG-F248A-r	gcCAGGGCATAACTAAAGCGATCGGCCTGAC
PaBcmG-Y288A-f	GAAATCCTGCATAATACCgcTCAGGAAAATAC
PaBcmG-Y288A-r	gcGGTATTATGCAGGATTTCATTCAGAAACAT
PaBcmG-Y288F-f	AATCCTGCATAATACCTtTCAGGAAAAT
PaBcmG-Y288F-r	CCCTGAGTATTTTCCTGAaAGGTATTAT
PaBcmG-Q94H-f	AGGTTGAACACTTTTTTCTGGAAAG
PaBcmG-Q94H-r	AGAAAAAAGTGTTCAACCTGATCA
PaBcmG-H154F-f	GTACATATTTCCTGACCTTTAACCA
PaBcmG-H154F-r	AAGGTCAGGAAATATGTACCCTGCAT
PaBcmG-L284N-f	AATGAAATCAACCATAATACCTATCAGG
PaBcmG-L284N-r	GTATTATGGTTGATTTCATTCAGAAAC
PaBcmG-Y288W-f	CATAATACCTGGCAGGAAAATACTCAG
PaBcmG-Y288W-r	TTTTCCTGCCAGGTATTATGCAGGATT
PaBcmG-L280A-f	CTGGTTCTGGAAGCAGATTTTGAAATGTTTgcG
PaBcmG-L280A-r	CAGGATTTCATTCgcAAACATTTCAAAATCTGC
paBcmG-L284A-f	ATGTTTCTGAATGAAATCgcaCATAATACCTAT
paBcmG-L284A-f	tgcGATTTCATTCAGAAACATTTCAAAATCTGC

Supplementary Figures

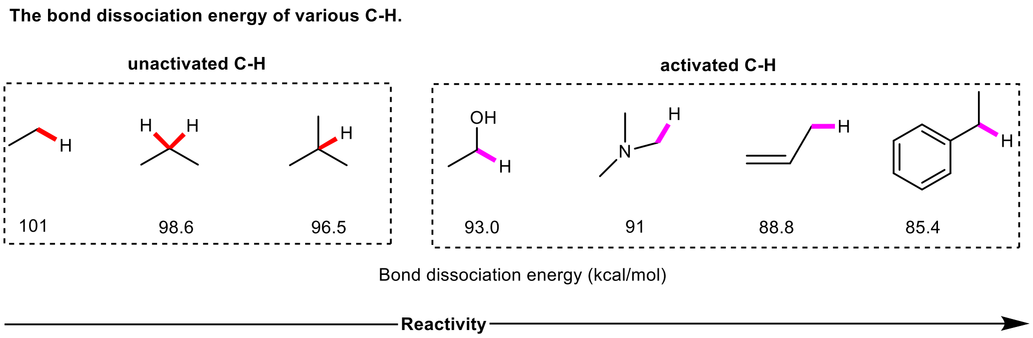

Supplementary Fig. 1. The bond dissociation energy of different types of C-H.

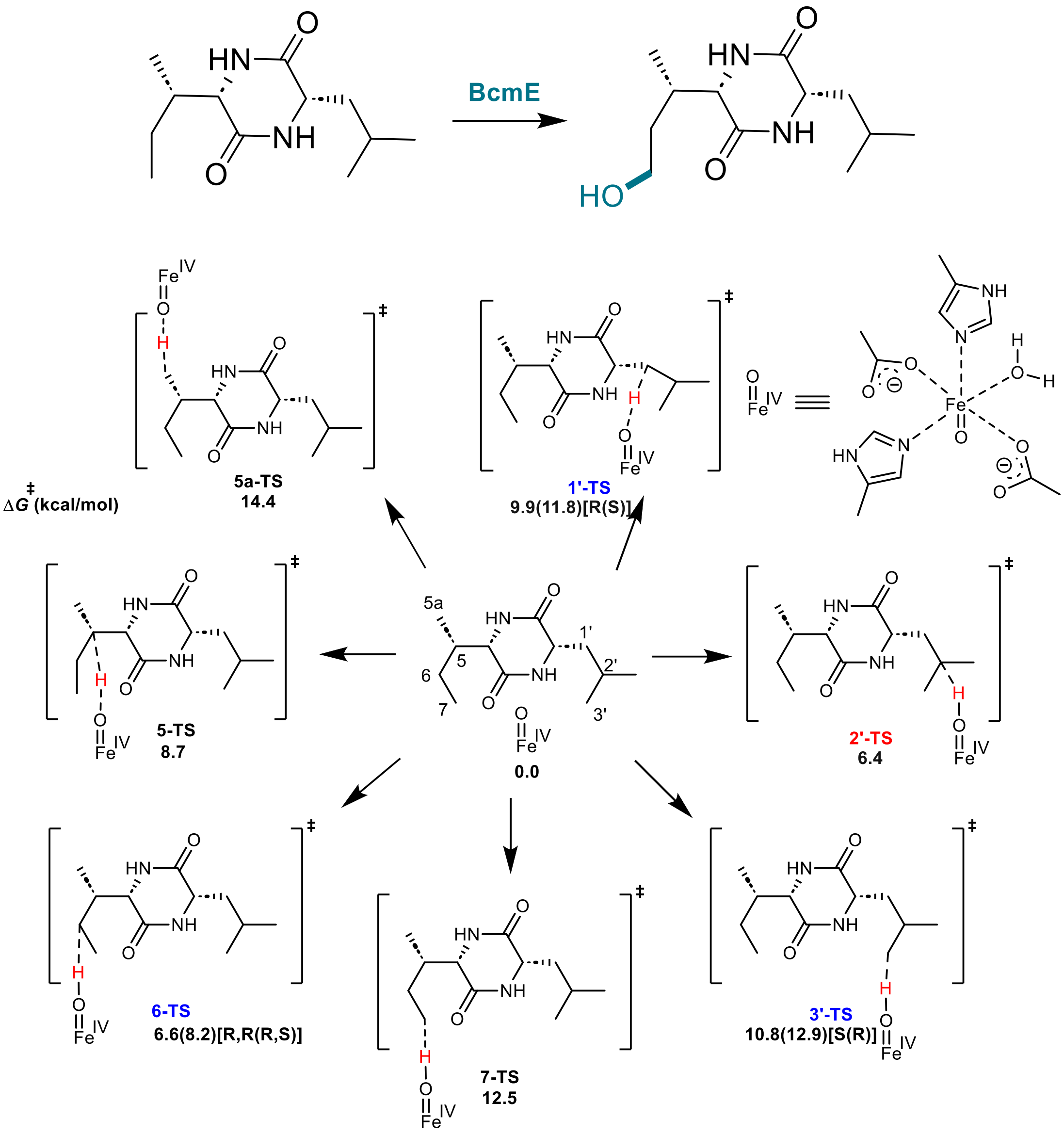

Supplementary Fig. 2. The intrinsic site selectivity of 1 hydroxylation. DFT-computed Gibbs free energies for seven different types of hydroxylation of 1 in BcmE by a catalyst model. Computed at the CPCM(chlorobenzene)-B3LYP-D3/6-311+G(2d,p)+SDD(Fe)//CPCM(chlorobenzene)-B3LYP-D3/6-31G(d,p)+LanL2DZ(Fe) level of theory.

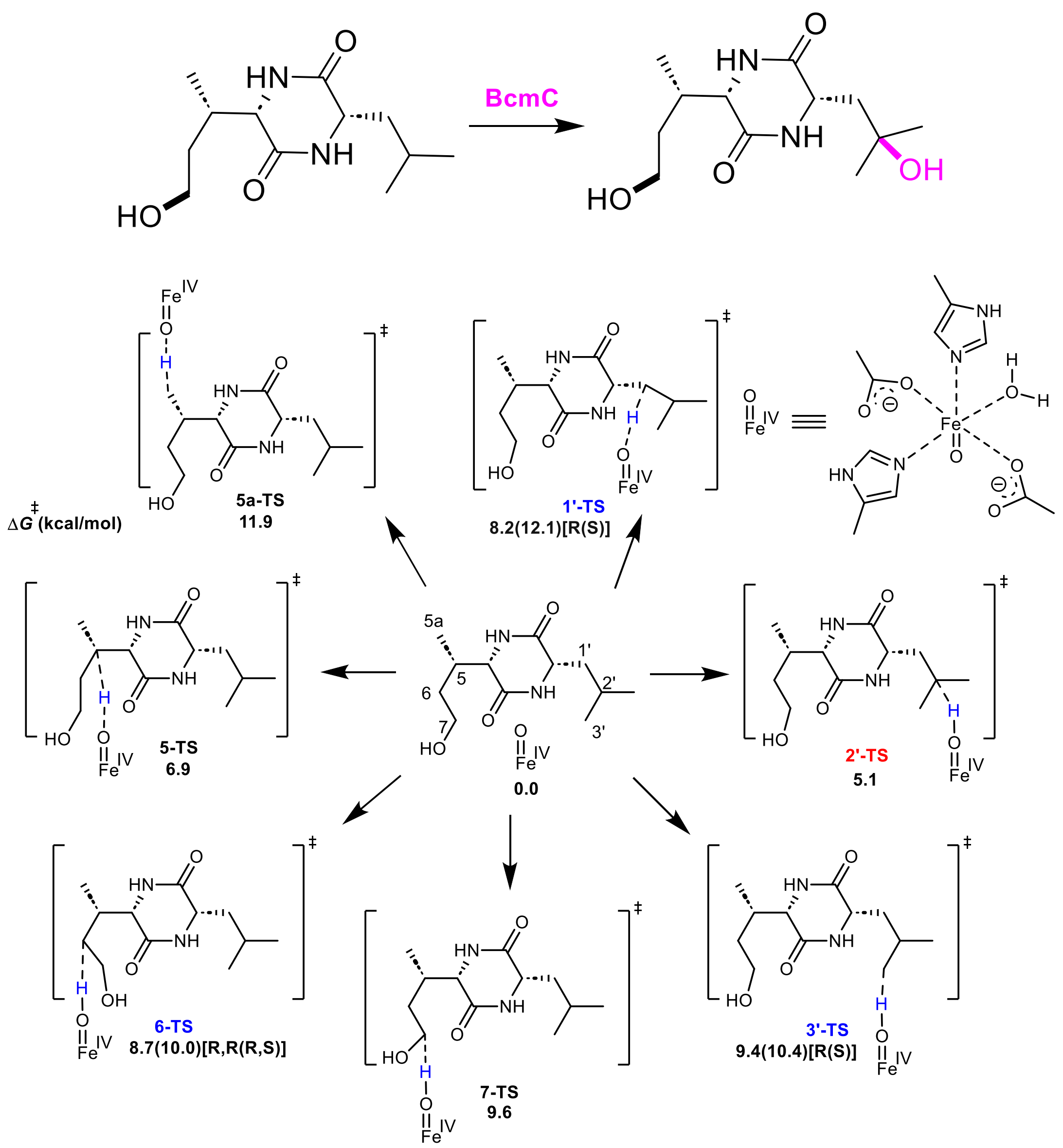

Supplementary Fig. 3. The intrinsic site selectivity of 2 hydroxylation. DFT-computed Gibbs free energies for seven different types of hydroxylation of **2** in BcmC by a catalyst model. Computed at the CPCM(chlorobenzene)-B3LYP-D3/6-311+G(2d,p)+SDD(Fe)//CPCM(chlorobenzene)-B3LYP-D3/6-31G(d,p)+LanL2DZ(Fe) level of theory.

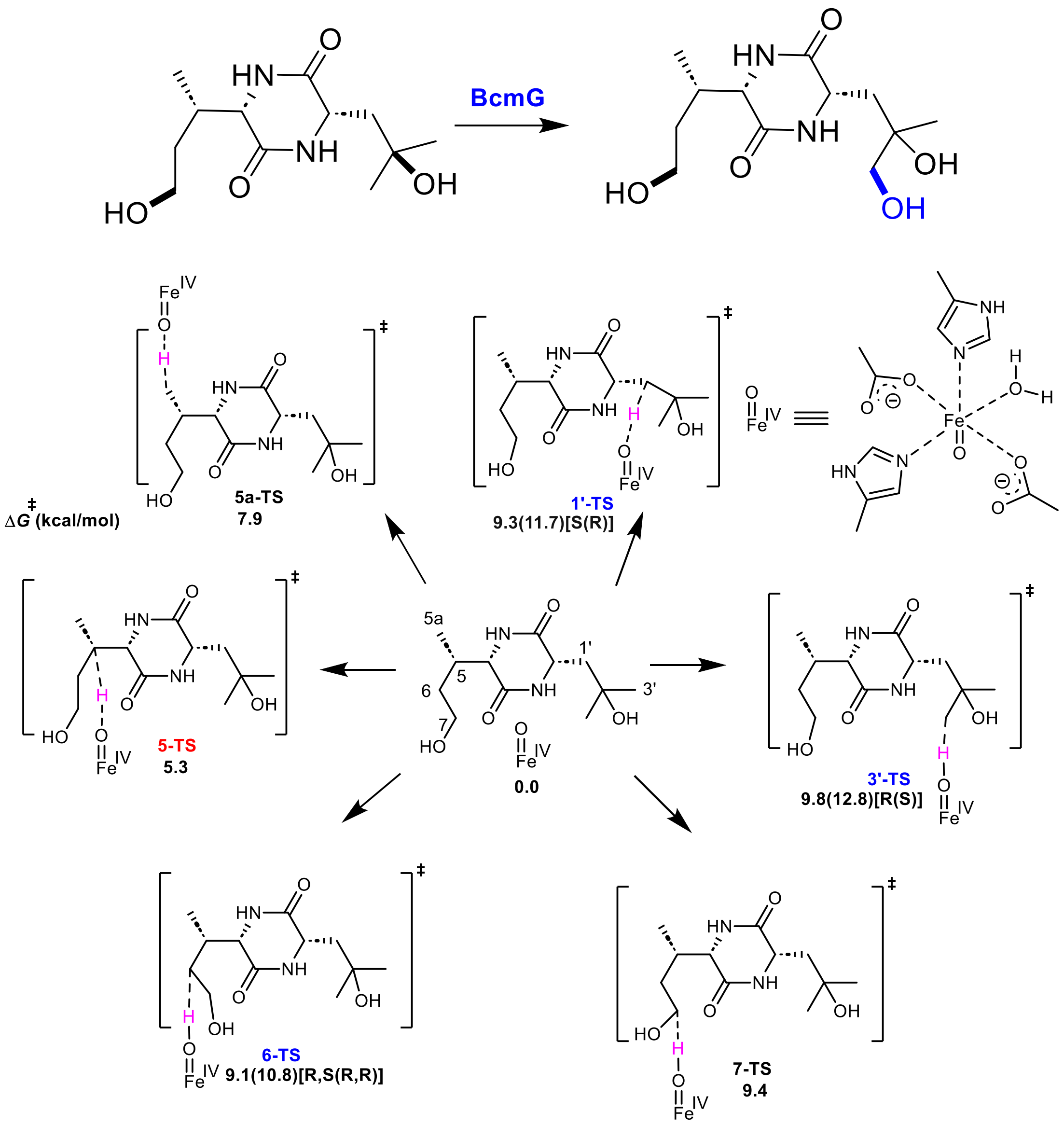

Supplementary Fig. 4. The intrinsic site selectivity of 3 hydroxylation. DFT-computed Gibbs free energies for seven different types of hydroxylation of **3** in BcmG by a catalyst model. Computed at the CPCM(chlorobenzene)-B3LYP-D3/6-311+G(2d,p)+SDD(Fe)//CPCM(chlorobenzene)-B3LYP-D3/6-31G(d,p)+LanL2DZ(Fe) level of theory.

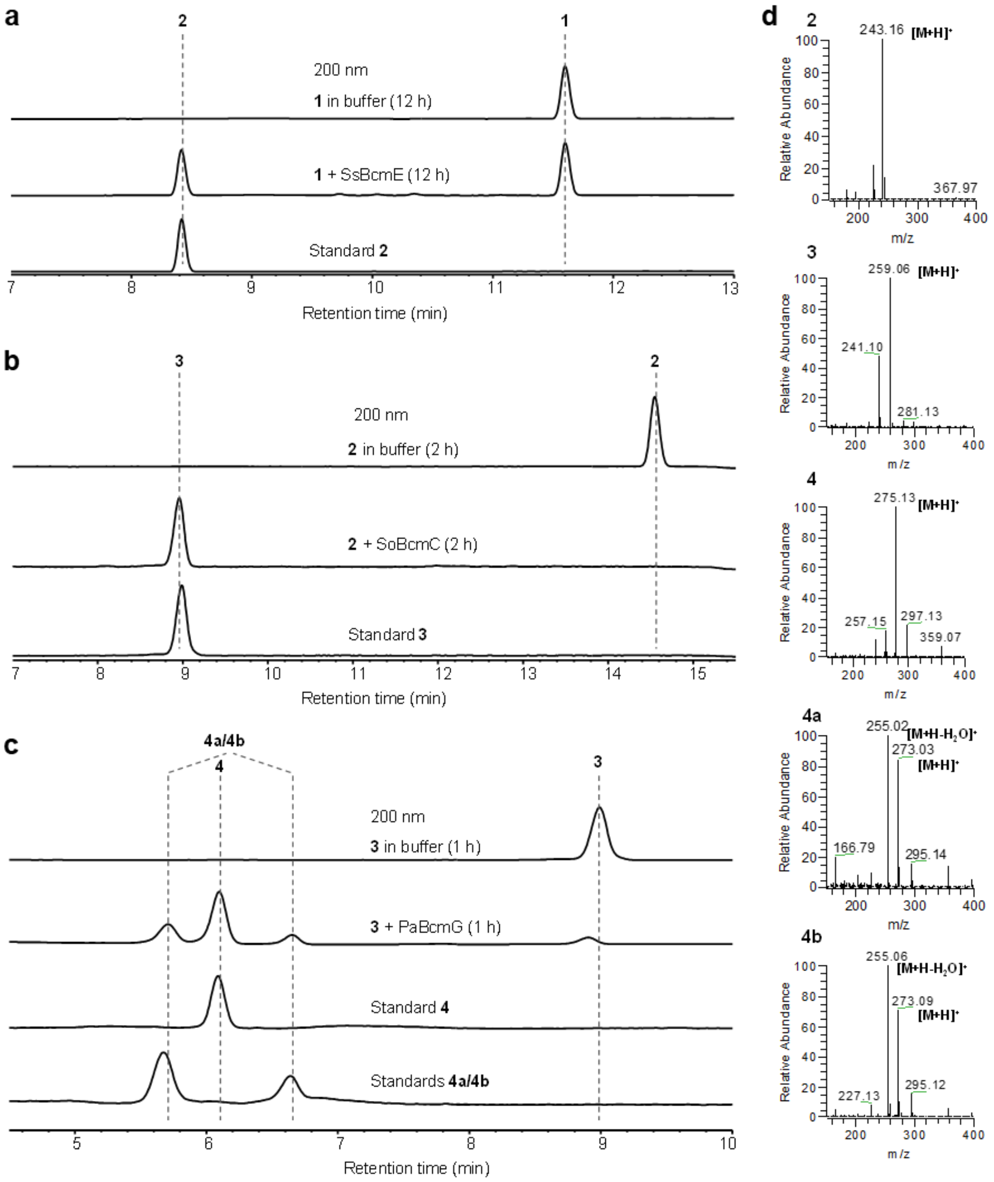

Supplementary Fig. 5. HPLC and LC-MS analyses of in vitro enzyme-catalyzed hydroxylation reactions. a, Representative HPLC chromatograms of the SsBcmE-catalyzed reaction mixtures using compound **1** as the substrate. **b**, Representative HPLC chromatograms of the SoBcmC-catalyzed reaction mixtures using compound **2** as the substrate. **c**, Representative HPLC chromatograms of the PaBcmG-catalyzed reaction mixtures using compound **3** as the substrate. d, (+)-ESI-MS spectra of **2**–**4**, **4a**, and **4b**. The structures of compounds **4a** and **4b** are shown in **Supplementary Fig. 14**.

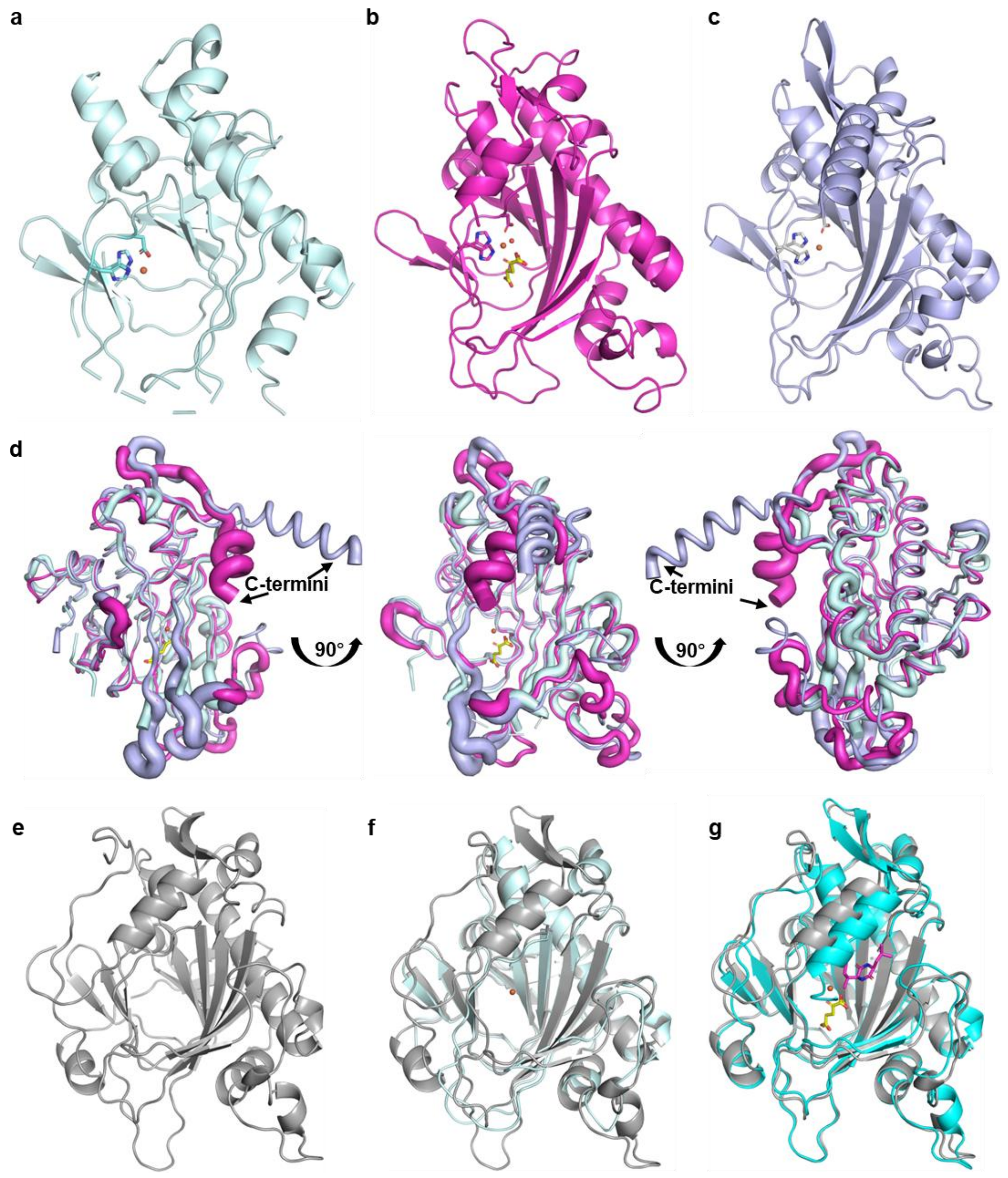

Supplementary Fig. 6. The structure of BcmE, BcmC and BcmG without substrates. The "HXDX$_n$H" was shown as sticks, which coordinated with Fe^{II} (colored in orange). **a**, The structure of SsBcmE was shown as cartoon (colored in pale cyan). The dash line was the missing loops of SsBcmE. **b**, The structure of SsBcmC was shown as cartoon (colored in magenta). Co-substrate αKG was shown as yellow sticks. **c**, The structure of PaBcmG was shown as cartoon (colored in light blue). **d**, The alignment of BcmE, BcmC and BcmG. The thickness of putty is directly related to the b-factor, the thicker the

higher. **e**, The whole length apo SsBcmE model built by AlphaFold2 (AF2 model, in grey). **f**, The alignment of crystal structural SsBcmE (colored in cyan, αKG shown as yellow sticks, substrate shown as magenta sticks) and its AF2 model, r.m.s.d. 0.57 Å. **g**, The alignment of complex structure and AF2 model, r.s.m.d. 0.82 Å.

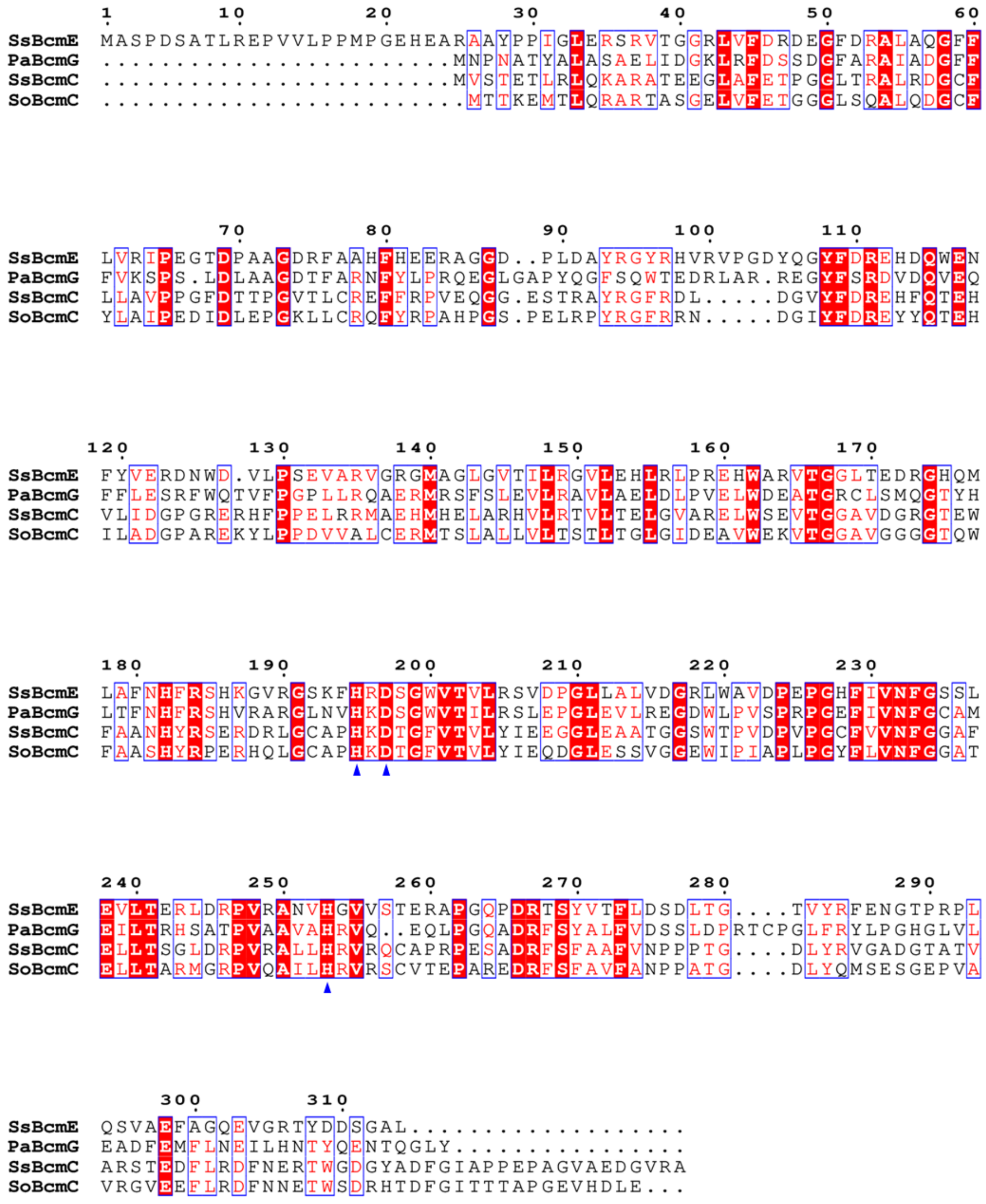

Supplementary Fig. 7. The sequence alignment of SsBcmE, SoBcmC, SsBcmC and PaBcmG. The conserved "HXD-Xn-H" motif was labeled by blue triangle. The three enzymes share 35-42% sequence identity, the sequence similarity between SsBcmC and SoBcmC is at approximately 56%.

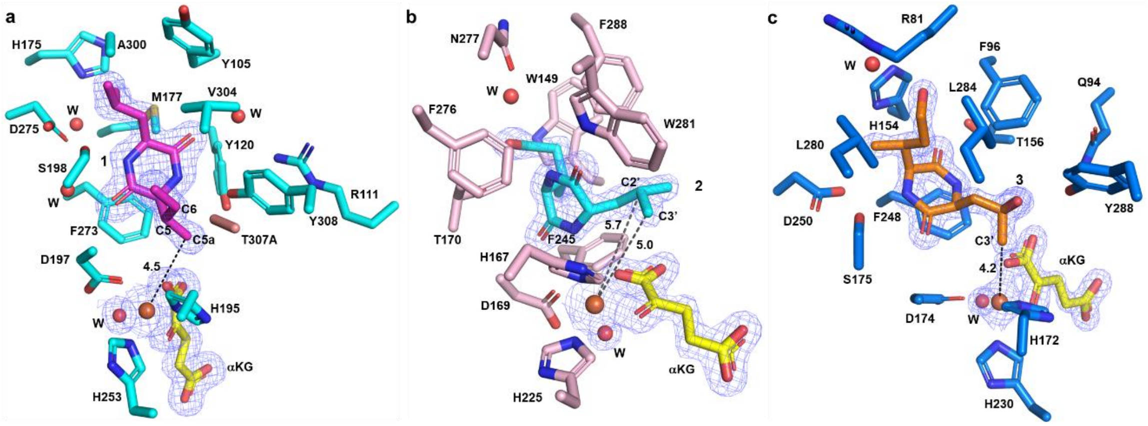

Supplementary Fig. 8. The omit map of substrate, αKG, Fe(II) and coordinating water in complex structure of SsBcmE^{T307A}, SoBcmC and PaBcmG. a, **b**, **c**, The x-ray structure of complex structures of SsBcmE^{T307A}•Fe^{II}•αKG•**1** (cyan), SoBcmC•Fe^{II}•αKG•**2** (pink) and PaBcmG•Fe^{II}•αKG•**3** (blue), respectively. The omit map (blue mesh) for substrates, αKG, Fe(II) and coordinated water in active site are contoured to 3.0 σ. The oxygen atoms were coloured in red, nitrogen atoms were coloured in blue and the carbon atoms of αKG, **1**, **2** and **3** were coloured in yellow, magenta, cyan and orange. The distance of the iron center to the closest carbon atoms or special carbon atoms is labelled with black dash line.

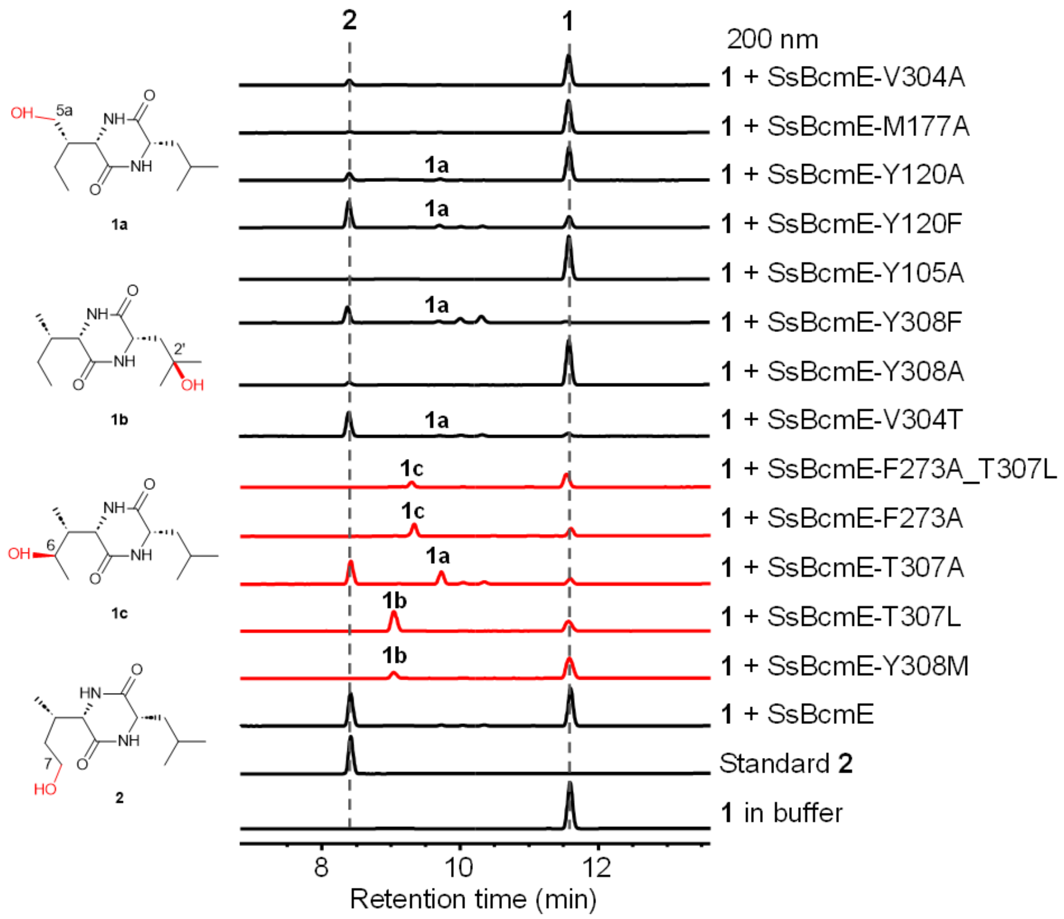

Supplementary Fig. 9. HPLC analysis of in vitro reaction of SsBcmE and its mutants. The enzymatic reaction (50 μL) containing 50 mM Tris-HCl buffer (pH 7.5), 0.6 mM **1**, 2 mM αKG, 2 mM l-ascorbic acid, 0.1 mM $FeSO_4 \cdot 7H_2O$, and 50 μg purified enzyme was incubated at 16 °C for 12 h. The reactions were quenched by the addition of 100 μL of pre-cooled methanol and centrifuged at 13,800 g for 30 min. The supernatants were analyzed by HPLC. The data show one representative experiment from three independent replicates with similar results. Compounds **1b** was also obtained by enzymatic scaled-up reaction of the SsBcmE-T307L mutant and its structure was identified by NMR. The NMR data of **1b** were consistent with the reported values[63,64].

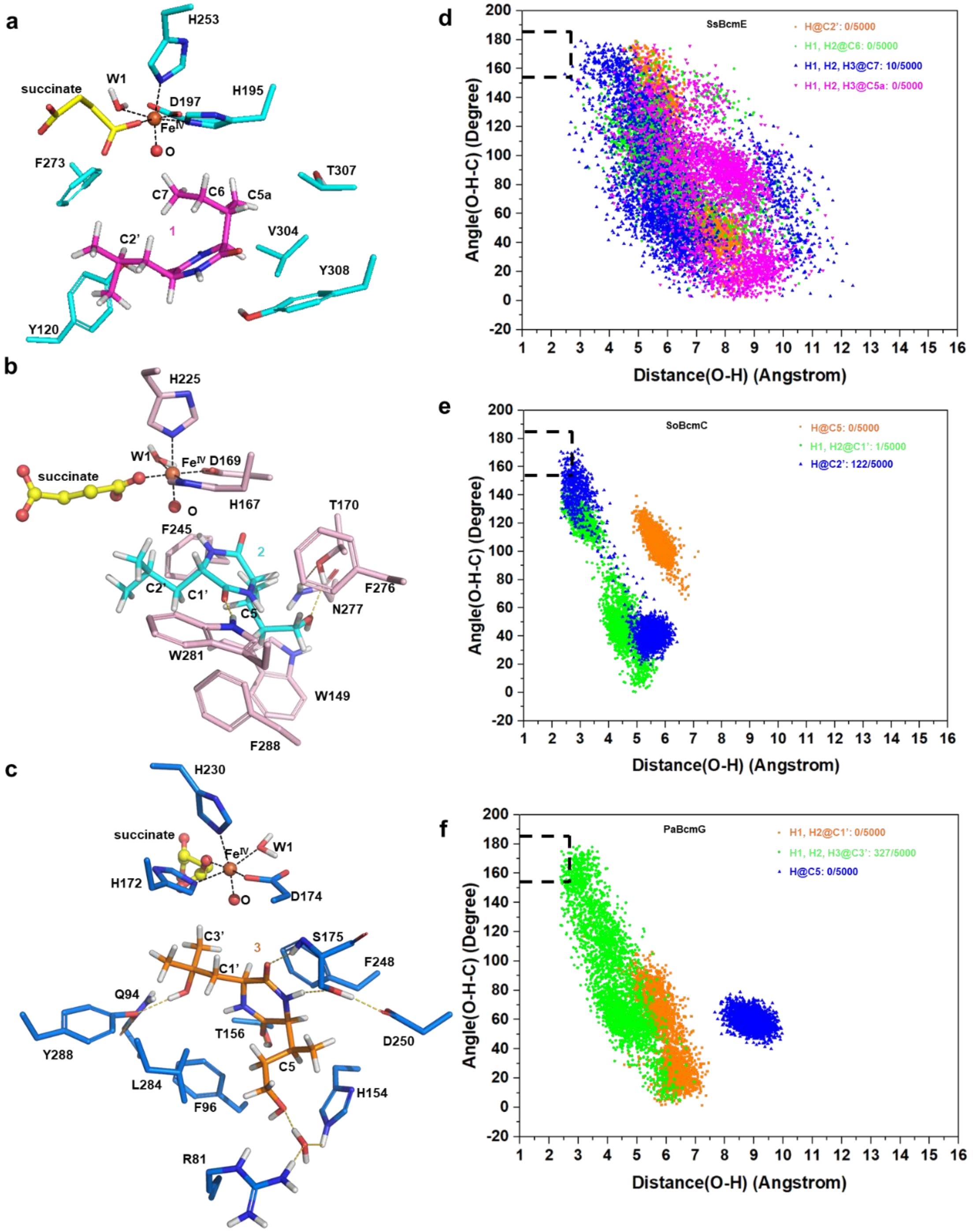

Supplementary Fig. 10. The stereo- and regioselectivity of C－H activation by SsBcmE, SoBcmC and PaBcmG. a, **b** and **c**, Representative MD snapshots of the reactant Fe^{IV}-oxo species of WT SsBcmE, SoBcmC and PaBcmG in complex with **1**, **2** and **3**, respectively. **d**, **e** and **f**, MD plots for the distances of the forming O−H bond and angles between the forming O−H bond and the breaking H−C bond in WT SsBcmE,

SoBcmC and PaBcmG complexes with **1**, **2** and **3**, respectively. According to the DFT-optimized structures of transition sates, we defined the active conformation as O−H distance is ≤ 2.8 Å (the sum of van der Waals atomic radii of oxygen and hydrogen atoms) and angle O−H−C is in the range of 170 ± 15 ° Source data are provided as a Source Data file.

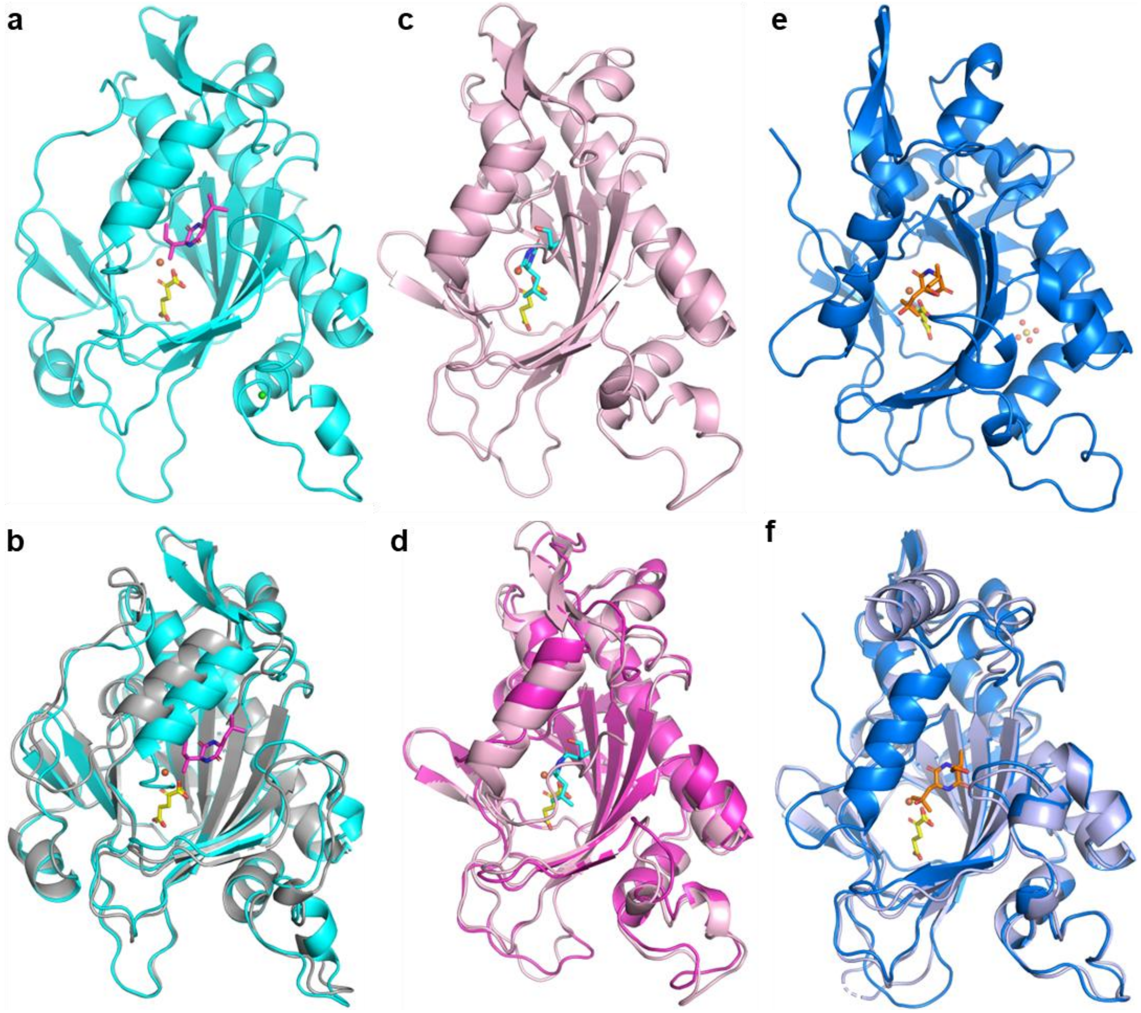

Supplementary Fig. 11. The alignment of apo and complex structures of SsBcmE, SoBcmC and PaBcmG. a, **c**, **e,** The complex structures of BcmE (cyan, substrate shown as magenta sticks), BcmC (pink, substrate shown as cyan sticks) and BcmG (blue, substrate shown as orange sticks) were shown as cartoon, respectively. αKG (yellow) and iron atoms (orange) were shown as sticks and spheres, repectively. **b**, **d**, **f,** The alignment of apo BcmE (AF2 model, grey)/ternary complex of BcmC (magenta)/ ternary complex of BcmG (light blue) and their corresponding complex structures, and the r.m.s.d. value was 0.82 Å, 0.75 Å and 0.64 Å, respectively.

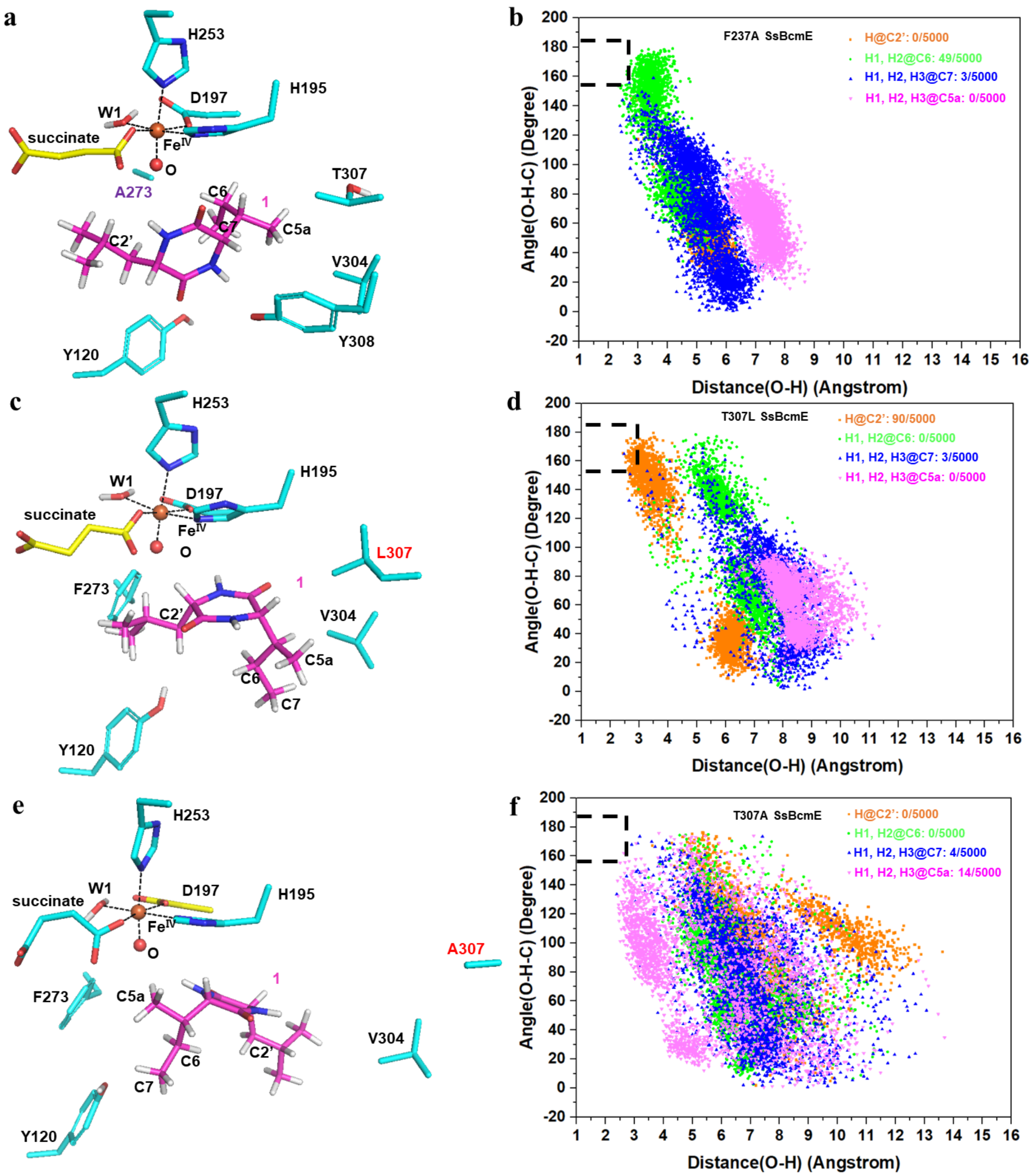

Supplementary Fig. 12. Origin of regio-selectivity in SsBcmE-catalysed hydroxylation reactions. **a**, **c** and **e**, Representative MD snapshots of the reactant Fe^{IV}-oxo species of F273A, T307L and T307A SsBcmE in complex with **1**, respectively. **b**, **d** and **f**, MD plots for the distances of the forming O−H bond and angles between the forming O−H bond and the breaking H−C bond in F273, T307L and T307A SsBcmE complexes with 1, respectively. According to the DFT-optimized structures of transition sates, we defined the active conformation as O−H distance is ≤2.8 Å (the sum of van der

Waals atomic radii of oxygen and hydrogen atoms) and angle O−H−C is in the range of 170 ± 15° Source data are provided as a Source Data file.

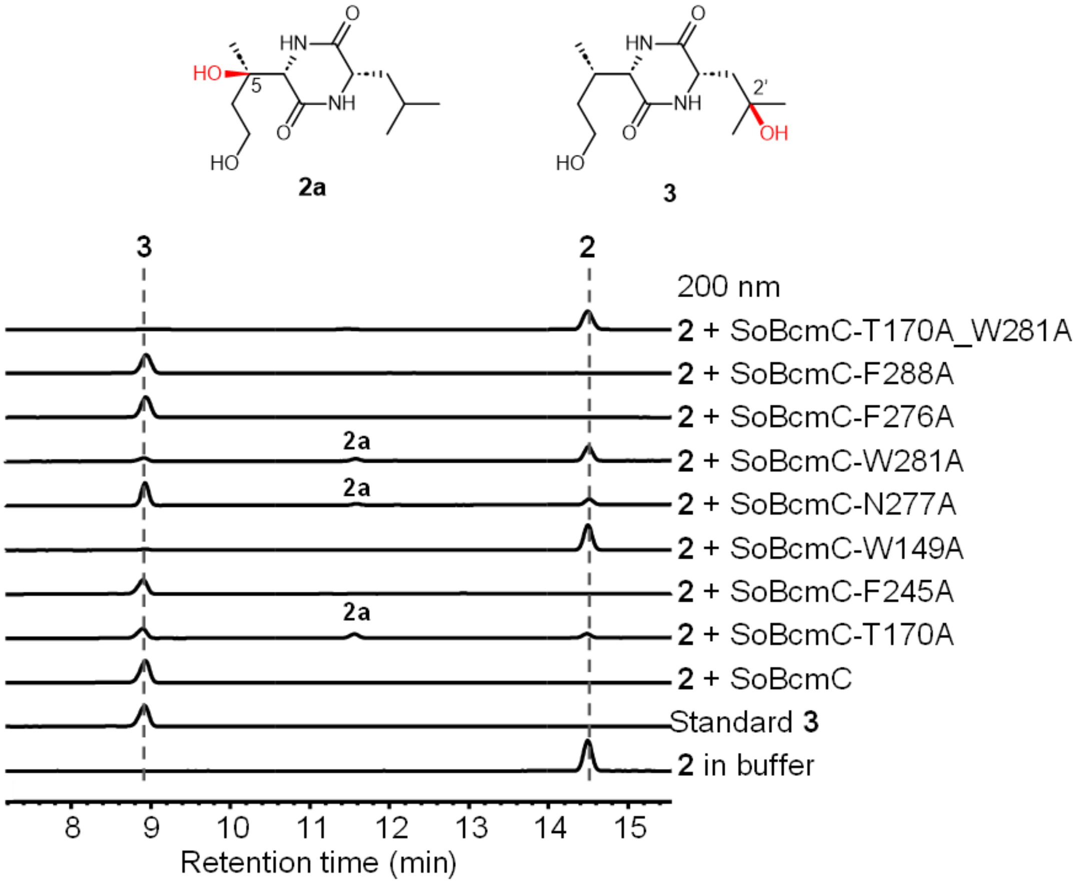

Supplementary Fig. 13. HPLC analysis of in vitro reaction of SoBcmC and its mutants. The enzymatic reaction (50 µL) containing 50 mM Tris-HCl buffer (pH 7.5), 0.6 mM **2**, 2 mM αKG, 2 mM l-ascorbic acid, 0.1 mM $FeSO_4 \cdot 7H_2O$, and 50 µg purified enzyme was incubated at 37 °C for 2 h. The reactions were quenched by the addition of 100 µL of pre-cooled methanol and centrifuged at 13,800 g for 30 min. The supernatants were analyzed by HPLC. The data show one representative experiment from three independent replicates with similar results.

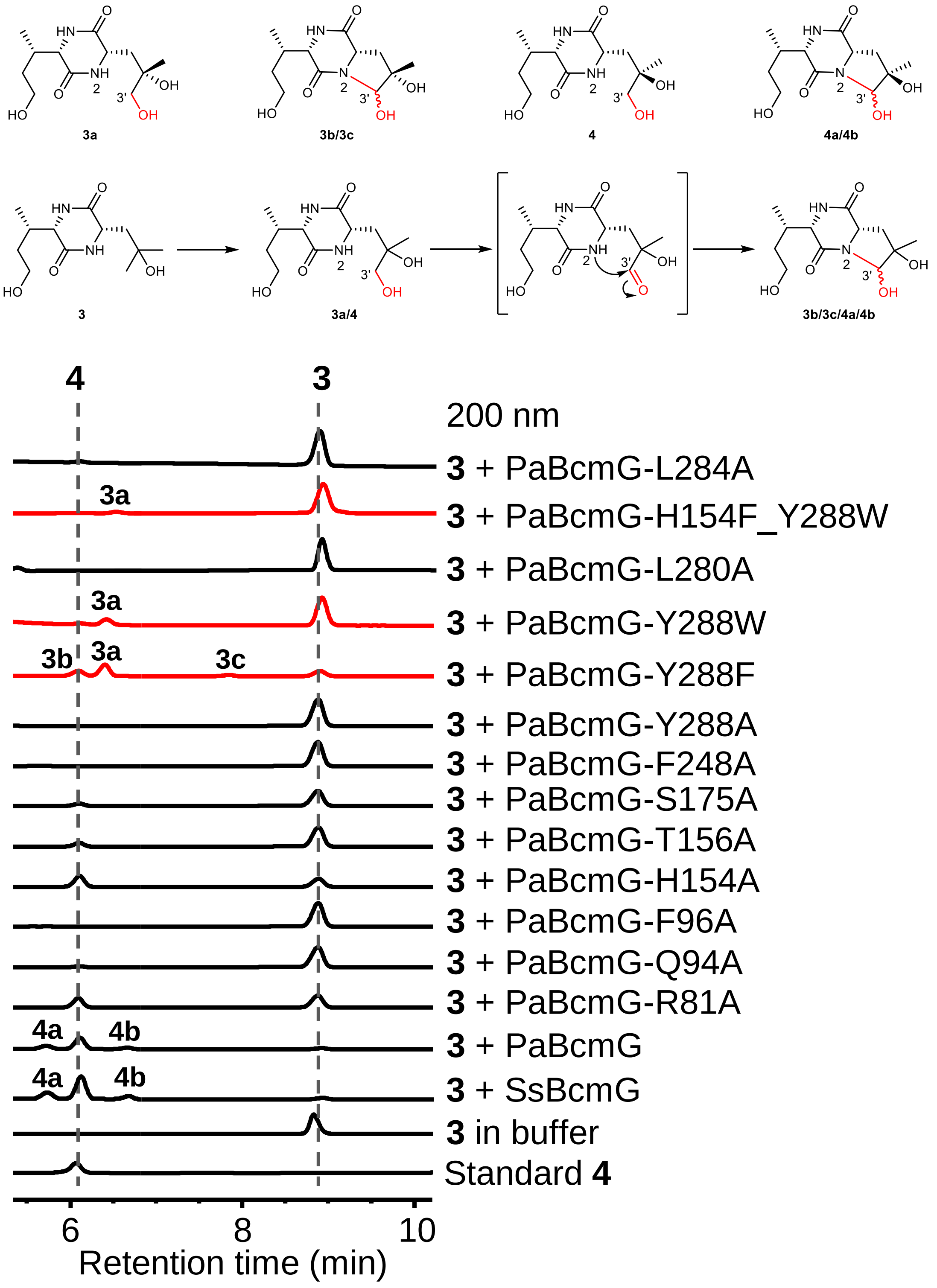

Supplementary Fig. 14. HPLC analysis of in vitro reaction of PaBcmG and its mutants. The enzymatic reaction (50 µL) containing 50 mM Tris-HCl buffer (pH 7.5), 0.6 mM **3**, 2 mM αKG, 2 mM l-ascorbic acid, 0.1 mM $FeSO_4 \cdot 7H_2O$, and 50 µg purified

enzyme was incubated at 37 °C for 1 h. The reactions were quenched by the addition of 100 μL of pre-cooled methanol and centrifuged at 13,800 g for 30 min. The supernatants were analyzed by HPLC. The data show one representative experiment from three independent replicates with similar results. Similar to **4a** and **4b** produced by WT PaBcmG and SsBcmG, the diastereomers **3b** and **3c** are also two overoxidation products. According to our previous research as well as other group studies on BcmG-catalyzed reaction[5,6,7], we proposed that PaBcmG-Y288F can catalyze the further oxidation of product **3a** at C3' to produce an aldehyde-containing intermediate, which undergoes addition by amide N2 to form **3b** and **3c**.

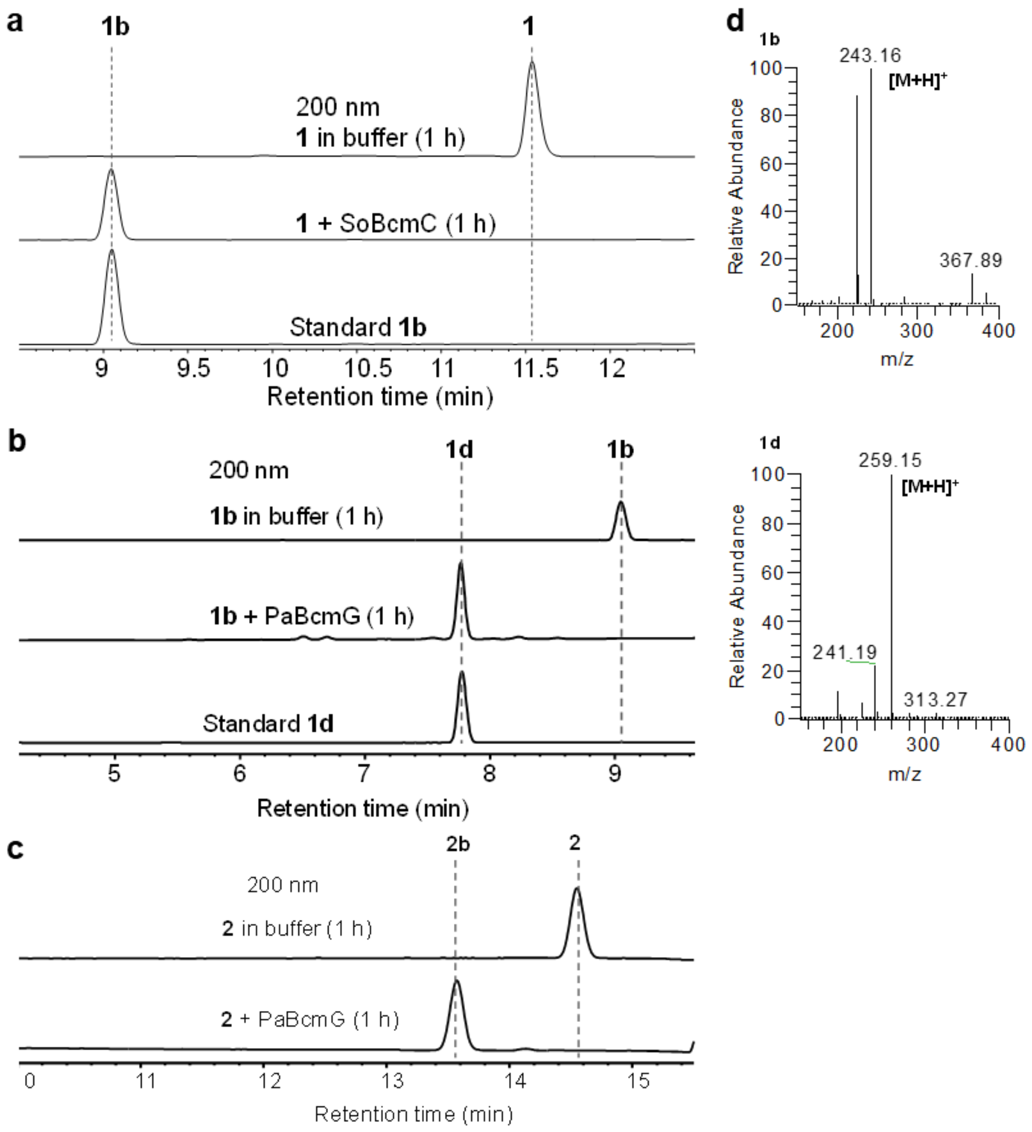

Supplementary Fig. 15. HPLC and LC-MS analyses of in vitro enzyme-catalyzed hydroxylation reactions. a, Representative HPLC chromatograms of the SoBcmC-catalyzed reaction mixtures using compound **1** as the substrate. **b**, Representative HPLC chromatograms of the PaBcmG-catalyzed reaction mixtures using compound **1b** as the substrate. **c**, Representative HPLC chromatograms of the PaBcmG-catalyzed reaction mixtures using compound **2** as the substrate. **d**, (+)-ESI-MS spectra of **1b**, and **1d**.

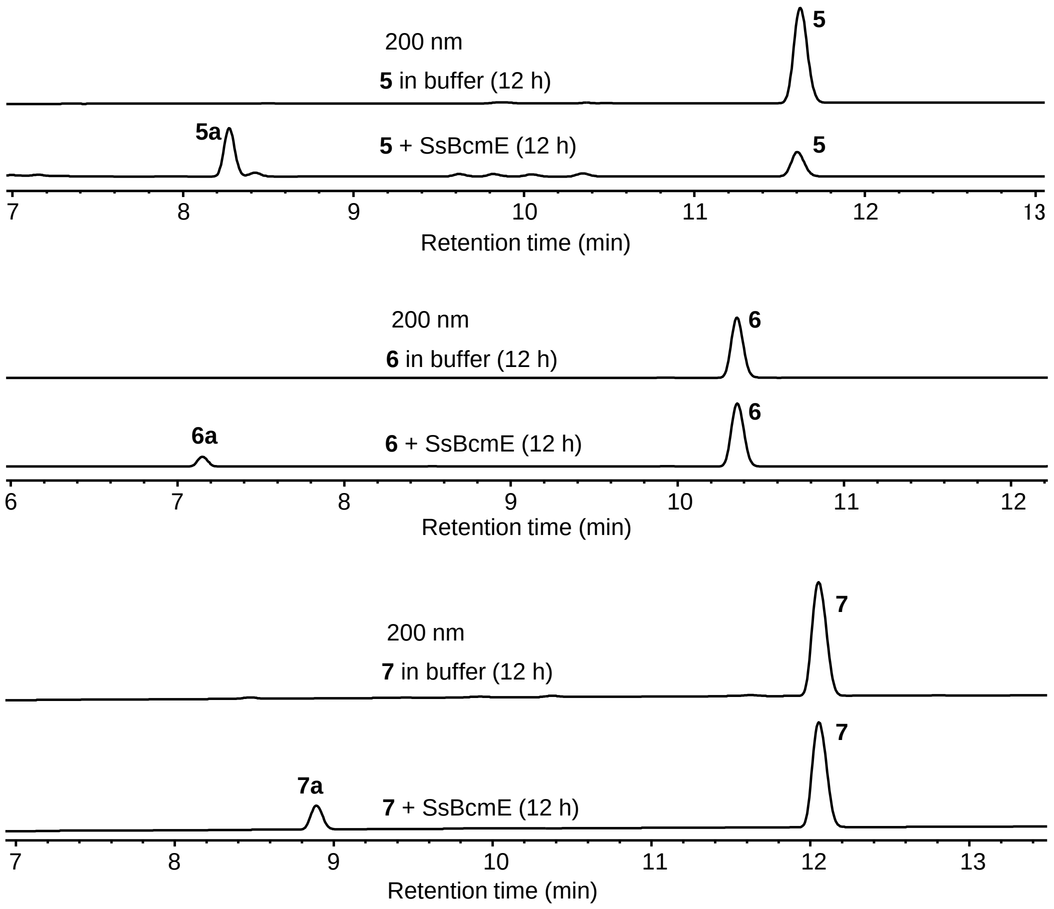

Supplementary Fig. 16. HPLC analysis of in vitro SsBcmE-catalyzed hydroxylation reactions using compounds 5–7 as substrates. The data show one representative experiment from three independent replicates with similar results.

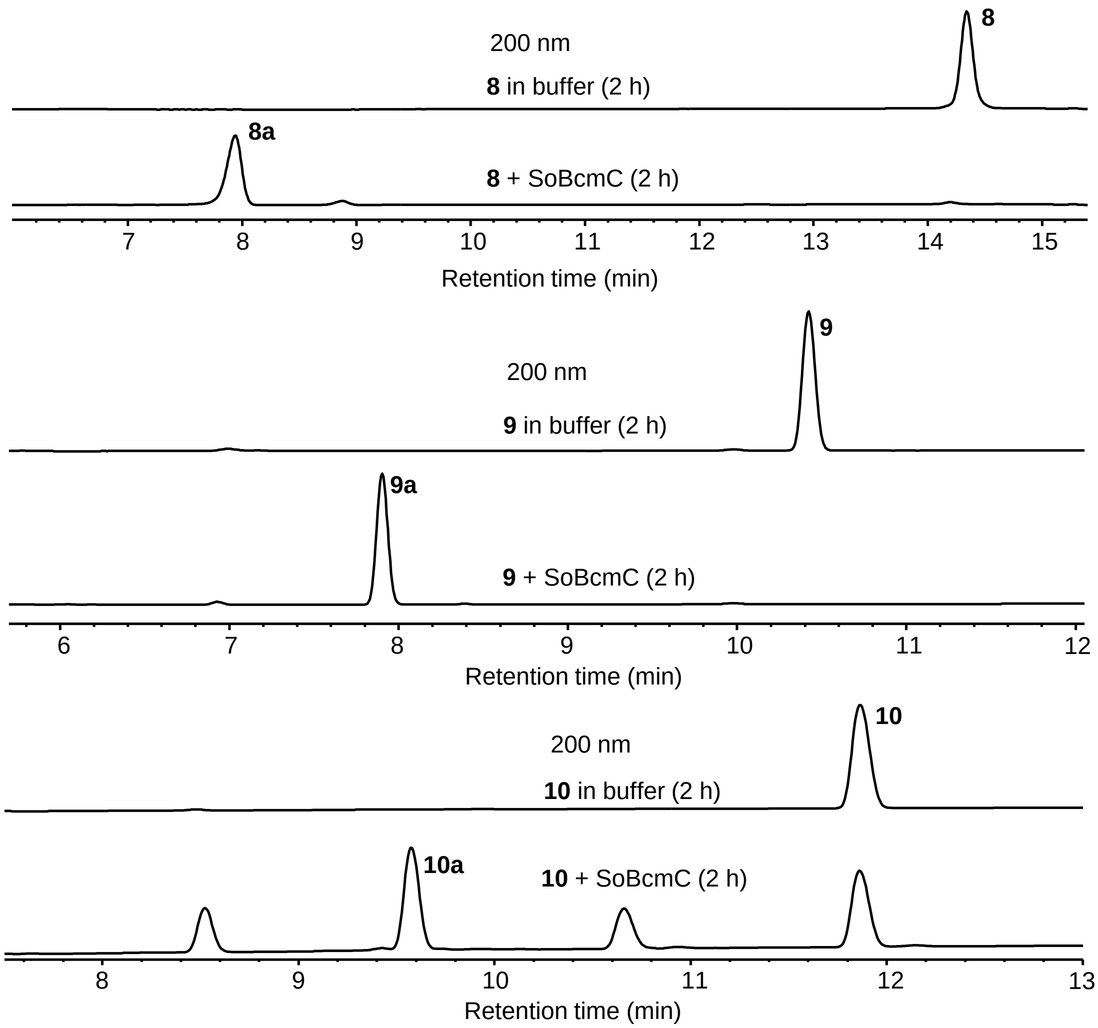

Supplementary Fig. 17. HPLC analysis of in vitro SoBcmC-catalyzed hydroxylation reactions using compounds 8–10 as substrates. The data show one representative experiment from three independent replicates with similar results.

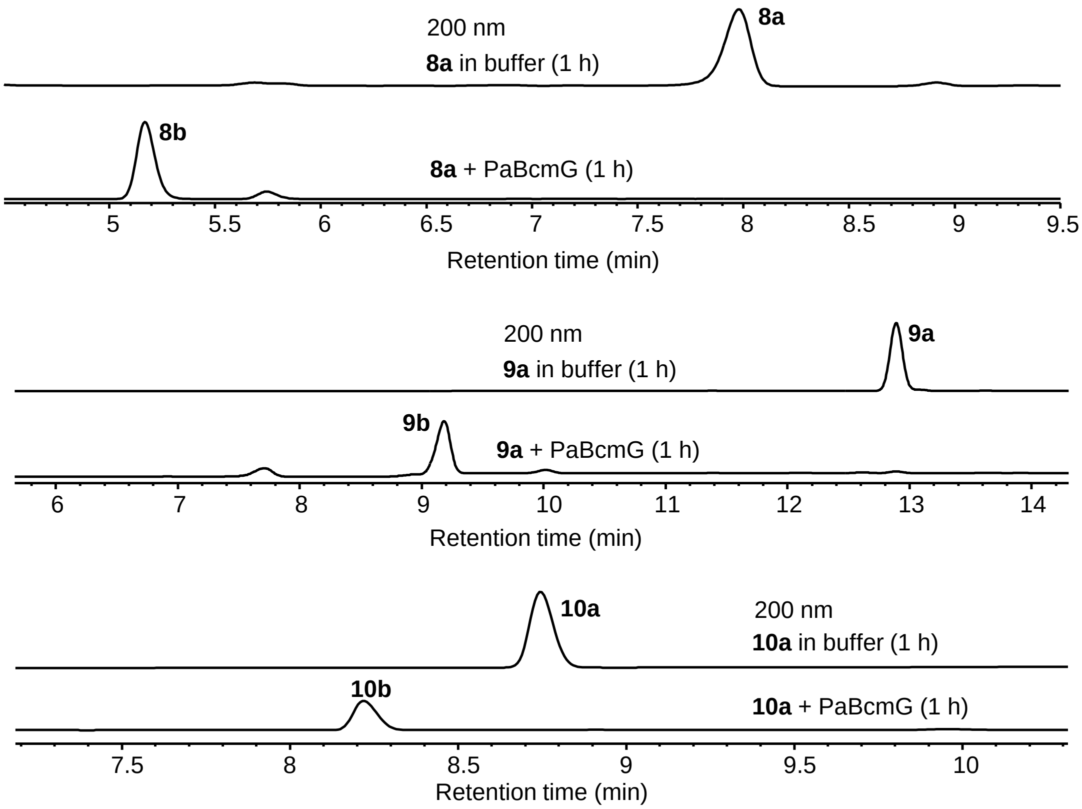

Supplementary Fig. 18. HPLC analysis of in vitro PaBcmG-catalyzed hydroxylation reactions using compounds 8a–10a as substrates. The data show one representative experiment from three independent replicates with similar results.

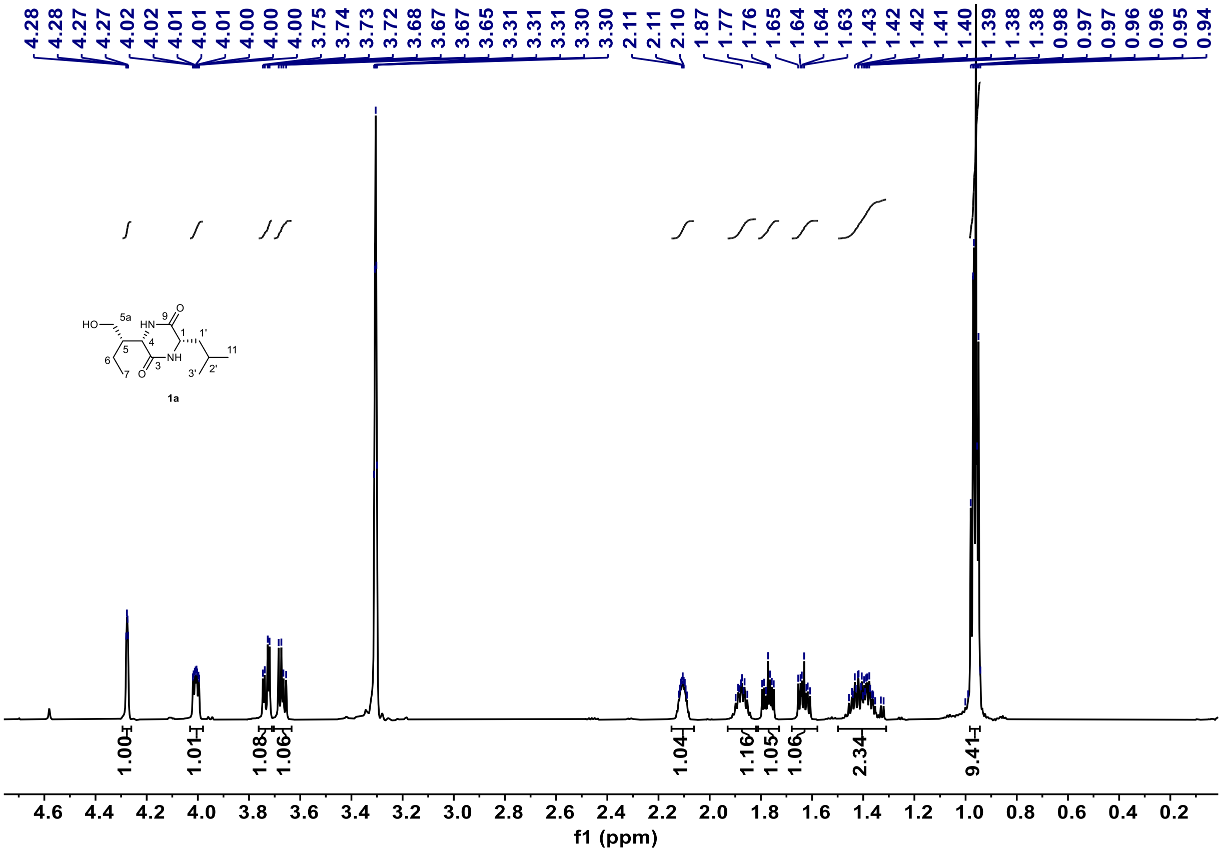

Supplementary Fig. 19. ^{1}H NMR spectrum of **1a** (600 MHz, CD_3OD).

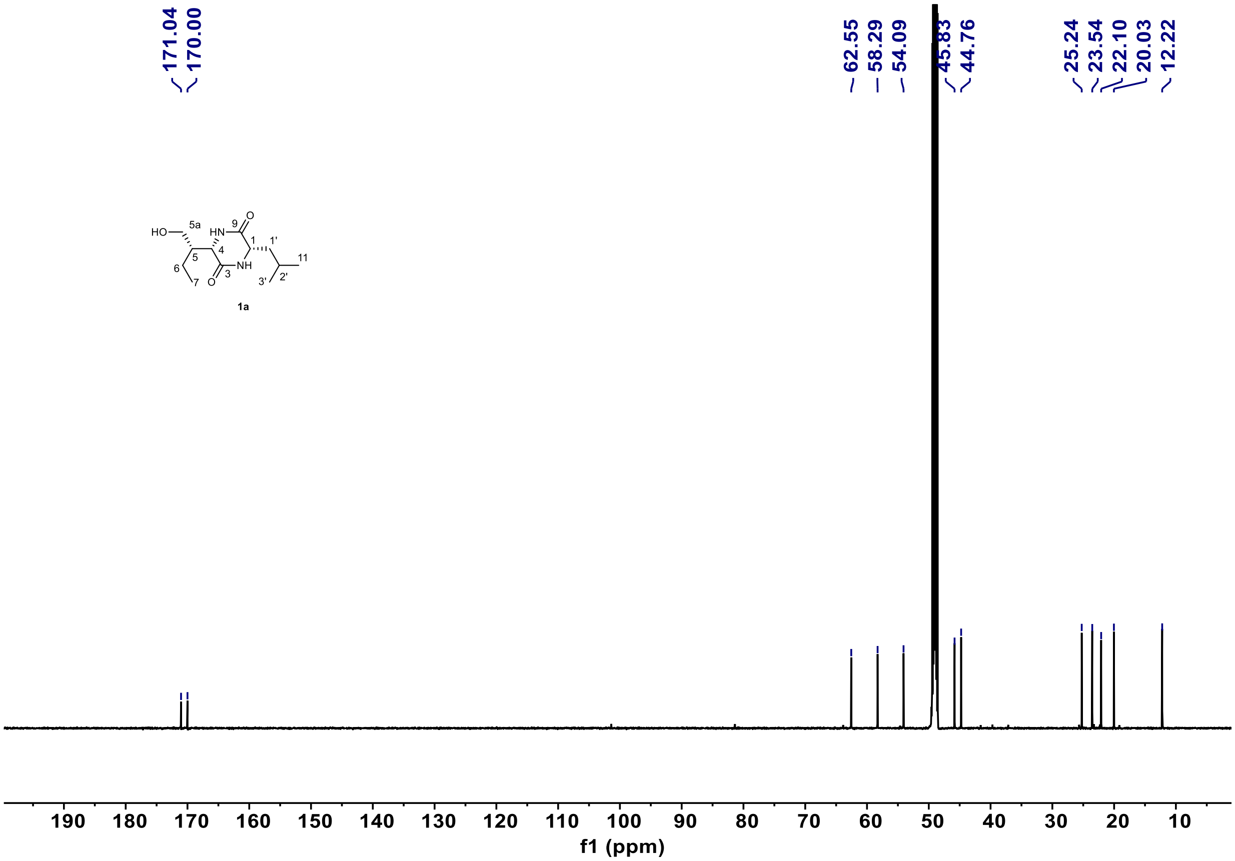

Supplementary Fig. 20. ^{13}C NMR spectrum of **1a** (150 MHz, CD_3OD).

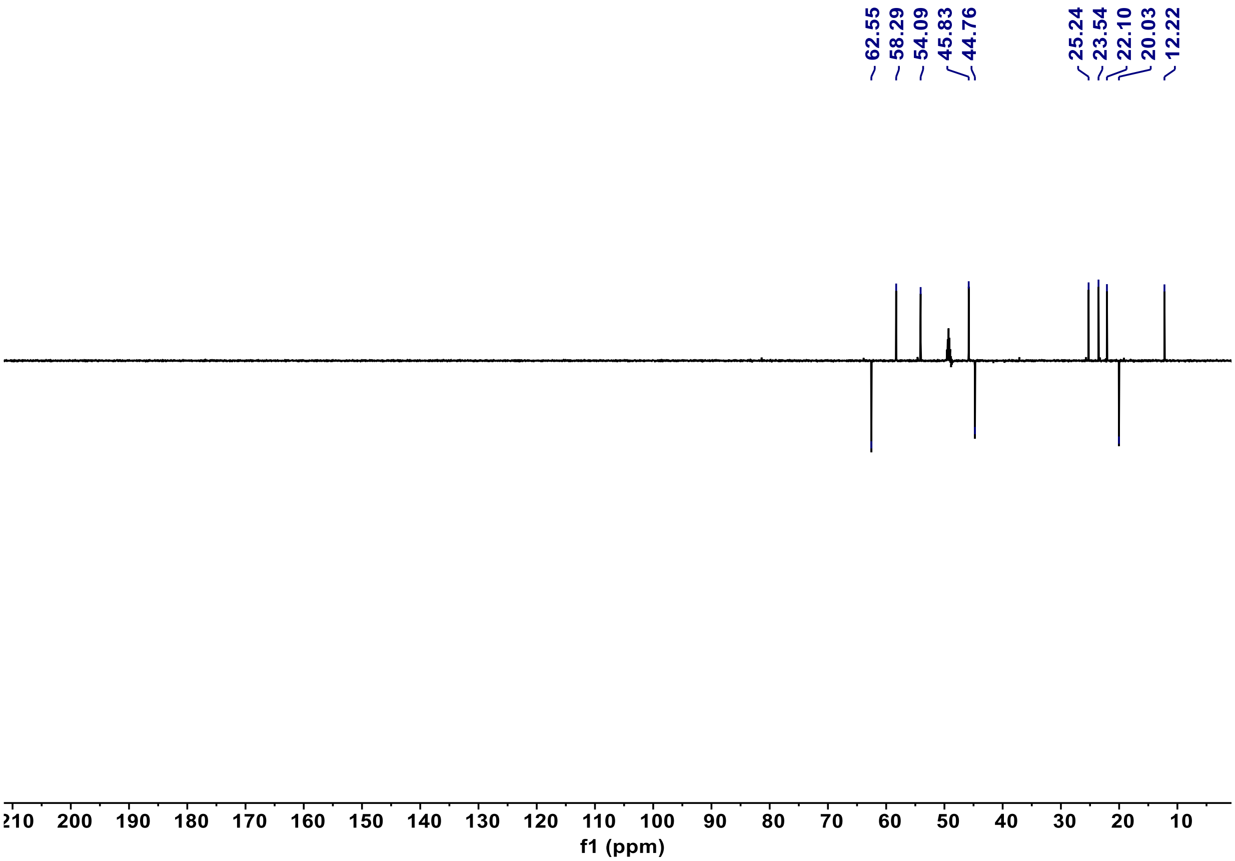

Supplementary Fig. 21. DEPT135 spectrum of **1a** (150 MHz, CD_3OD).

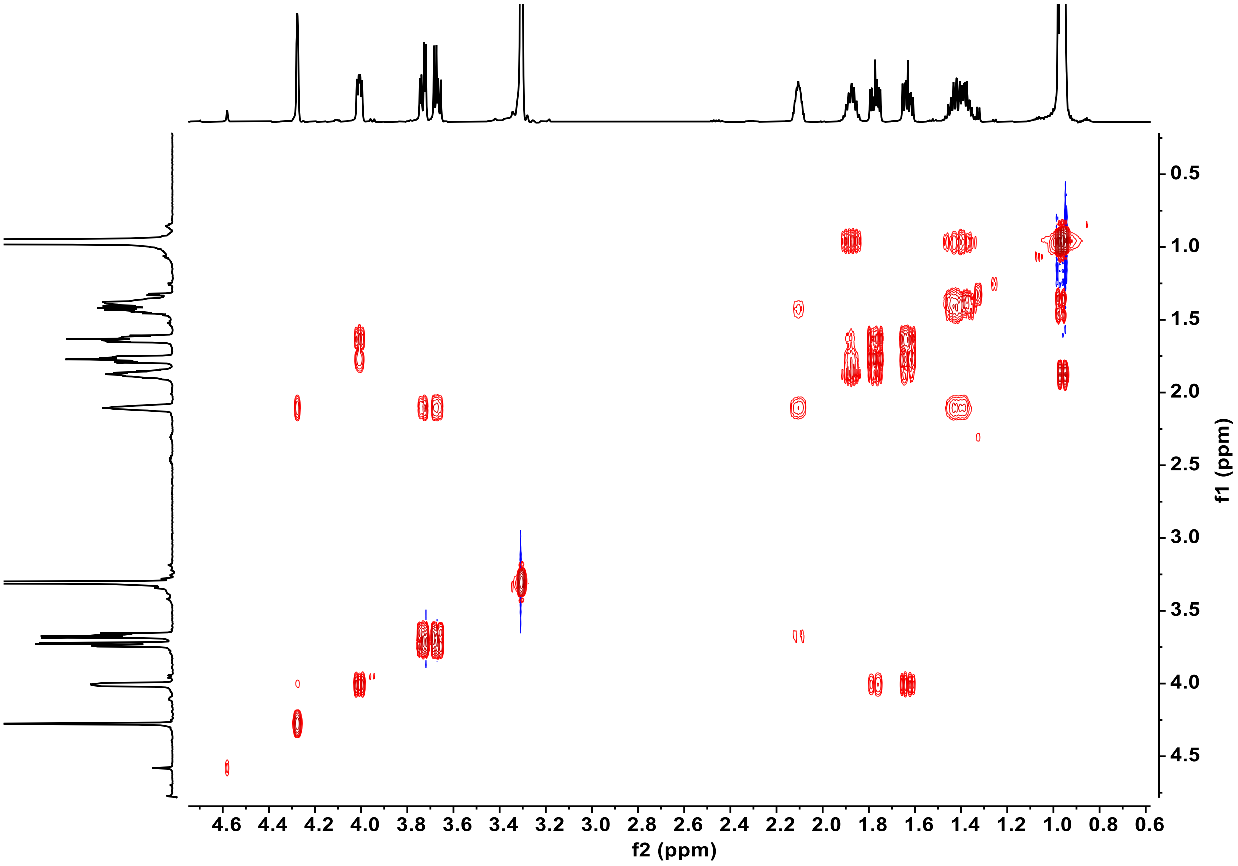

Supplementary Fig. 22. ^{1}H-^{1}H COSY spectrum of **1a** (600 MHz, CD_3OD).

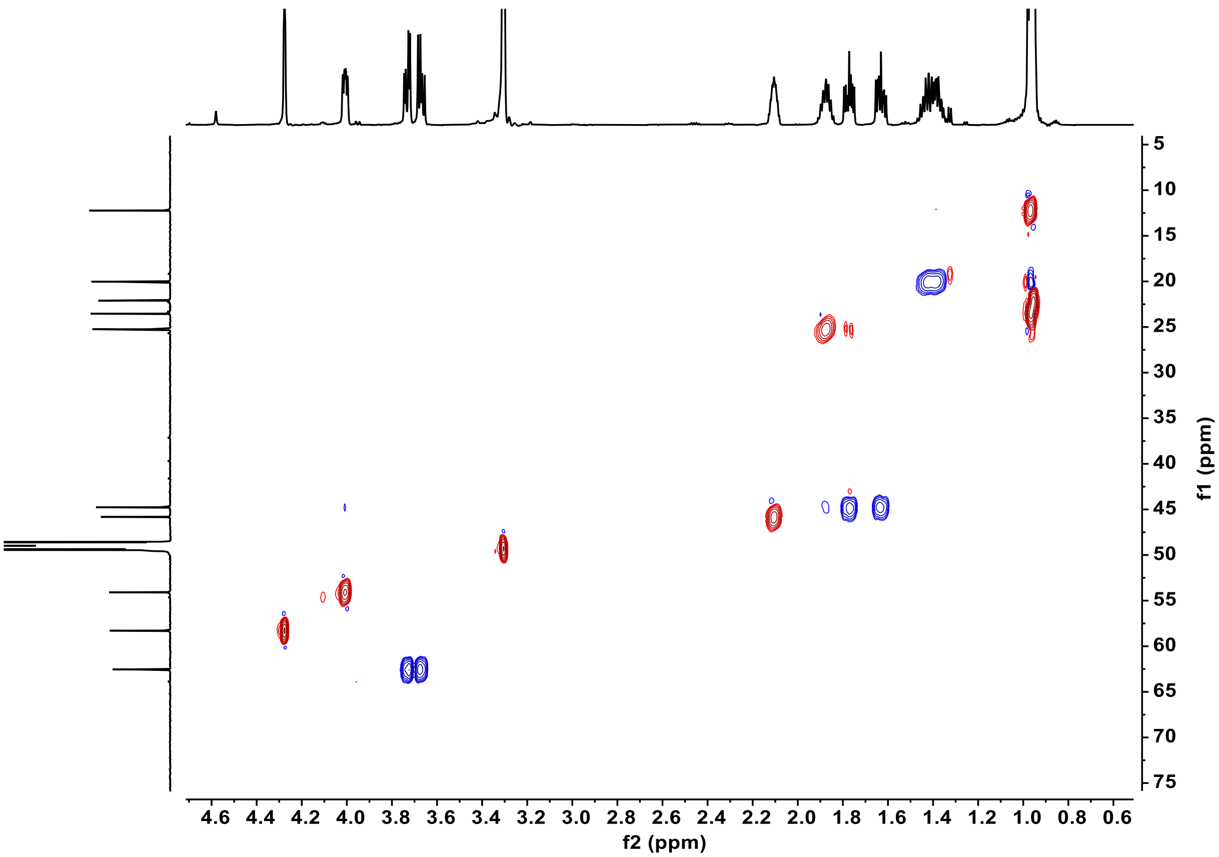

Supplementary Fig. 23. HSQC spectrum of **1a** (600 MHz, CD_3OD).

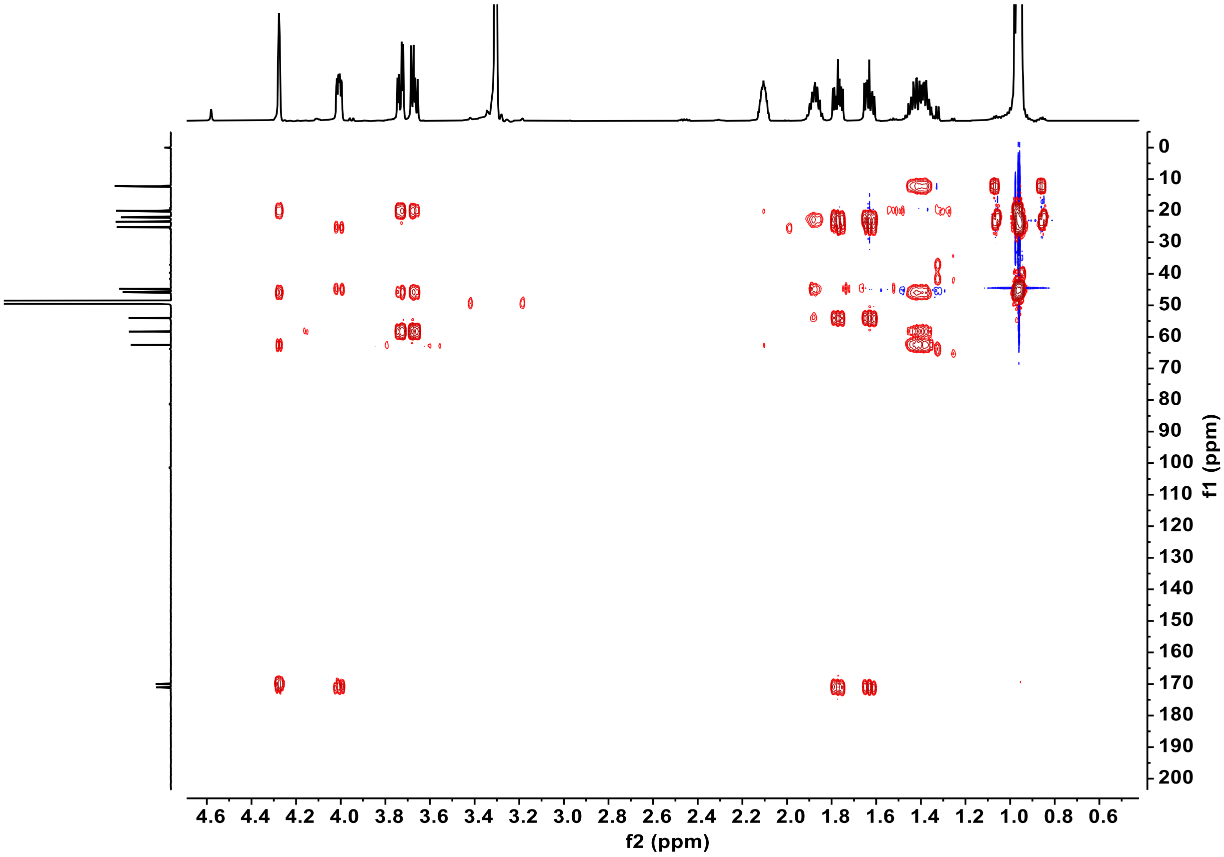

Supplementary Fig. 24. HMBC spectrum of **1a** (600 MHz, CD_3OD).

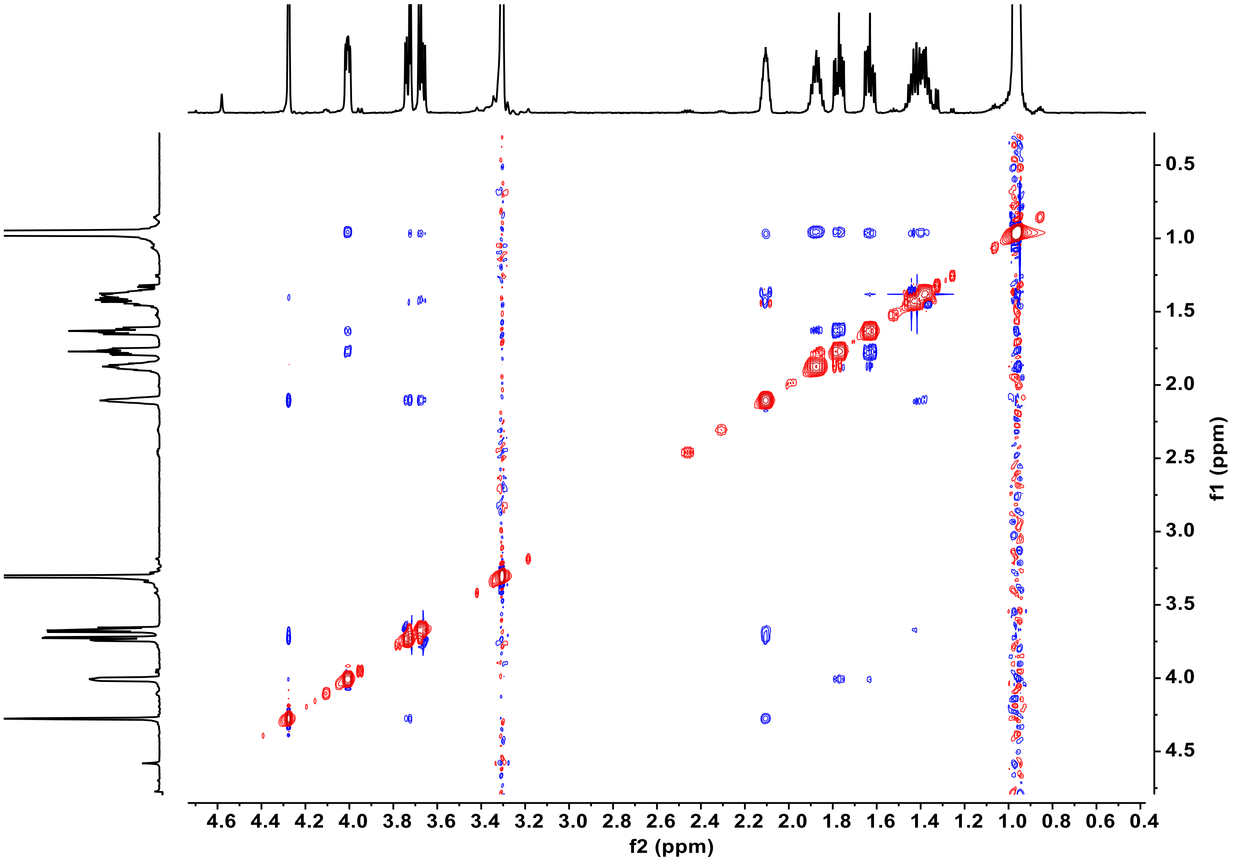

Supplementary Fig. 25. ^{1}H-^{1}H NOESY spectrum of **1a** (600 MHz, CD_3OD).

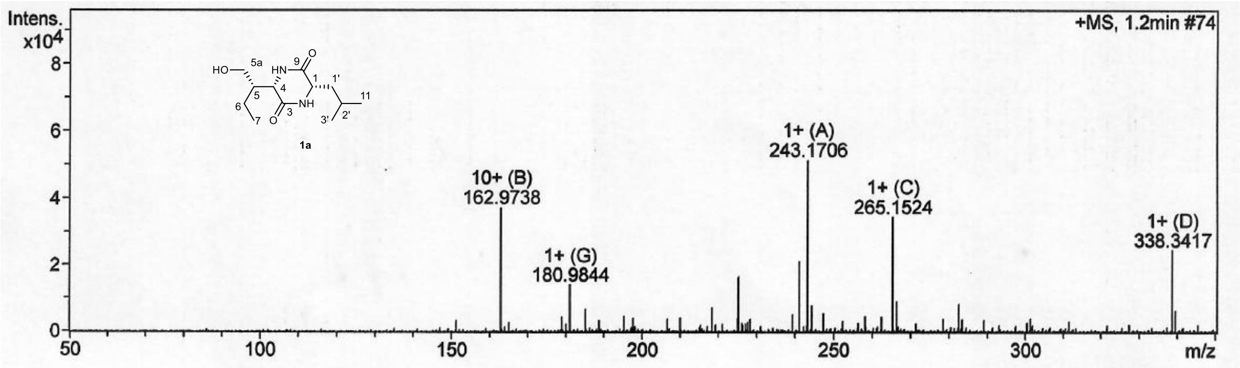

Meas. m/z	#	Formula	Score	m/z	err [ppm]	Mean err [ppm]	mSigma	rdb	e⁻ Conf	N-Rule
243.1706	1	C 12 H 23 N 2 O 3	100.00	243.1703	-1.2	-1.4	10.4	2.5	even	ok
265.1524	1	C 12 H 22 N 2 Na O 3	100.00	265.1523	-0.4	-1.7	74.1	2.5	even	ok

Supplementary Fig. 26. HR-ESI-MS (positive) spectrum of **1a**.

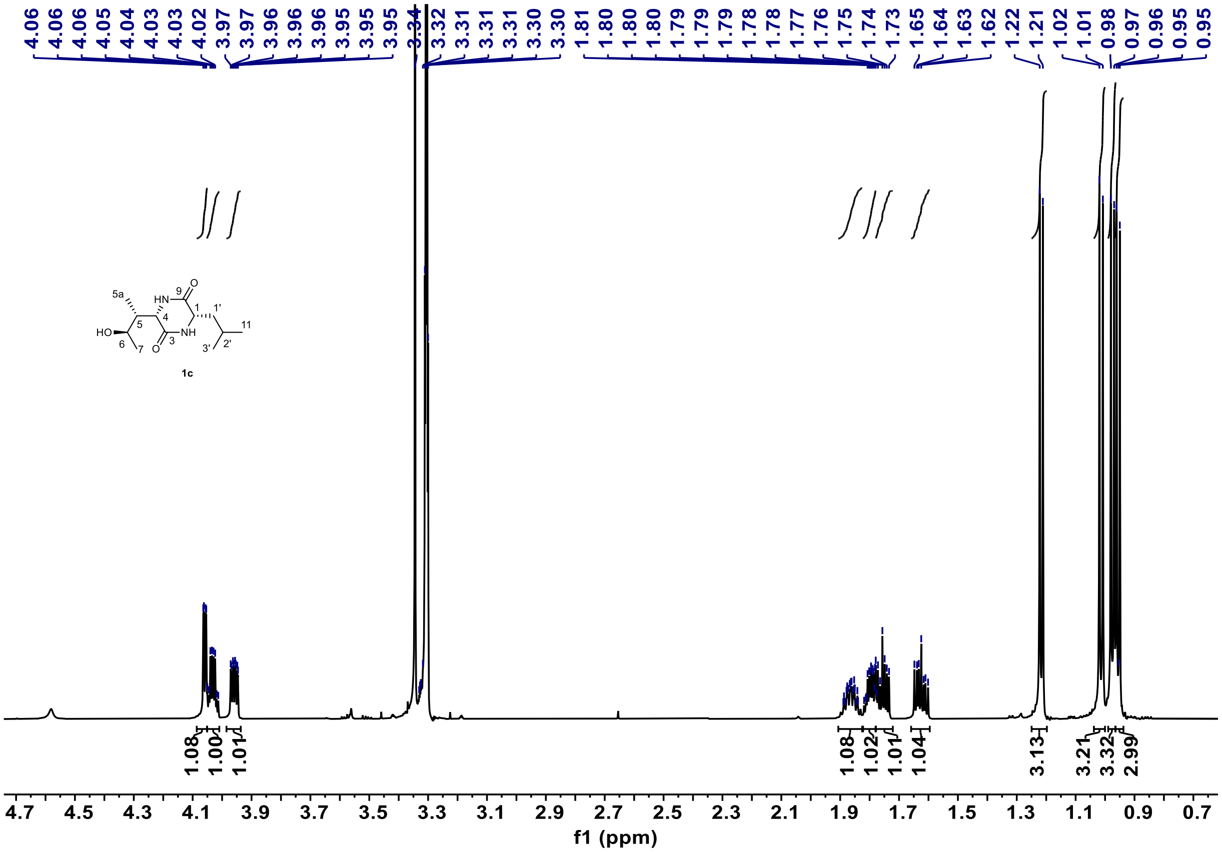

Supplementary Fig. 27. ^{1}H NMR spectrum of **1c** (600 MHz, CD_3OD).

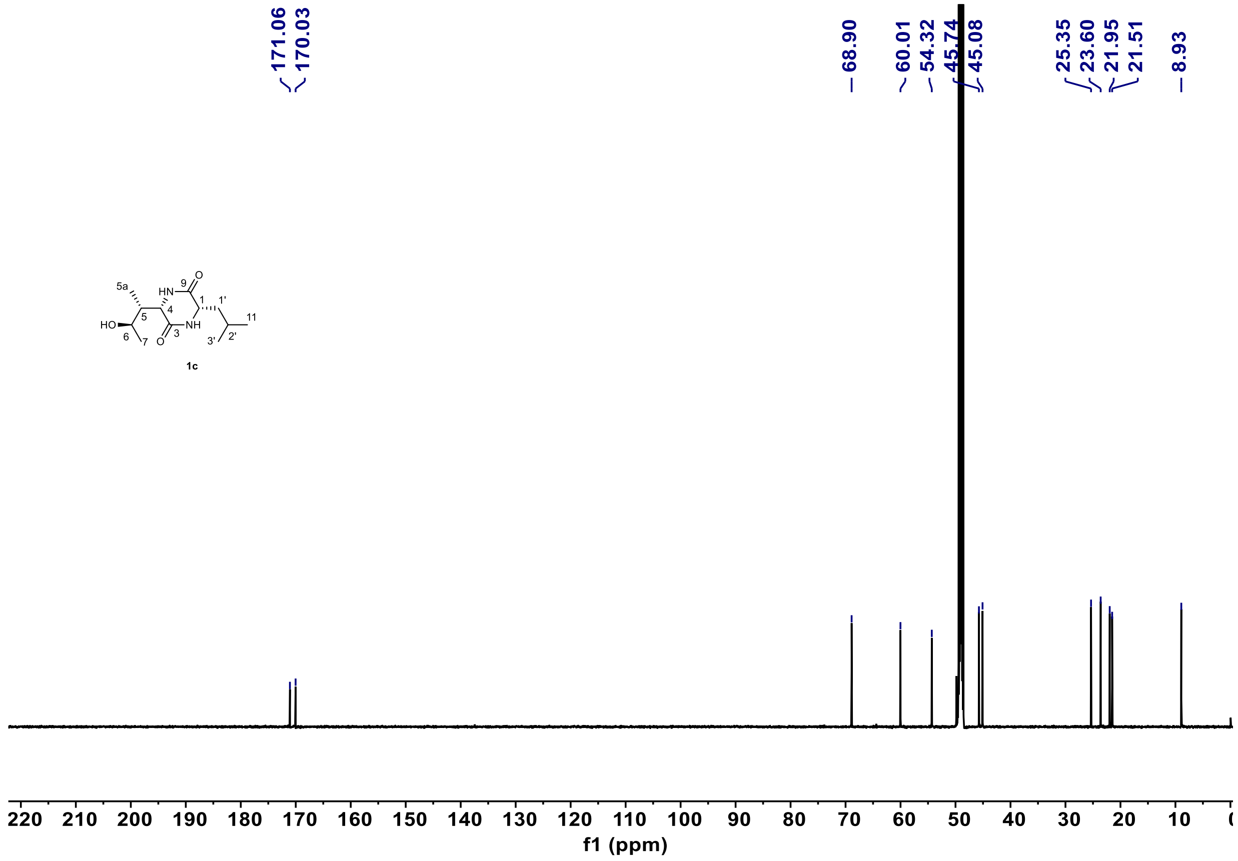

Supplementary Fig. 28. ^{13}C NMR spectrum of **1c** (150 MHz, CD_3OD).

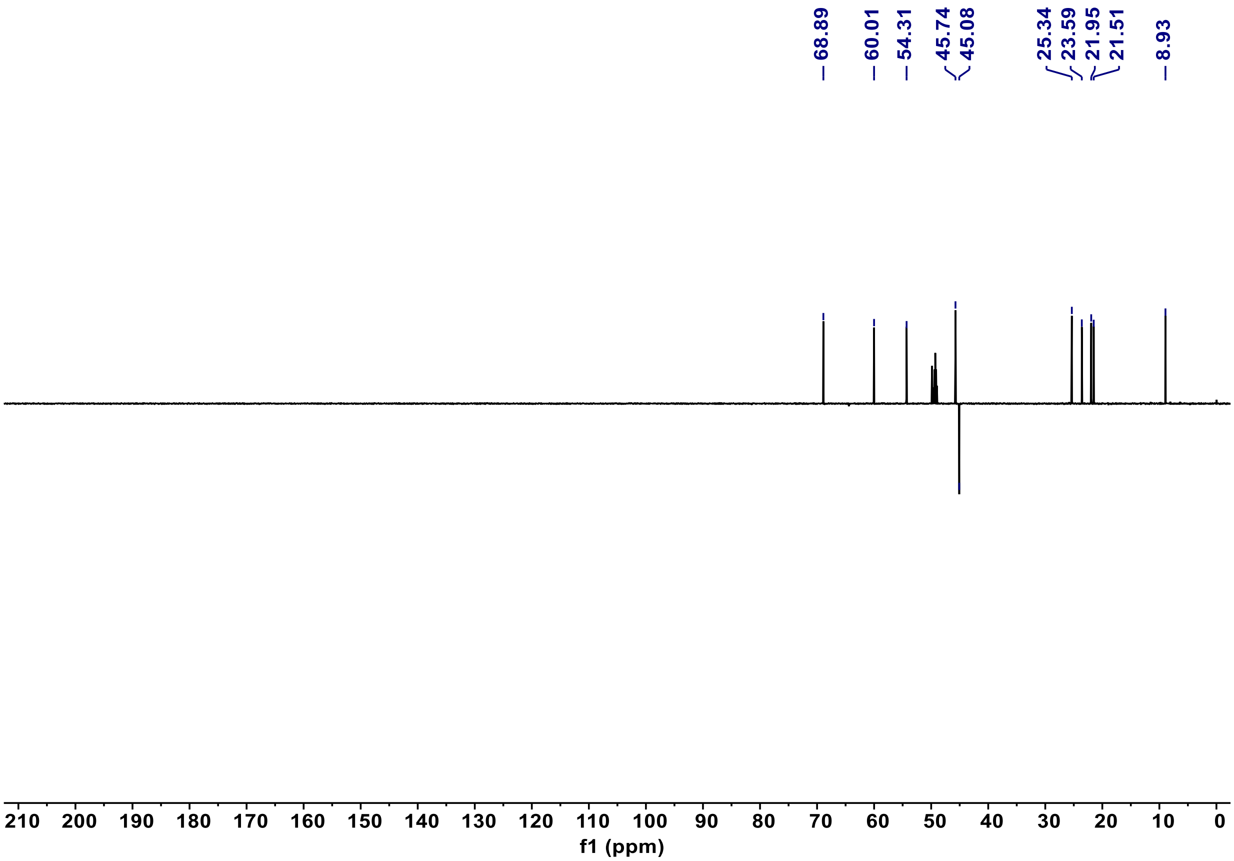

Supplementary Fig. 29. DEPT135 spectrum of **1c** (150 MHz, CD_3OD).

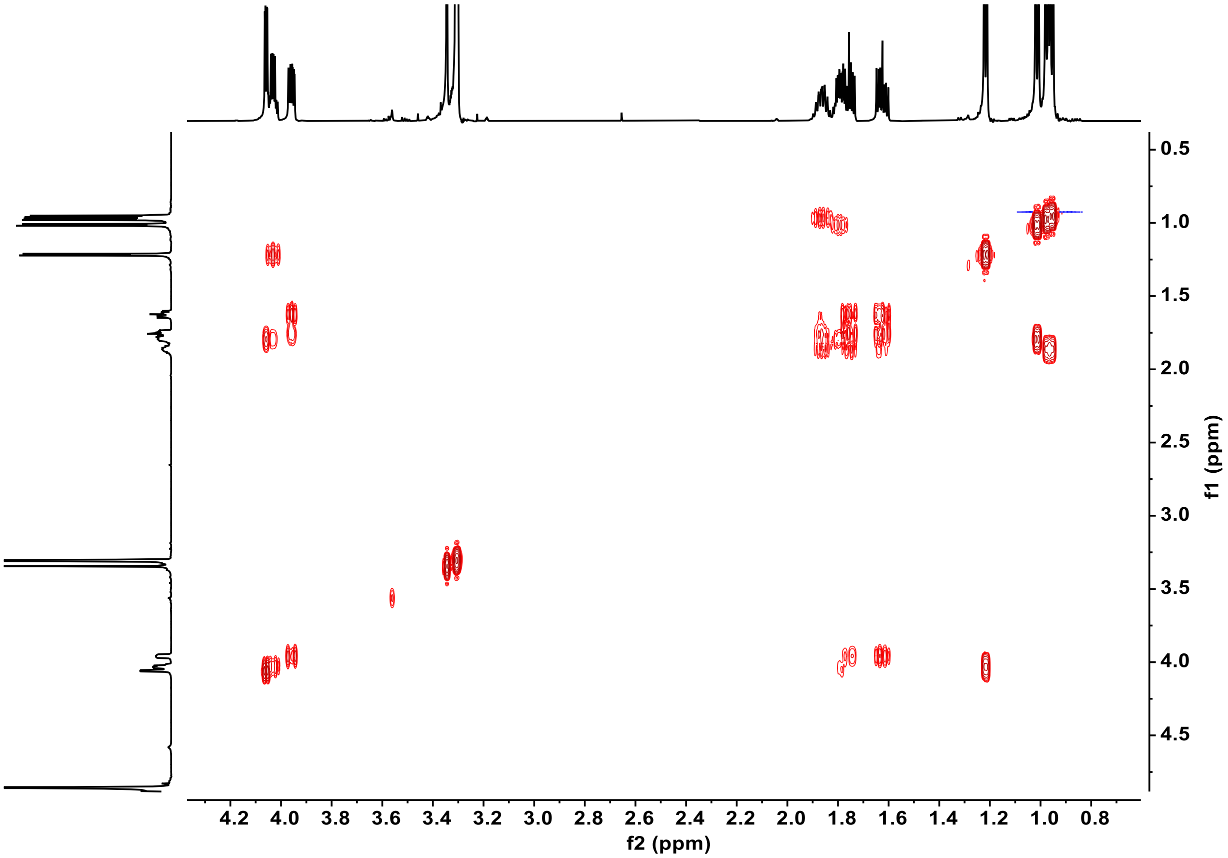

Supplementary Fig. 30. ^{1}H-^{1}H COSY spectrum of **1c** (600 MHz, CD_3OD).

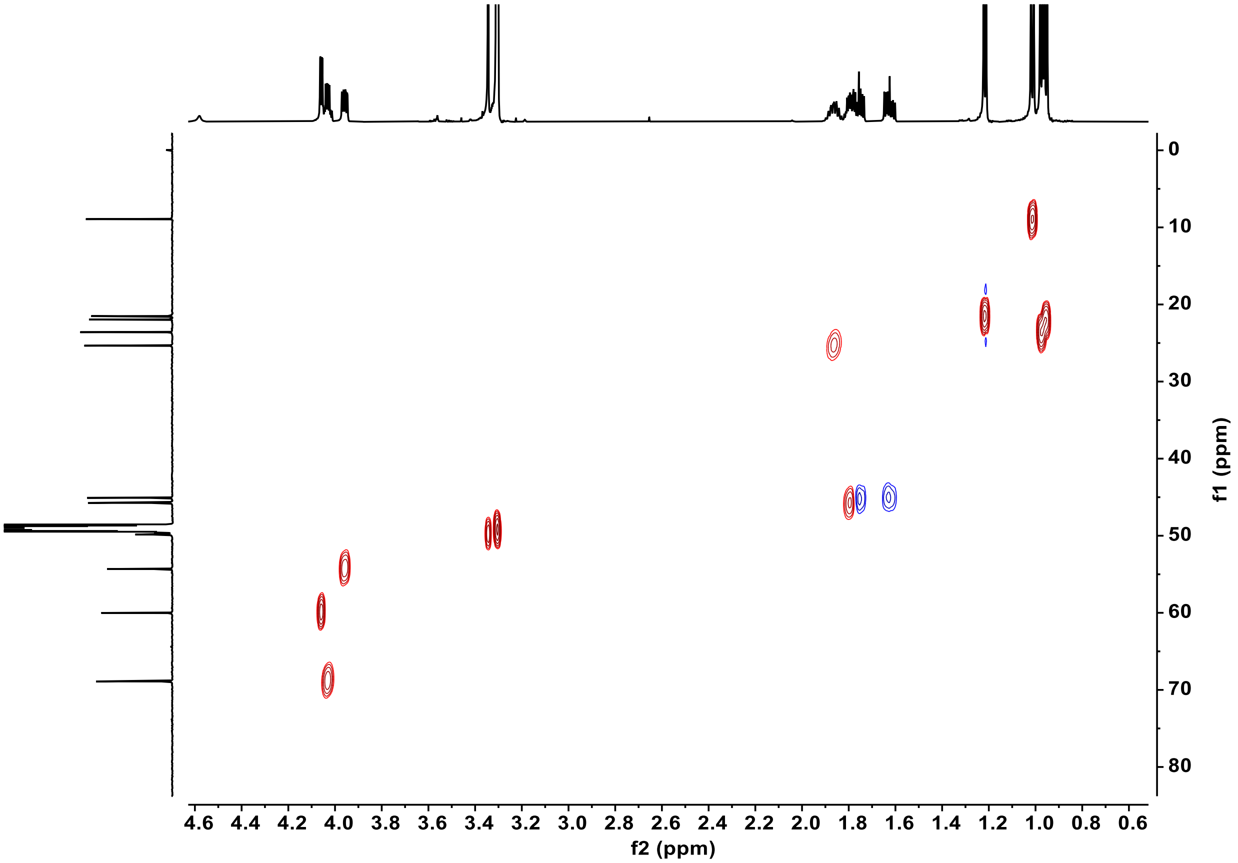

Supplementary Fig. 31. HSQC spectrum of **1c** (600 MHz, CD_3OD).

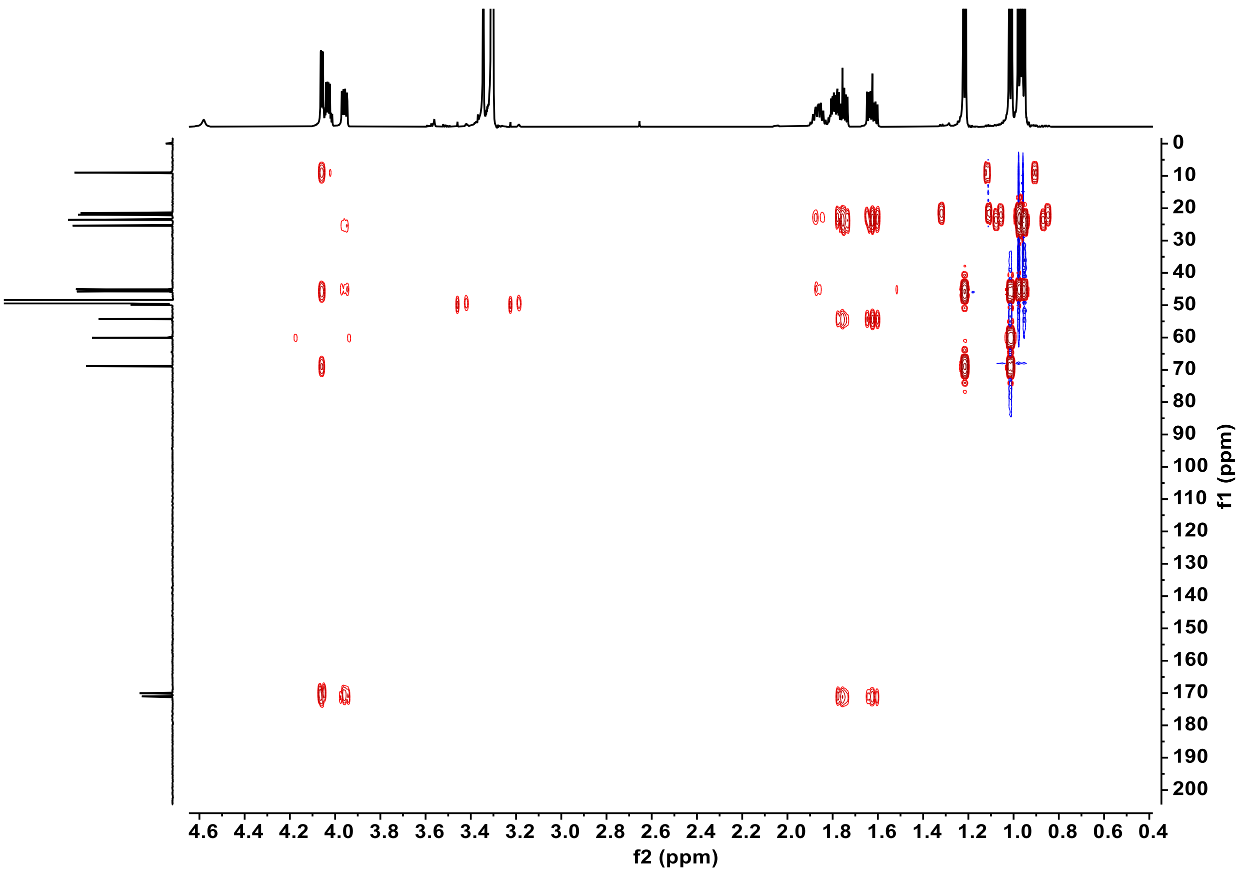

Supplementary Fig. 32. HMBC spectrum of **1c** (600 MHz, CD_3OD).

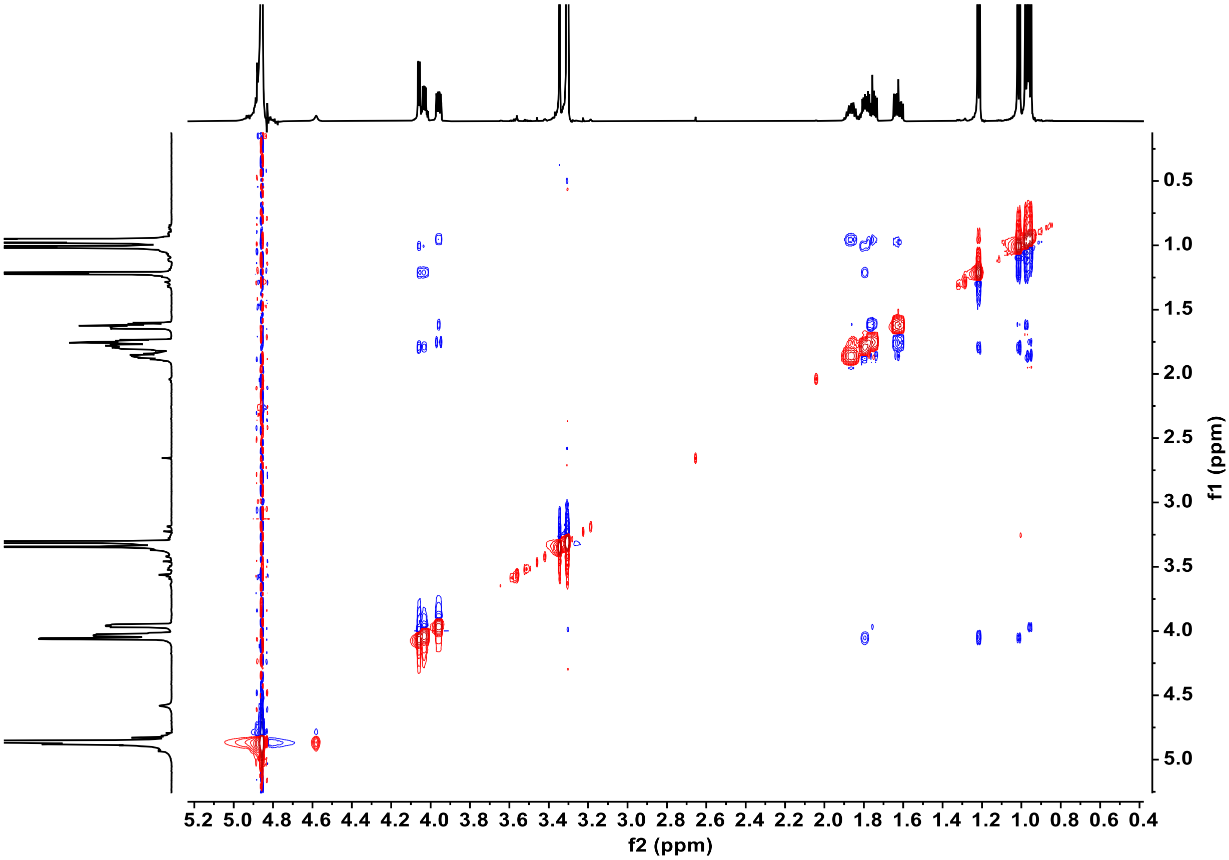

Supplementary Fig. 33. ^{1}H-^{1}H NOESY spectrum of **1c** (600 MHz, CD_3OD).

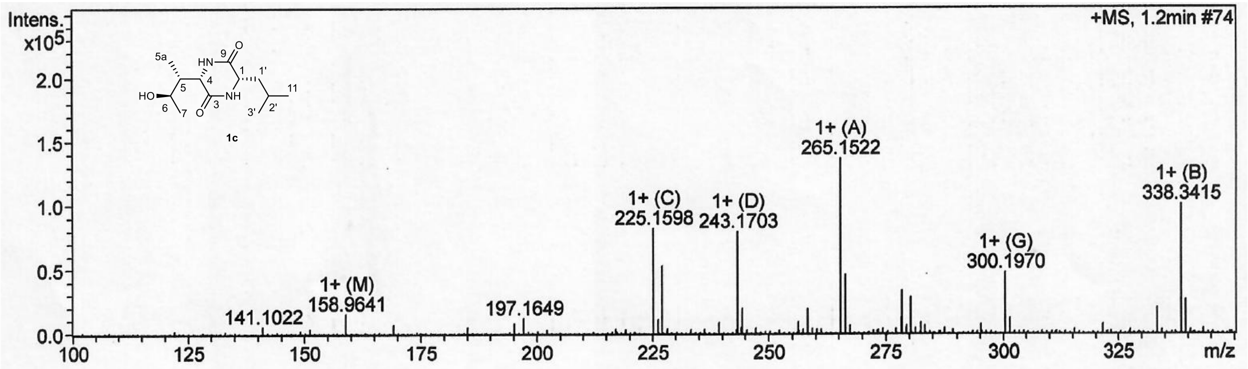

Meas. m/z	#	Formula	Score	m/z	err [ppm]	Mean err [ppm]	mSigma	rdb	e⁻ Conf	N-Rule
243.1703	1	C 12 H 23 N 2 O 3	100.00	243.1703	0.1	-1.2	66.1	2.5	even	ok
265.1522	1	C 12 H 22 N 2 Na O 3	100.00	265.1523	0.1	-1.9	115.2	2.5	even	ok

Supplementary Fig. 34. HR-ESI-MS (positive) spectrum of **1c**.

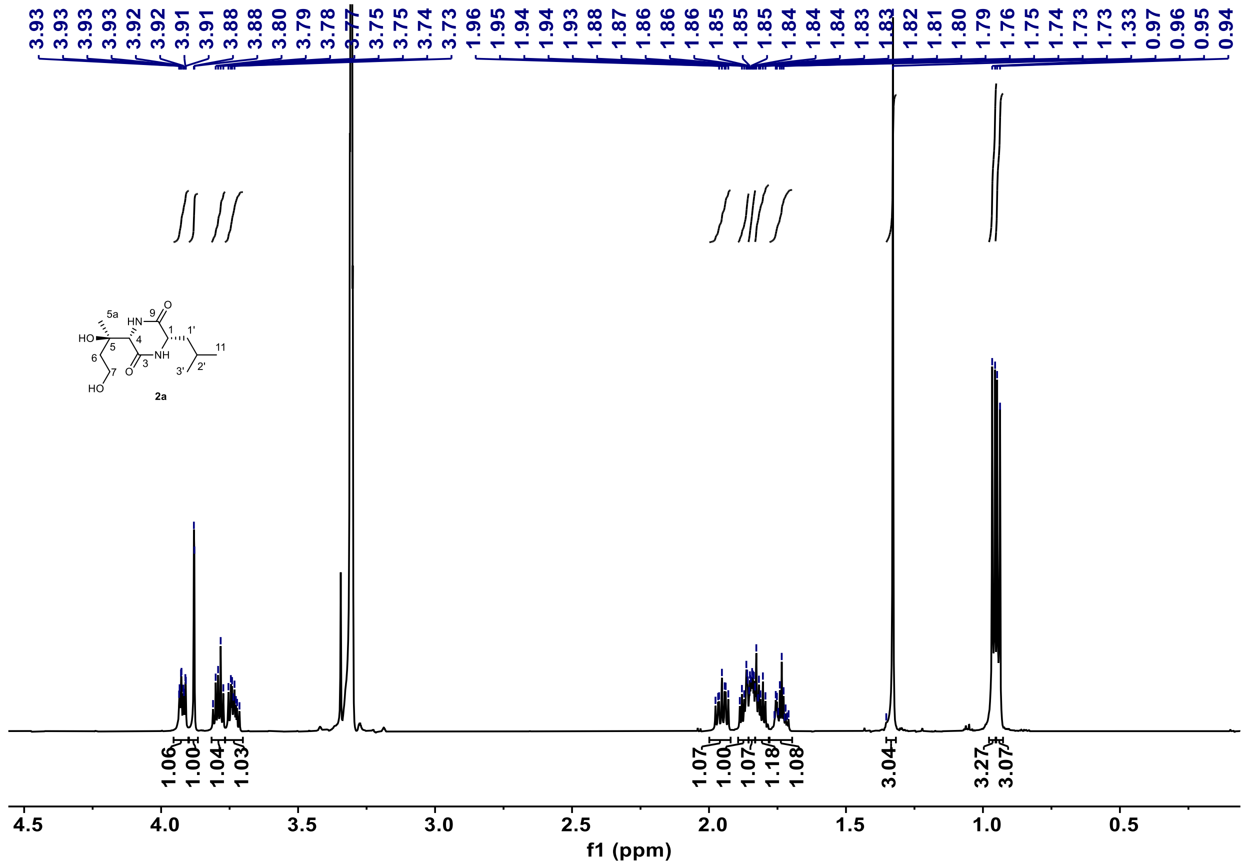

Supplementary Fig. 35. ^{1}H NMR spectrum of **2a** (600 MHz, CD_3OD).

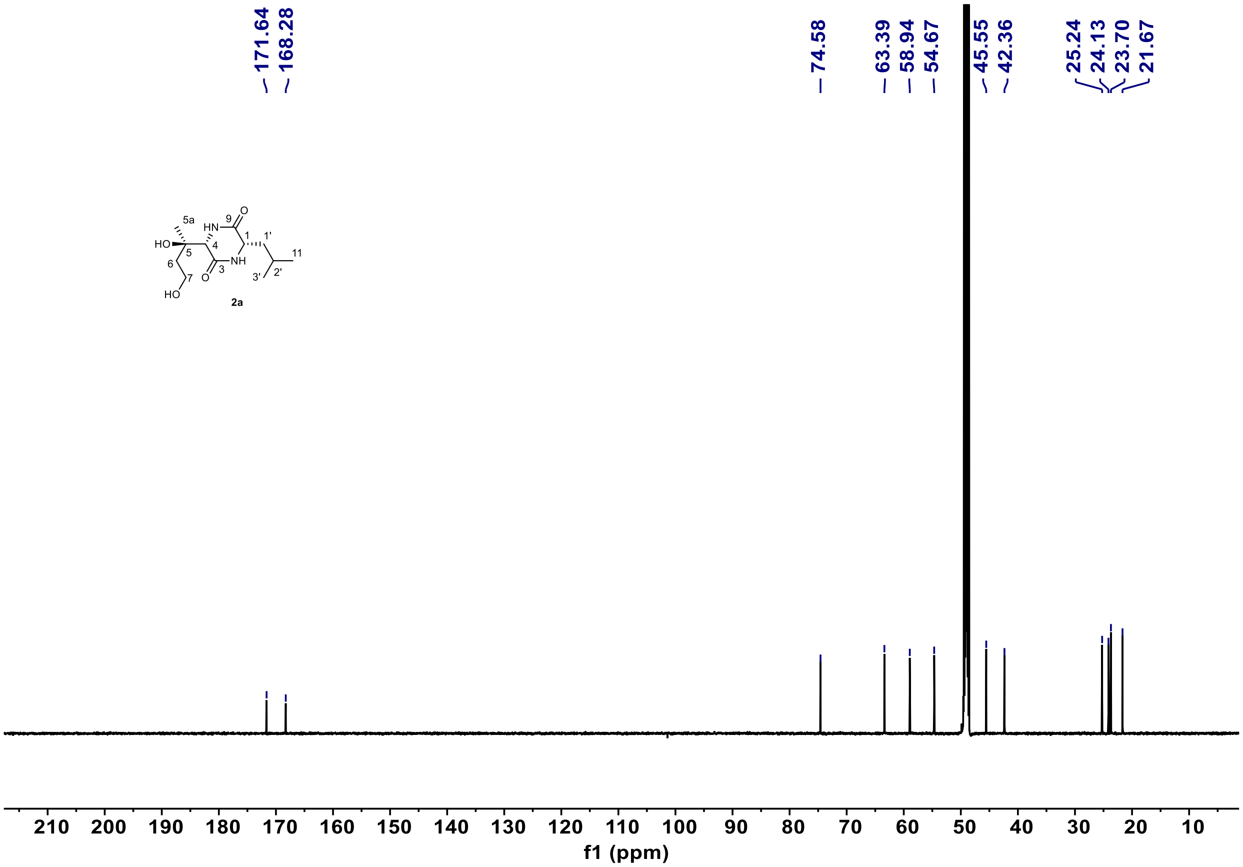

Supplementary Fig. 36. ^{13}C NMR spectrum of **2a** (150 MHz, CD_3OD).

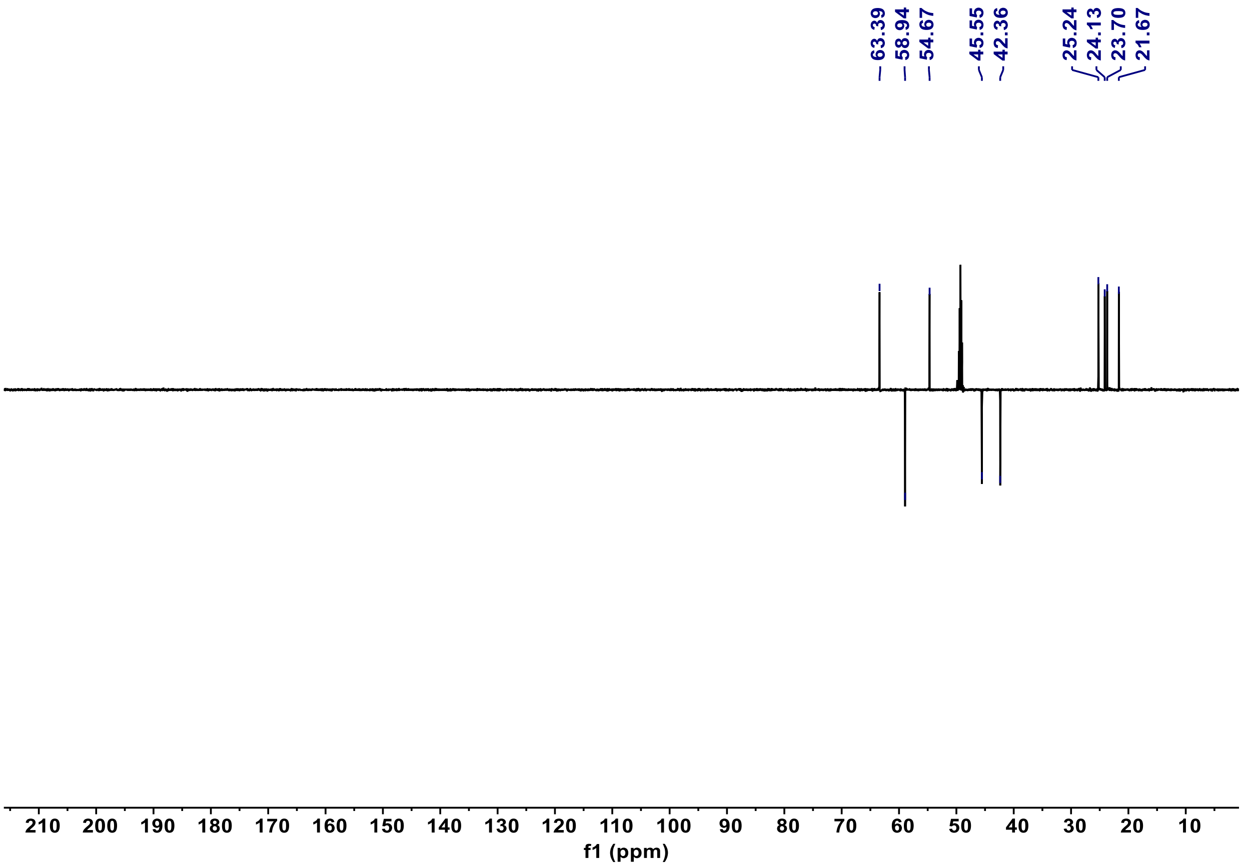

Supplementary Fig. 37. DEPT135 spectrum of **2a** (150 MHz, CD_3OD).

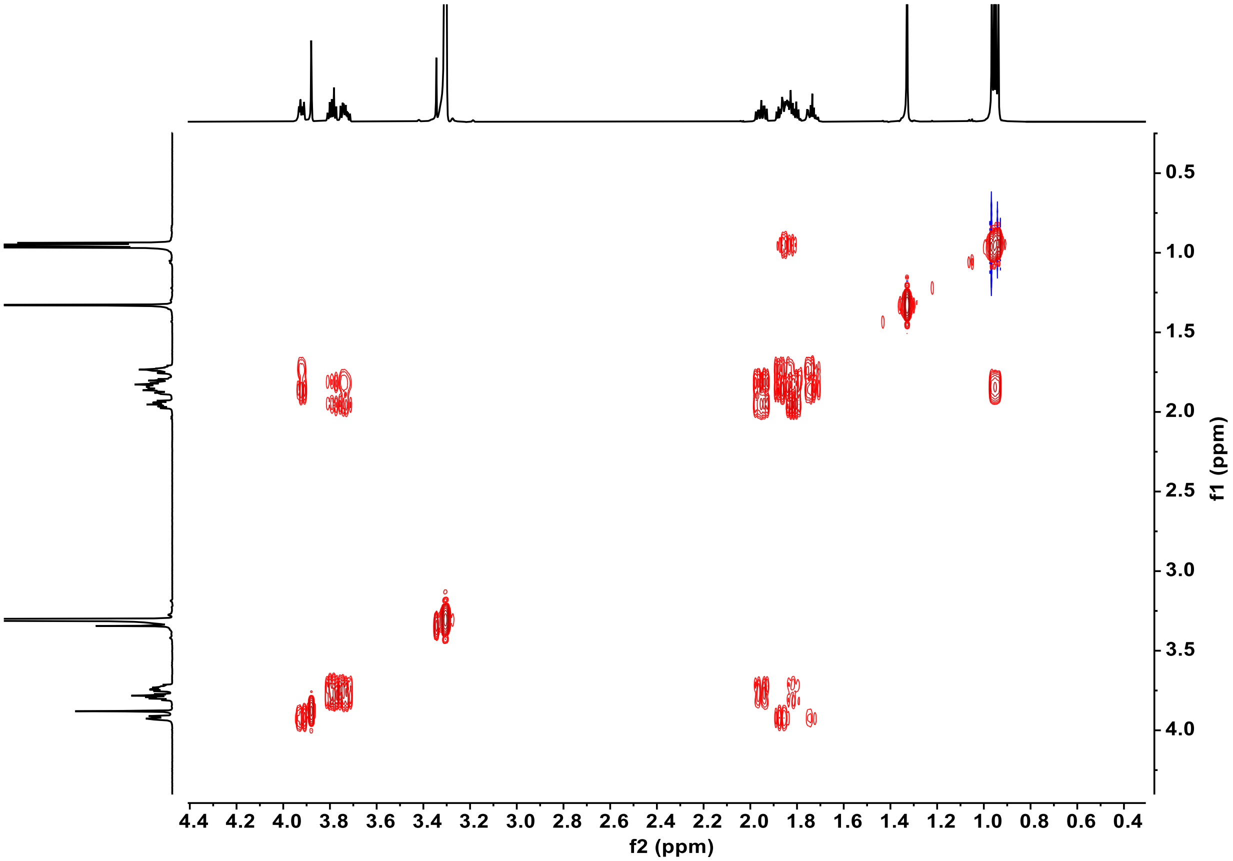

Supplementary Fig. 38. ^1H-^1H COSY spectrum of **2a** (600 MHz, CD_3OD).

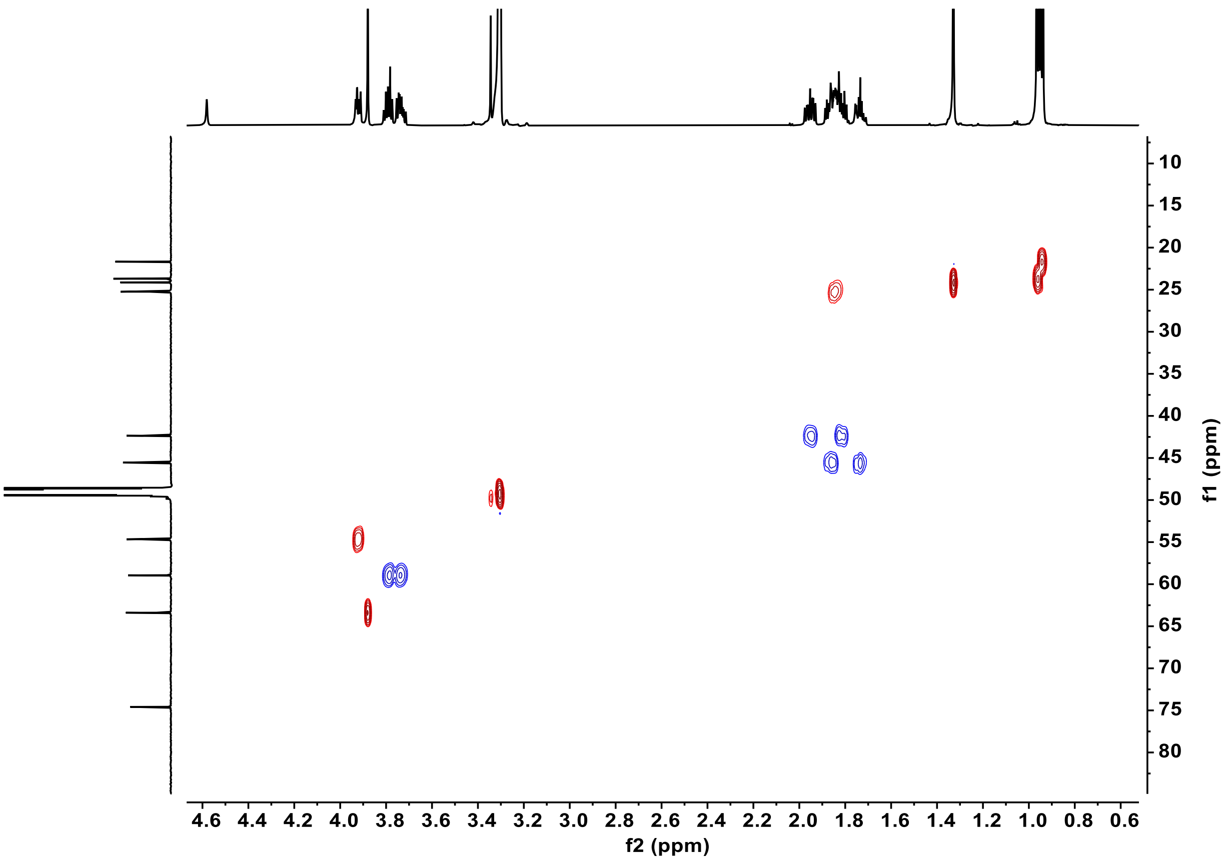

Supplementary Fig. 39. HSQC spectrum of **2a** (600 MHz, CD_3OD).

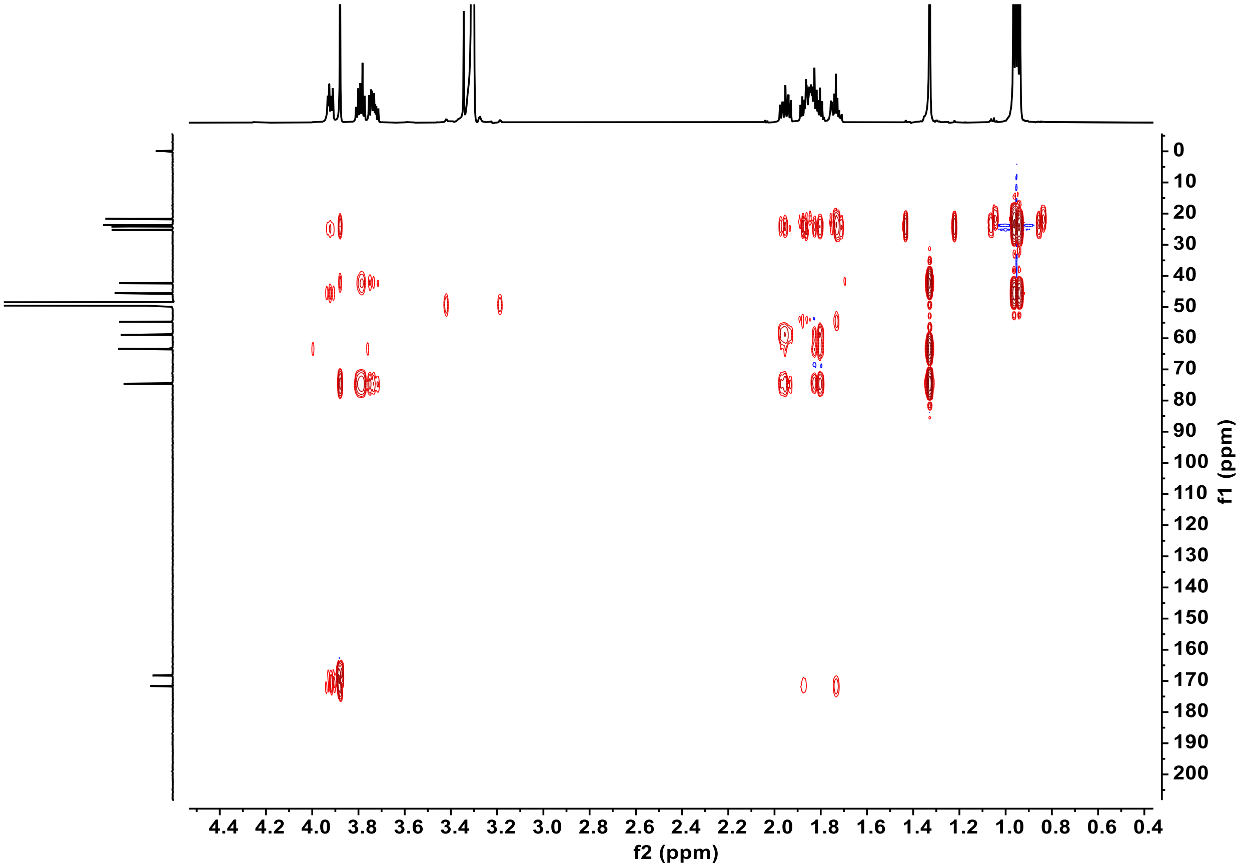

Supplementary Fig. 40. HMBC spectrum of **2a** (600 MHz, CD_3OD).

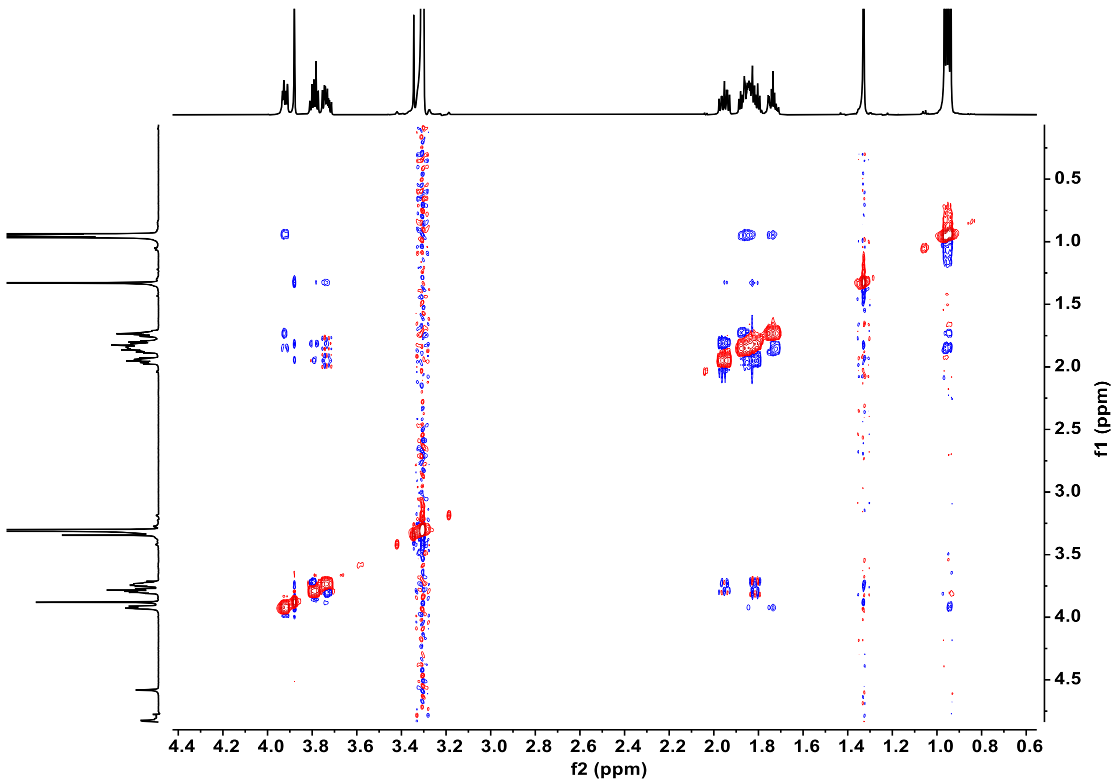

Supplementary Fig. 41. ^{1}H-^{1}H NOESY spectrum of **2a** (600 MHz, CD_3OD).

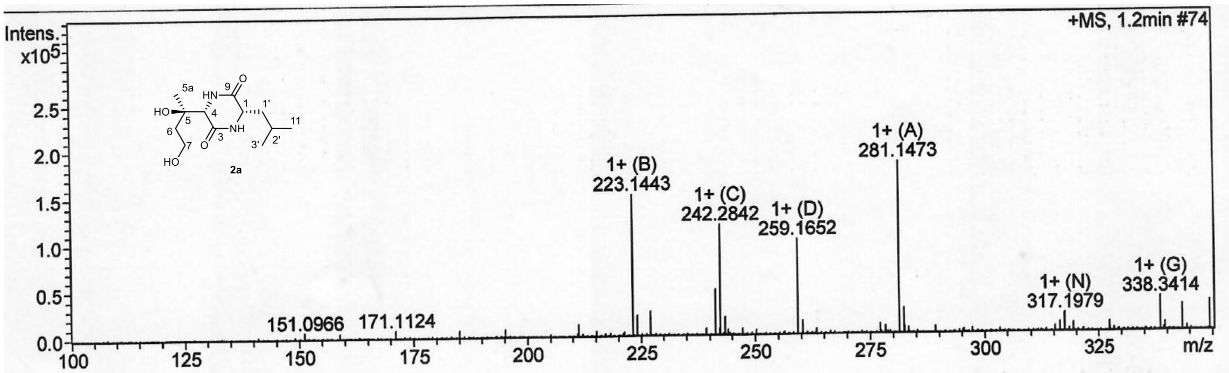

Meas. m/z	#	Formula	Score	m/z	err [ppm]	Mean err [ppm]	mSigma	rdb	e⁻ Conf	N-Rule
259.1652	1	C 12 H 23 N 2 O 4	100.00	259.1652	0.1	-0.0	2.3	2.5	even	ok
281.1473	1	C 12 H 22 N 2 Na O 4	100.00	281.1472	-0.4	-0.4	4.7	2.5	even	ok

Supplementary Fig. 42. HR-ESI-MS (positive) spectrum of **2a** (600 MHz, CD_3OD).

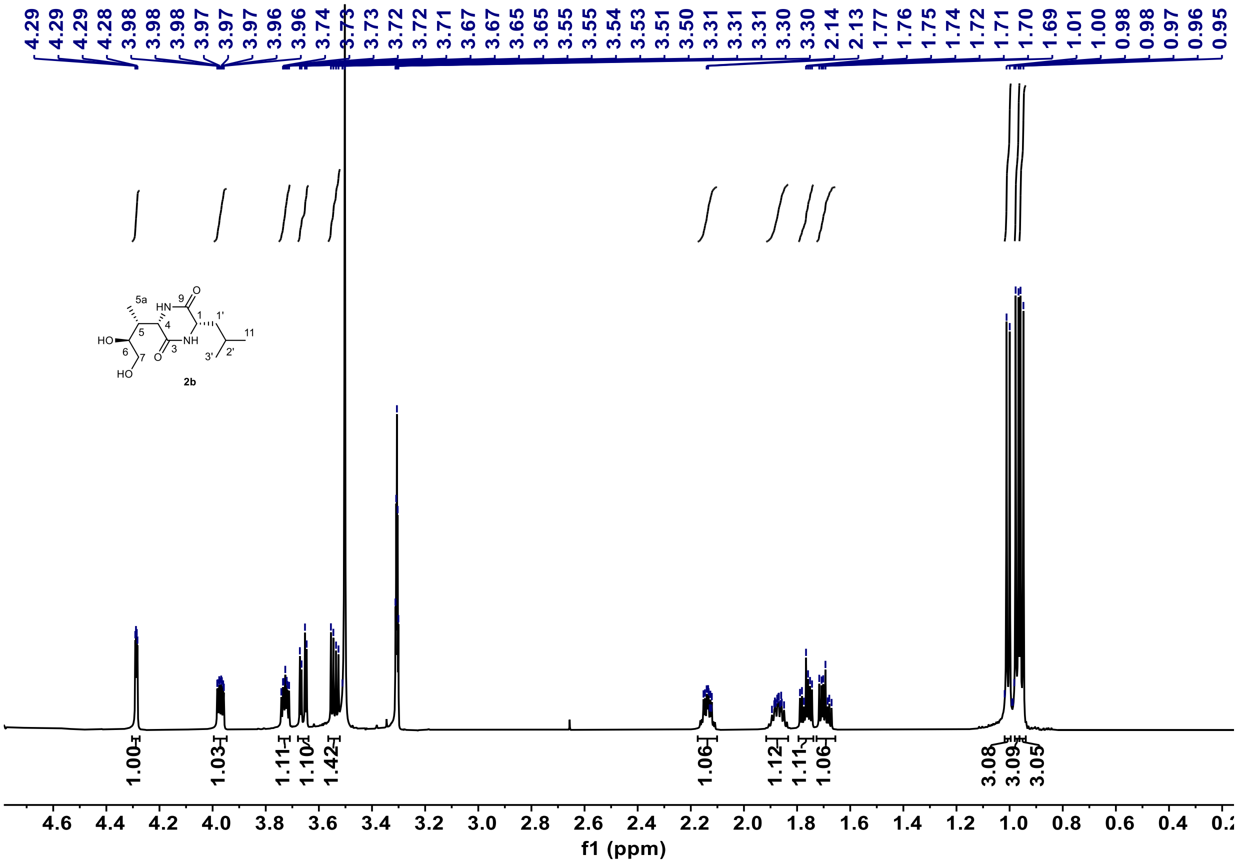

Supplementary Fig. 43. ^{1}H NMR spectrum of **2b** (600 MHz, CD_3OD).

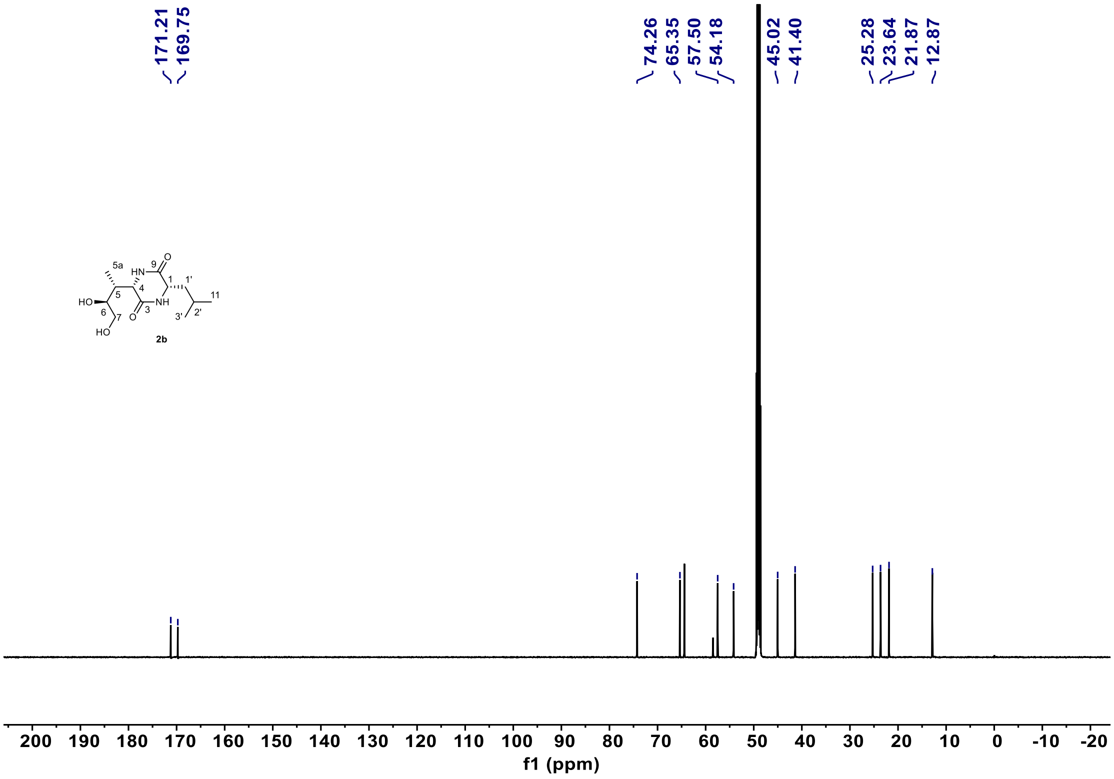

Supplementary Fig. 44. ^{13}C NMR spectrum of **2b** (150 MHz, CD_3OD).

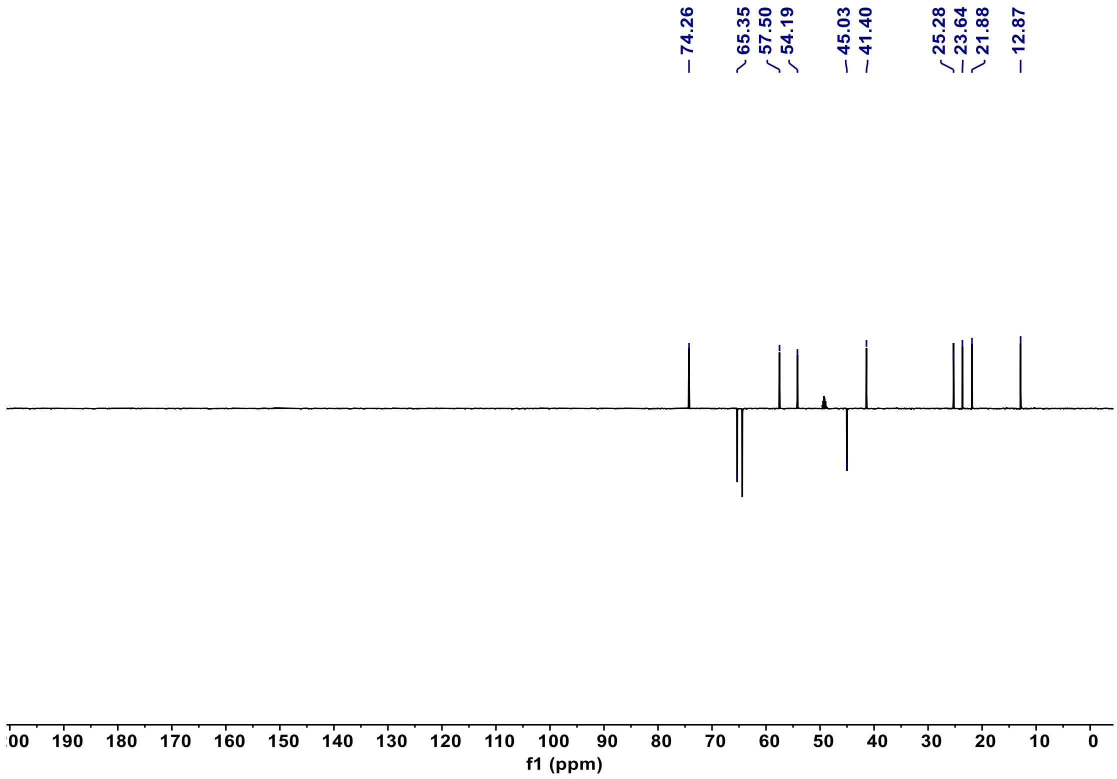

Supplementary Fig. 45. DEPT135 spectrum of **2b** (150 MHz, CD_3OD).

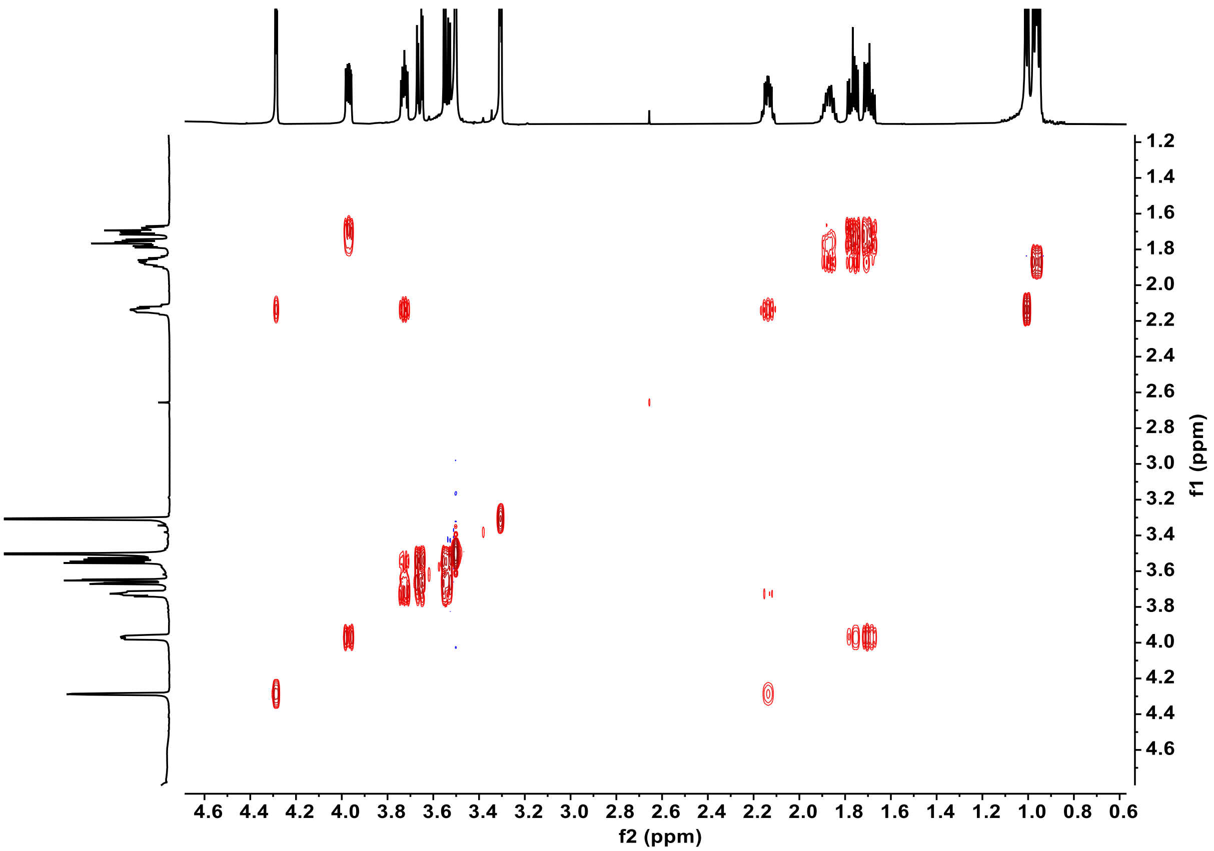

Supplementary Fig. 46. ^{1}H-^{1}H COSY spectrum of **2b** (600 MHz, CD_3OD).

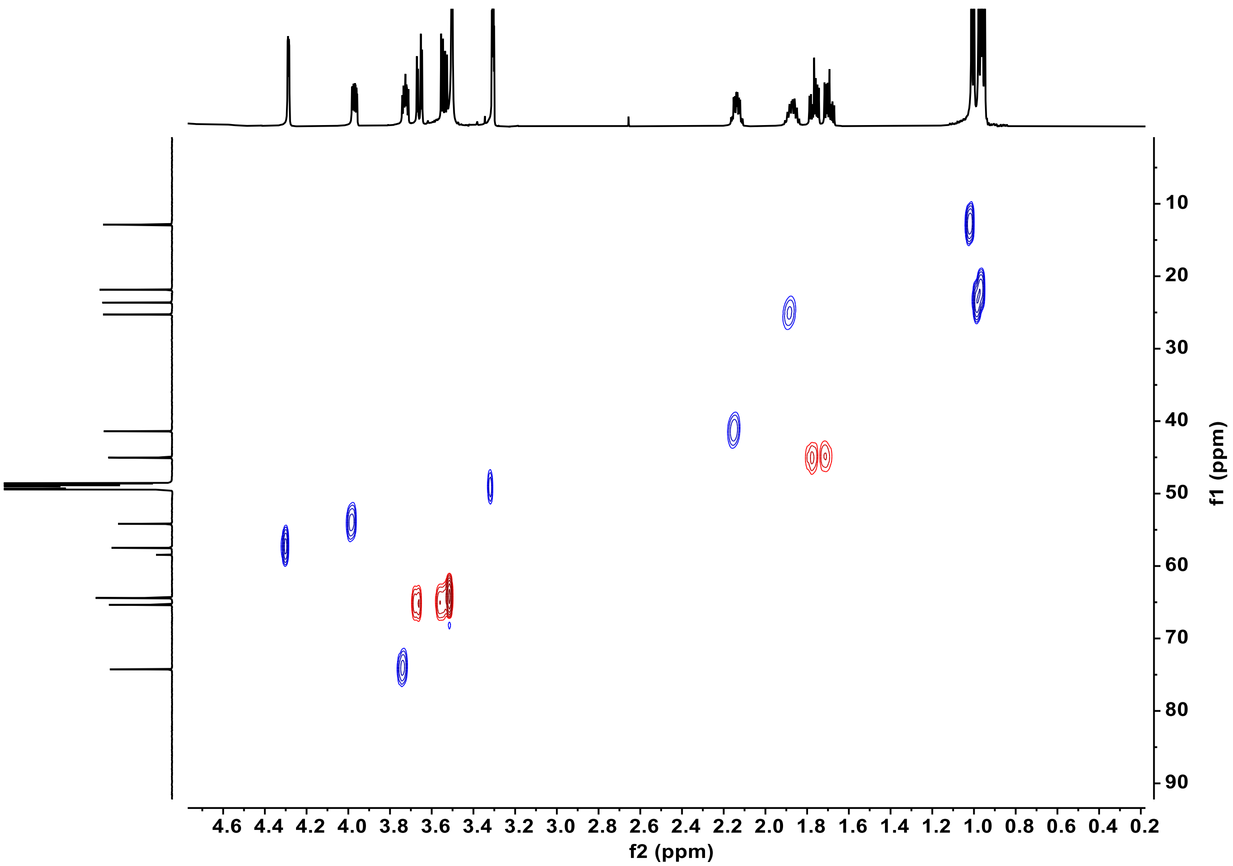

Supplementary Fig. 47. HSQC spectrum of **2b** (600 MHz, CD_3OD).

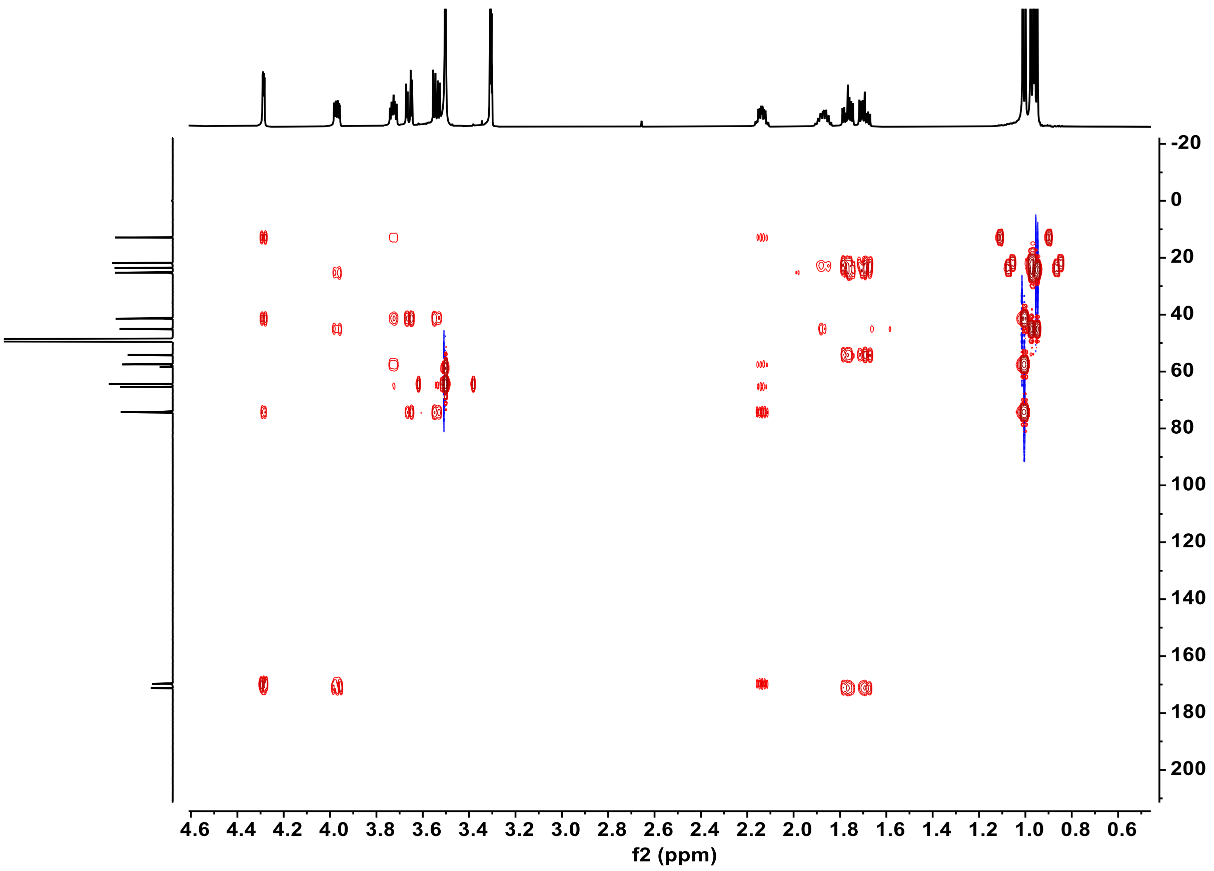

Supplementary Fig. 48. HMBC spectrum of **2b** (600 MHz, CD_3OD).

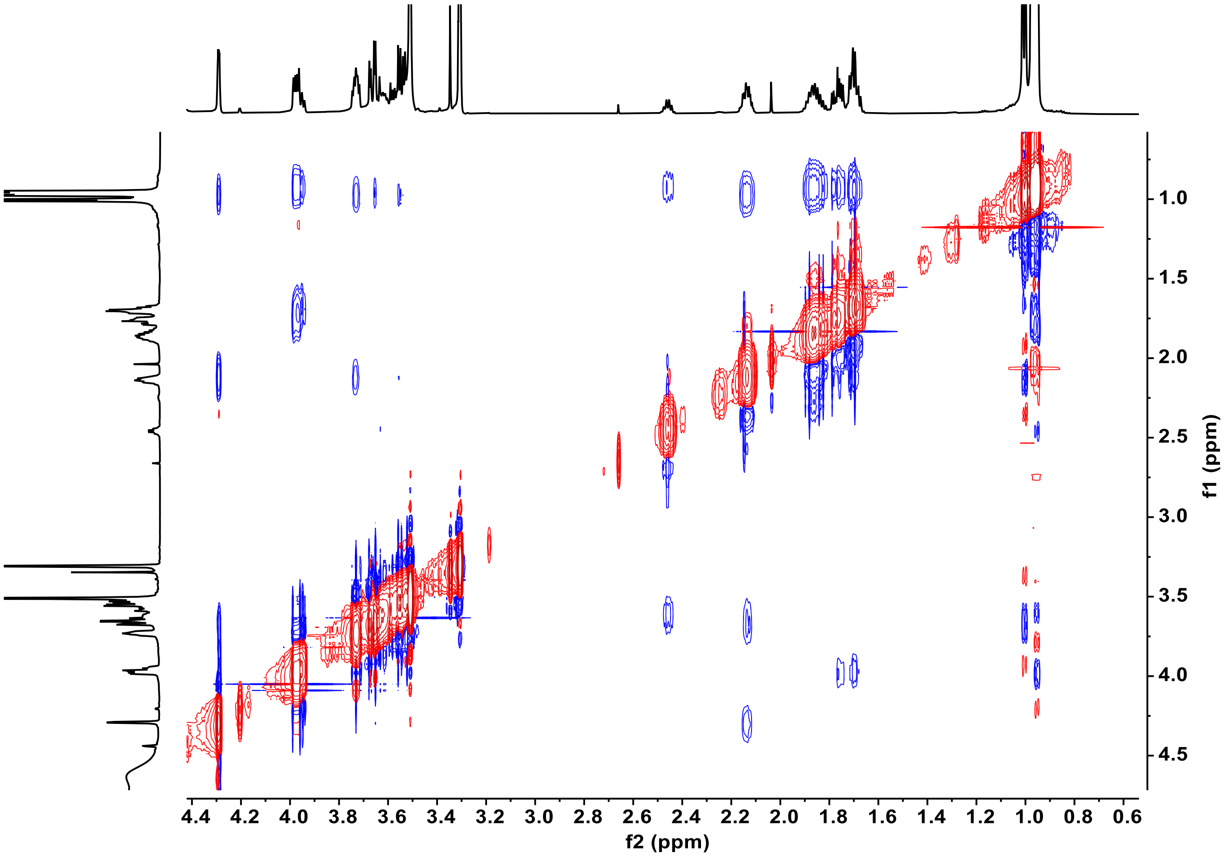

Supplementary Fig. 49. ^1H-^1H NOESY spectrum of **2b** (600 MHz, CD_3OD).

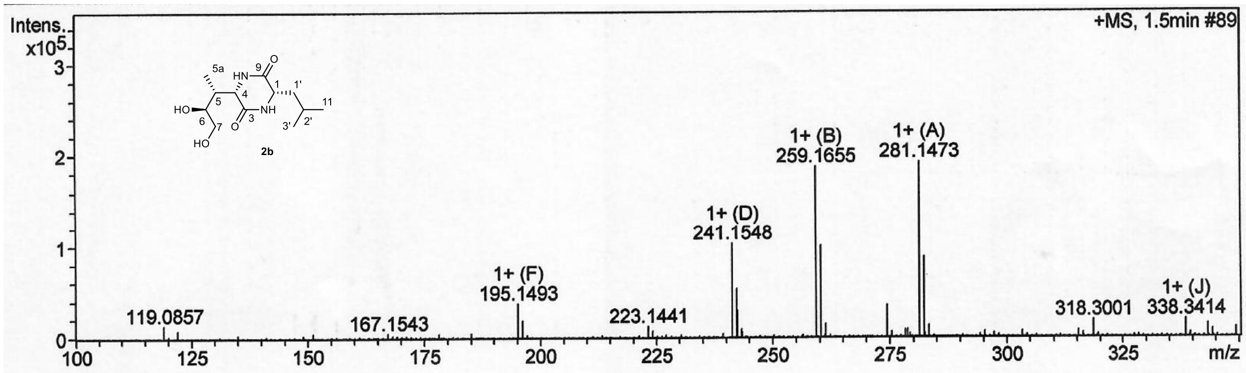

Meas. m/z	#	Formula	Score	m/z	err [ppm]	Mean err [ppm]	mSigma	rdb	e⁻ Conf	N-Rule
259.1655	1	C 12 H 23 N 2 O 4	100.00	259.1652	-1.2	-4.1	231.4	2.5	even	ok
281.1473	1	C 12 H 22 N 2 Na O 4	100.00	281.1472	-0.6	-3.5	188.4	2.5	even	ok

Supplementary Fig. 50. HR-ESI-MS (positive) spectrum of **2b**.

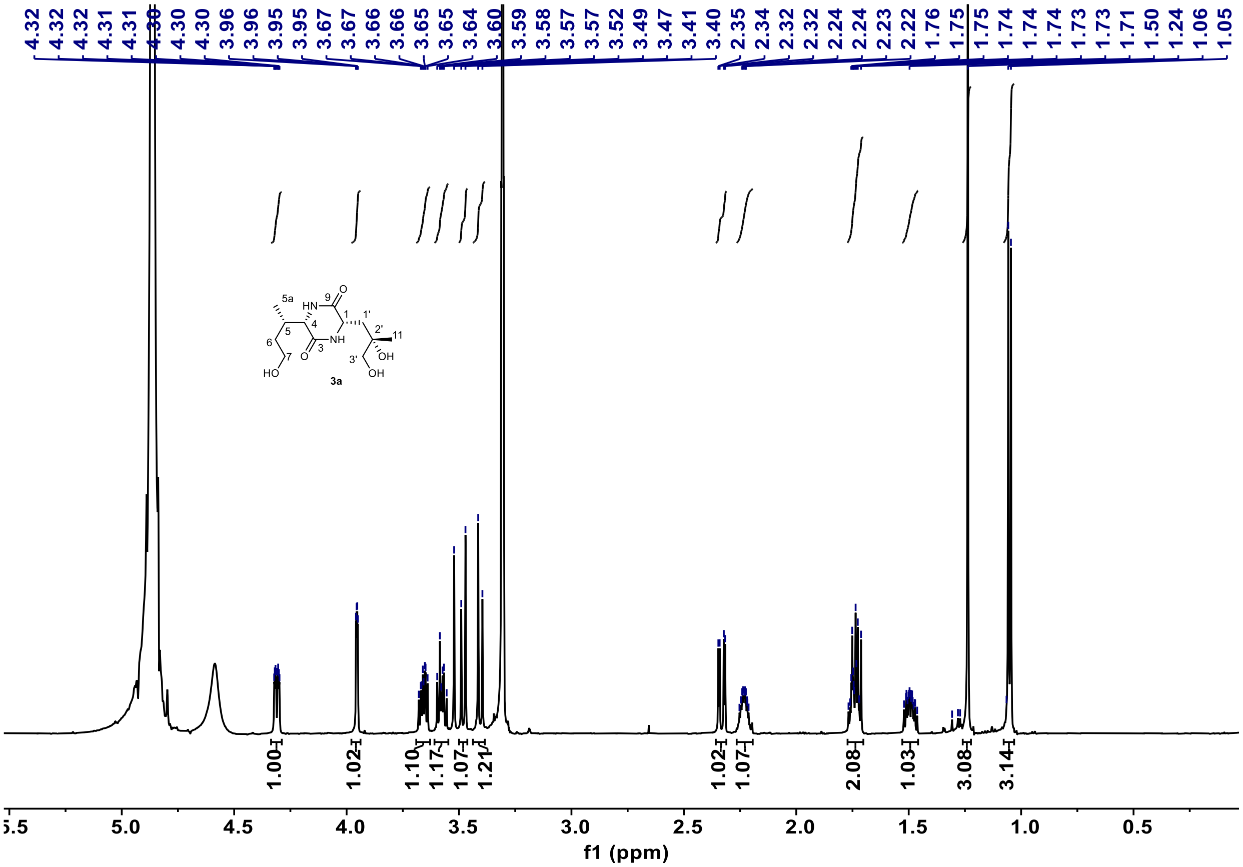

Supplementary Fig. 51. ^{1}H NMR spectrum of **3a** (600 MHz, CD_3OD).

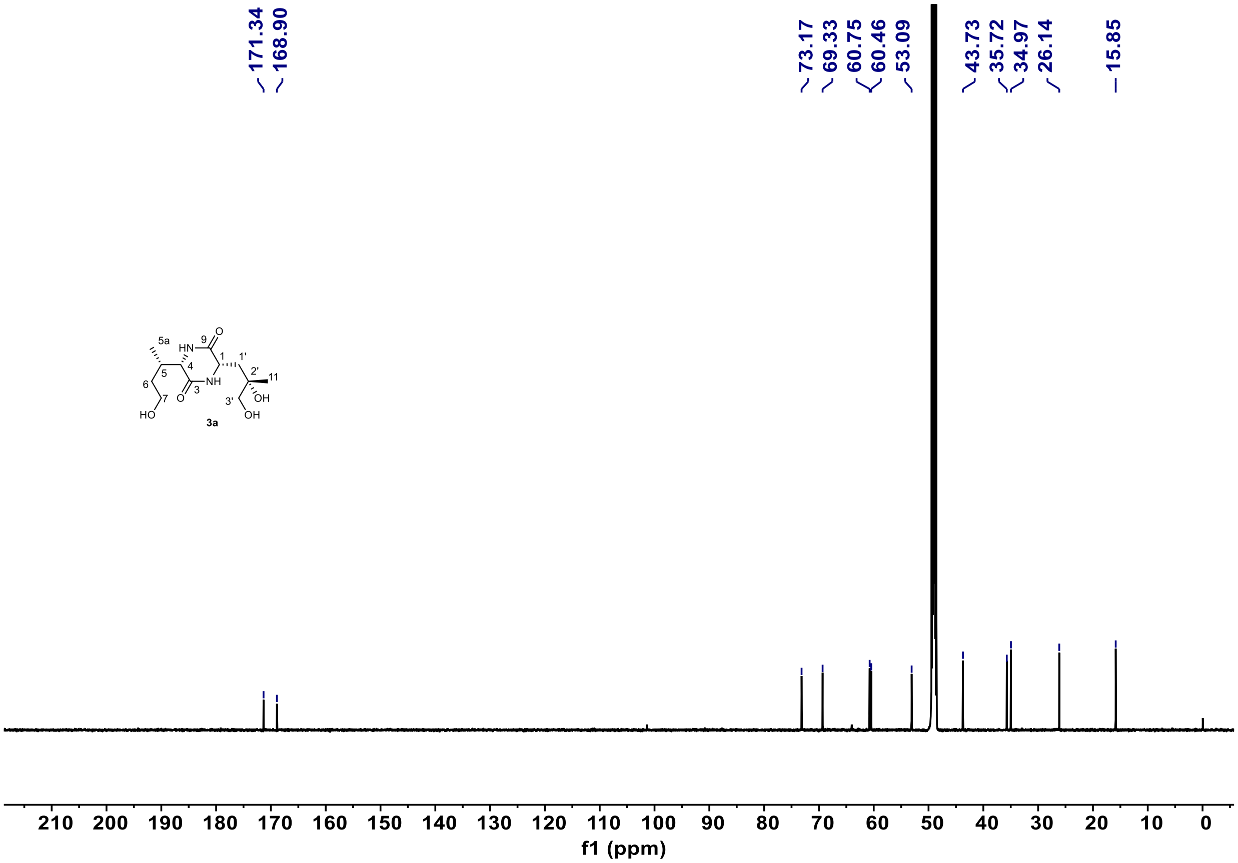

Supplementary Fig. 52. ^{13}C NMR spectrum of **3a** (150 MHz, CD_3OD).

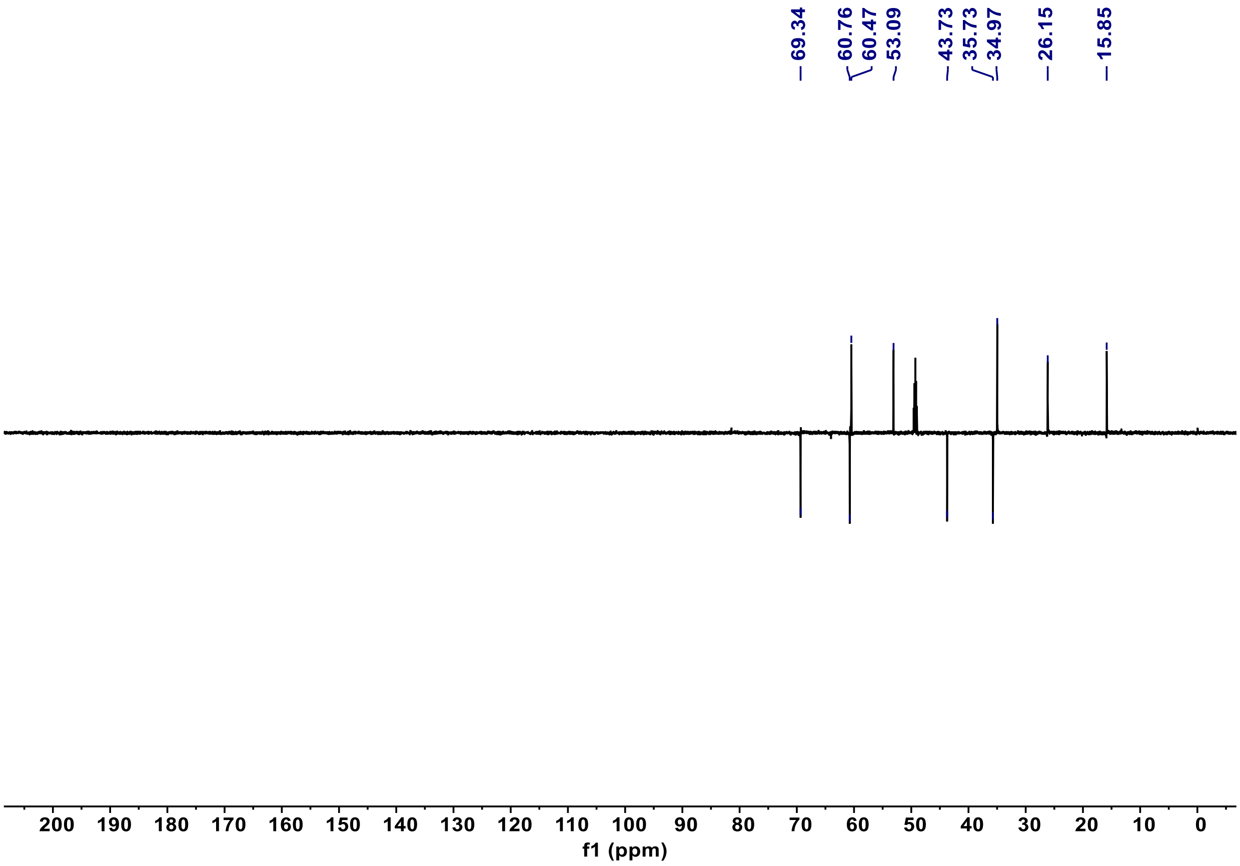

Supplementary Fig. 53. DEPT135 spectrum of **3a** (150 MHz, CD_3OD).

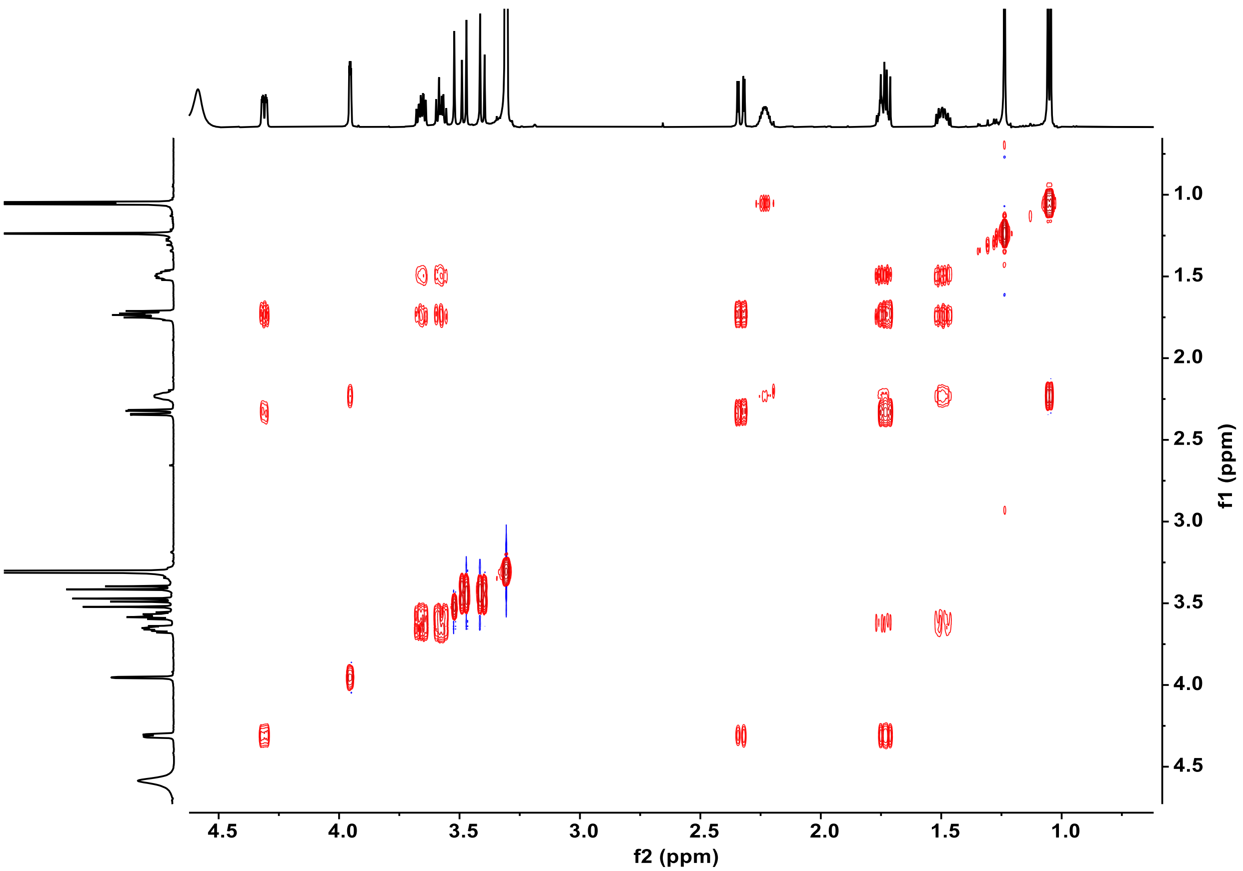

Supplementary Fig. 54. ^1H-^1H COSY spectrum of **3a** (600 MHz, CD_3OD).

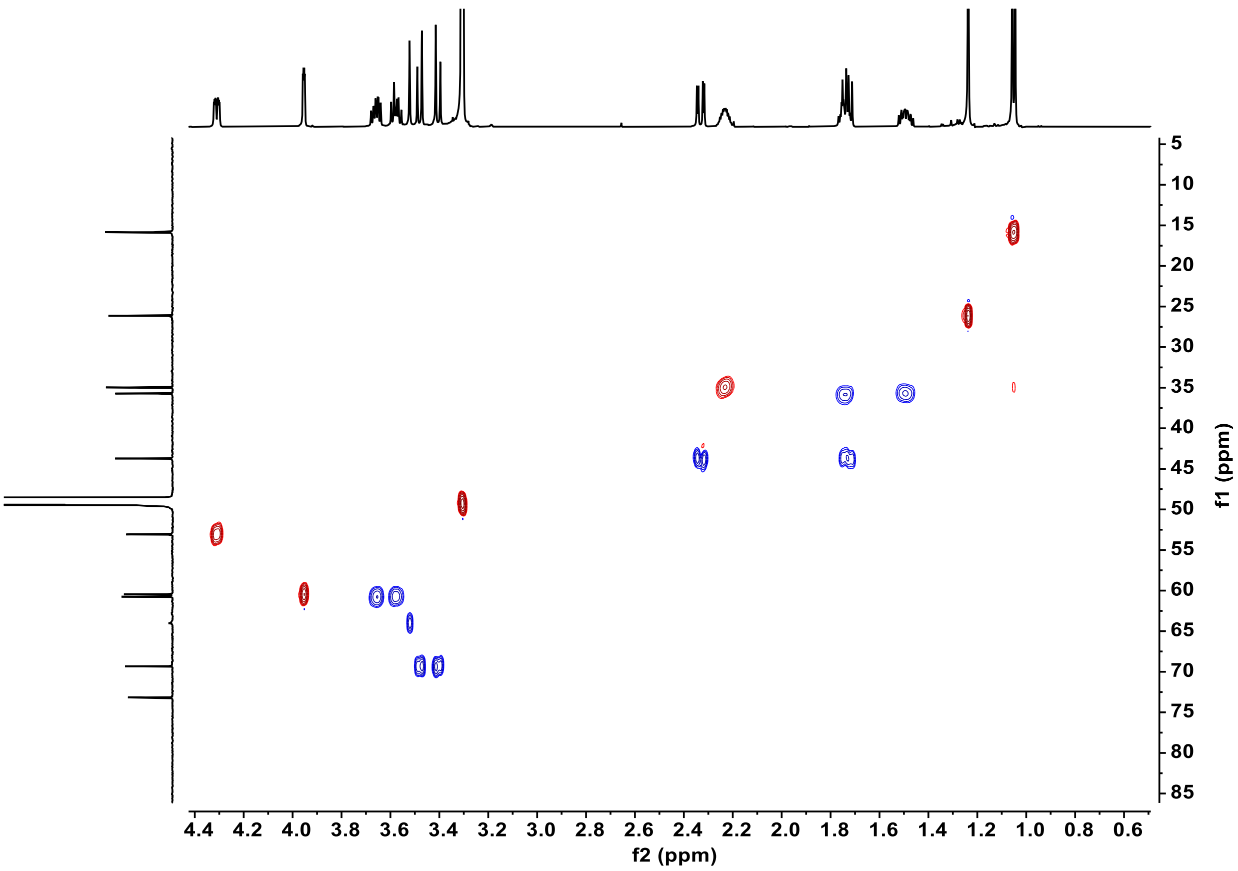

Supplementary Fig. 55. HSQC spectrum of **3a** (600 MHz, CD_3OD).

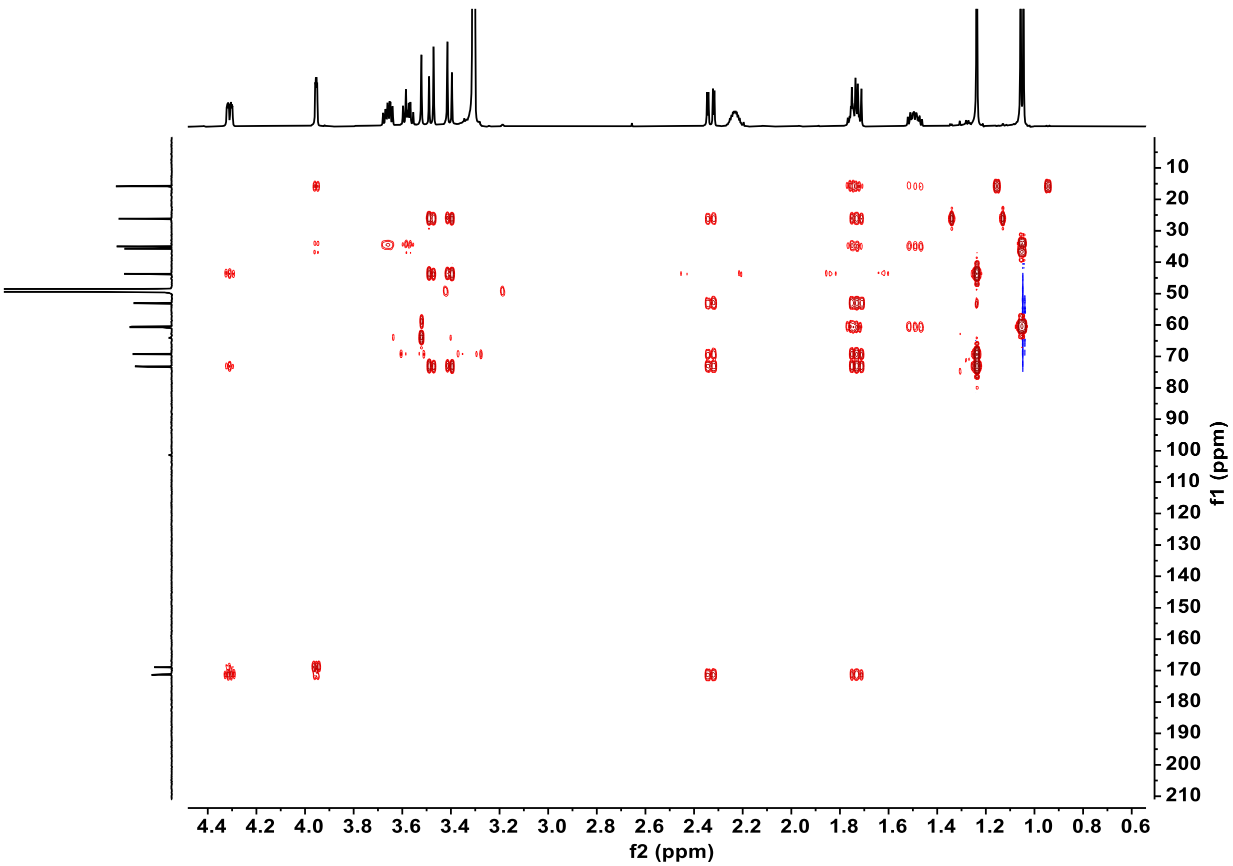

Supplementary Fig. 56. HMBC spectrum of **3a** (600 MHz, CD_3OD).

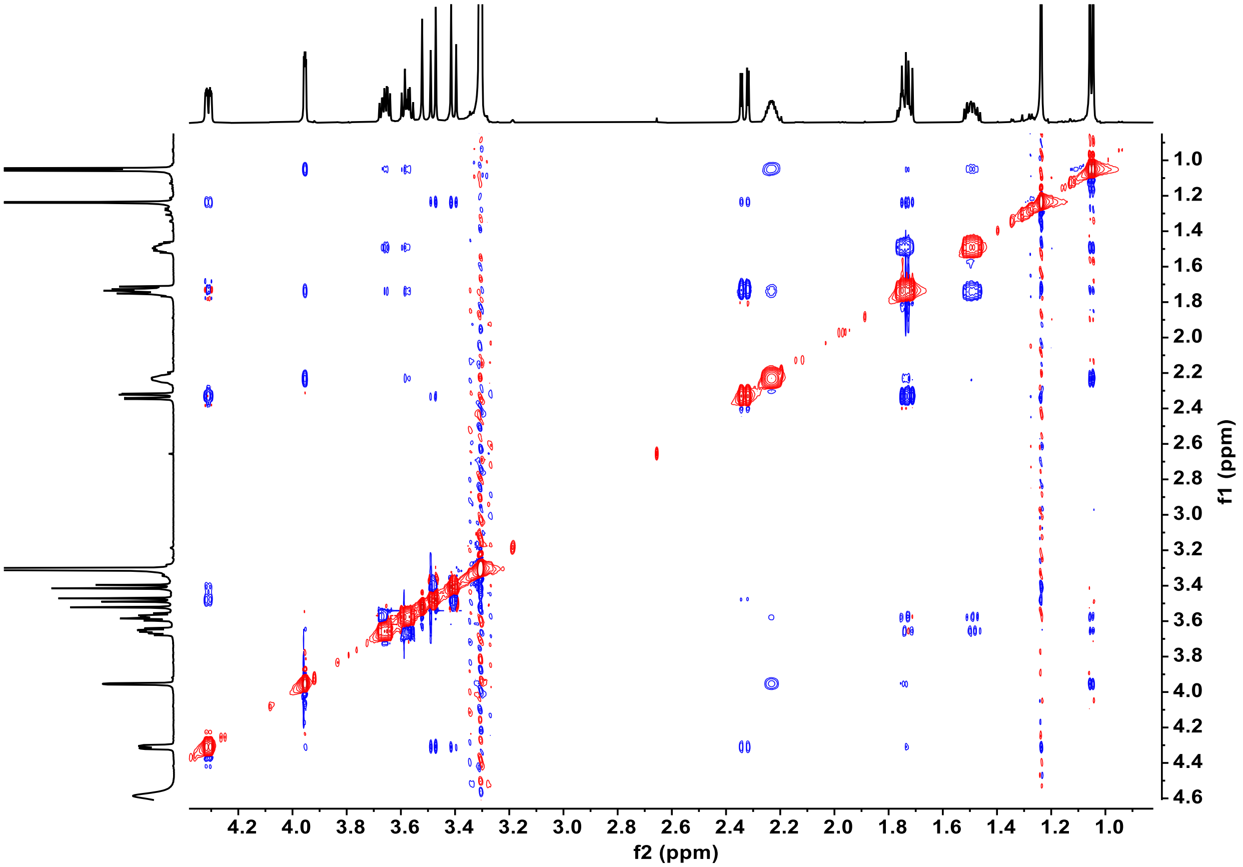

Supplementary Fig. 57. ^{1}H-^{1}H NOESY spectrum of **3a** (600 MHz, CD_3OD).

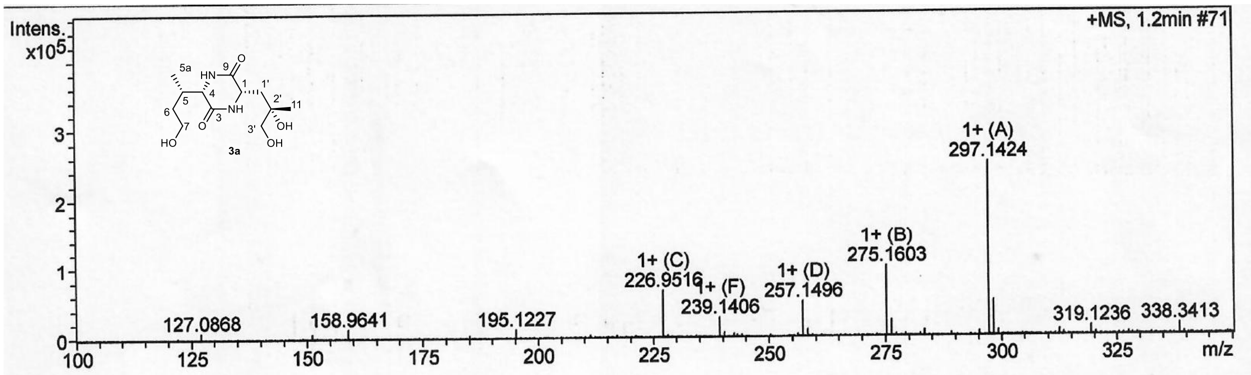

Meas. m/z	#	Formula	Score	m/z	err [ppm]	Mean err [ppm]	mSigma	rdb	e⁻ Conf	N-Rule
275.1603	1	C 12 H 23 N 2 O 5	100.00	275.1601	-0.7	-1.6	50.1	2.5	even	ok
297.1424	1	C 12 H 22 N 2 Na O 5	100.00	297.1421	-0.9	-1.4	37.3	2.5	even	ok

Supplementary Fig. 58. HR-ESI-MS (positive) spectrum of **3a**.

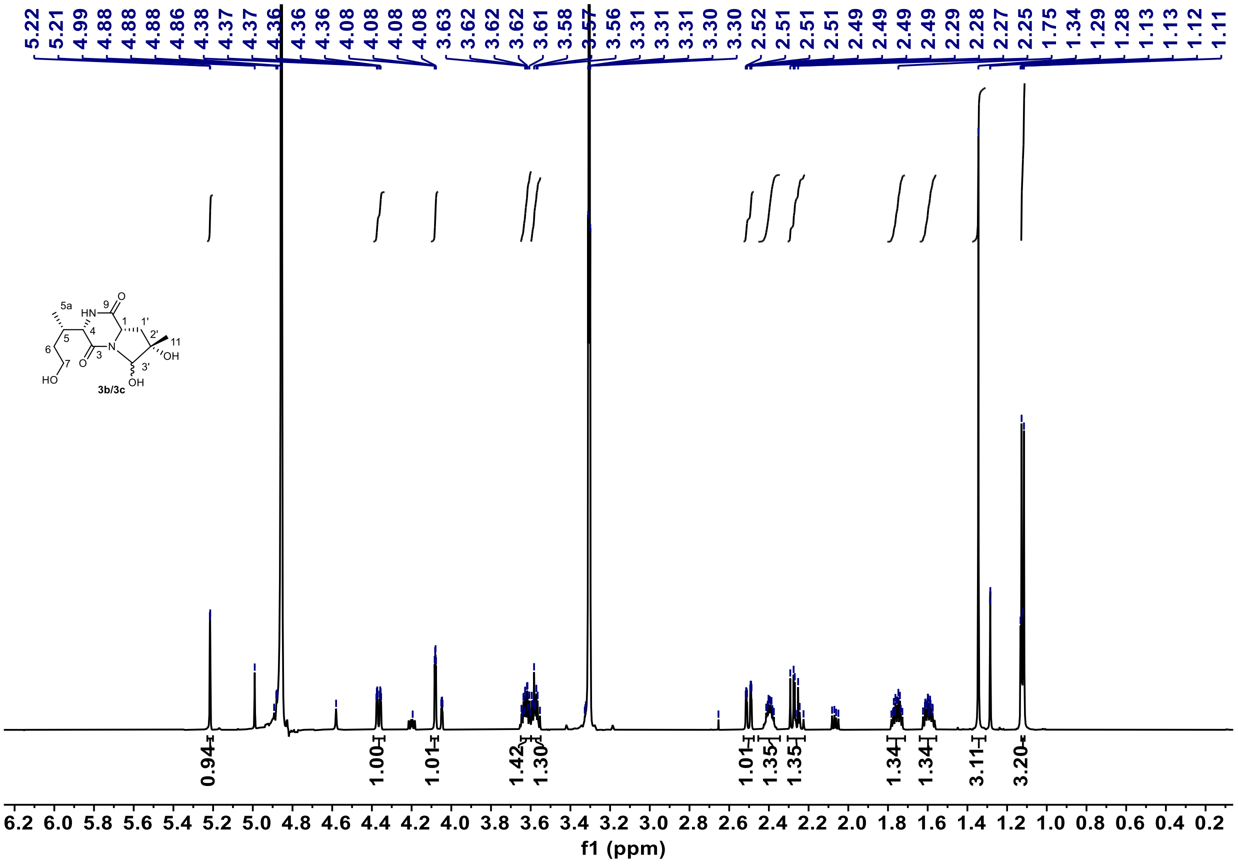

Supplementary Fig. 59. ^{1}H NMR spectrum of **3b/3c** (600 MHz, CD_3OD).

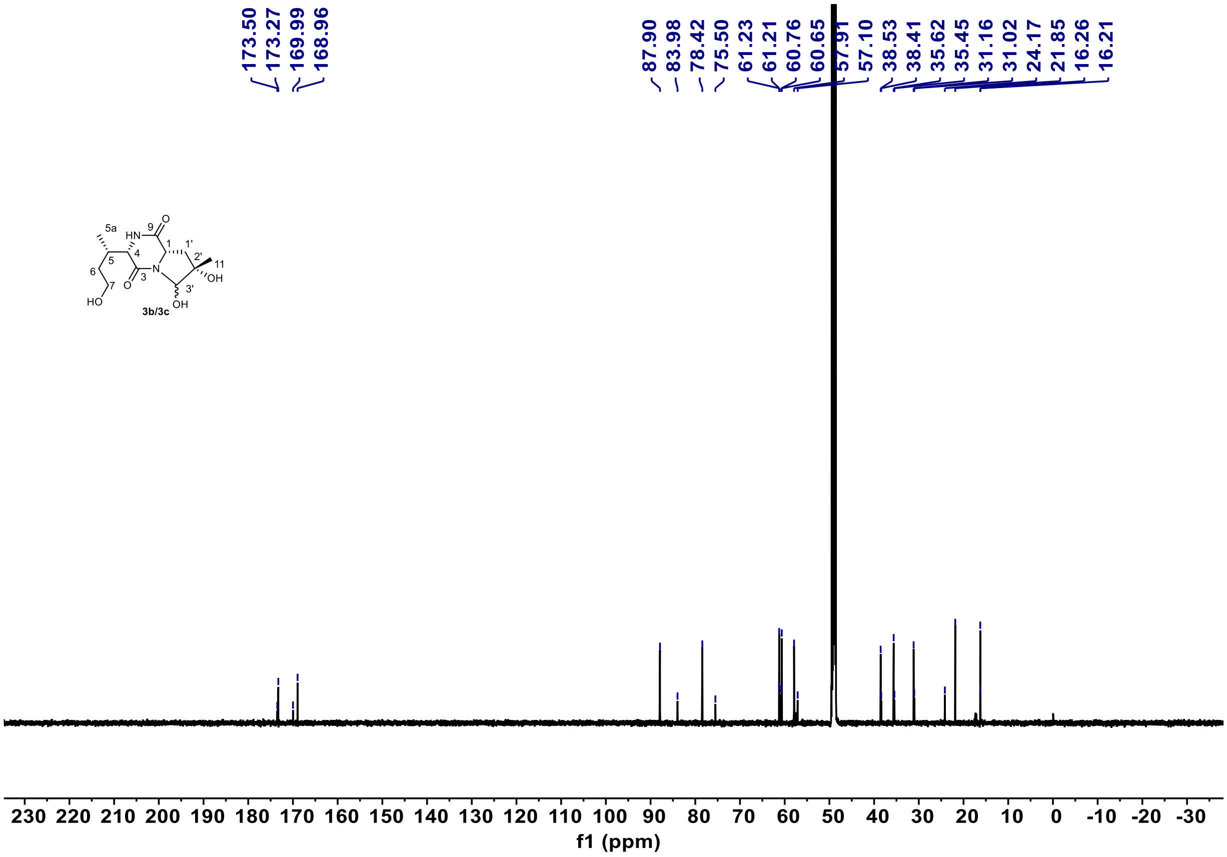

Supplementary Fig. 60. ^{13}C NMR spectrum of **3b/3c** (150 MHz, CD_3OD).

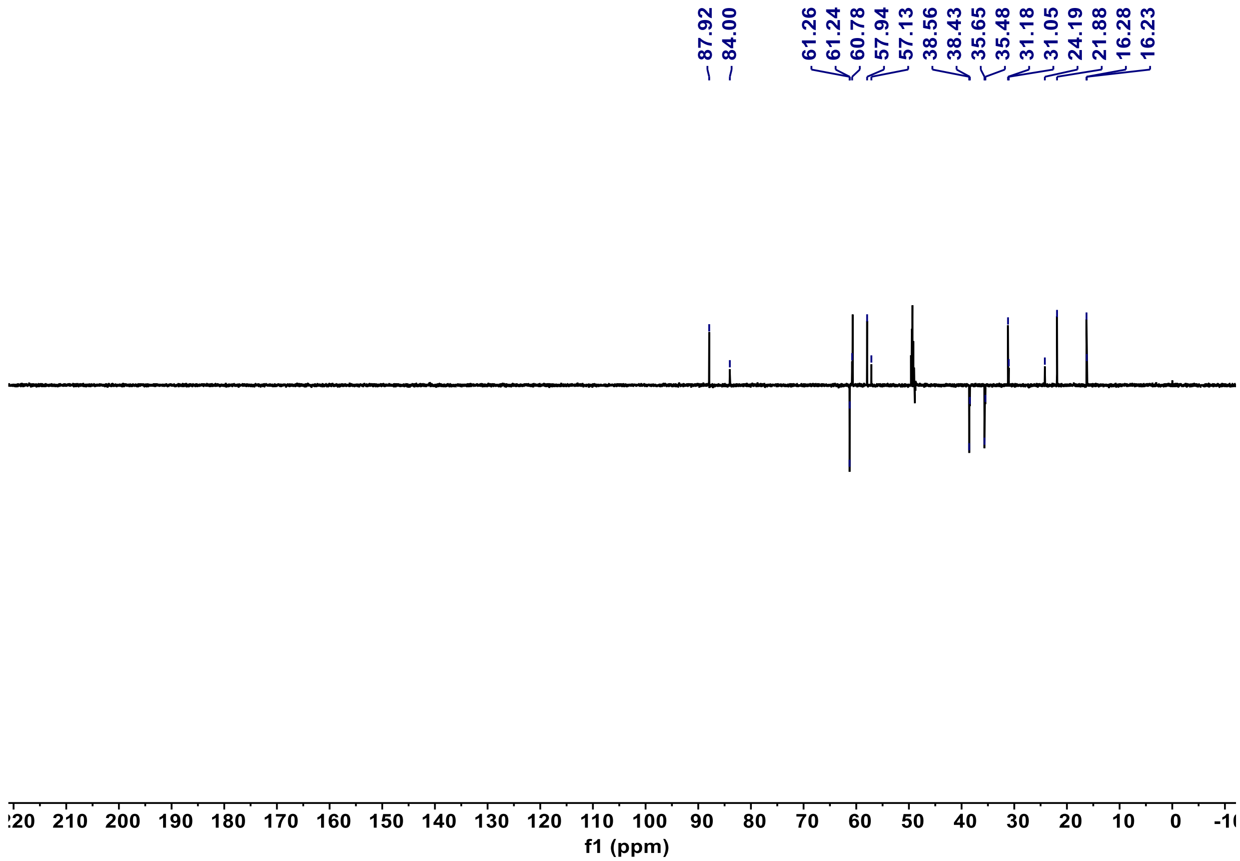

Supplementary Fig. 61. DEPT135 spectrum of **3b/3c** (150 MHz, CD_3OD).

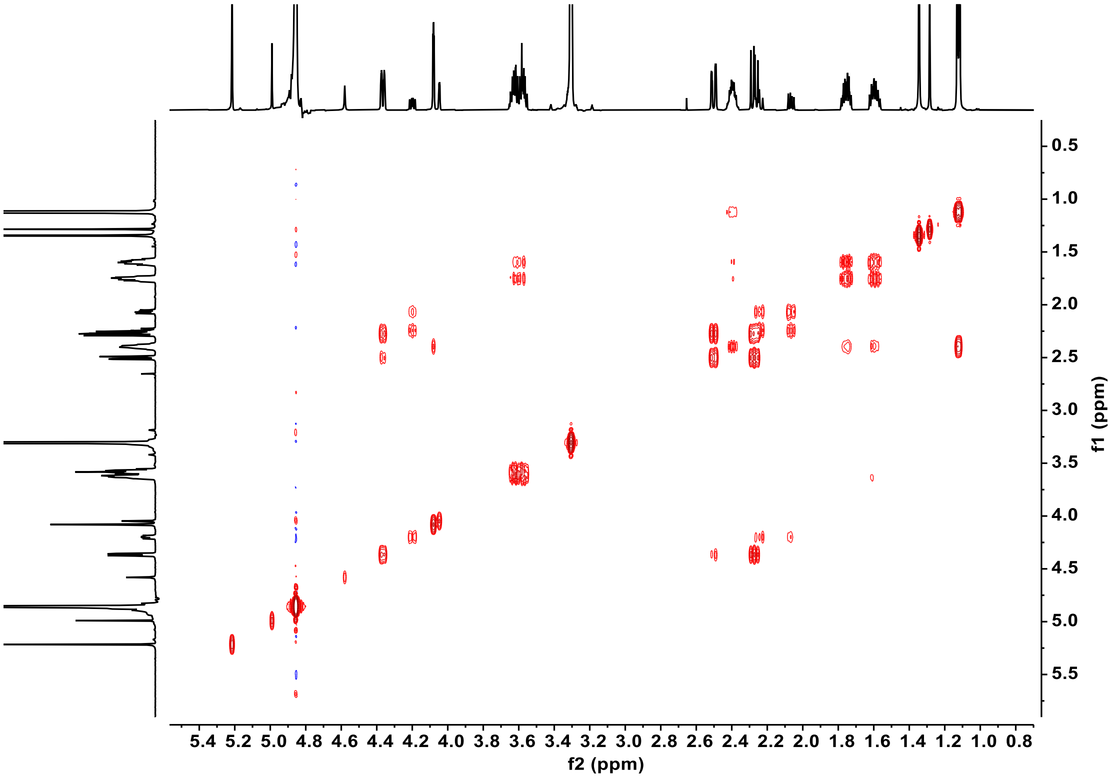

Supplementary Fig. 62. ^1H-^1H COSY spectrum of **3b/3c** (600 MHz, CD_3OD).

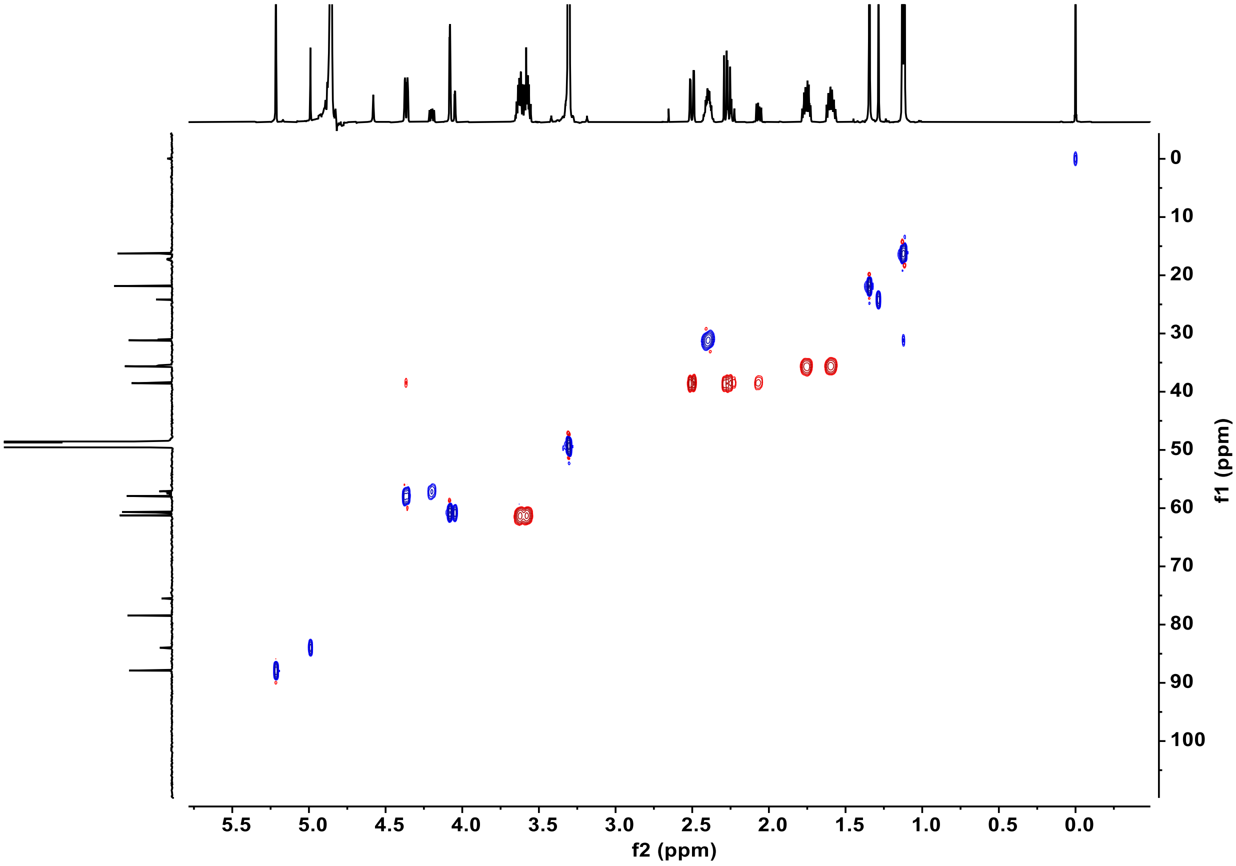

Supplementary Fig. 63. HSQC spectrum of **3b/3c** (600 MHz, CD_3OD).

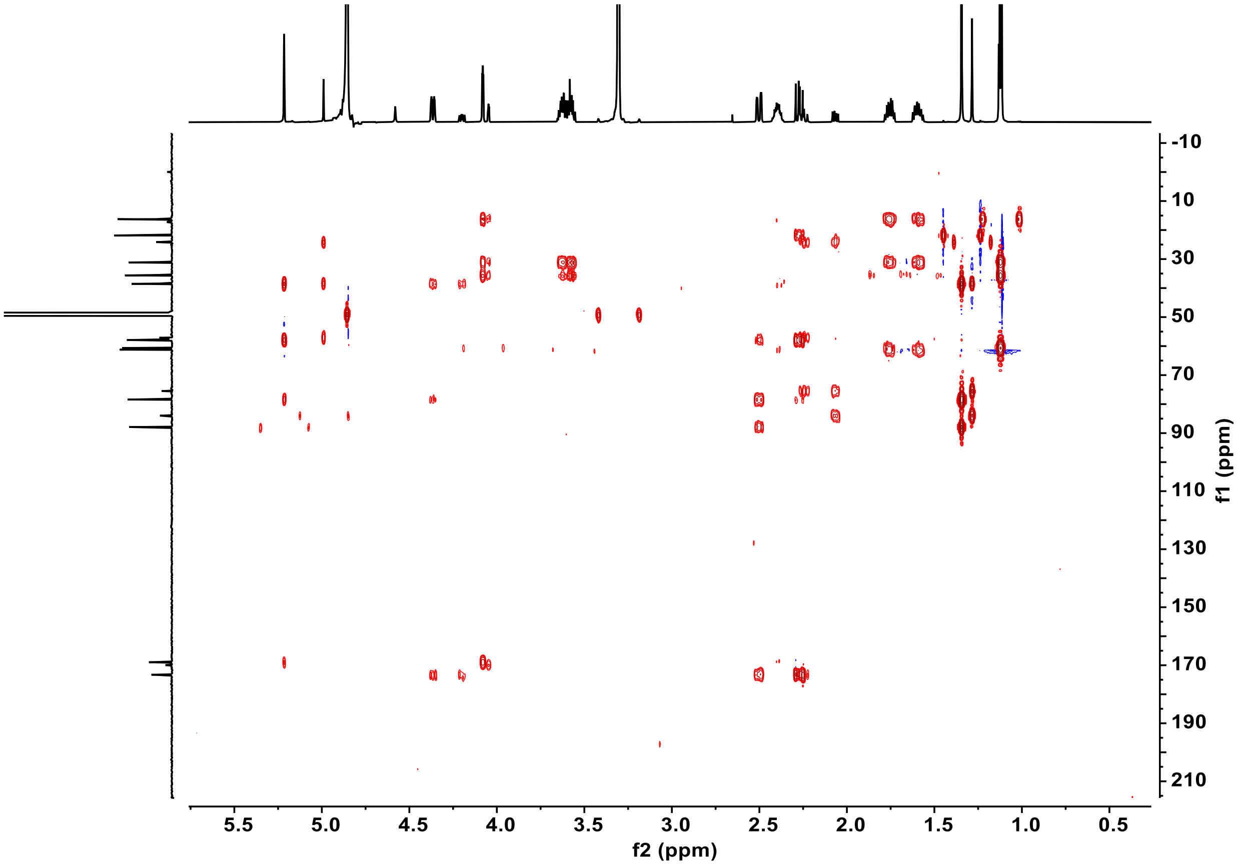

Supplementary Fig. 64. HMBC spectrum of **3b/3c** (600 MHz, CD_3OD).

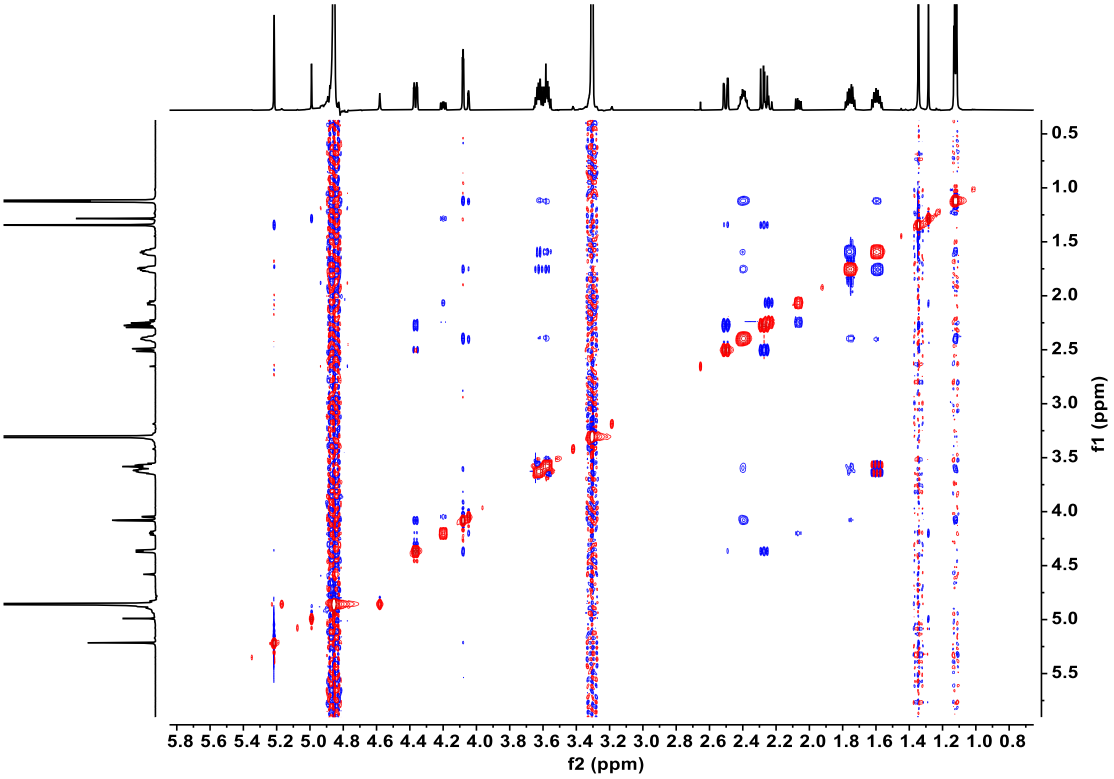

Supplementary Fig. 65. ^{1}H-^{1}H NOESY spectrum of **3b/3c** (600 MHz, CD_3OD).

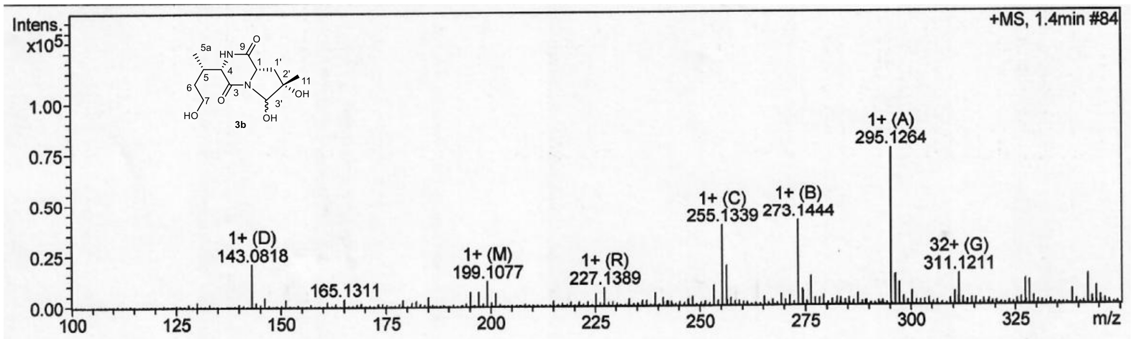

Meas. m/z	#	Formula	Score	m/z	err [ppm]	Mean err [ppm]	mSigma	rdb	e⁻ Conf	N-Rule
273.1444	1	C 12 H 21 N 2 O 5	100.00	273.1445	0.4	-0.3	32.5	3.5	even	ok
295.1264	1	C 12 H 20 N 2 Na O 5	100.00	295.1264	0.1	-0.2	31.2	3.5	even	ok

Supplementary Fig. 66. HR-ESI-MS (positive) spectrum of **3b**.

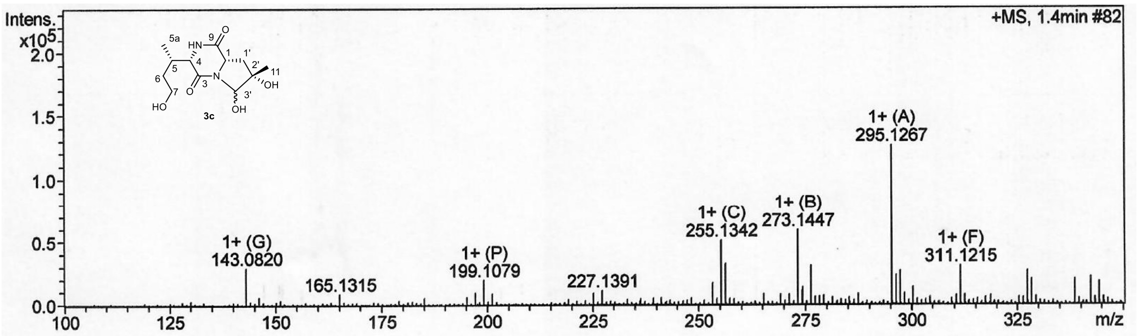

Meas. m/z	#	Formula	Score	m/z	err [ppm]	Mean err [ppm]	mSigma	rdb	e⁻ Conf	N-Rule
273.1447	1	C 12 H 21 N 2 O 5	100.00	273.1445	-0.7	-1.0	35.0	3.5	even	ok
295.1267	1	C 12 H 20 N 2 Na O 5	100.00	295.1264	-1.0	-1.1	29.2	3.5	even	ok

Supplementary Fig. 67. HR-ESI-MS (positive) spectrum of **3c**.

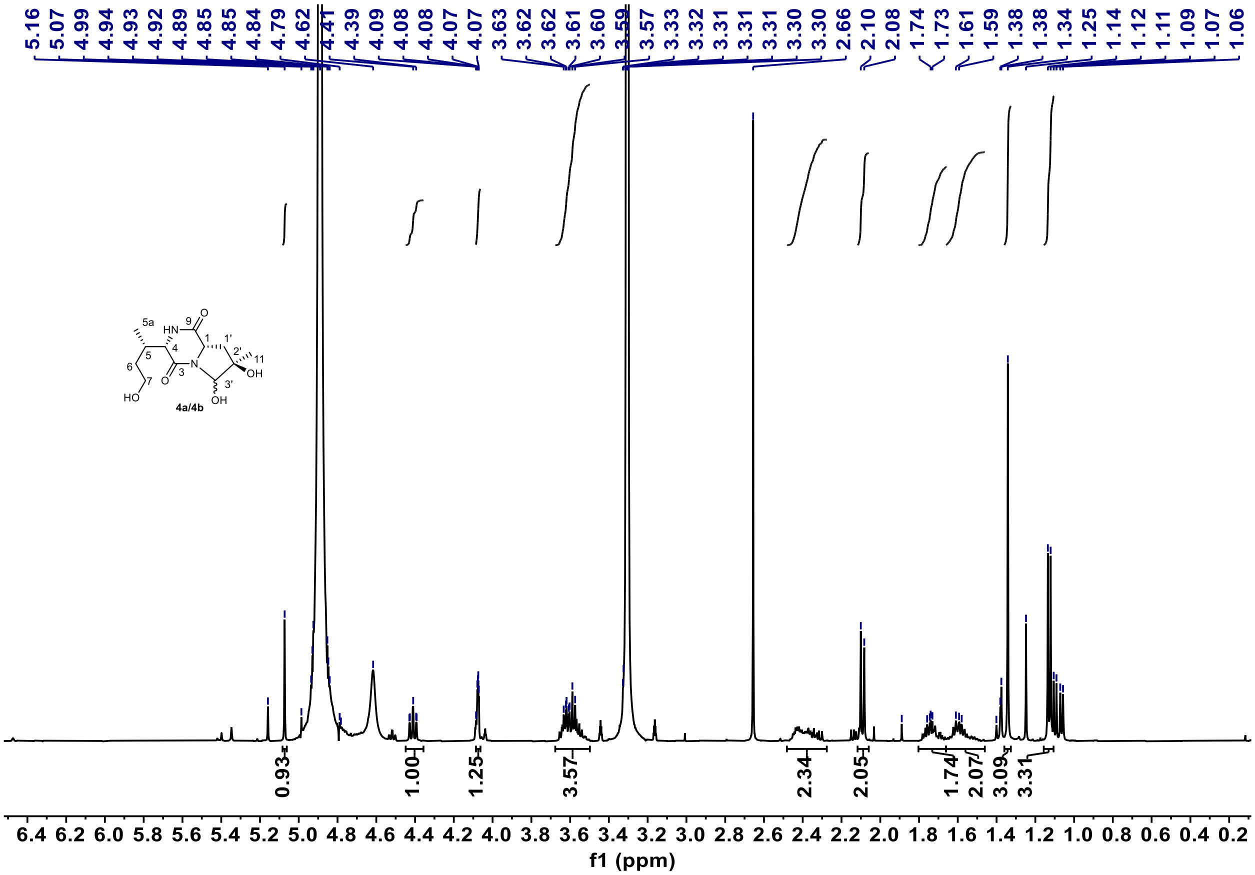

Supplementary Fig. 68. ^{1}H NMR spectrum of **4a/4b** (600 MHz, CD_3OD).

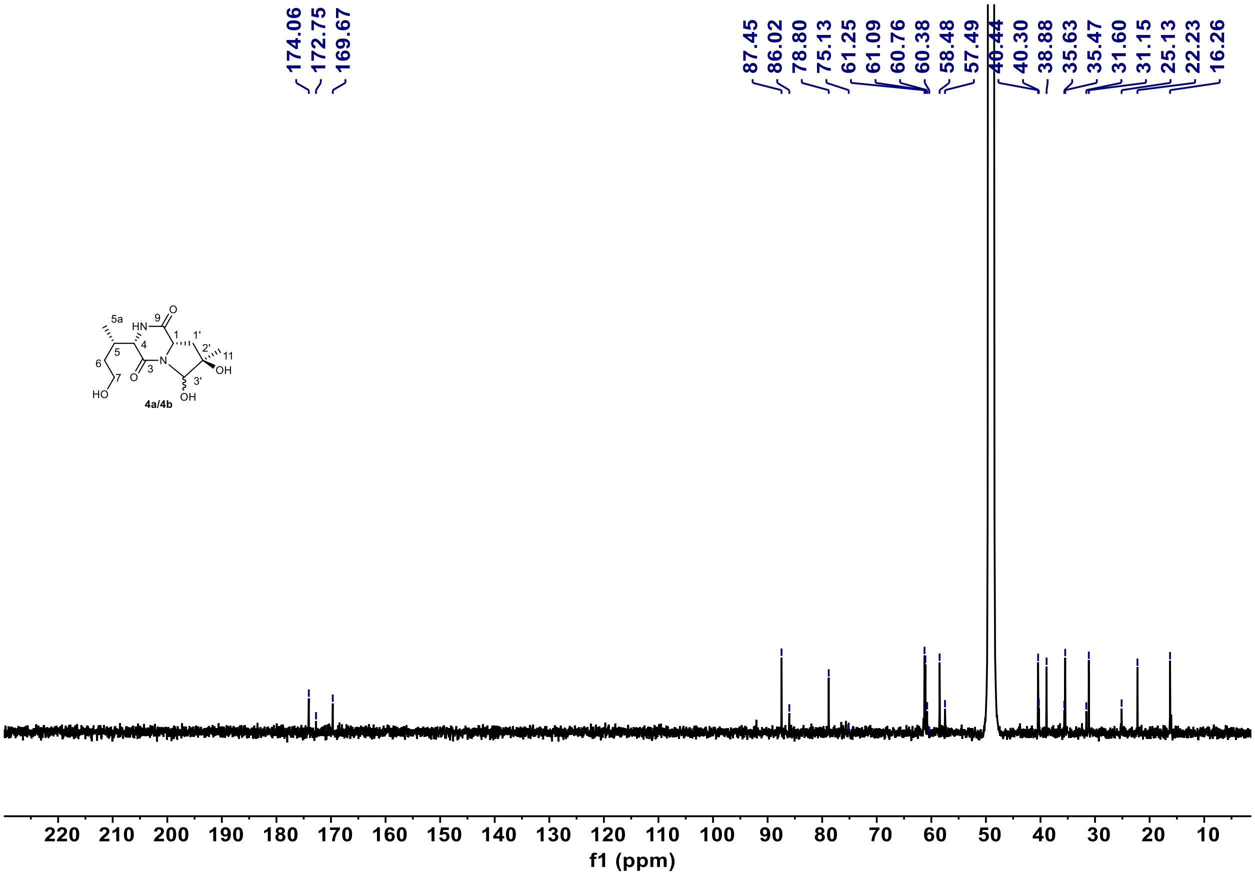

Supplementary Fig. 69. ^{13}C NMR spectrum of **4a/4b** (150 MHz, CD_3OD).

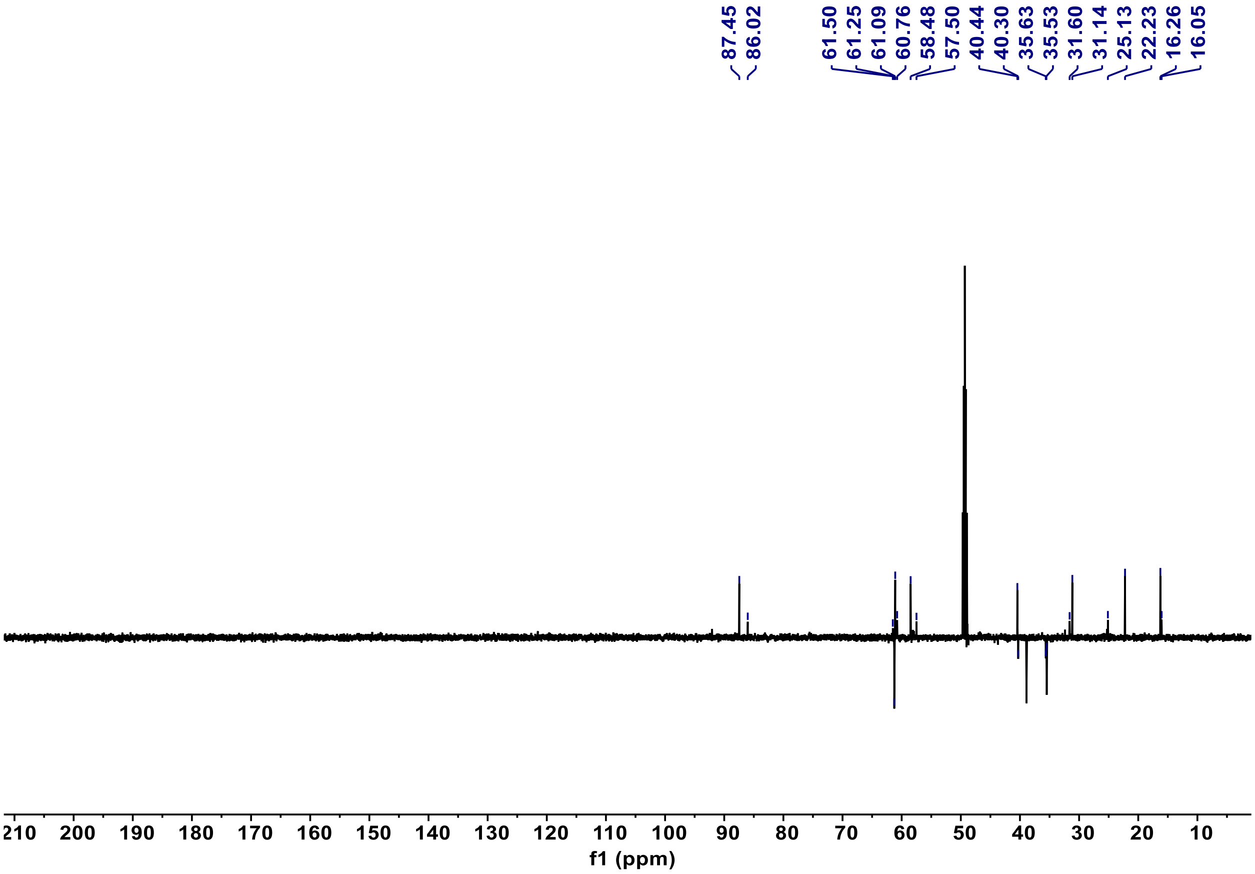

Supplementary Fig. 70. DEPT135 spectrum of **4a/4b** (150 MHz, CD_3OD).

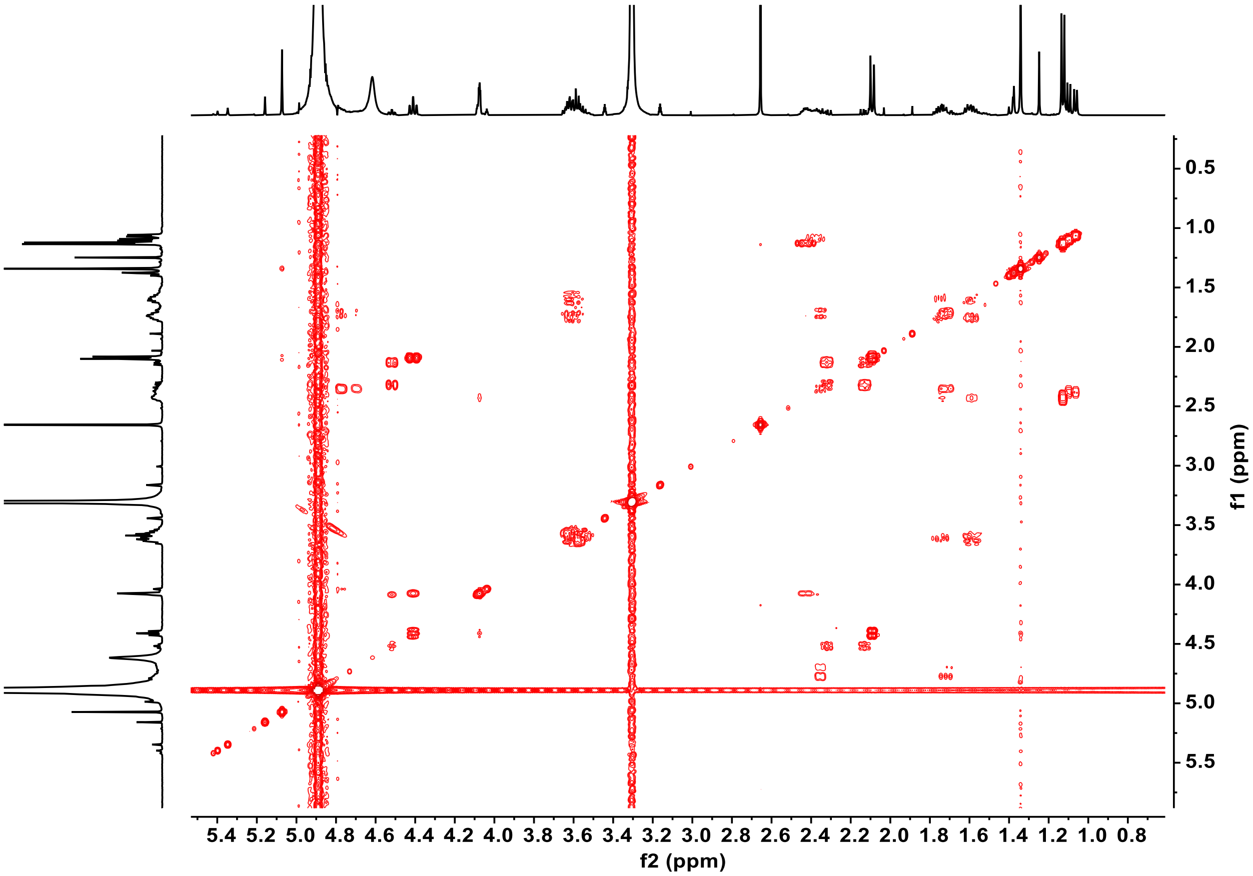

Supplementary Fig. 71. ^1H-^1H COSY spectrum of **4a/4b** (600 MHz, CD_3OD).

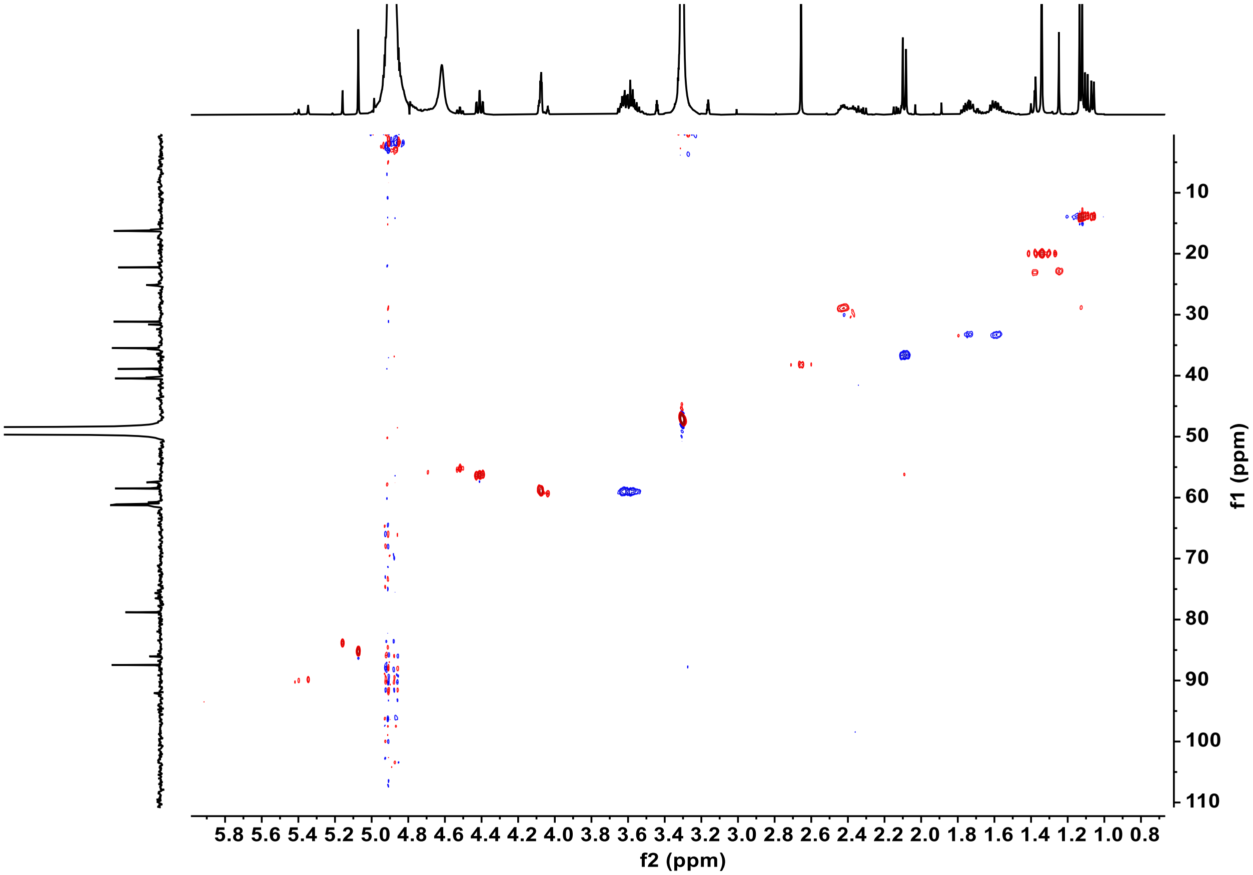

Supplementary Fig. 72. HSQC spectrum of **4a/4b** (600 MHz, CD_3OD).

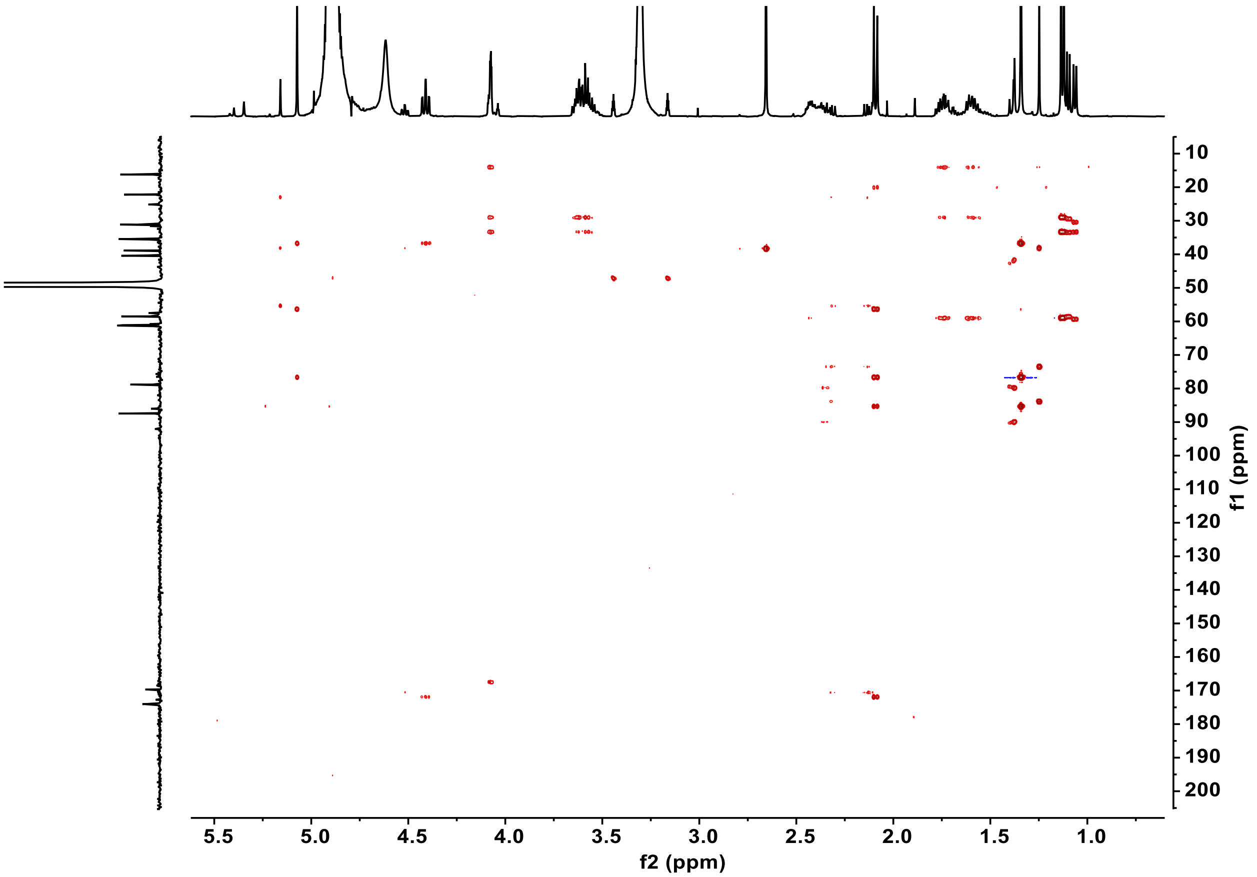

Supplementary Fig. 73. HMBC spectrum of **4a/4b** (600 MHz, CD_3OD).

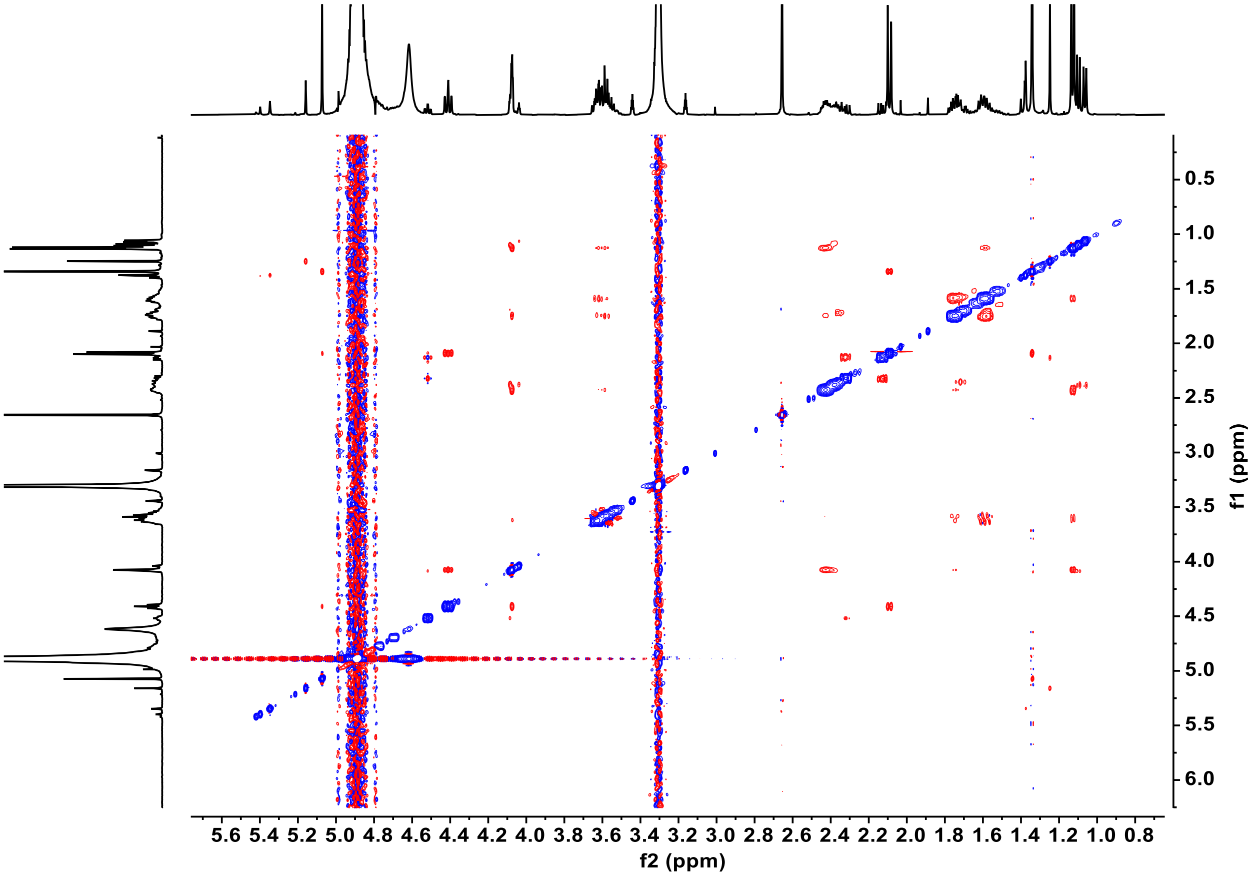

Supplementary Fig. 74. ^{1}H-^{1}H NOESY spectrum of **4a/4b** (600 MHz, CD_3OD).

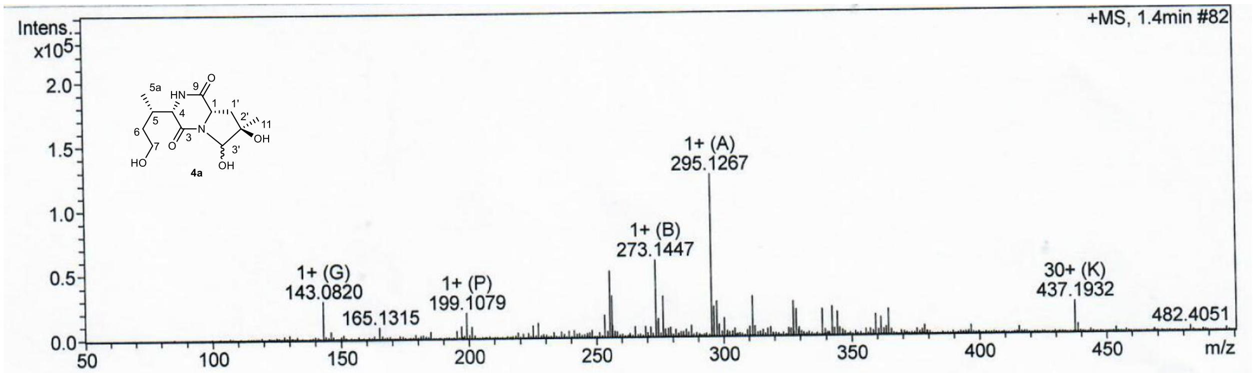

Meas. m/z	#	Formula	Score	m/z	err [ppm]	Mean err [ppm]	mSigma	rdb	e⁻ Conf	N-Rule
273.1447	1	C 12 H 21 N 2 O 5	100.00	273.1445	-0.7	-1.0	35.0	3.5	even	ok
295.1267	1	C 12 H 20 N 2 Na O 5	100.00	295.1264	-1.0	-1.1	29.2	3.5	even	ok

Supplementary Fig. 75. HR-ESI-MS (positive) spectrum of **4a**.

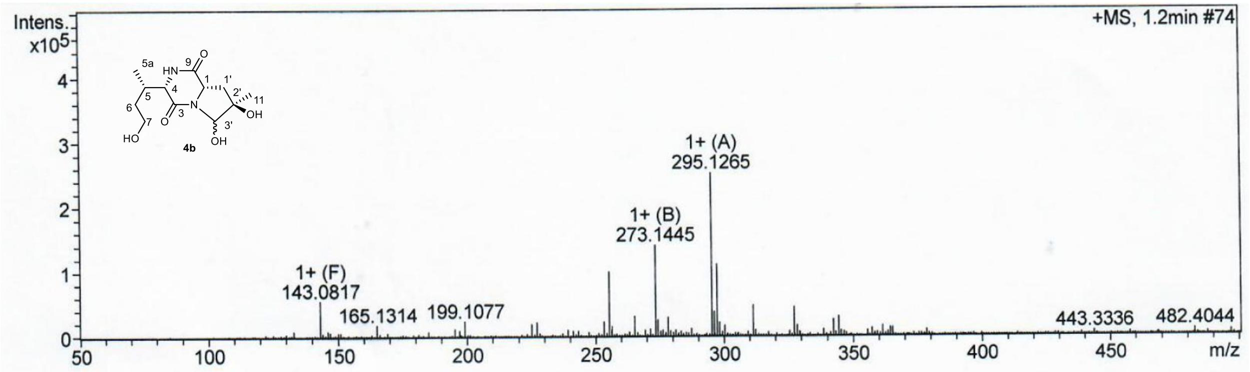

Meas. m/z	#	Formula	Score	m/z	err [ppm]	Mean err [ppm]	mSigma	rdb	e⁻ Conf	N-Rule
273.1445	1	C 12 H 21 N 2 O 5	100.00	273.1445	-0.1	-0.0	25.9	3.5	even	ok
295.1265	1	C 12 H 20 N 2 Na O 5	100.00	295.1264	-0.3	-0.3	13.0	3.5	even	ok

Supplementary Fig. 76. HR-ESI-MS (positive) spectrum of **4b**.

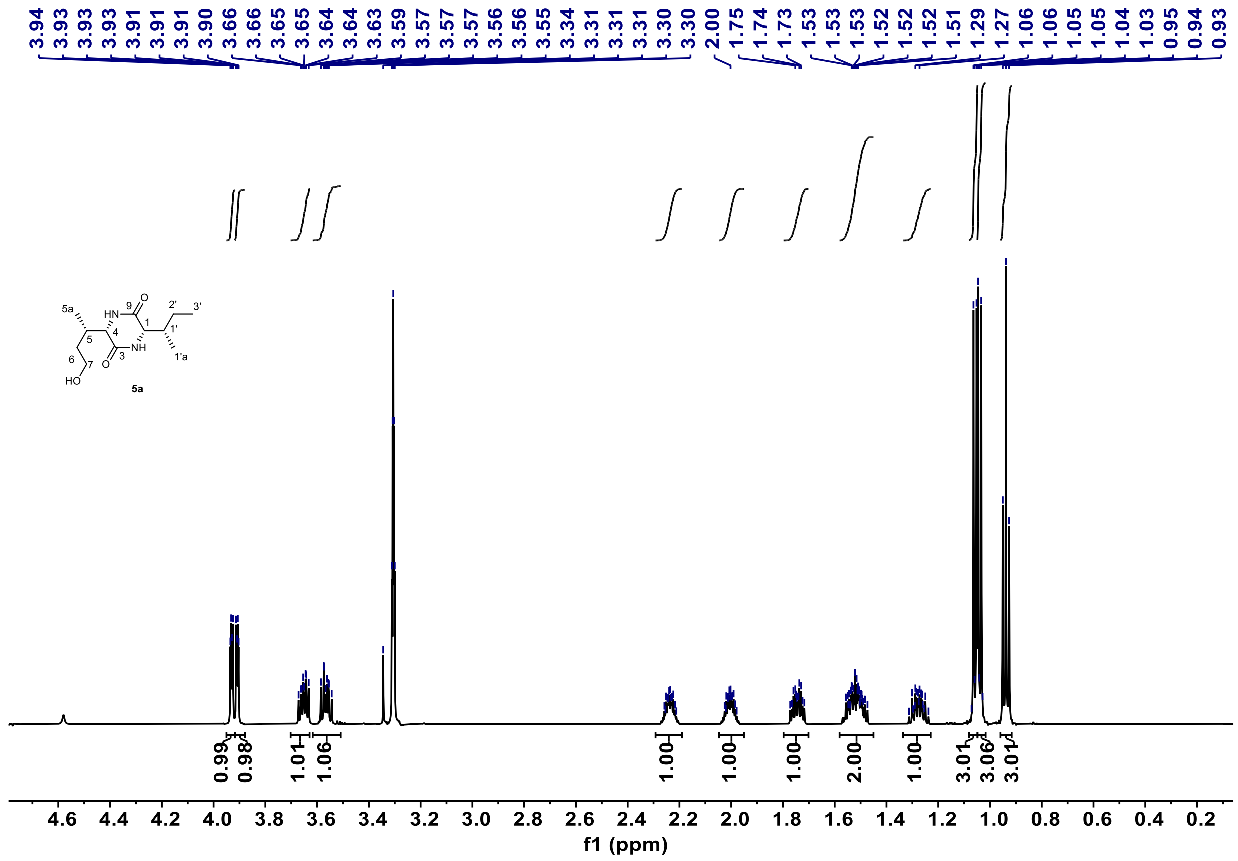

Supplementary Fig. 77. ^{1}H NMR spectrum of **5a** (600 MHz, CD_3OD).

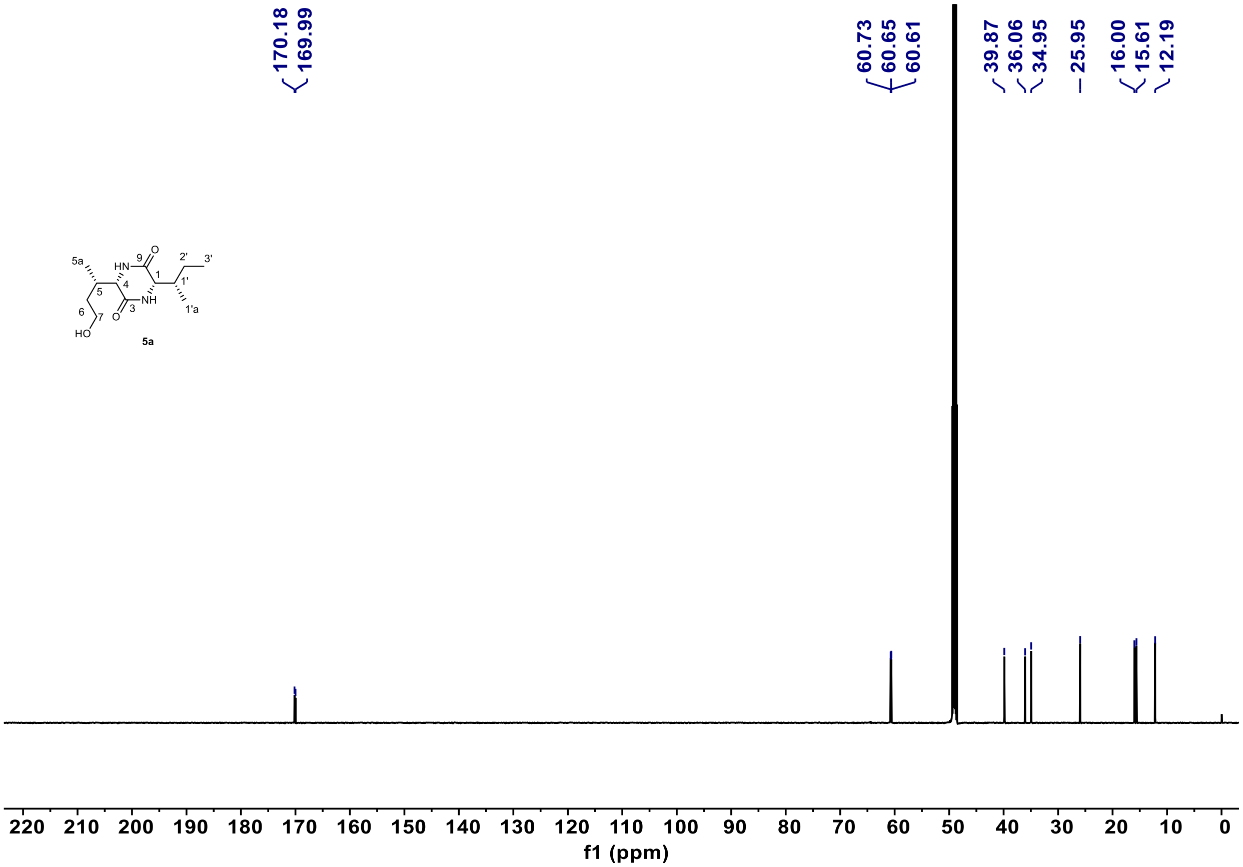

Supplementary Fig. 78. ^{13}C NMR spectrum of **5a** (150 MHz, CD_3OD).

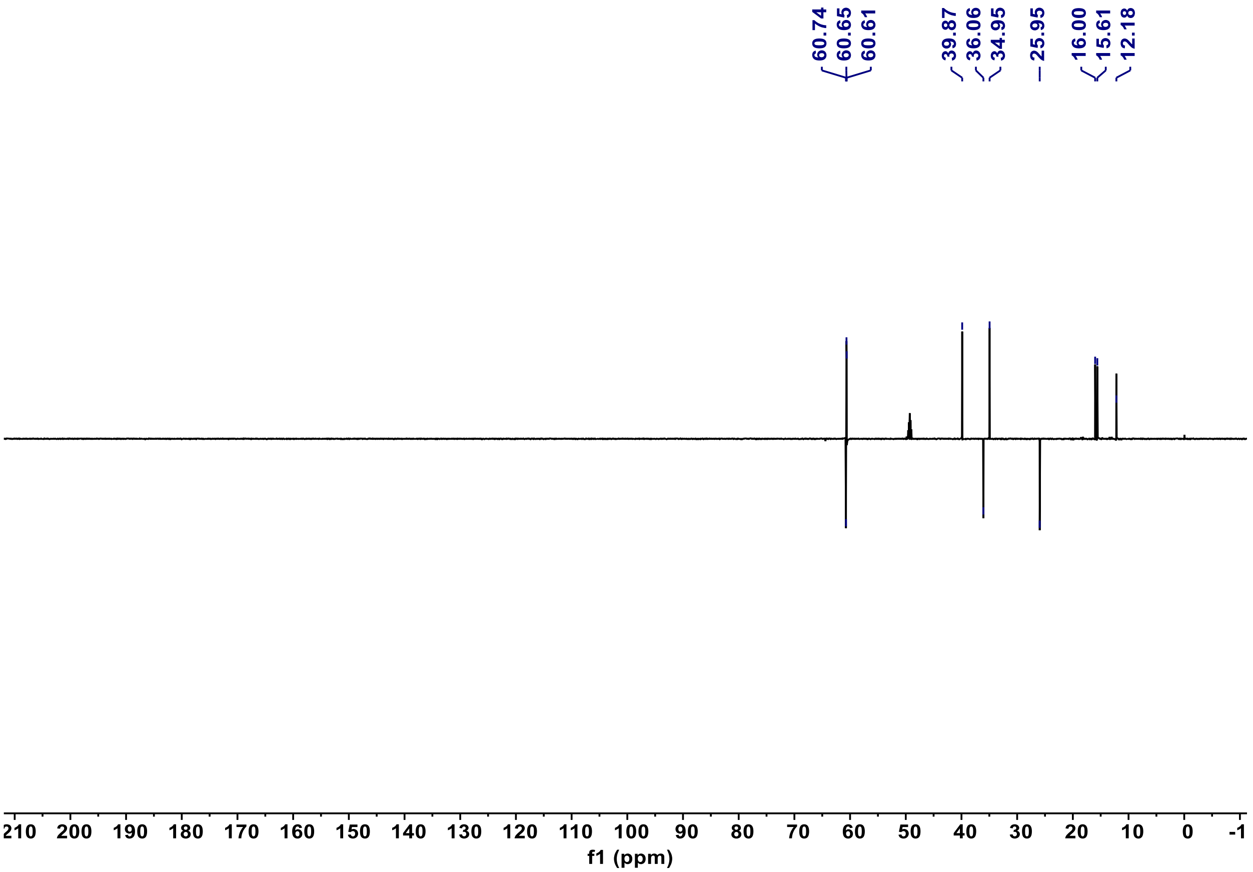

Supplementary Fig. 79. DEPT135 spectrum of **5a** (150 MHz, CD_3OD).

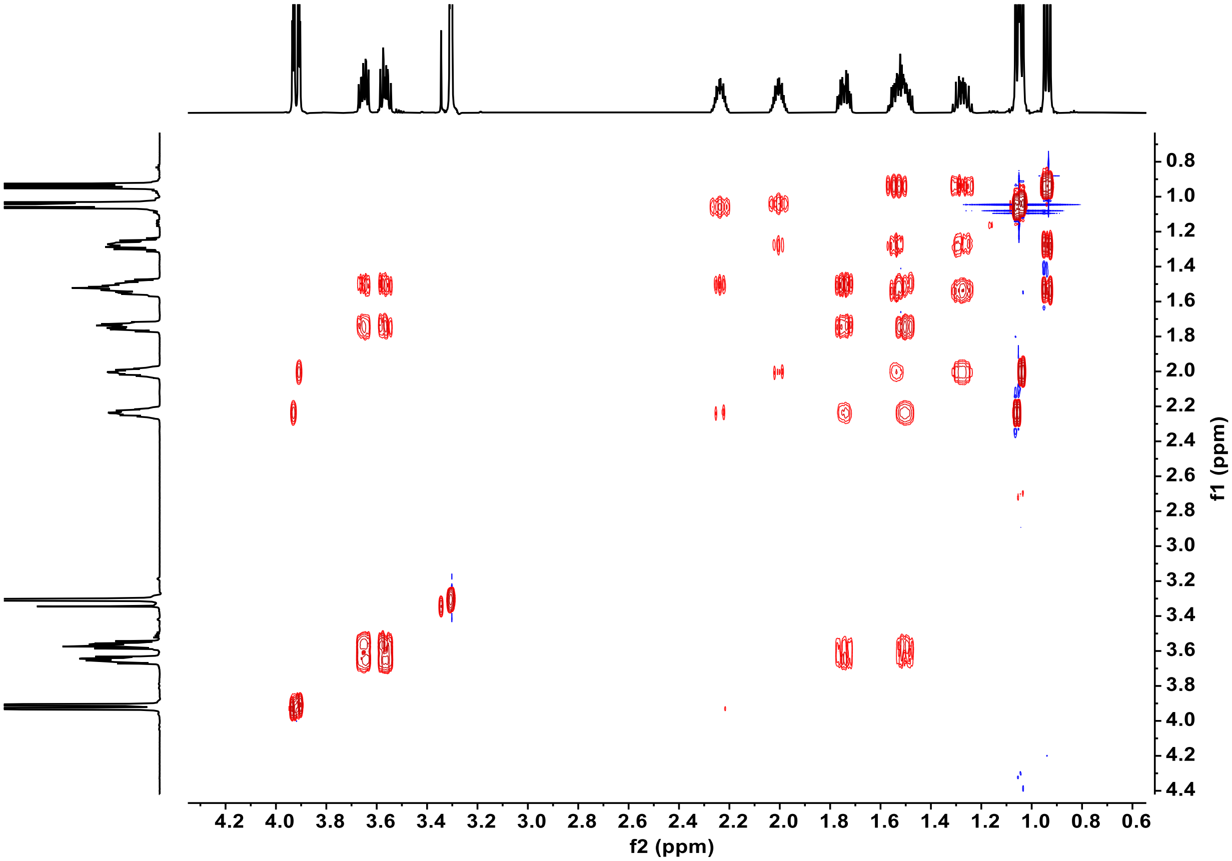

Supplementary Fig. 80. ^{1}H-^{1}H COSY spectrum of **5a** (600 MHz, CD_3OD).

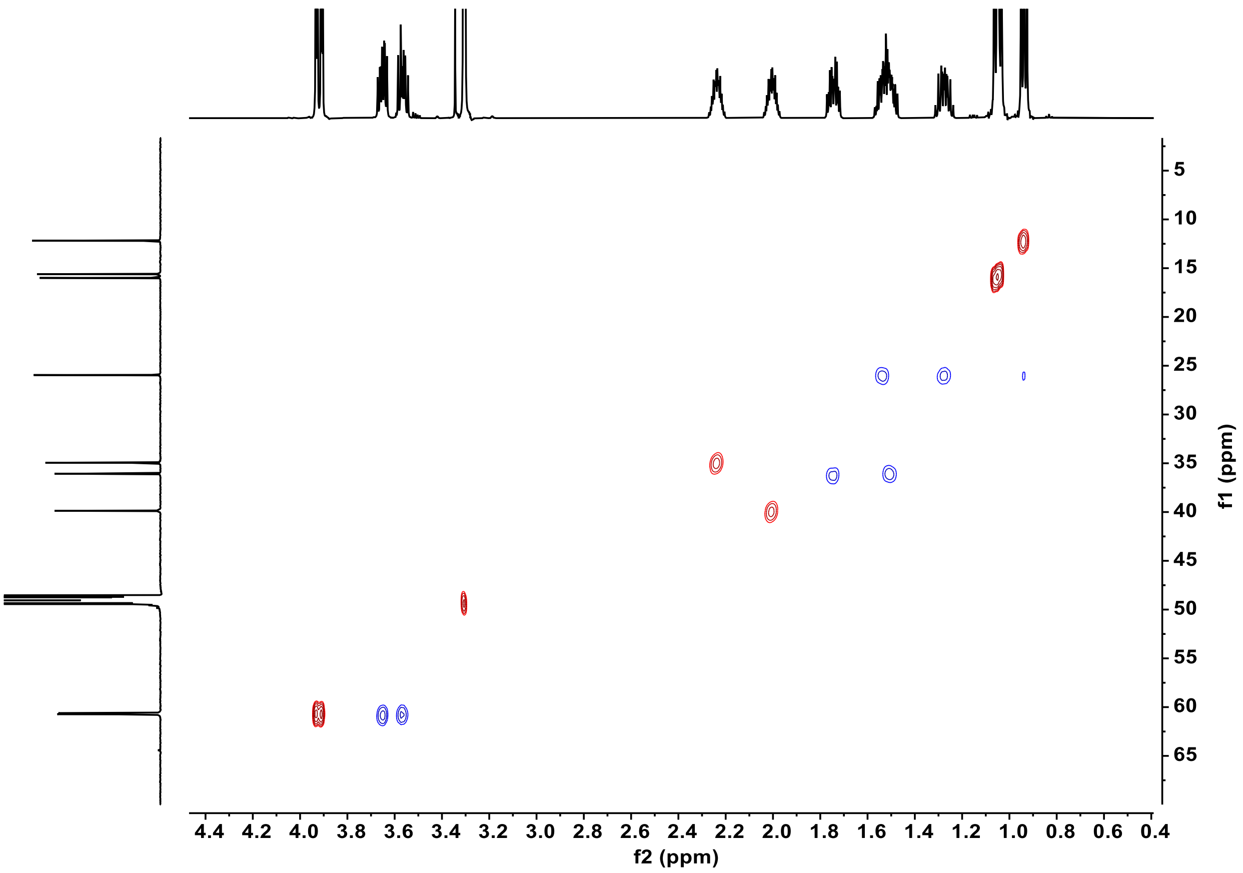

Supplementary Fig. 81. HSQC spectrum of **5a** (600 MHz, CD_3OD).

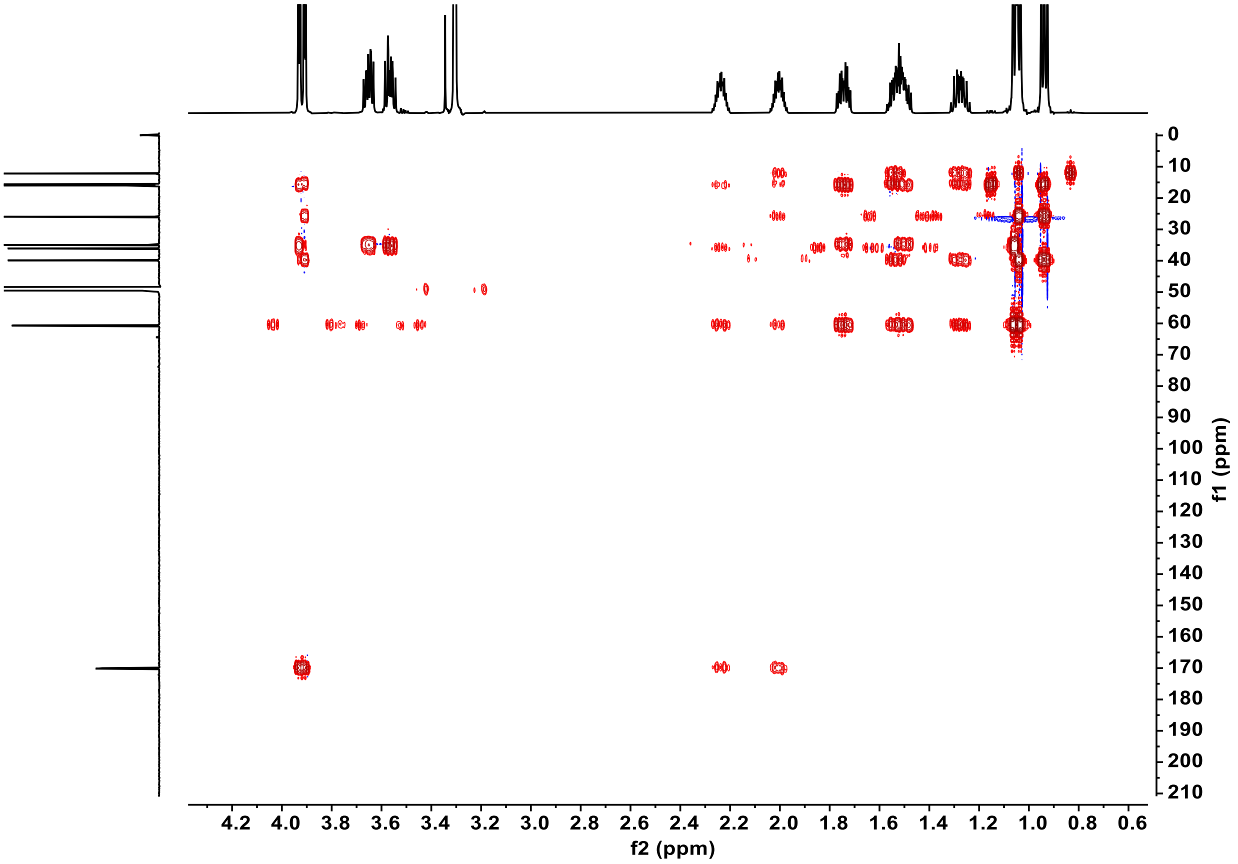

Supplementary Fig. 82. HMBC spectrum of **5a** (600 MHz, CD_3OD).

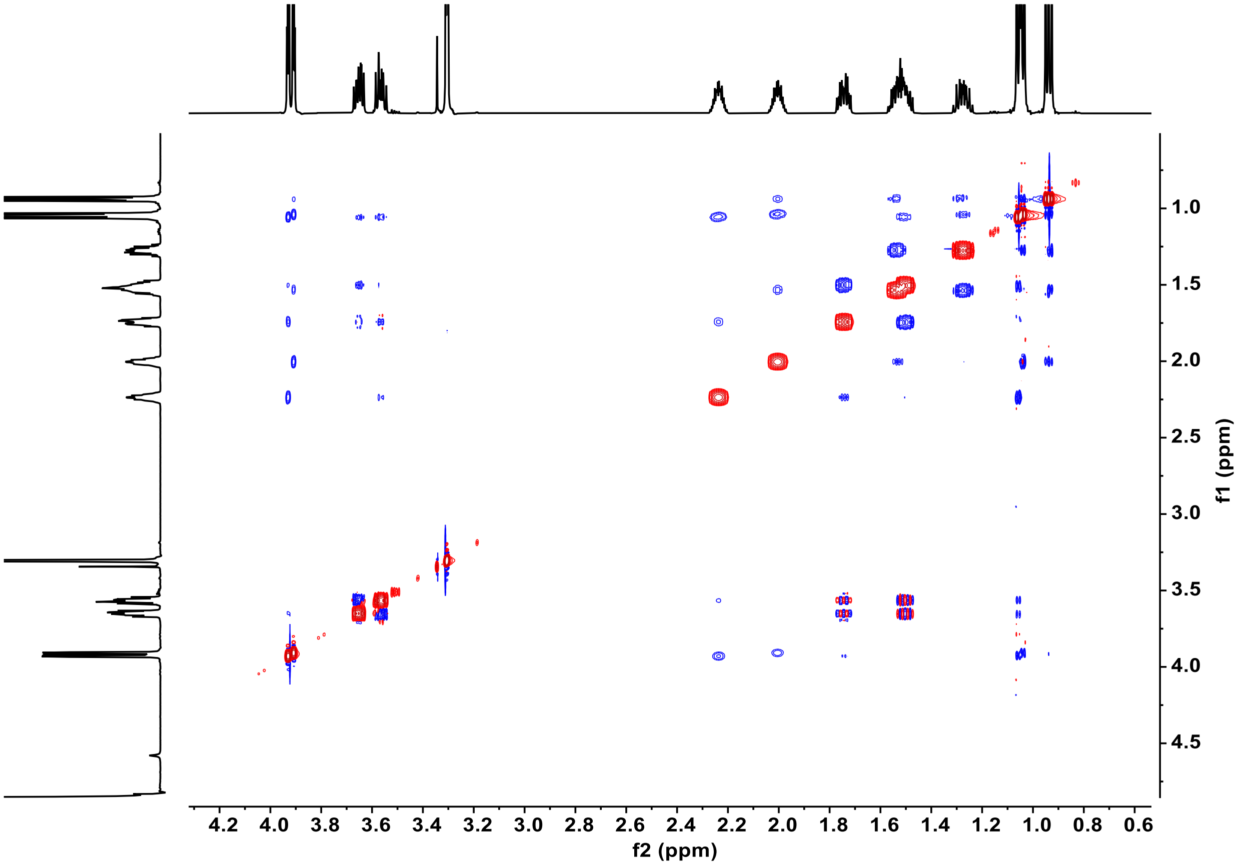

Supplementary Fig. 83. ^{1}H-^{1}H NOESY spectrum of **5a** (600 MHz, CD_3OD).

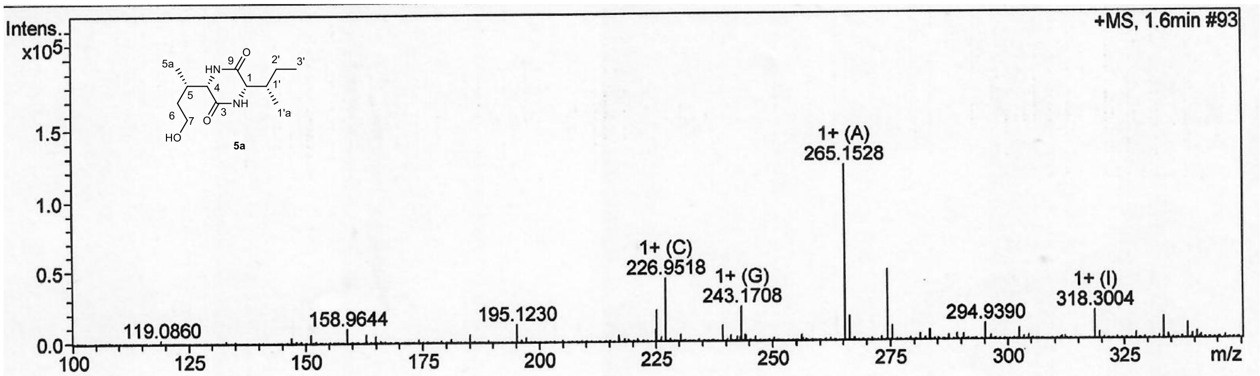

Meas. m/z	#	Formula	Score	m/z	err [ppm]	Mean err [ppm]	mSigma	rdb	e⁻ Conf	N-Rule
243.1708	1	C 12 H 23 N 2 O 3	100.00	243.1703	-2.0	-1.8	2.2	2.5	even	ok
265.1528	1	C 12 H 22 N 2 Na O 3	100.00	265.1523	-2.1	-1.9	1.4	2.5	even	ok

Supplementary Fig. 84. HR-ESI-MS (positive) spectrum of **5a**.

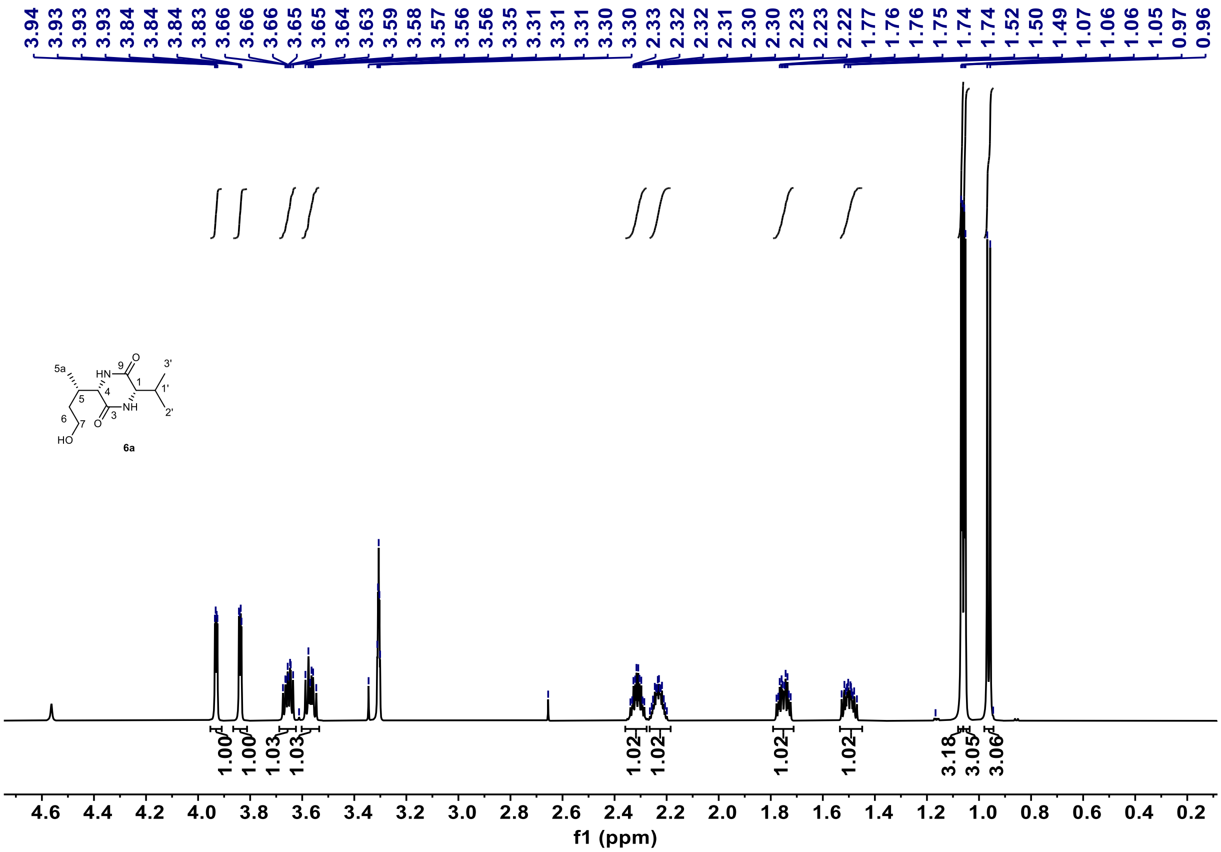

Supplementary Fig. 85. ^{1}H NMR spectrum of **6a** (600 MHz, CD_3OD).

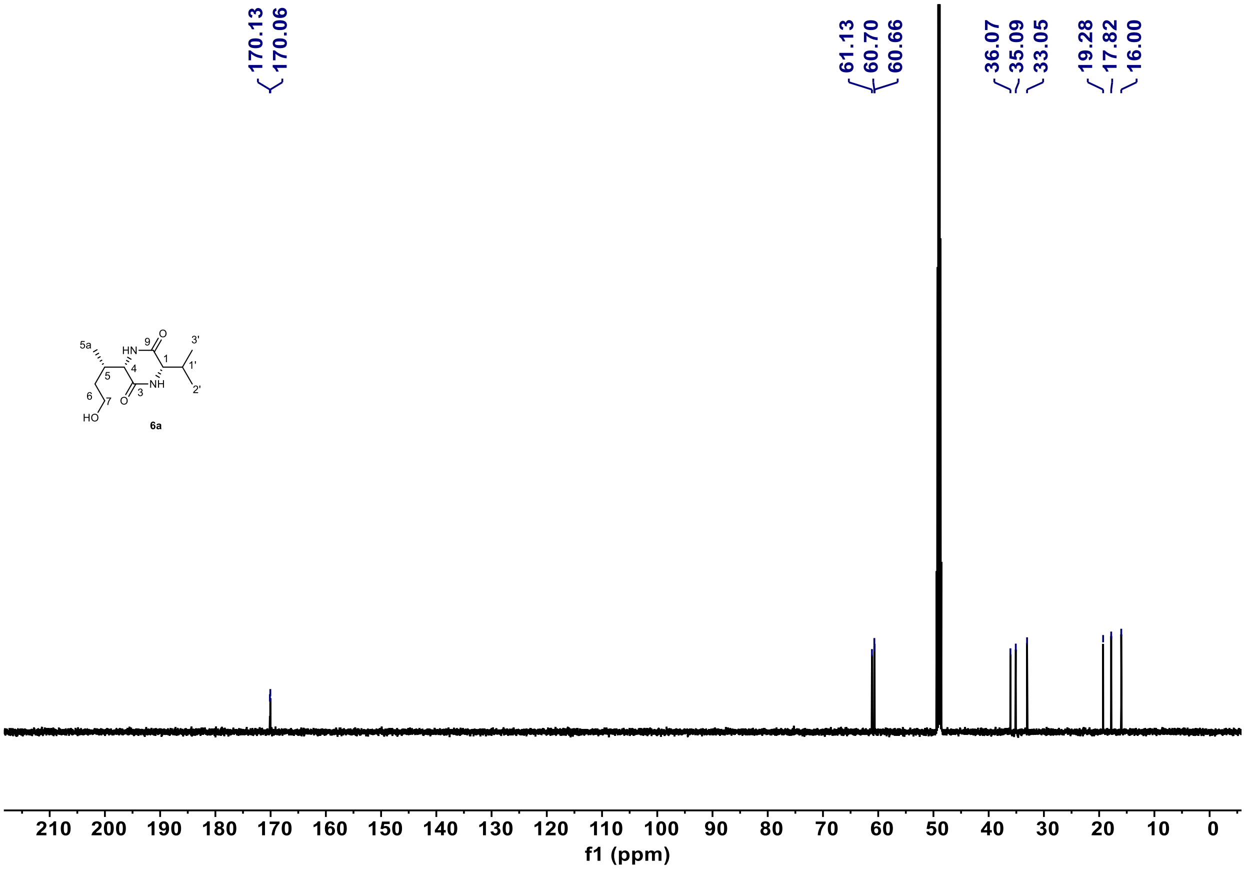

Supplementary Fig. 86. ^{13}C NMR spectrum of **6a** (150 MHz CD_3OD).

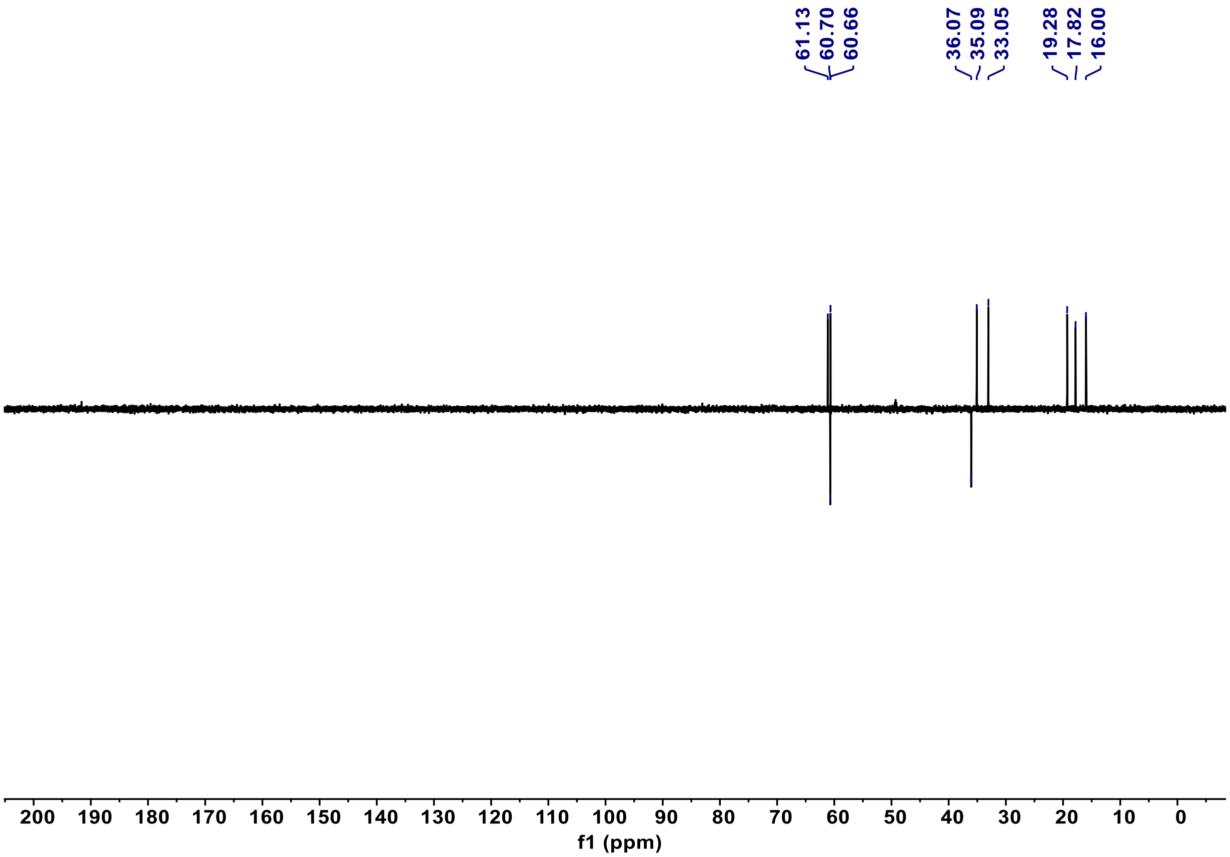

Supplementary Fig. 87. DEPT135 spectrum of **6a** (150 MHz, CD_3OD).

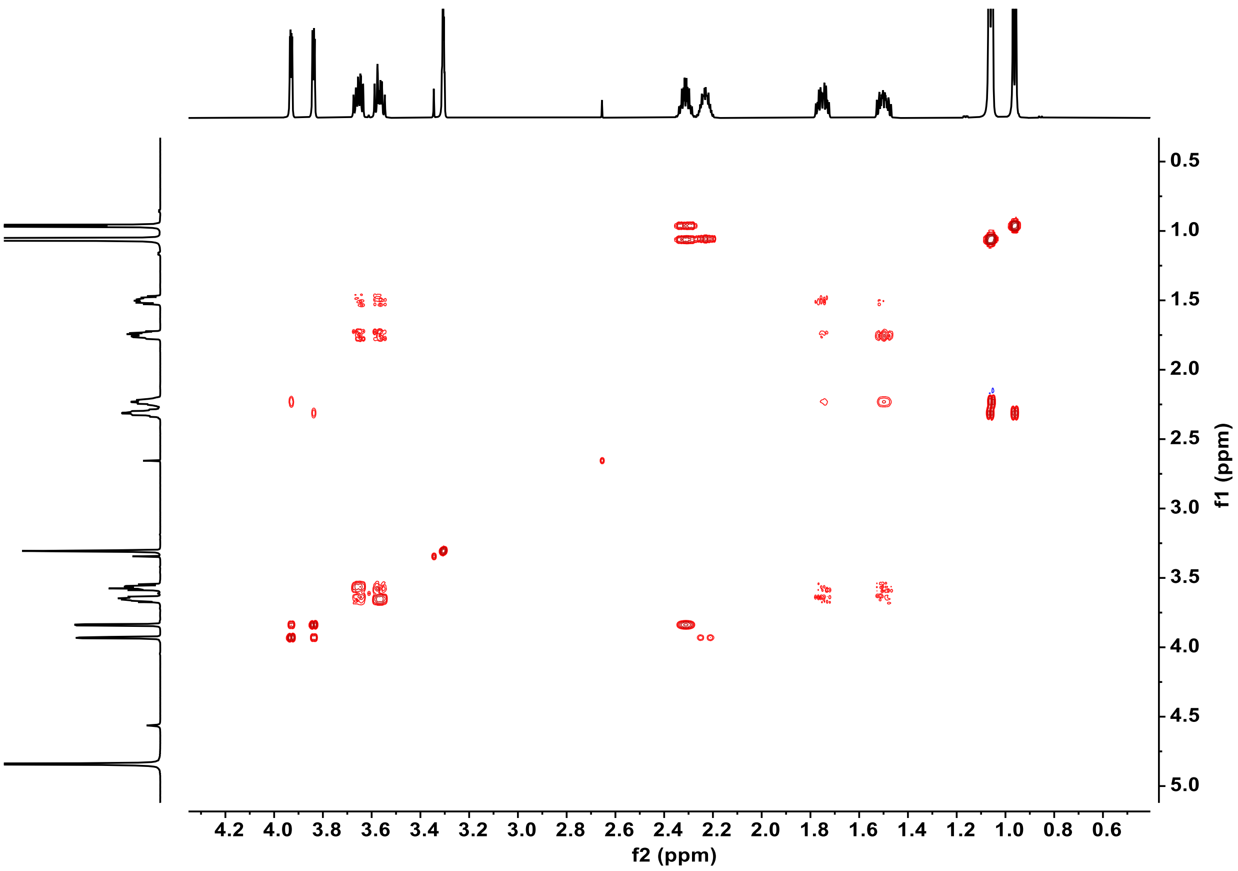

Supplementary Fig. 88. ^{1}H-^{1}H COSY spectrum of **6a** (600 MHz, CD_3OD).

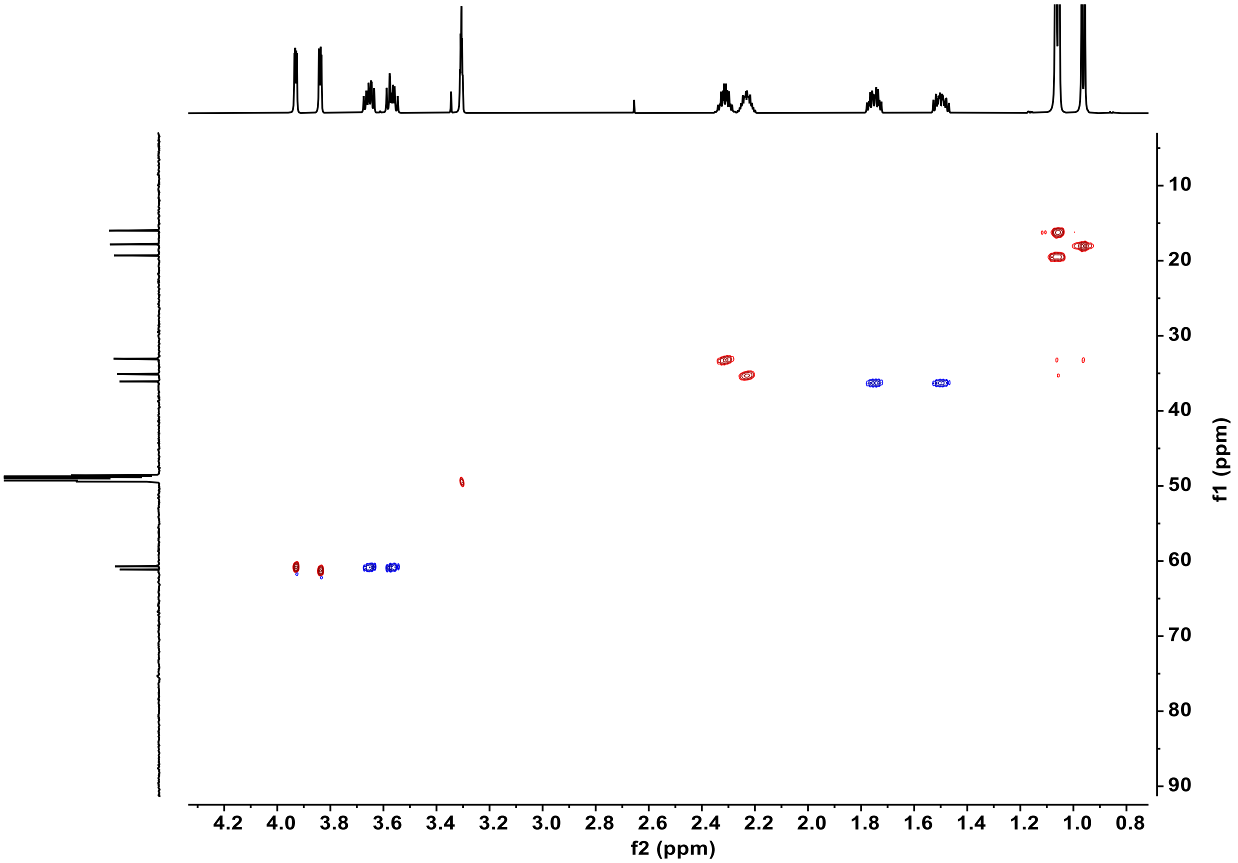

Supplementary Fig. 89. HSQC spectrum of **6a** (600 MHz, CD_3OD).

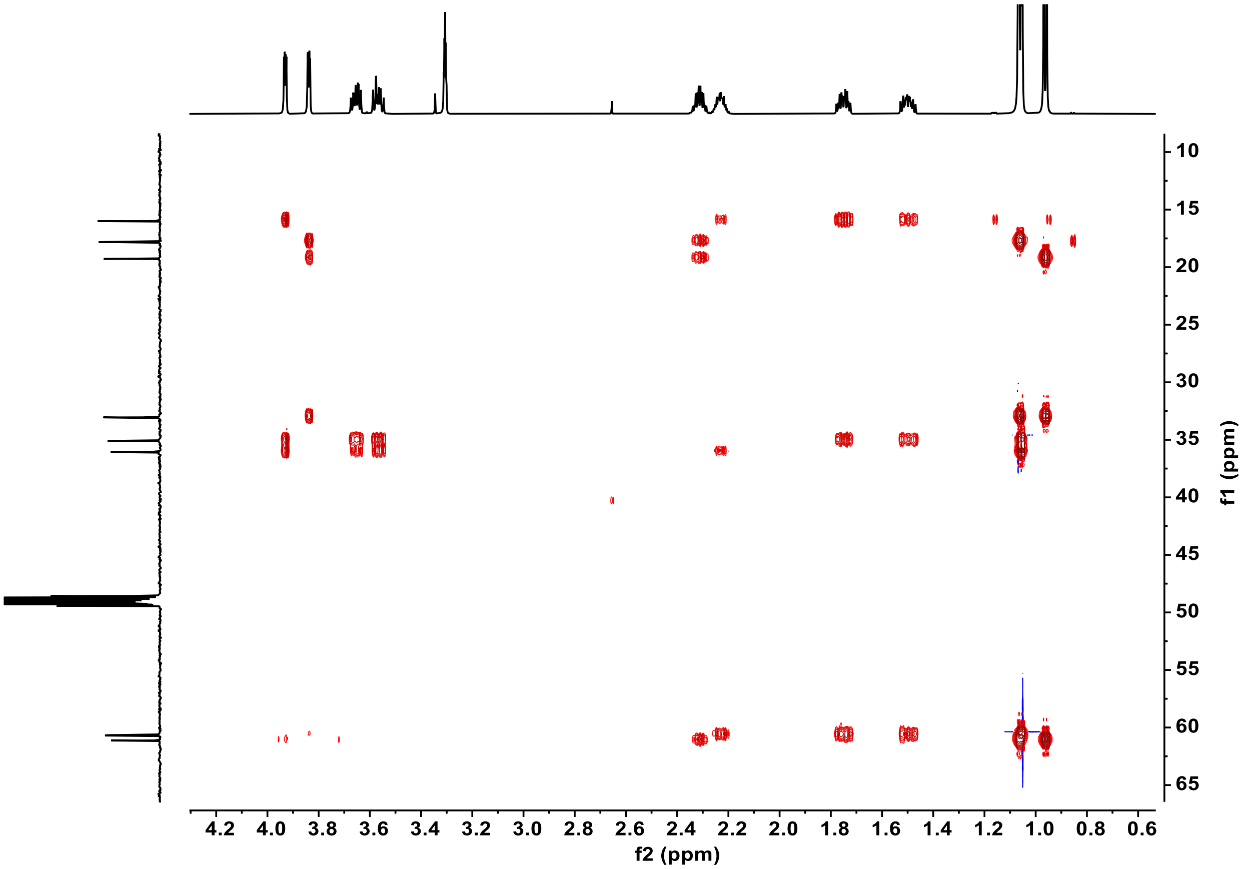

Supplementary Fig. 90. HMBC spectrum of **6a** (600 MHz, CD_3OD).

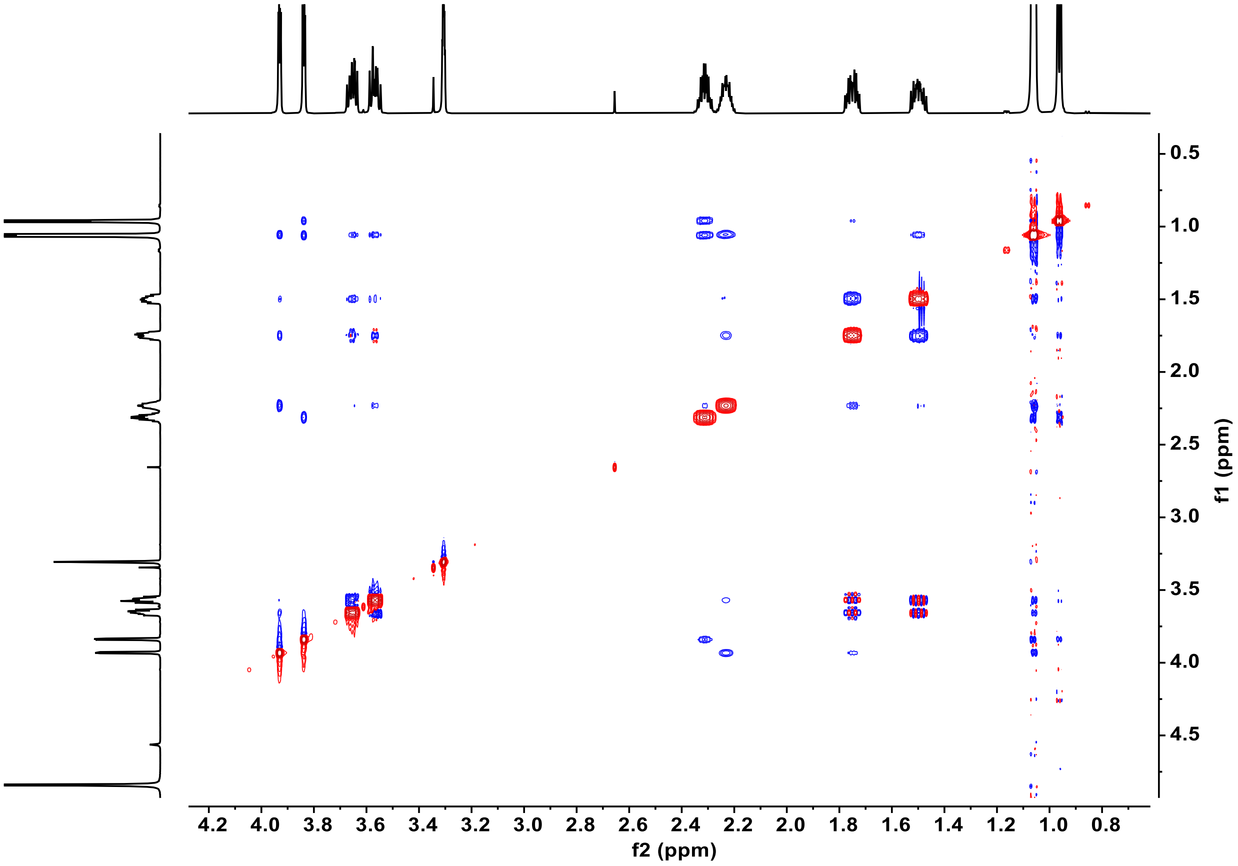

Supplementary Fig. 91. ^{1}H-^{1}H NOESY spectrum of **6a** (600 MHz, CD_3OD).

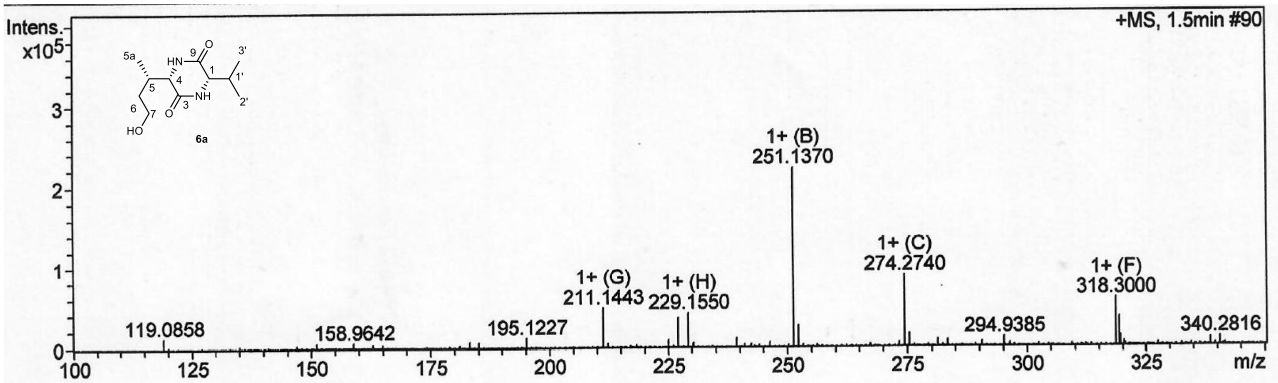

Meas. m/z	#	Formula	Score	m/z	err [ppm]	Mean err [ppm]	mSigma	rdb	e⁻ Conf	N-Rule
229.1550	1	C 11 H 21 N 2 O 3	100.00	229.1547	-1.3	-1.0	2.1	2.5	even	ok
251.1370	1	C 11 H 20 N 2 Na O 3	100.00	251.1366	-1.5	-1.1	3.0	2.5	even	ok

Supplementary Fig. 92. HR-ESI-MS (positive) spectrum of 6a.

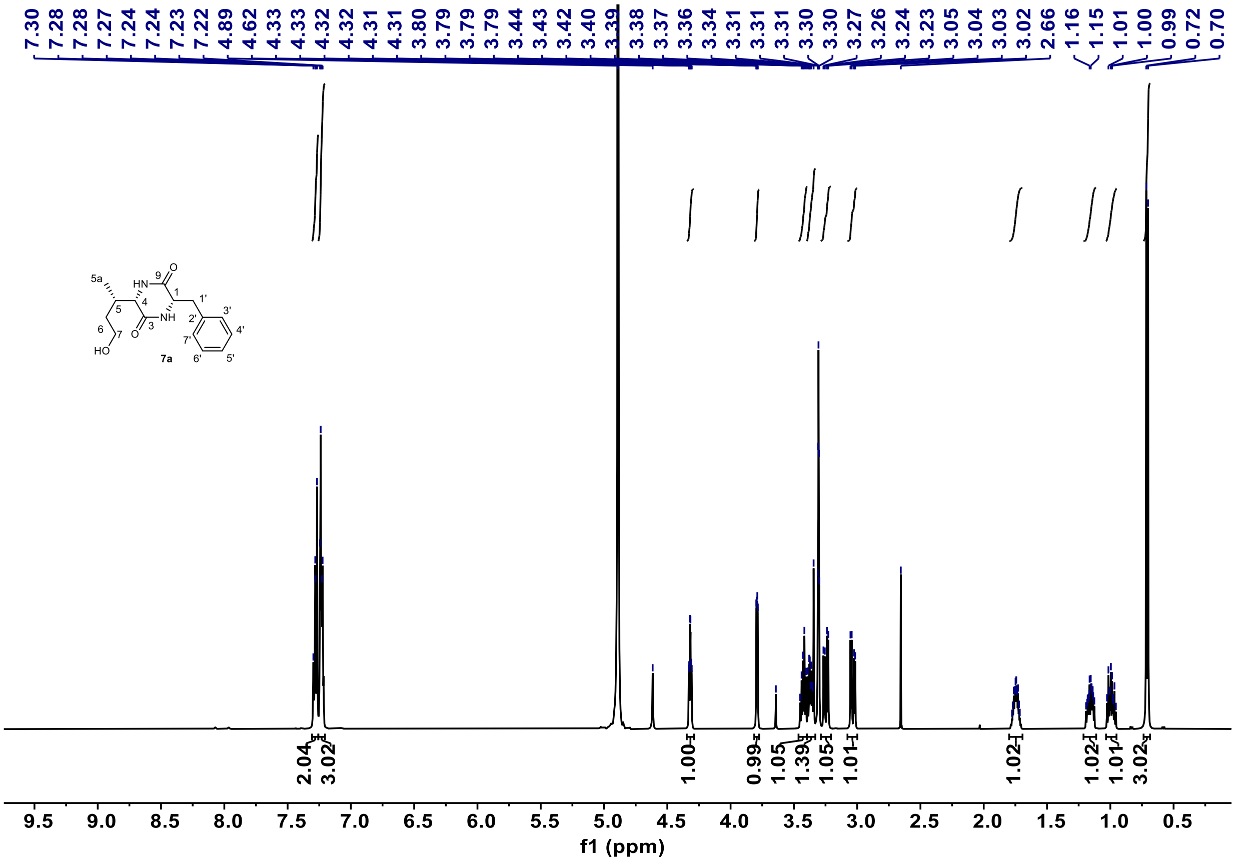

Supplementary Fig. 93. ^{1}H NMR spectrum of **7a** (500 MHz, CD_3OD).

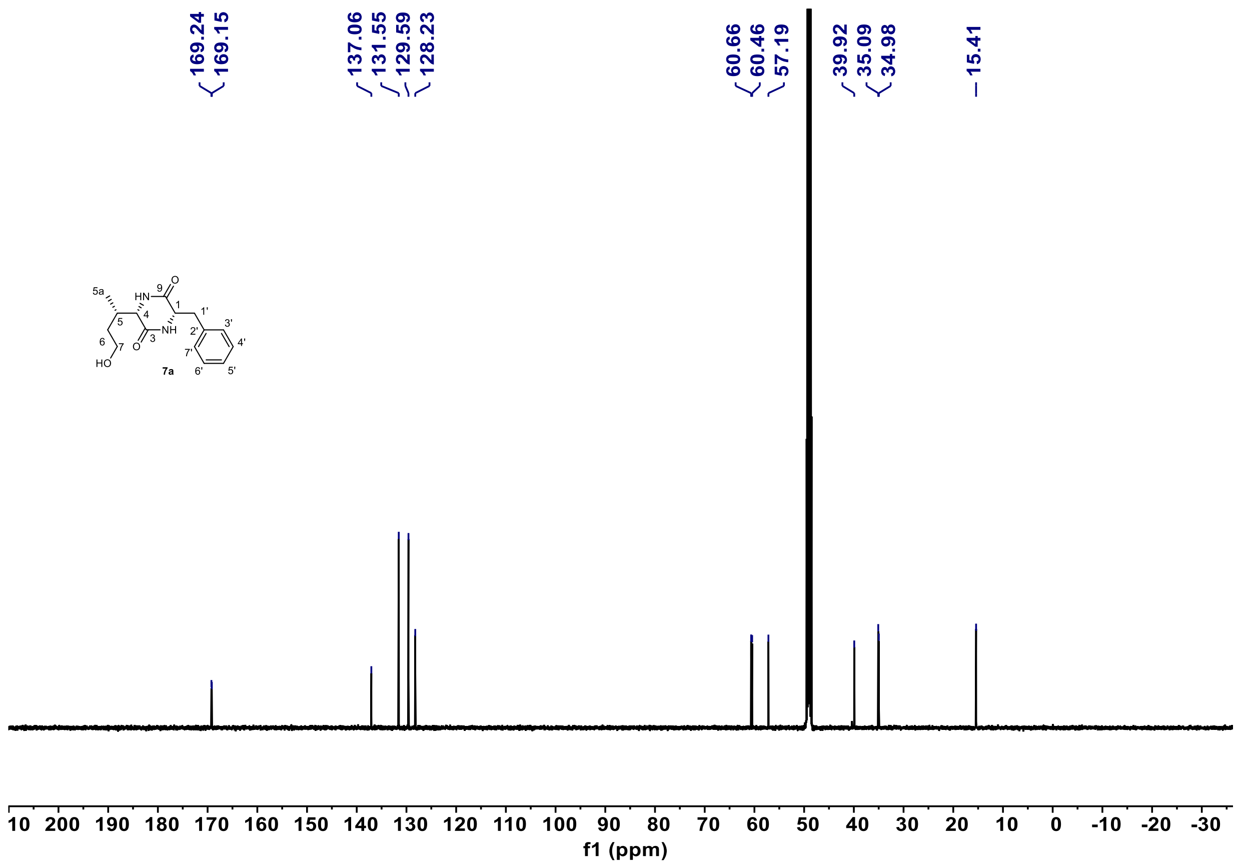

Supplementary Fig. 94. ^{13}C NMR spectrum of **7a** (125 MHz, CD_3OD).

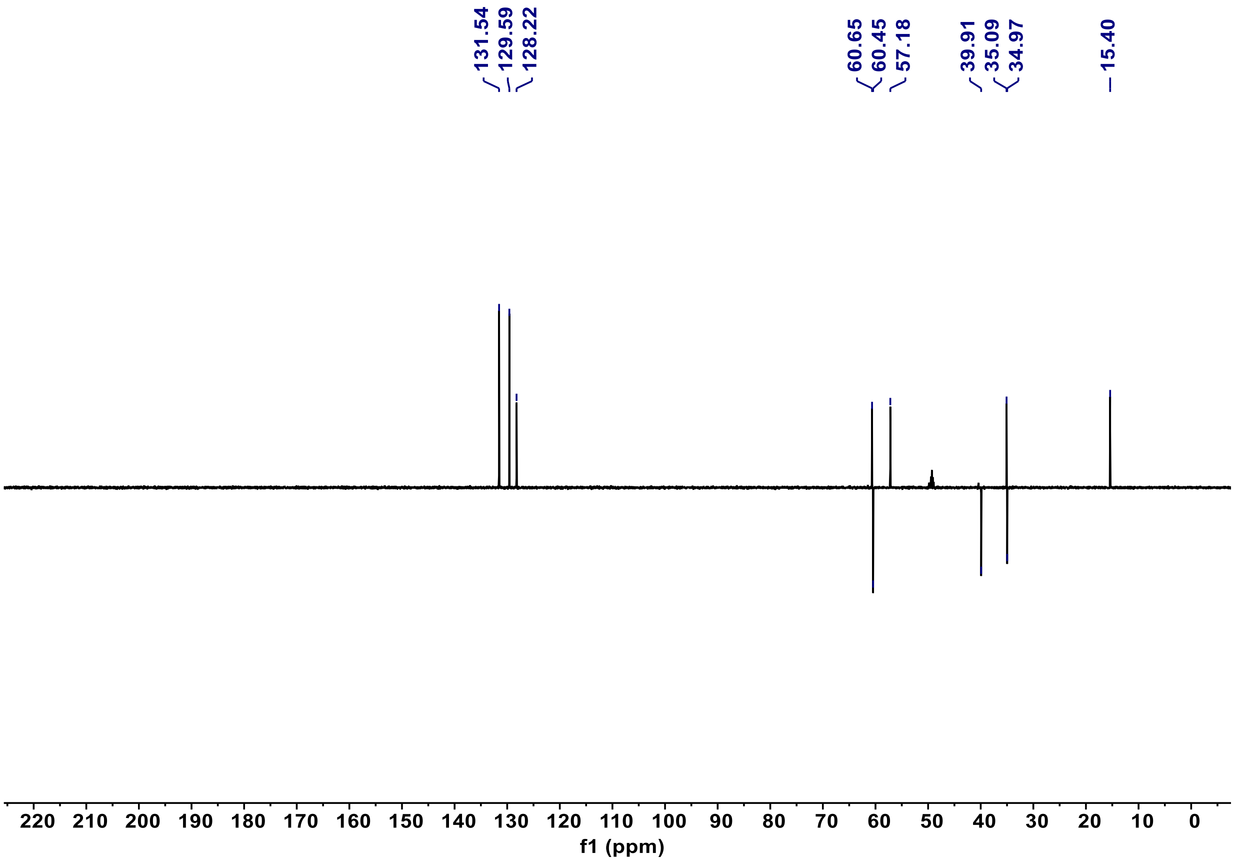

Supplementary Fig. 95. DEPT135 spectrum of **7a** (125 MHz, CD_3OD).

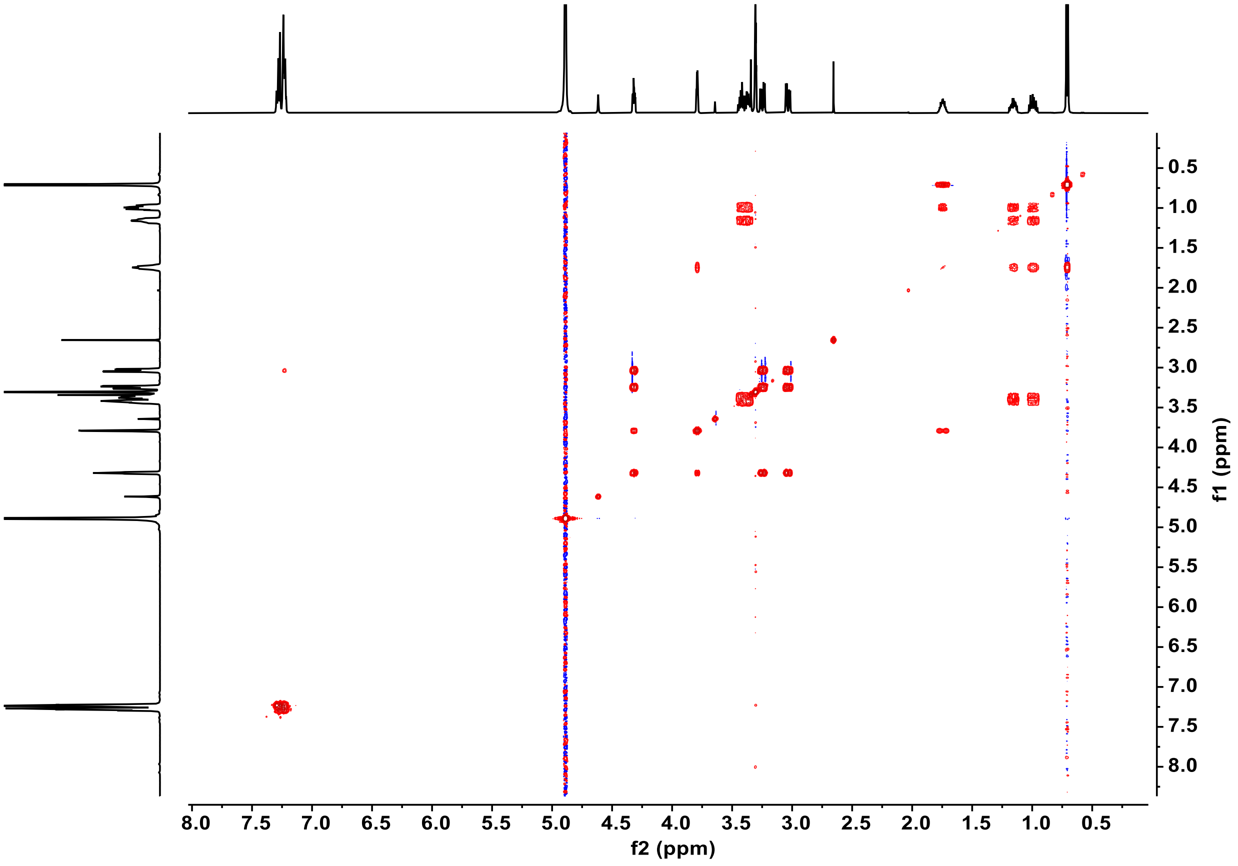

Supplementary Fig. 96. ^{1}H-^{1}H COSY spectrum of **7a** (500 MHz, CD_3OD).

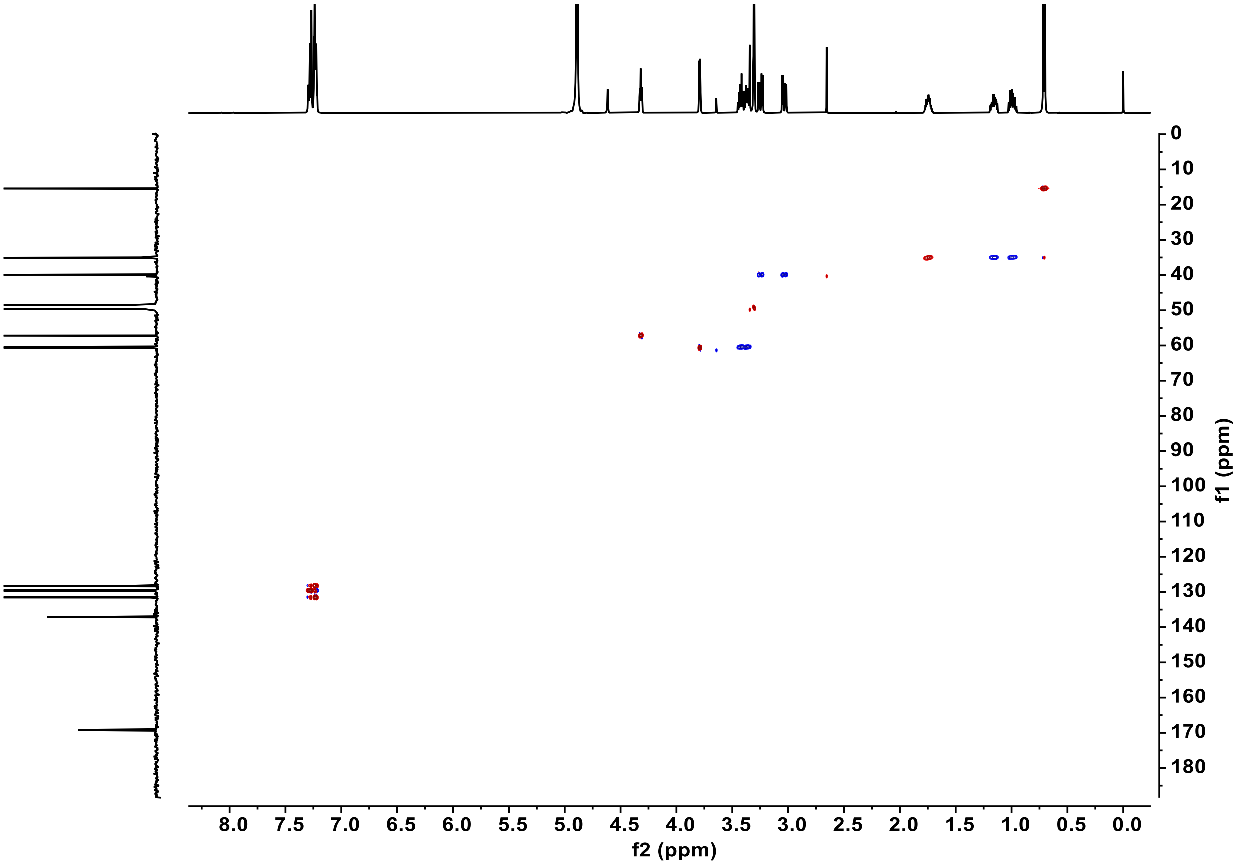

Supplementary Fig. 97. HSQC spectrum of **7a** (500 MHz, CD_3OD).

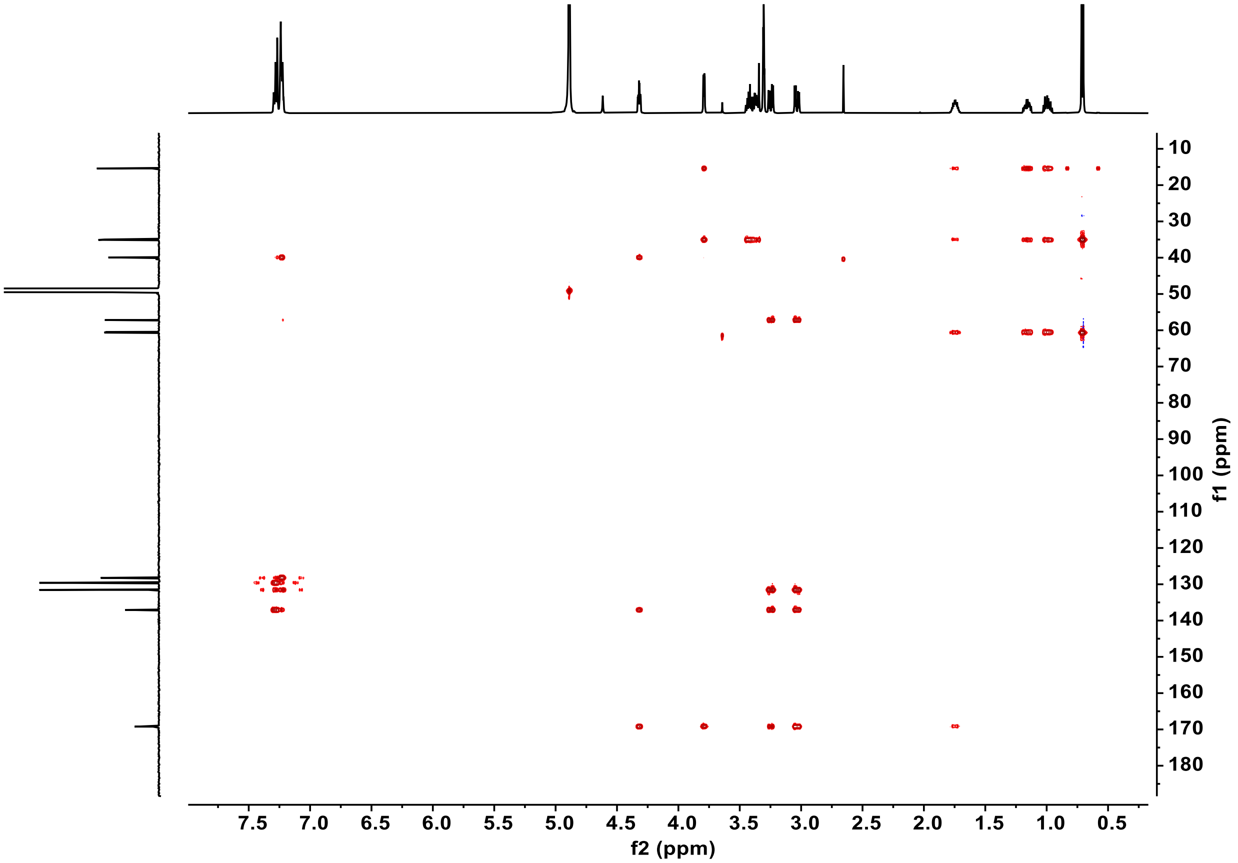

Supplementary Fig. 98. HMBC spectrum of **7a** (500 MHz, CD_3OD).

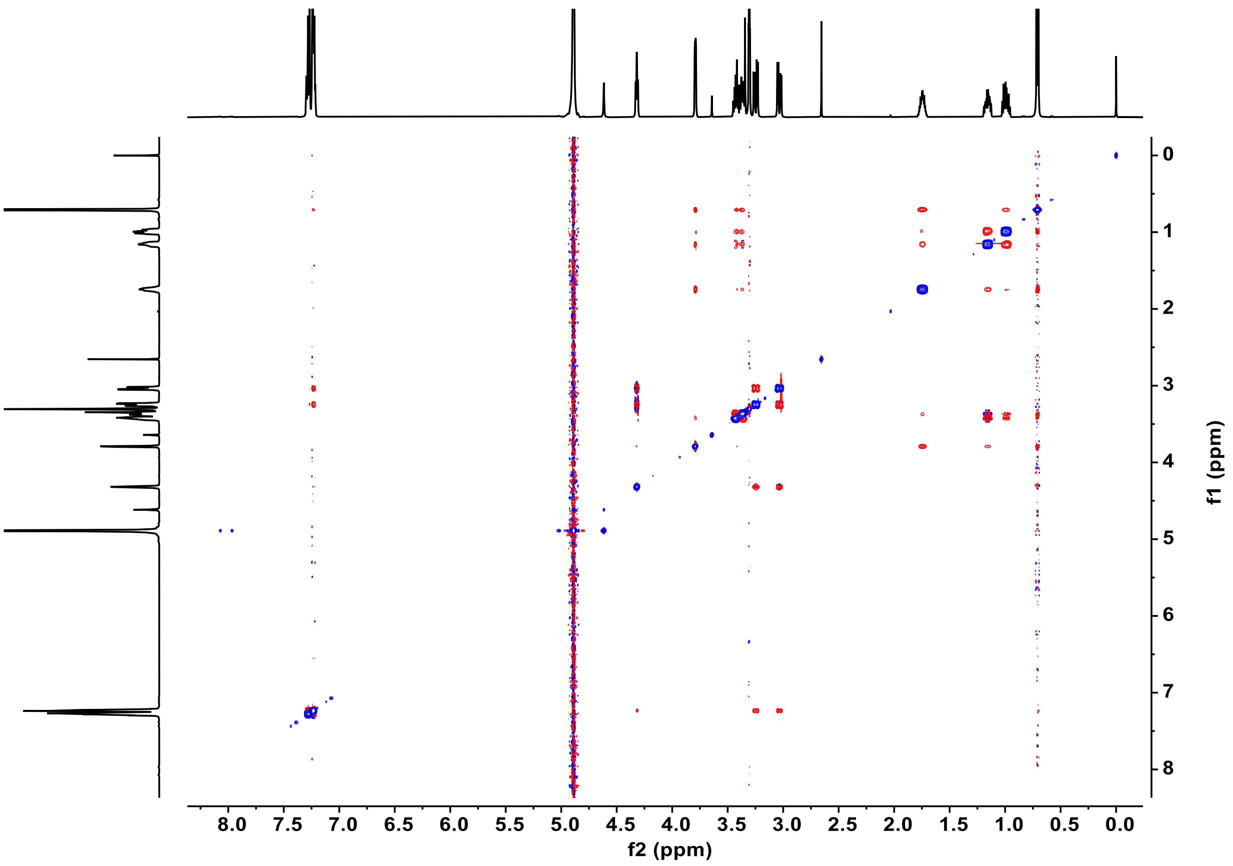

Supplementary Fig. 99. ^{1}H-^{1}H NOESY spectrum of **7a** (500 MHz, CD_3OD).

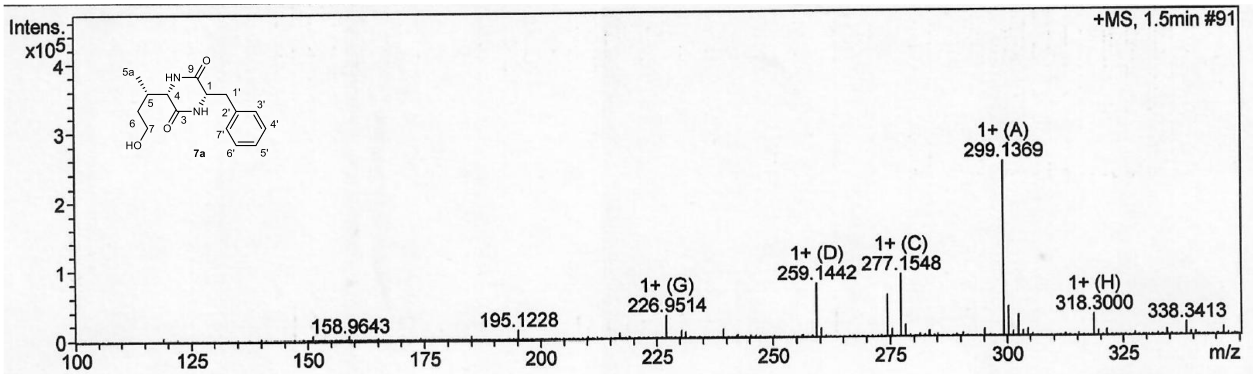

Meas. m/z	#	Formula	Score	m/z	err [ppm]	Mean err [ppm]	mSigma	rdb	e⁻ Conf	N-Rule
277.1548	1	C 15 H 21 N 2 O 3	100.00	277.1547	-0.6	-0.3	5.2	6.5	even	ok
299.1369	1	C 15 H 20 N 2 Na O 3	100.00	299.1366	-0.9	-0.6	3.2	6.5	even	ok

Supplementary Fig. 100. HRSI-MS (positive) spectrum of **7a**.

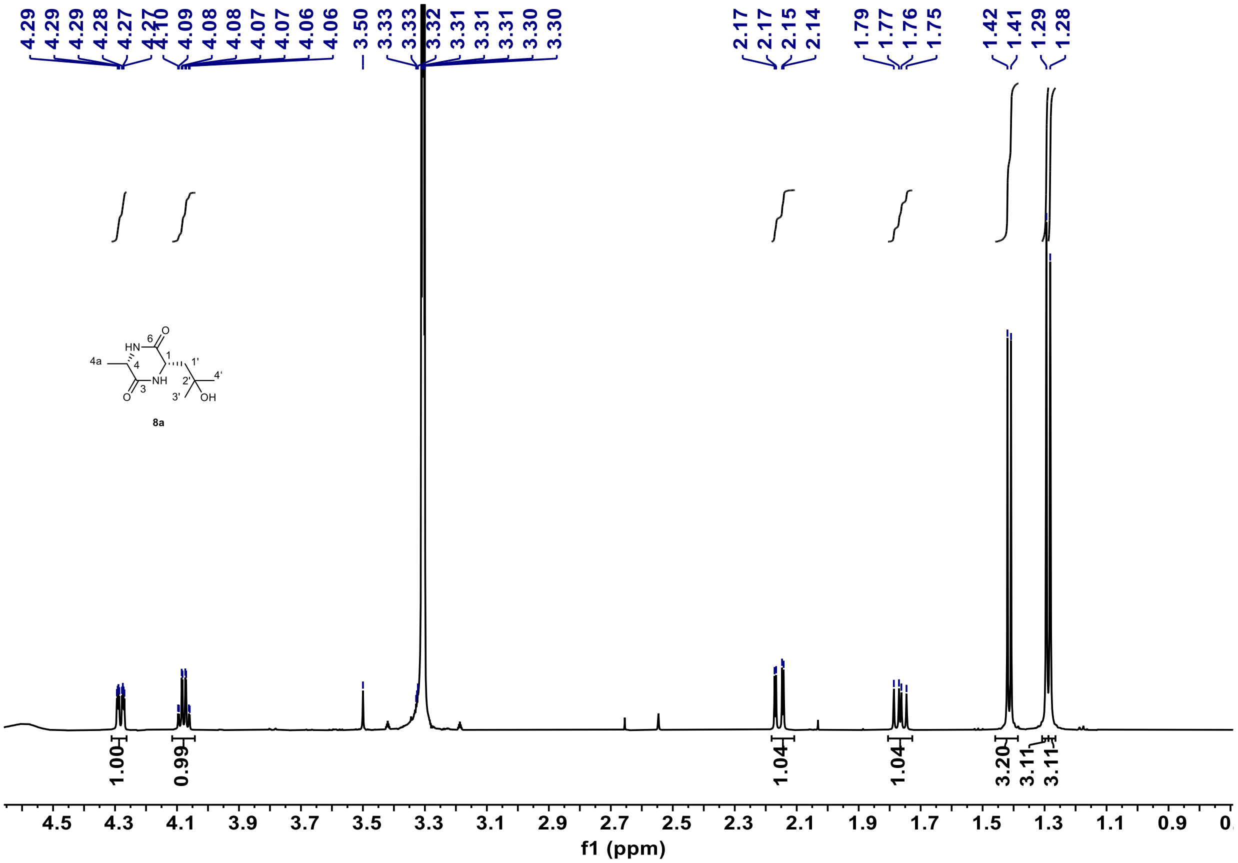

Supplementary Fig. 101. ^{1}H NMR spectrum of **8a** (600 MHz, CD_3OD).

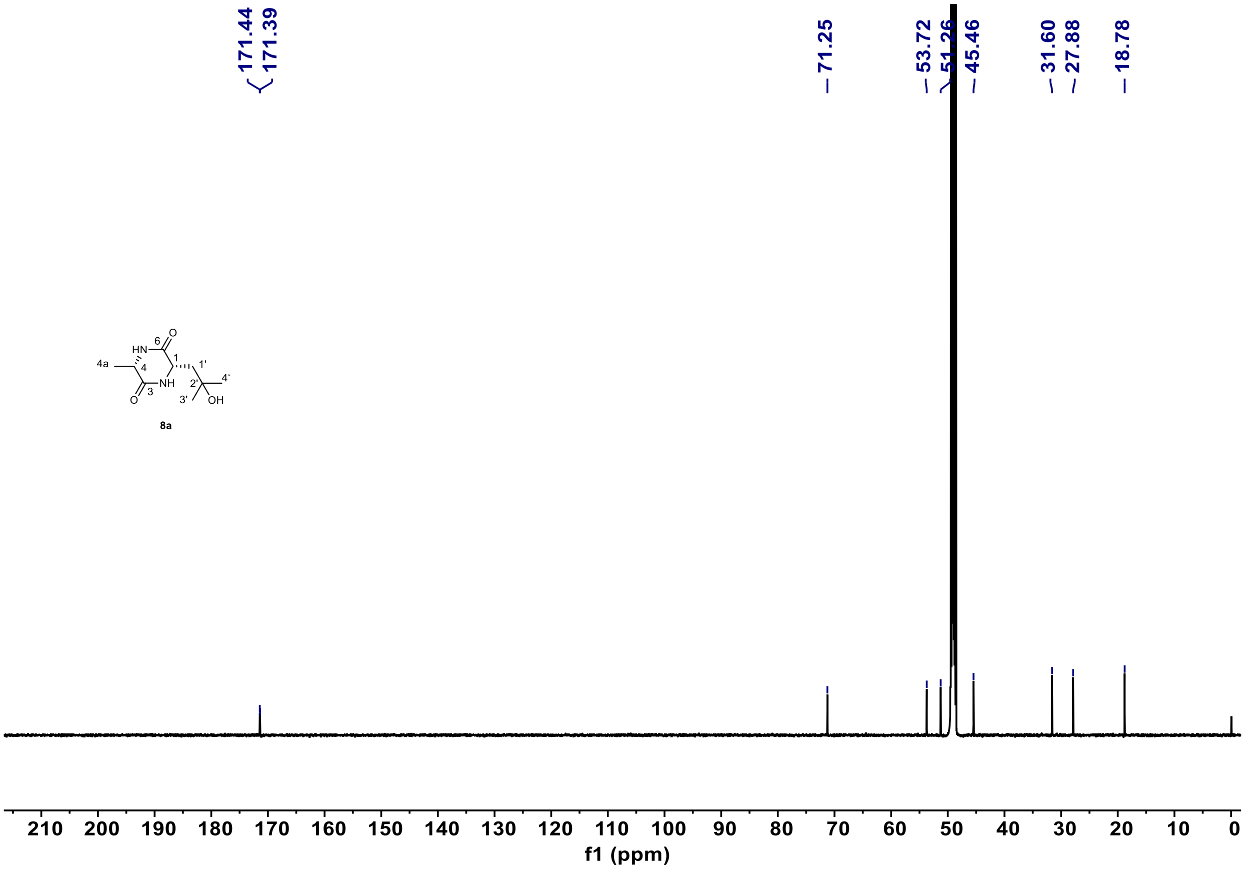

Supplementary Fig. 102. ^{13}C NMR spectrum of **8a** (150 MHz, CD_3OD).

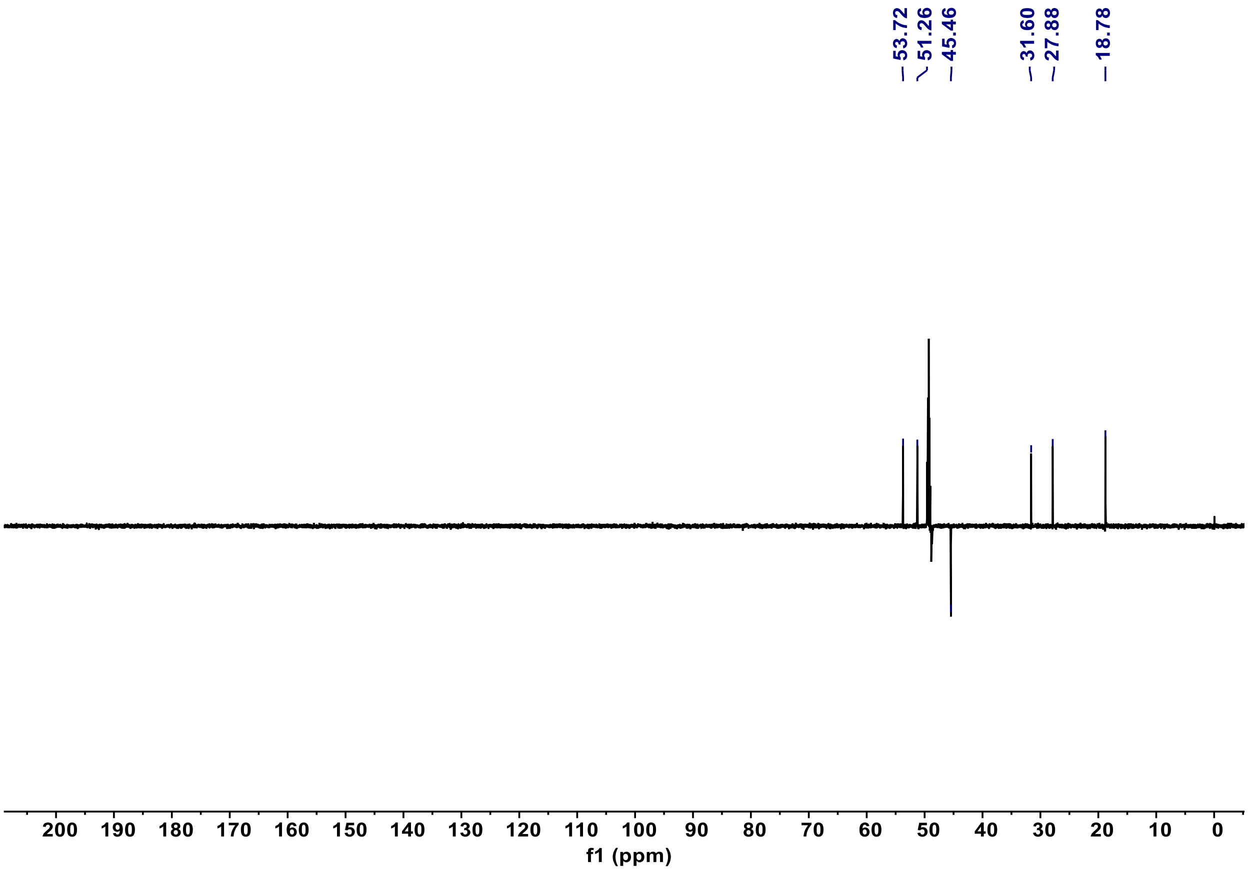

Supplementary Fig. 103. DEPT135 spectrum of **8a** (150 MHz, CD_3OD).

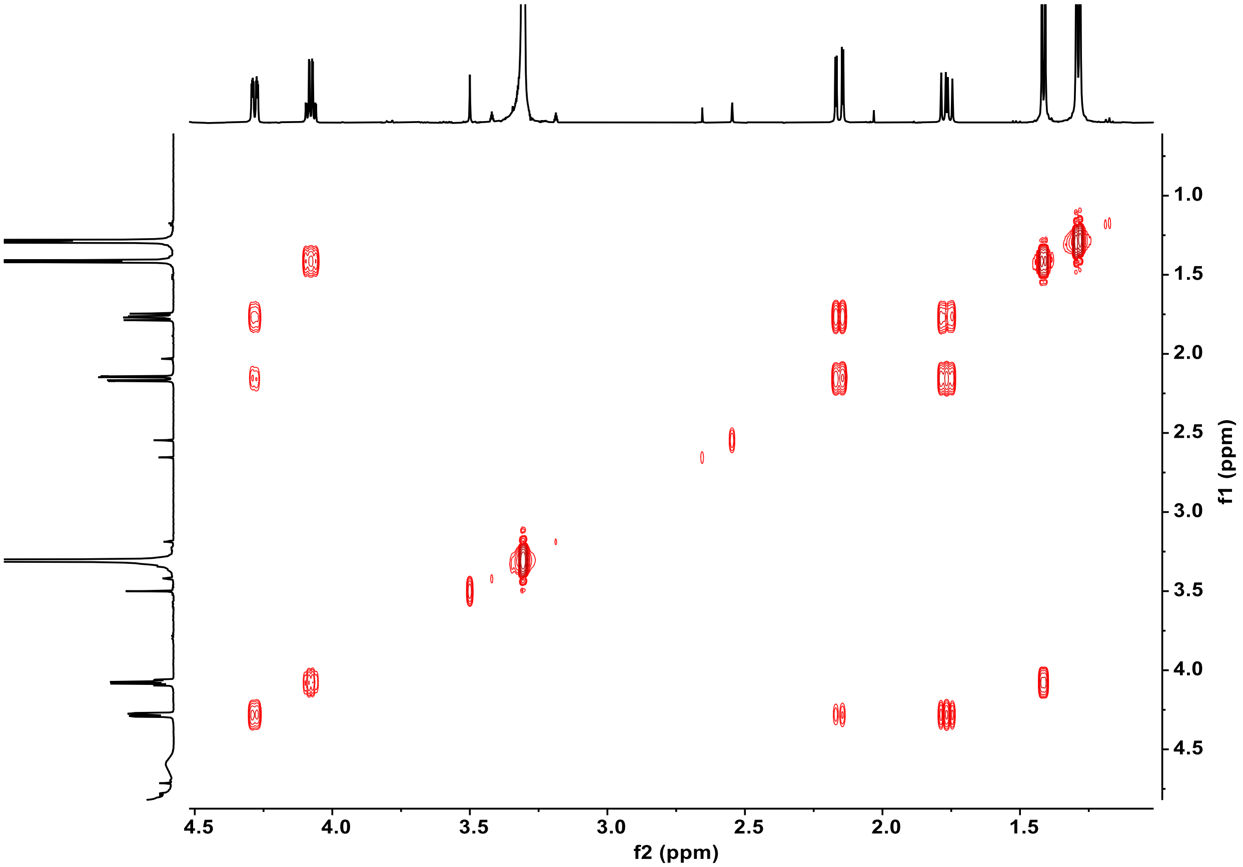

Supplementary Fig. 104. ^{1}H-^{1}H COSY spectrum of **8a** (600 MHz, CD_3OD).

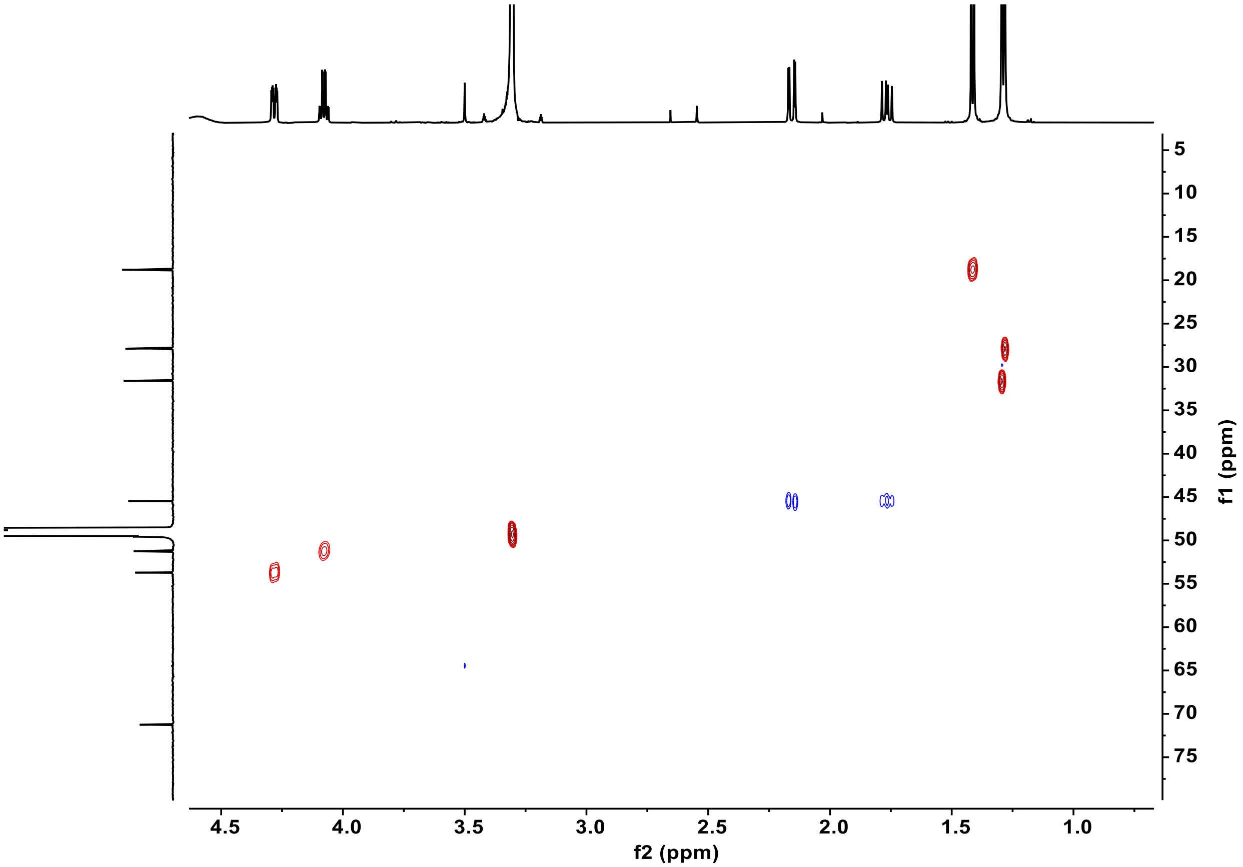

Supplementary Fig. 105. HSQC spectrum of **8a** (600 MHz, CD_3OD).

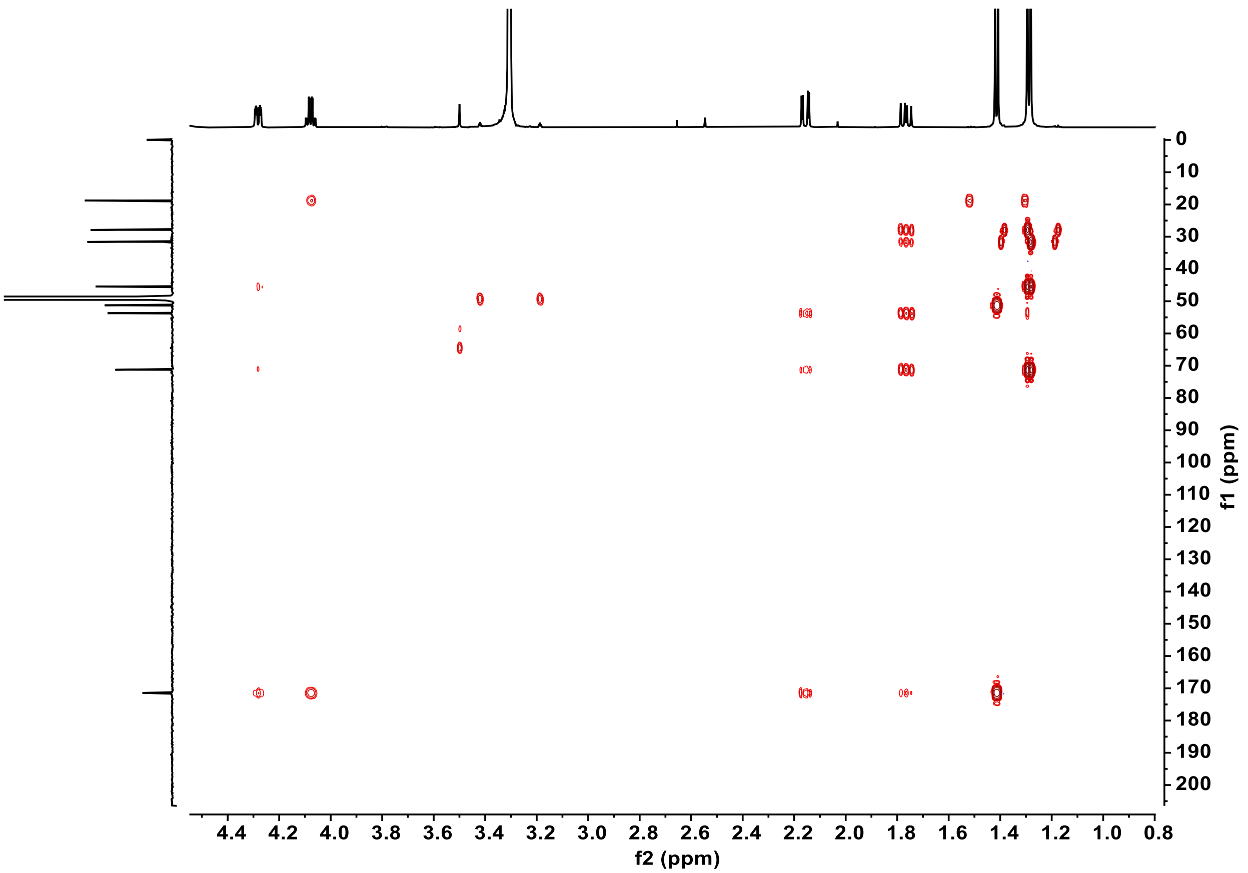

Supplementary Fig. 106. HMBC spectrum of **8a** (600 MHz, CD_3OD).

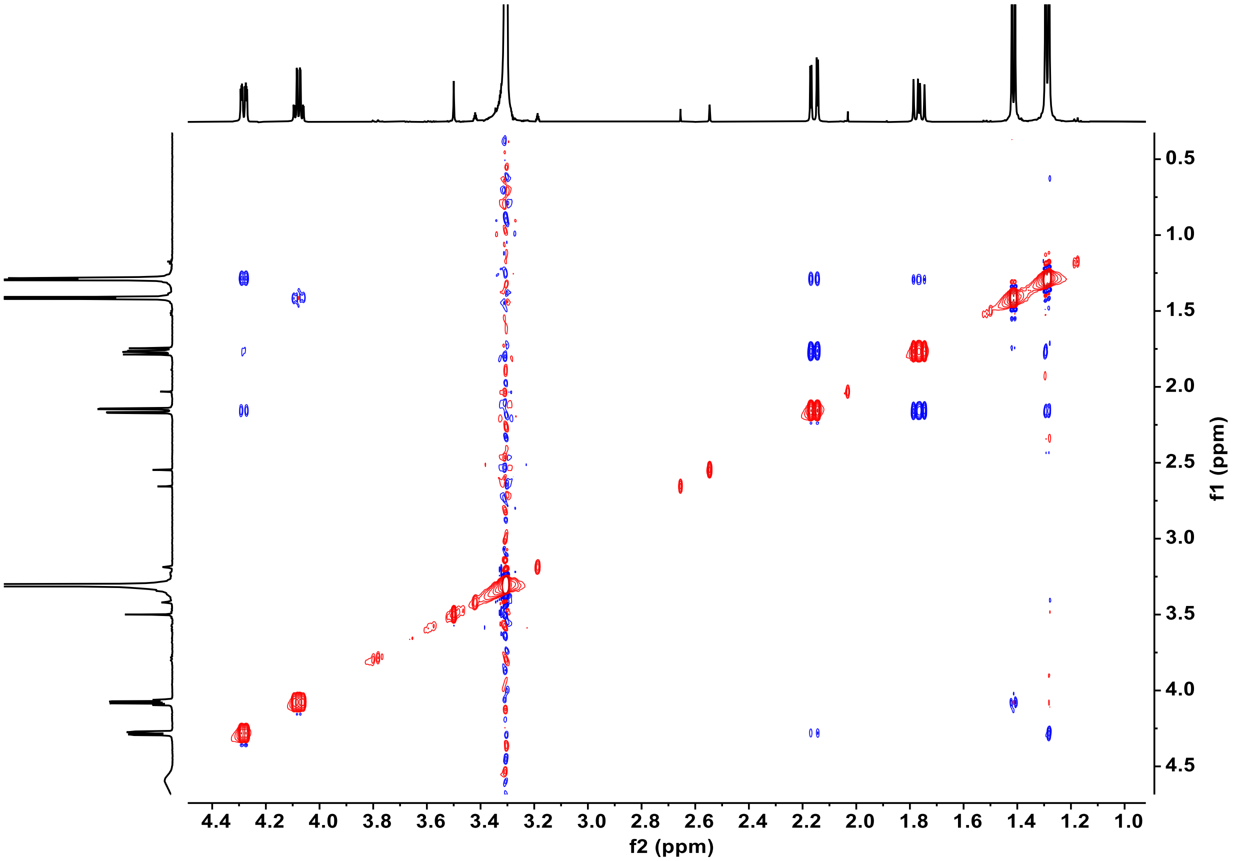

Supplementary Fig. 107. ^{1}H-^{1}H NOESY spectrum of **8a** (600 MHz, CD_3OD).

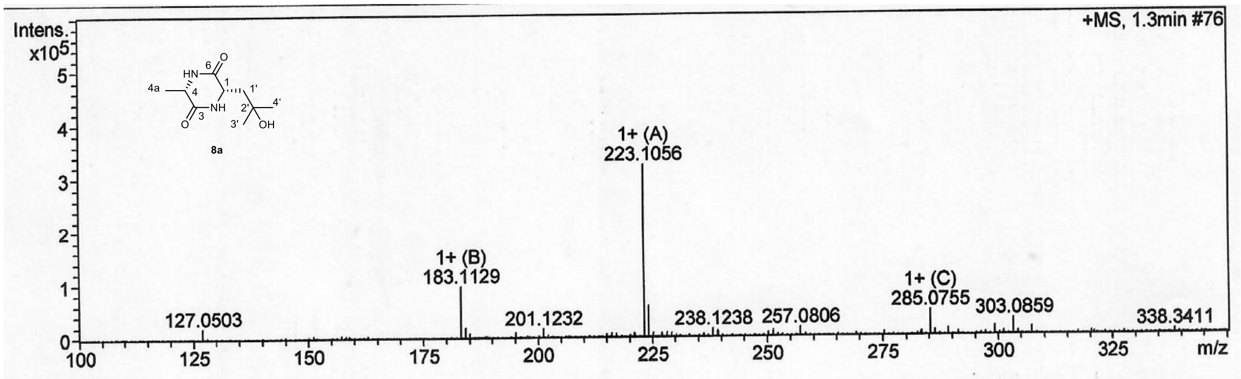

Meas. m/z	#	Formula	Score	m/z	err [ppm]	Mean err [ppm]	mSigma	rdb	e⁻ Conf	N-Rule
201.1232	1	C 9 H 17 N 2 O 3	100.00	201.1234	0.7	-1.3	97.4	2.5	even	ok
223.1056	1	C 9 H 16 N 2 Na O 3	100.00	223.1053	-1.1	-2.5	45.3	2.5	even	ok

Supplementary Fig. 108. HR-ESI-MS (positive) spectrum of **8a**.

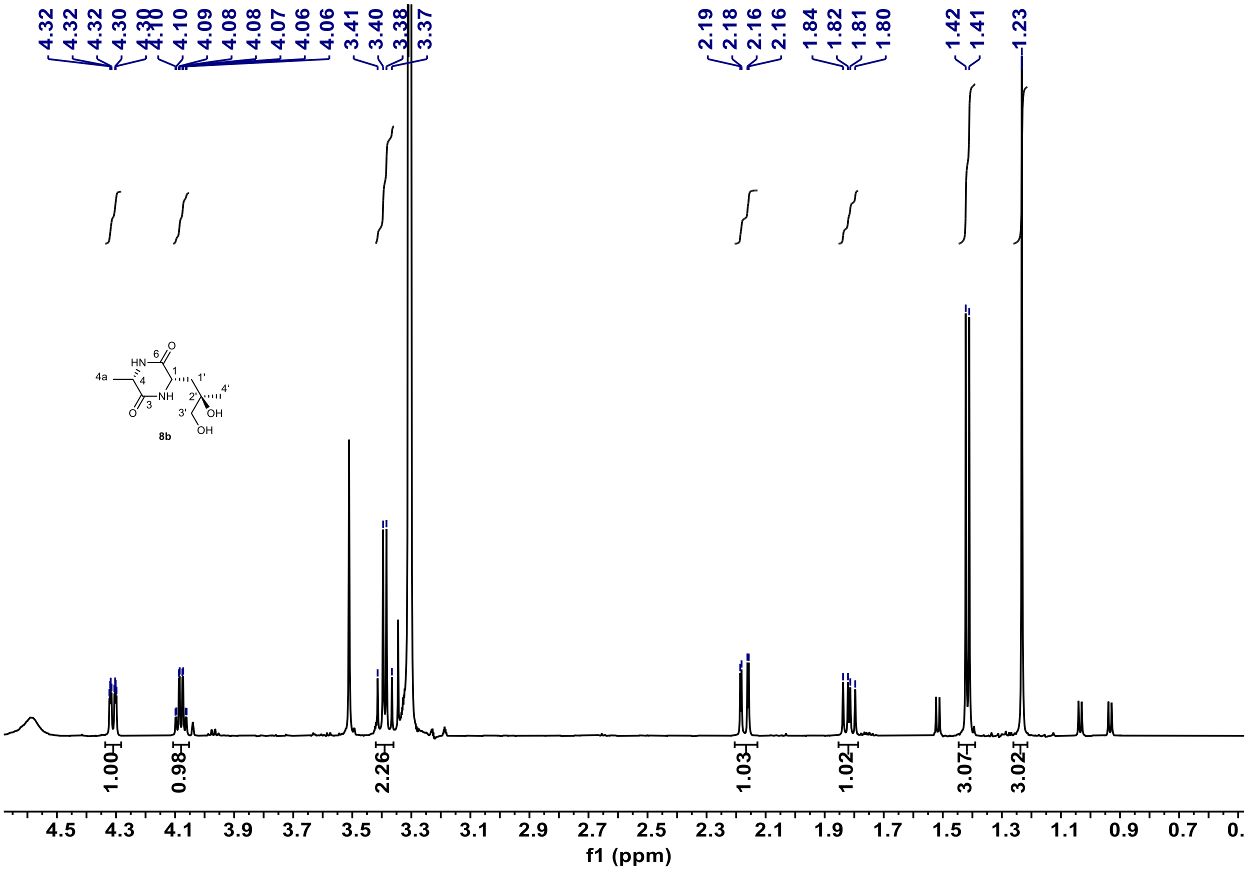

Supplementary Fig. 109. ^{1}H NMR spectrum of **8b** (600 MHz, CD_3OD).

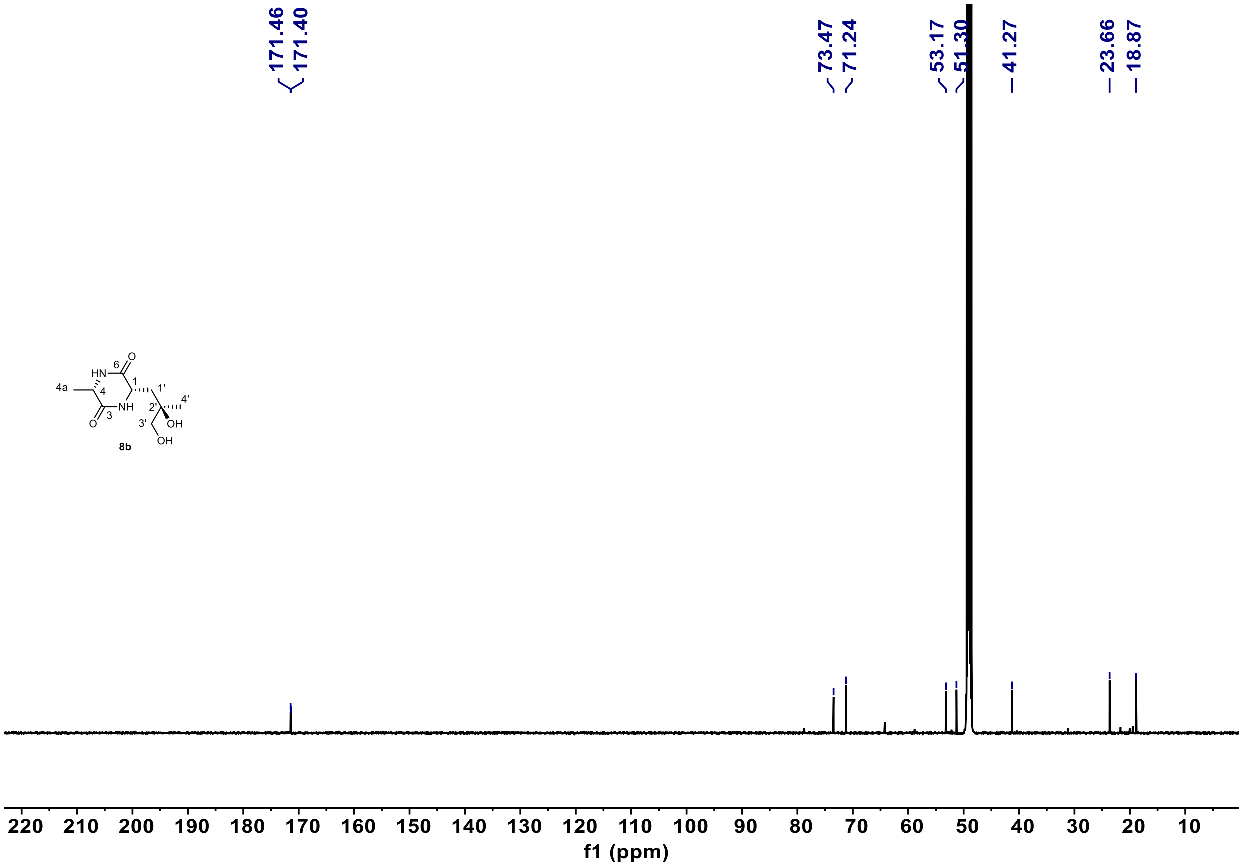

Supplementary Fig. 110. ^{13}C NMR spectrum of **8b** (150 MHz, CD_3OD).

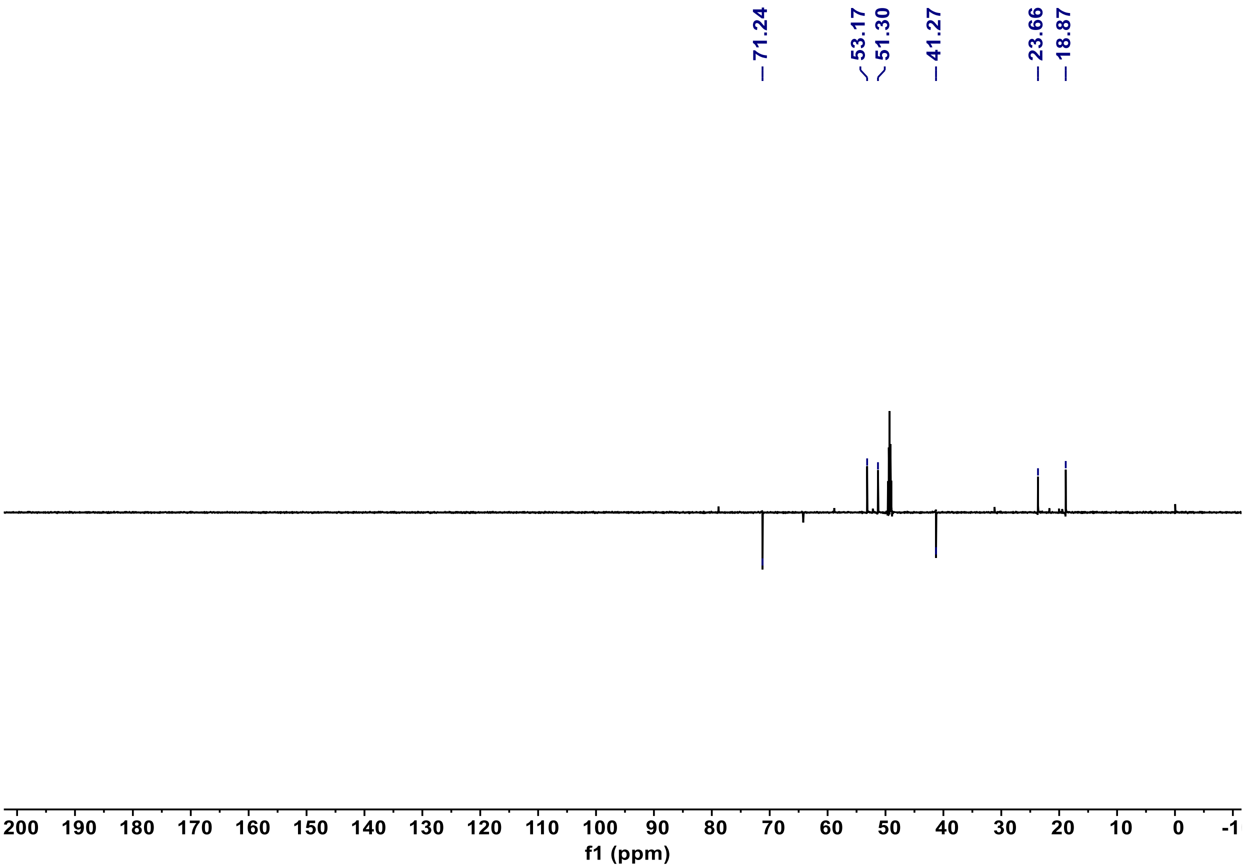

Supplementary Fig. 111. DEPT135 spectrum of **8b** (150 MHz, CD_3OD).

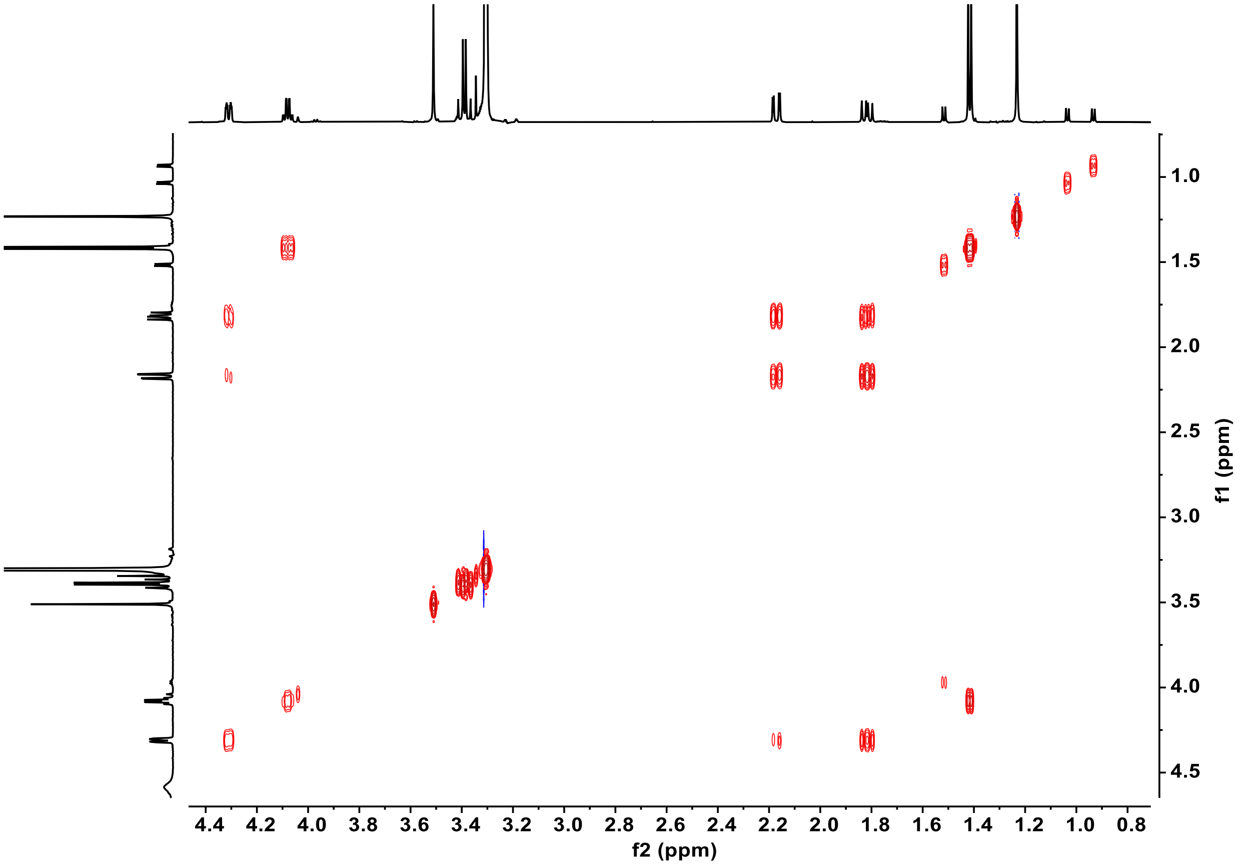

Supplementary Fig. 112. ^{1}H-^{1}H COSY spectrum of **8b** (600 MHz, CD_3OD).

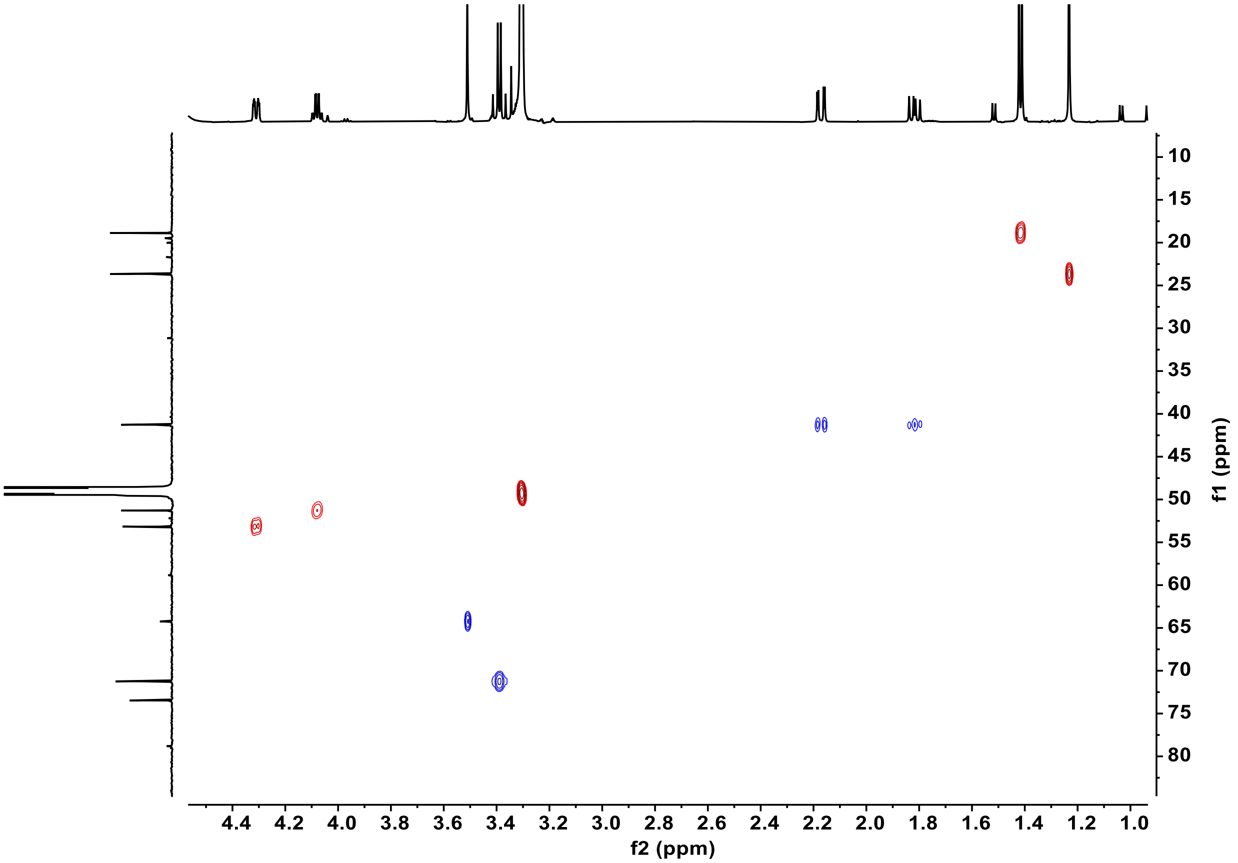

Supplementary Fig. 113. HSQC spectrum of **8b** (600 MHz, CD_3OD).

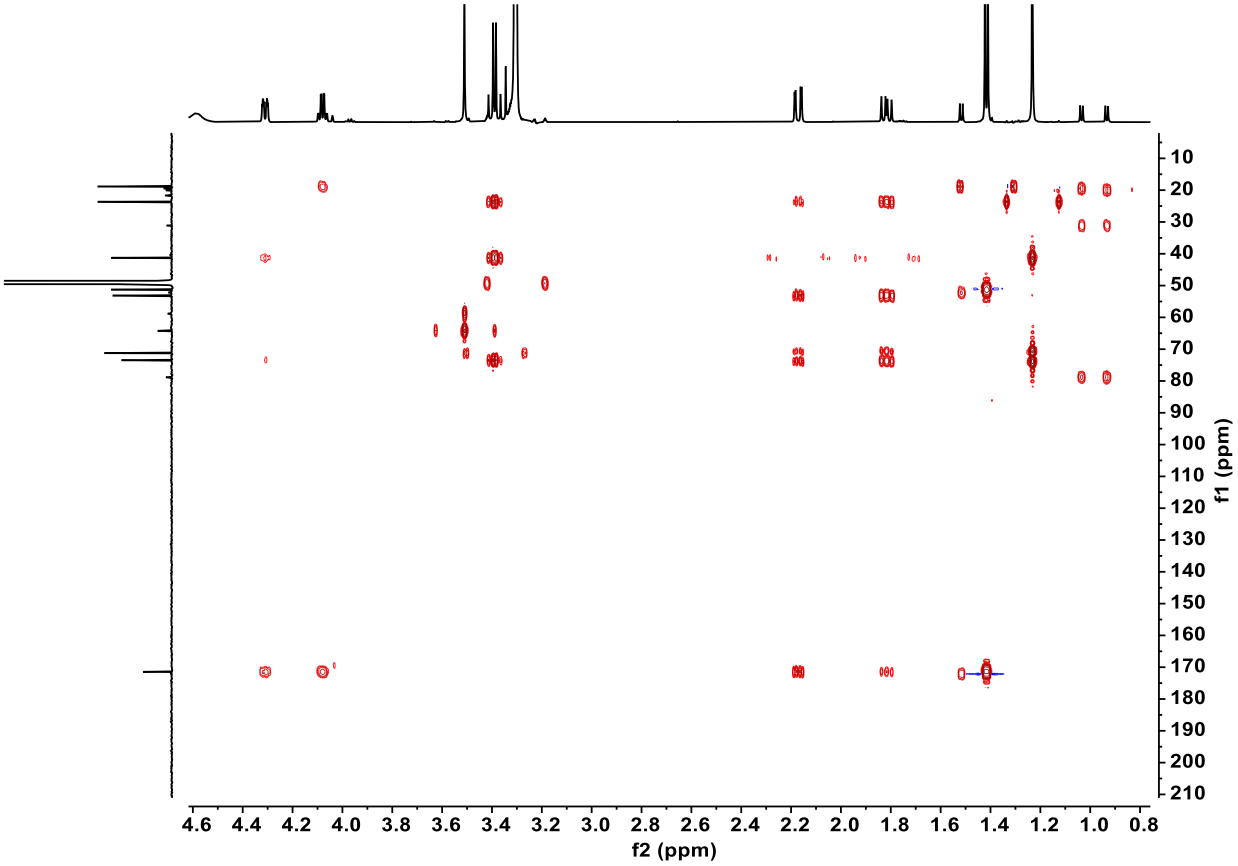

Supplementary Fig. 114. HMBC spectrum of **8b** (600 MHz, CD_3OD).

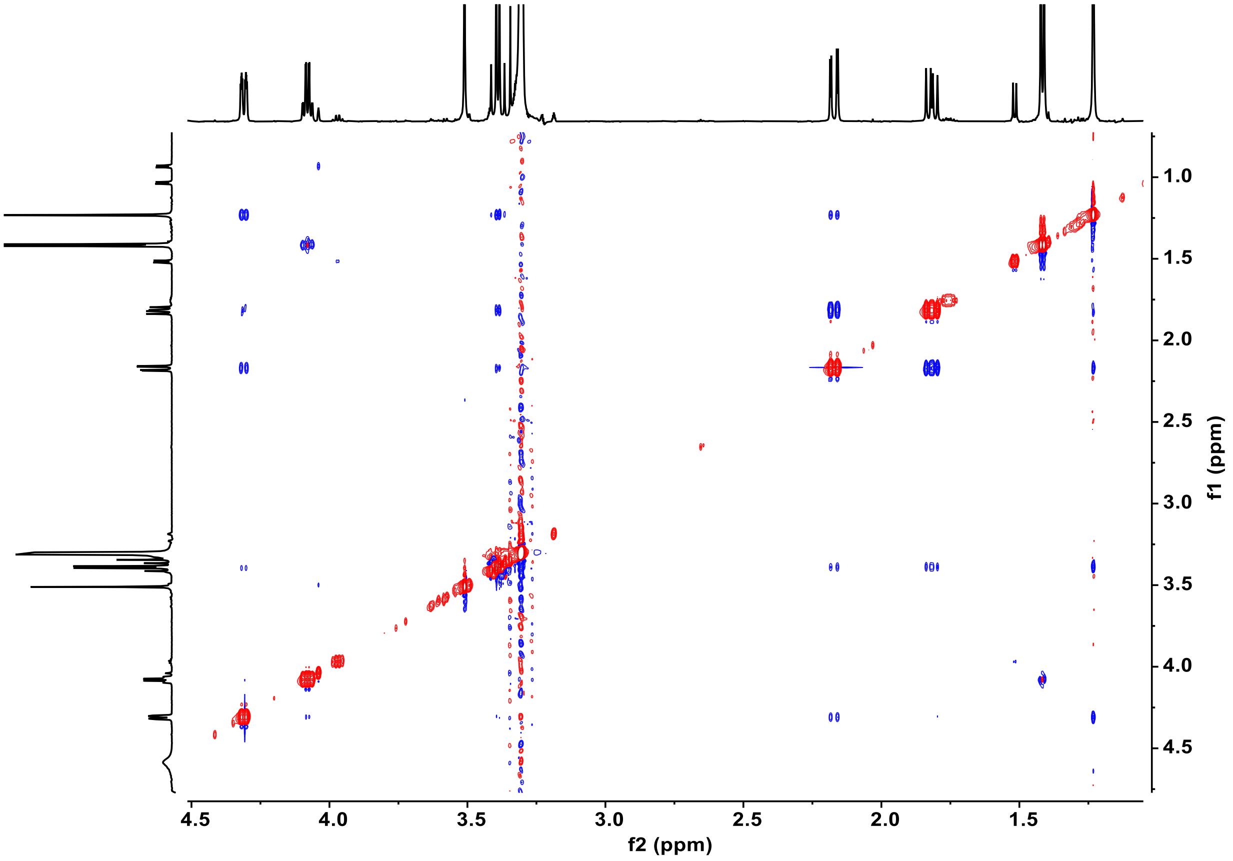

Supplementary Fig. 115. ^1H-^1H NOESY spectrum of **8b** (600 MHz, CD_3OD).

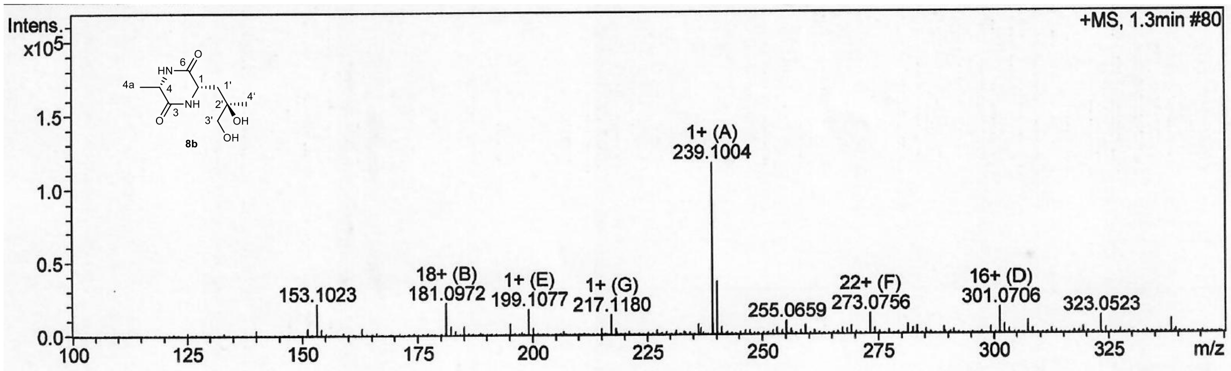

Meas. m/z	#	Formula	Score	m/z	err [ppm]	Mean err [ppm]	mSigma	rdb	e⁻ Conf	N-Rule
217.1180	1	C 9 H 17 N 2 O 4	100.00	217.1183	1.5	-1.2	112.3	2.5	even	ok
239.1004	1	C 9 H 16 N 2 Na O 4	100.00	239.1002	-0.5	-3.0	117.4	2.5	even	ok

Supplementary Fig. 116. HR-ESI-MS (positive) spectrum of **8b**.

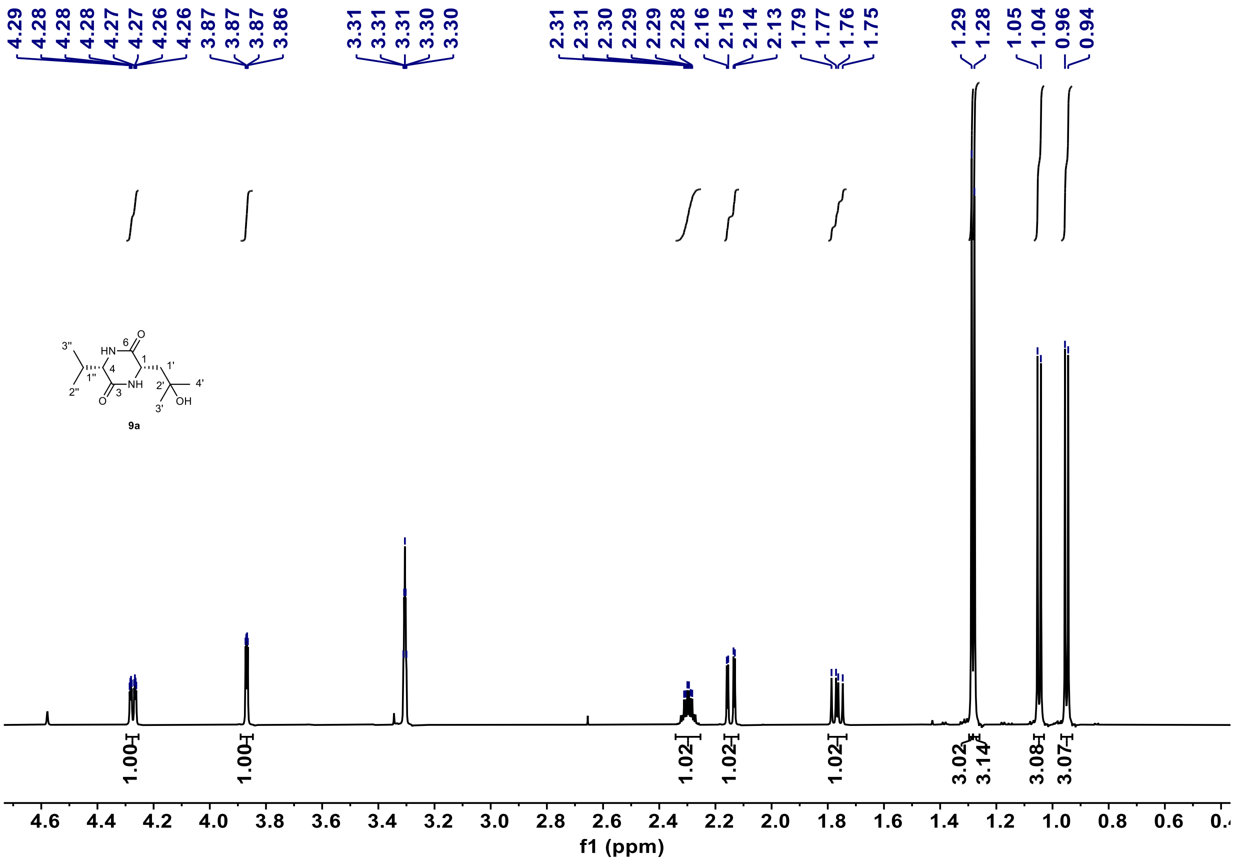

Supplementary Fig. 117. ^{1}H NMR spectrum of **9a** (600 MHz, CD_3OD).

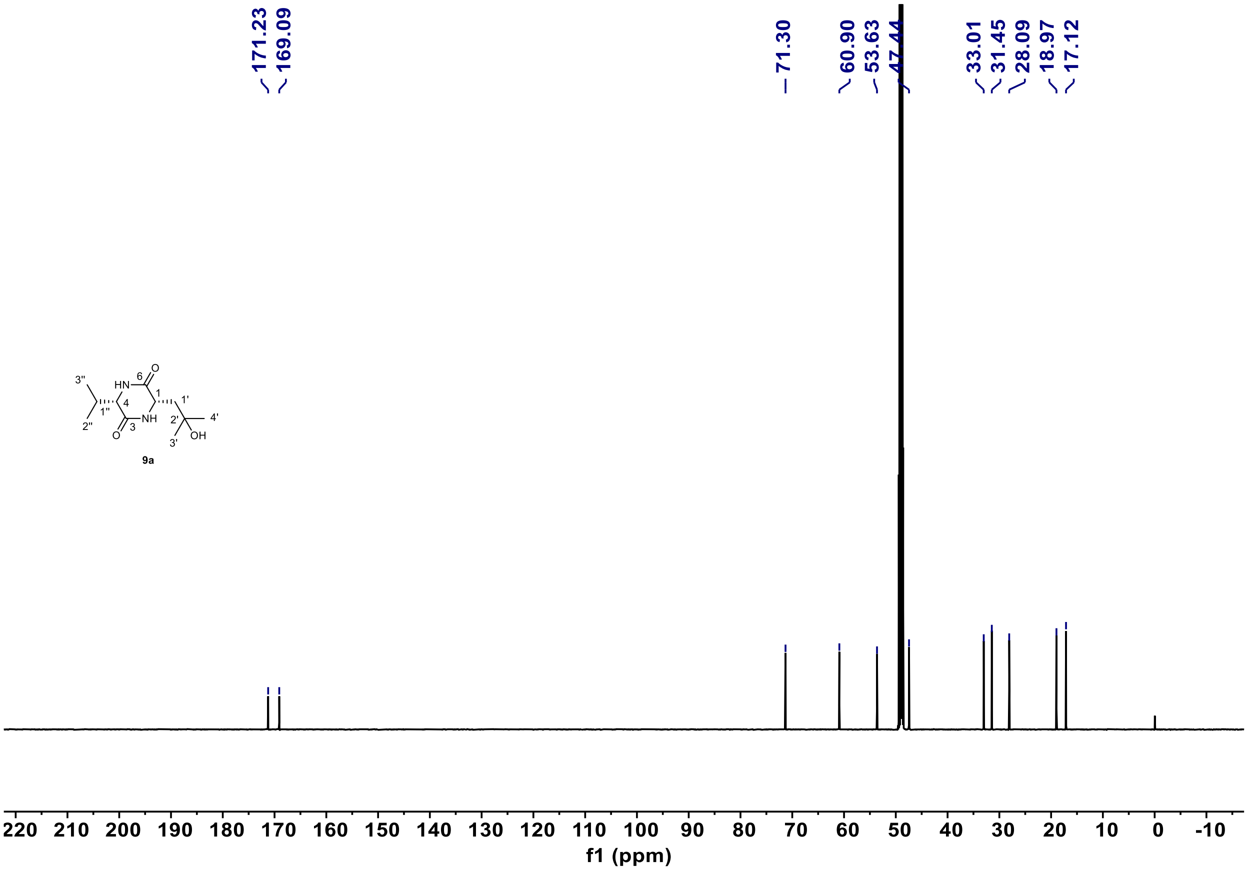

Supplementary Fig. 118. ^{1}H NMR spectrum of **9a** (600 MHz, CD_3OD).

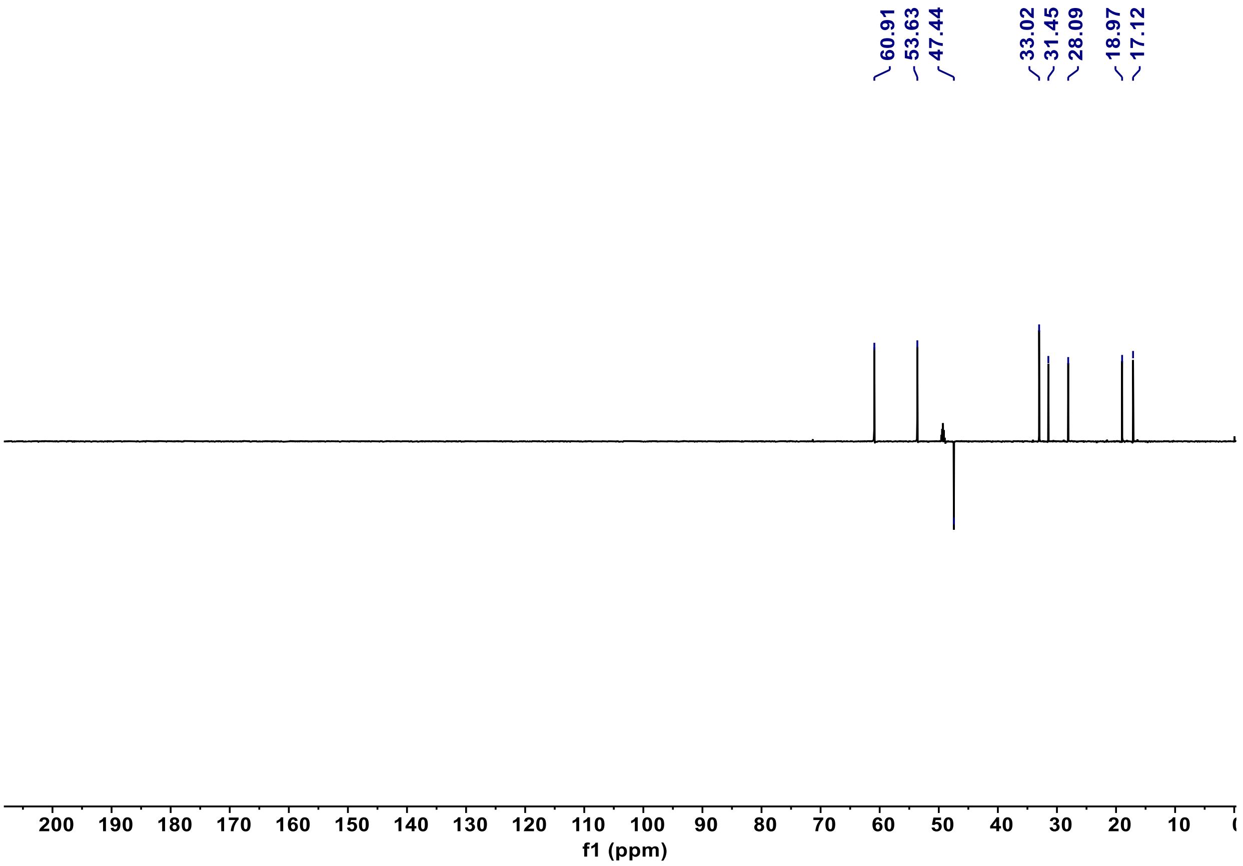

Supplementary Fig. 119. DEPT135 spectrum of **9a** (150 MHz, CD_3OD).

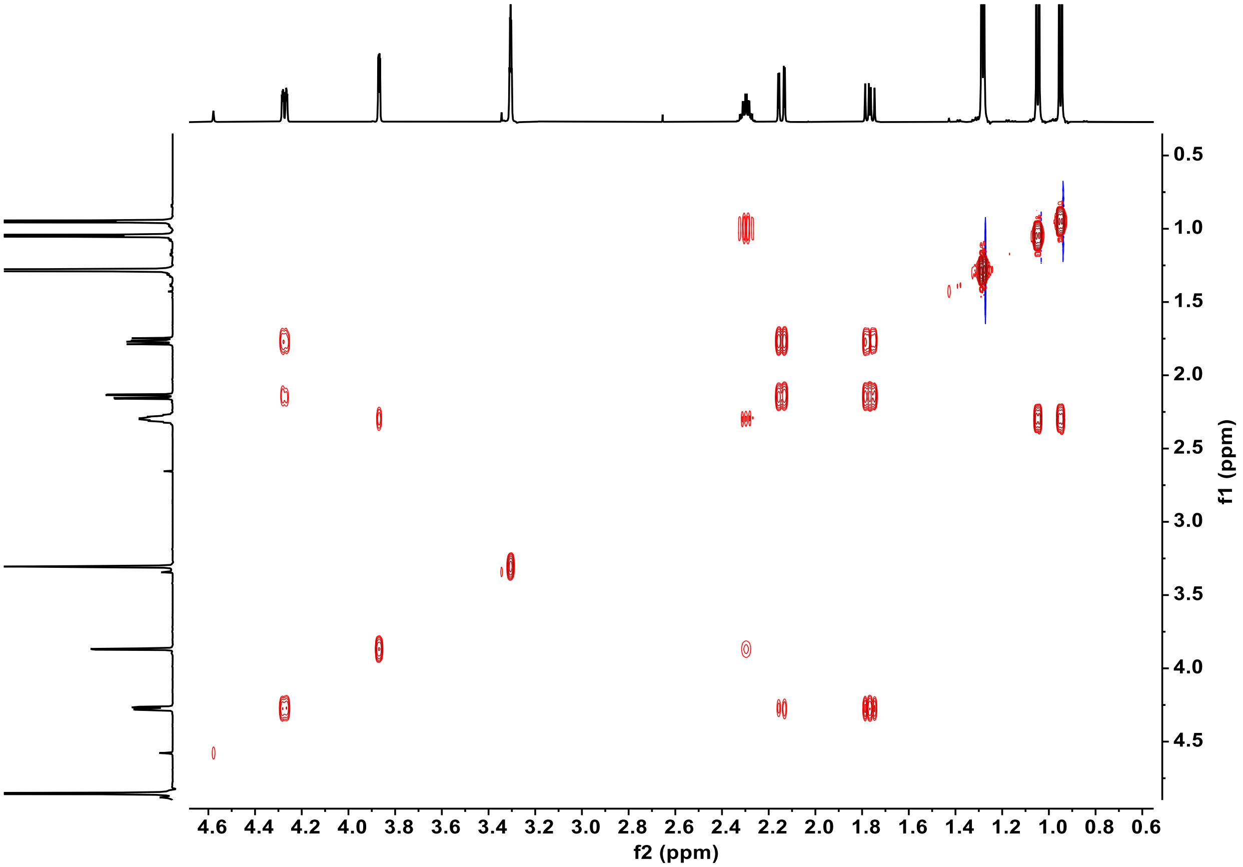

Supplementary Fig. 120. ^{1}H-^{1}H COSY spectrum of **9a** (600 MHz, CD_3OD).

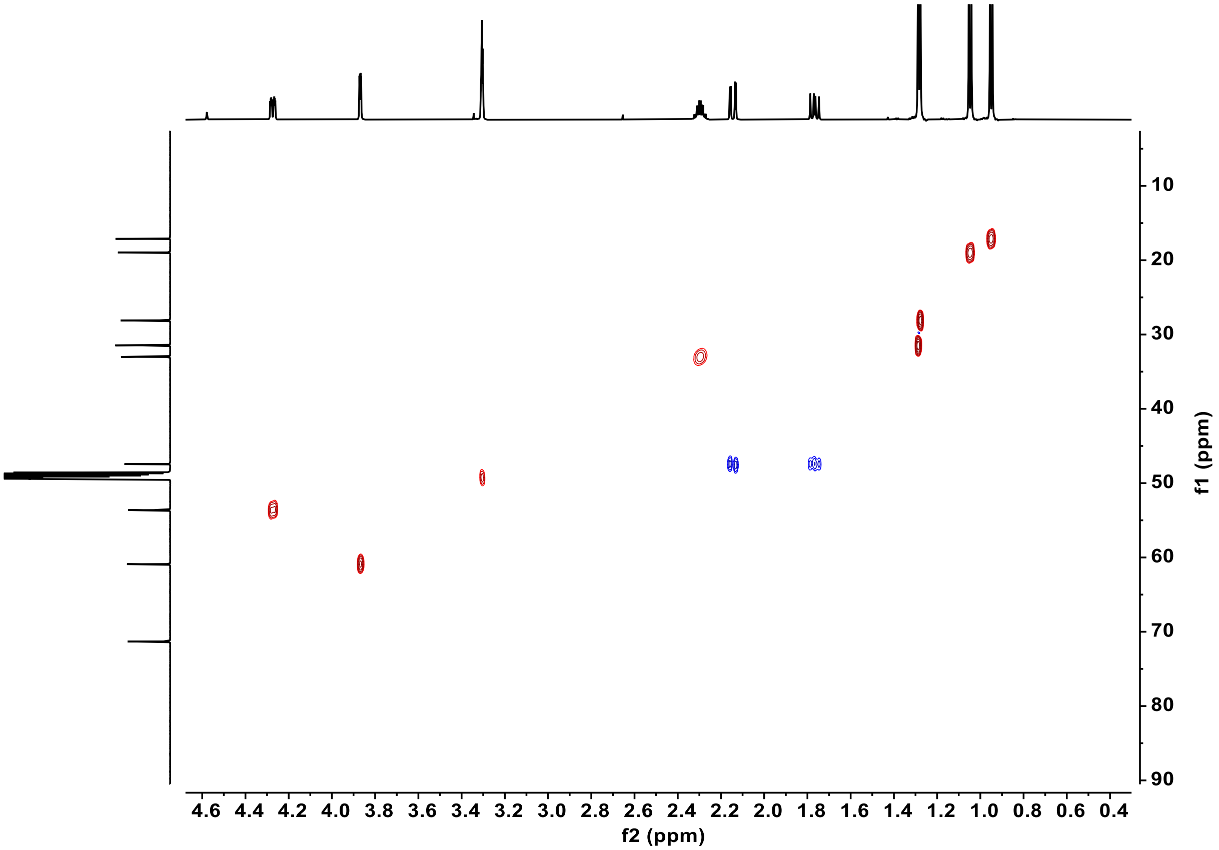

Supplementary Fig. 121. HSQC spectrum of **9a** (600 MHz, CD_3OD).

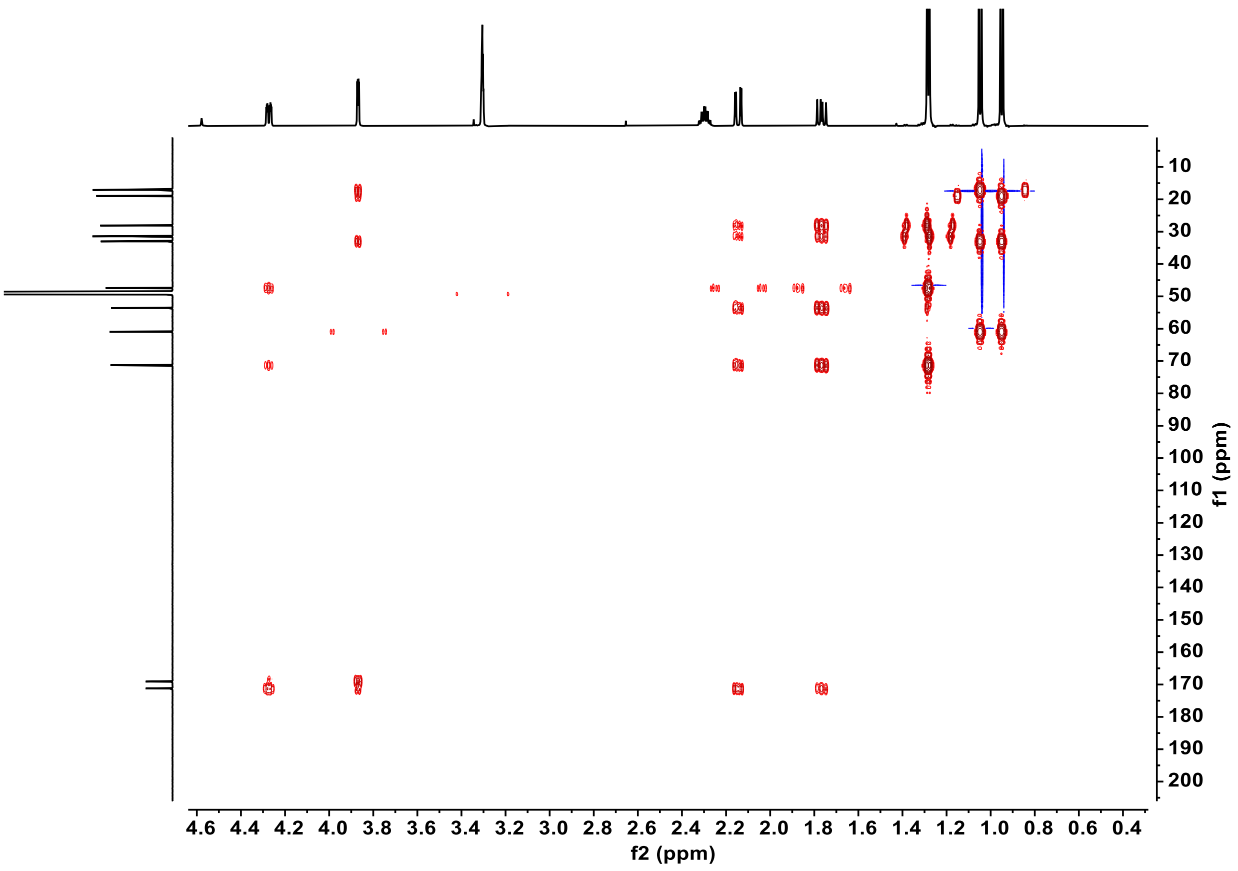

Supplementary Fig. 122. HMBC spectrum of **9a** (600 MHz, CD_3OD).

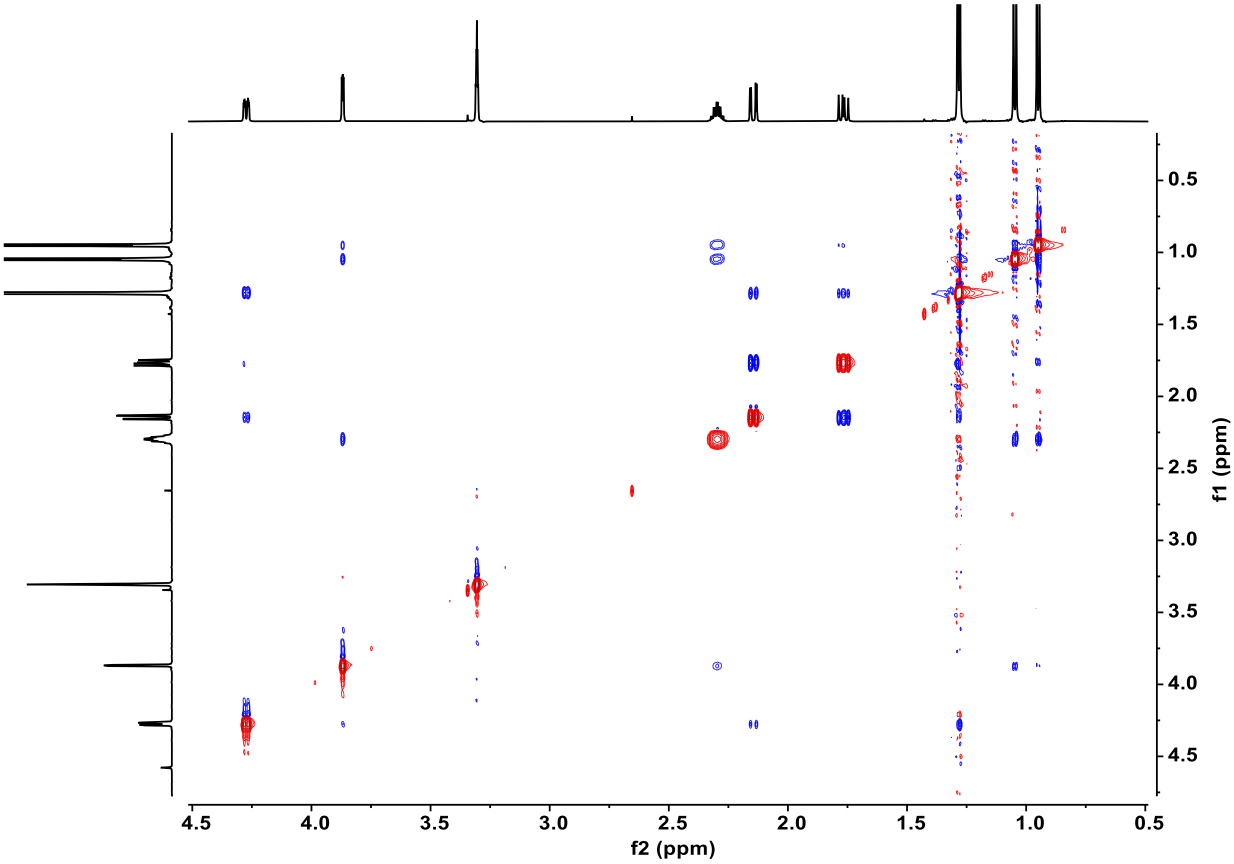

Supplementary Fig. 123. ^{1}H-^{1}H NOESY spectrum of **9a** (600 MHz, CD_3OD).

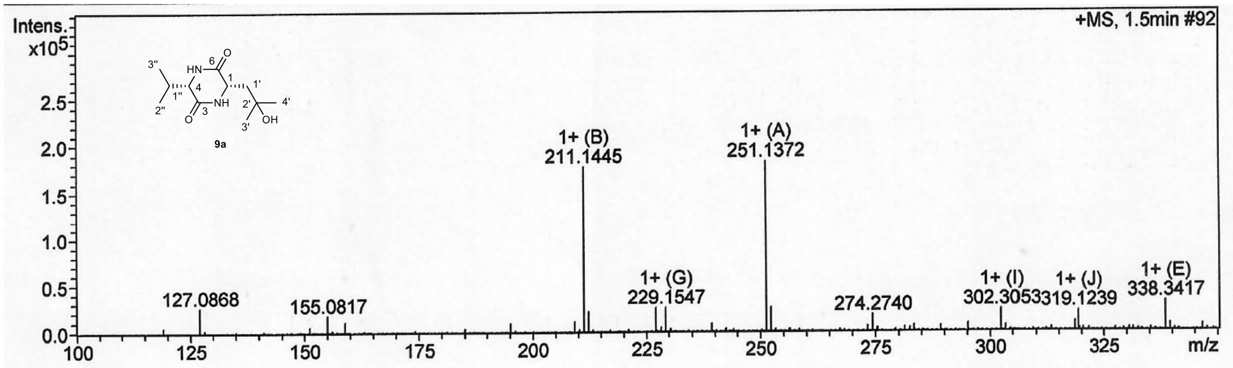

Meas. m/z	#	Formula	Score	m/z	err [ppm]	Mean err [ppm]	mSigma	rdb	e⁻ Conf	N-Rule
229.1547	1	C 11 H 21 N 2 O 3	100.00	229.1547	-0.2	-0.0	4.2	2.5	even	ok
251.1372	1	C 11 H 20 N 2 Na O 3	100.00	251.1366	-2.3	-1.9	8.7	2.5	even	ok

Supplementary Fig. 124. HR-ESI-MS (positive) spectrum of **9a**.

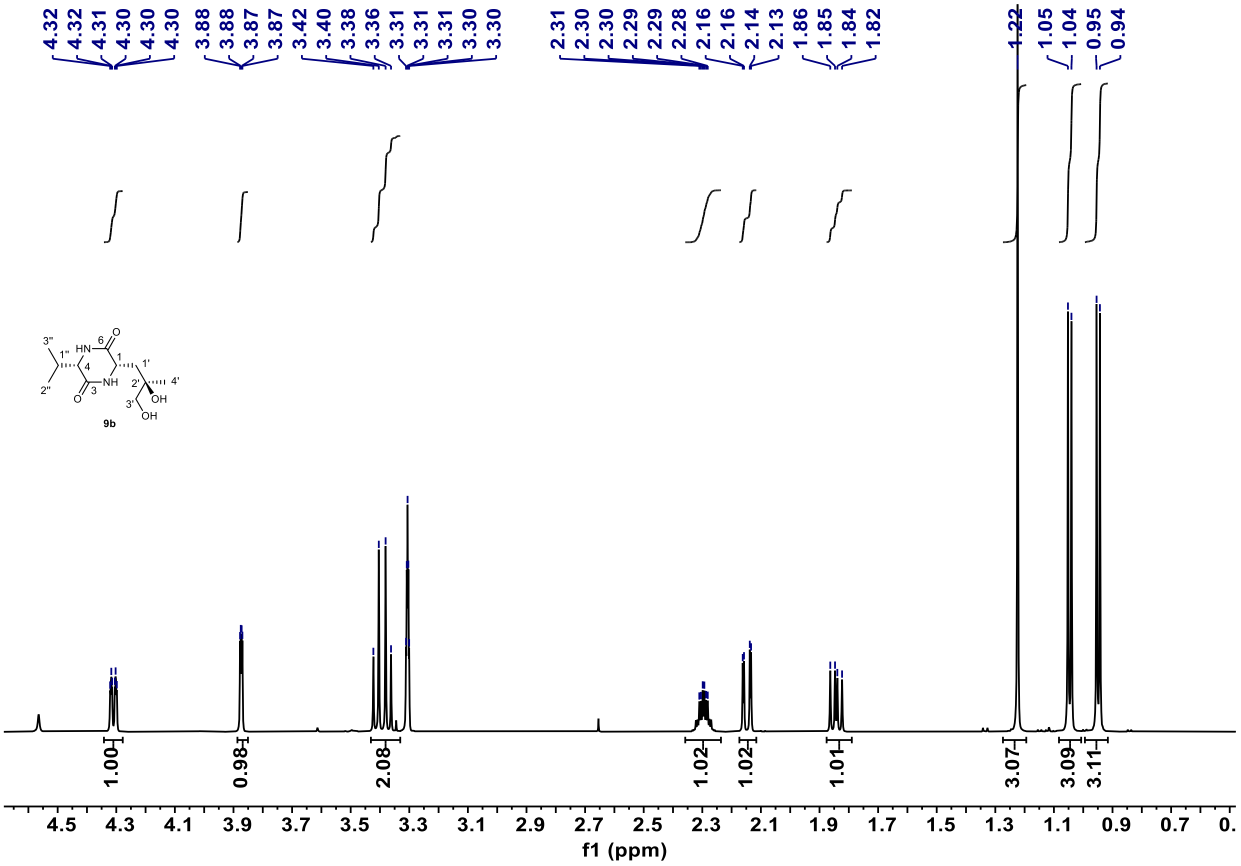

Supplementary Fig. 125. ^{1}H NMR spectrum of **9b** (600 MHz, CD_3OD).

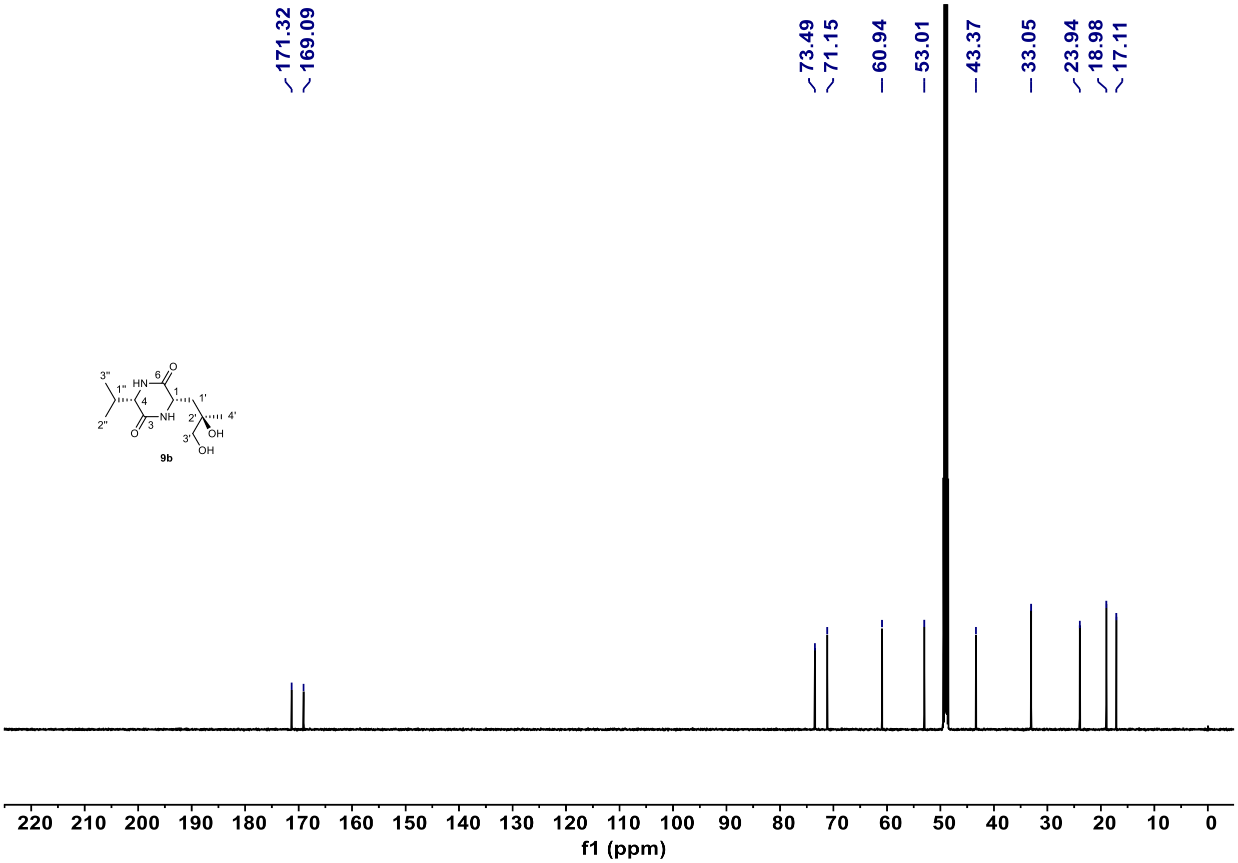

Supplementary Fig. 126. ^{13}C NMR spectrum of **9b** (150 MHz, CD_3OD).

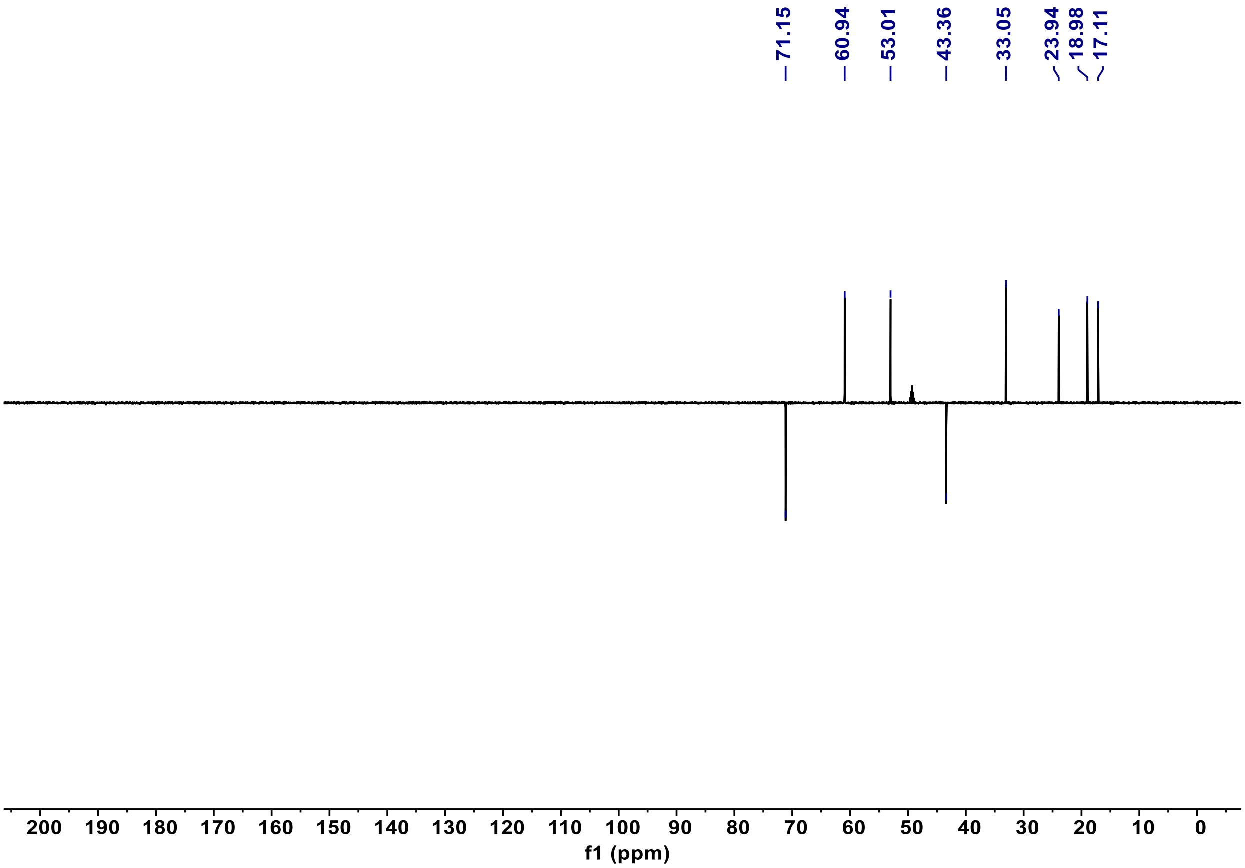

Supplementary Fig. 127. DEPT135 spectrum of **9b** (150 MHz, CD_3OD).

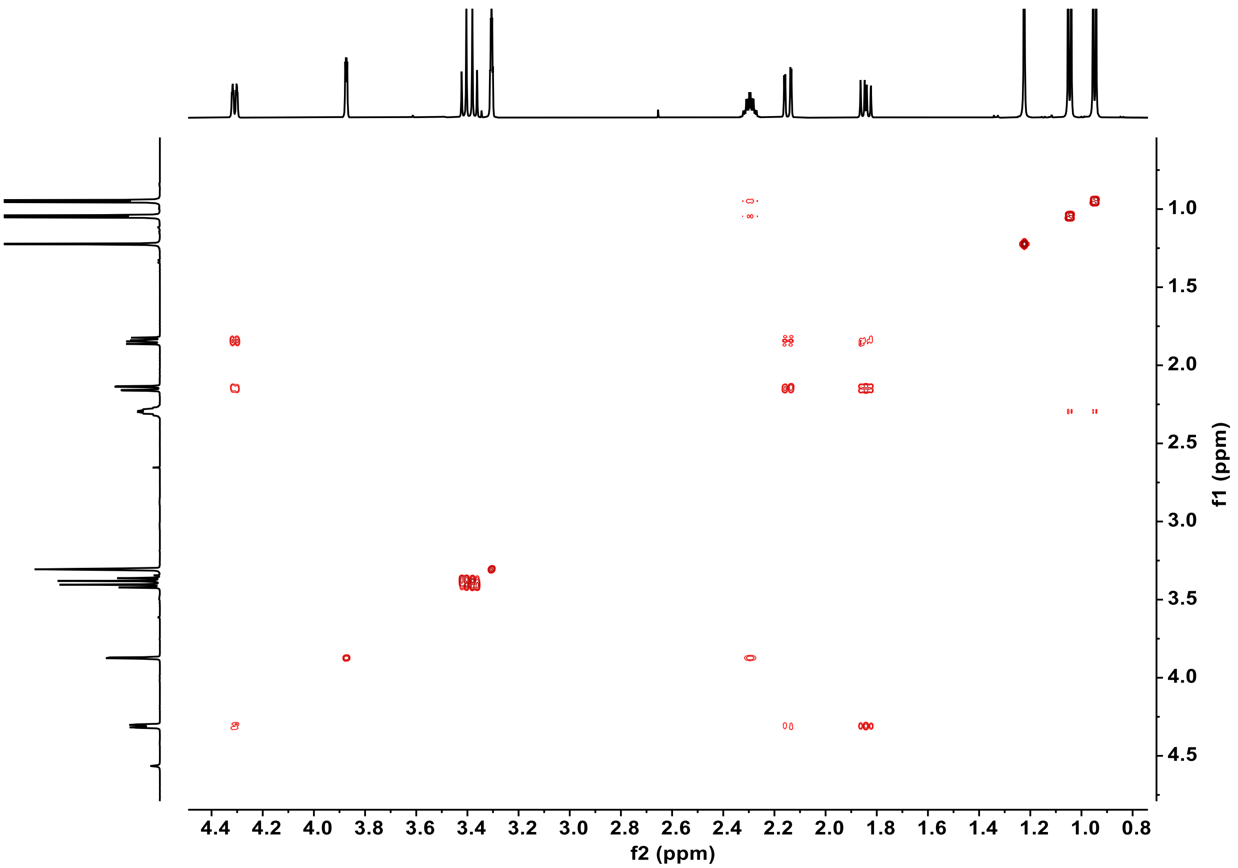

Supplementary Fig. 128. ^{1}H-^{1}H COSY spectrum of **9b** (600 MHz, CD_3OD).

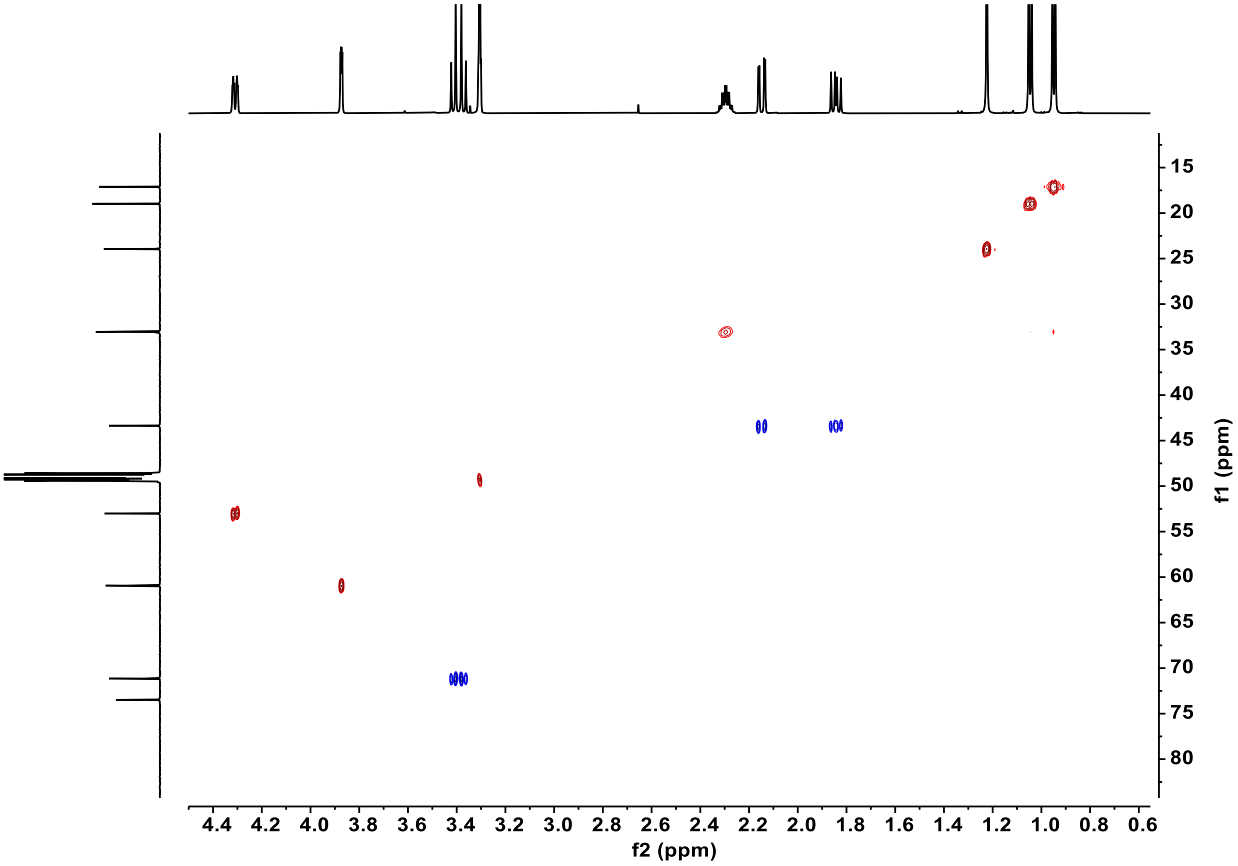

Supplementary Fig. 129. HSQC spectrum of **9b** (600 MHz, CD_3OD).

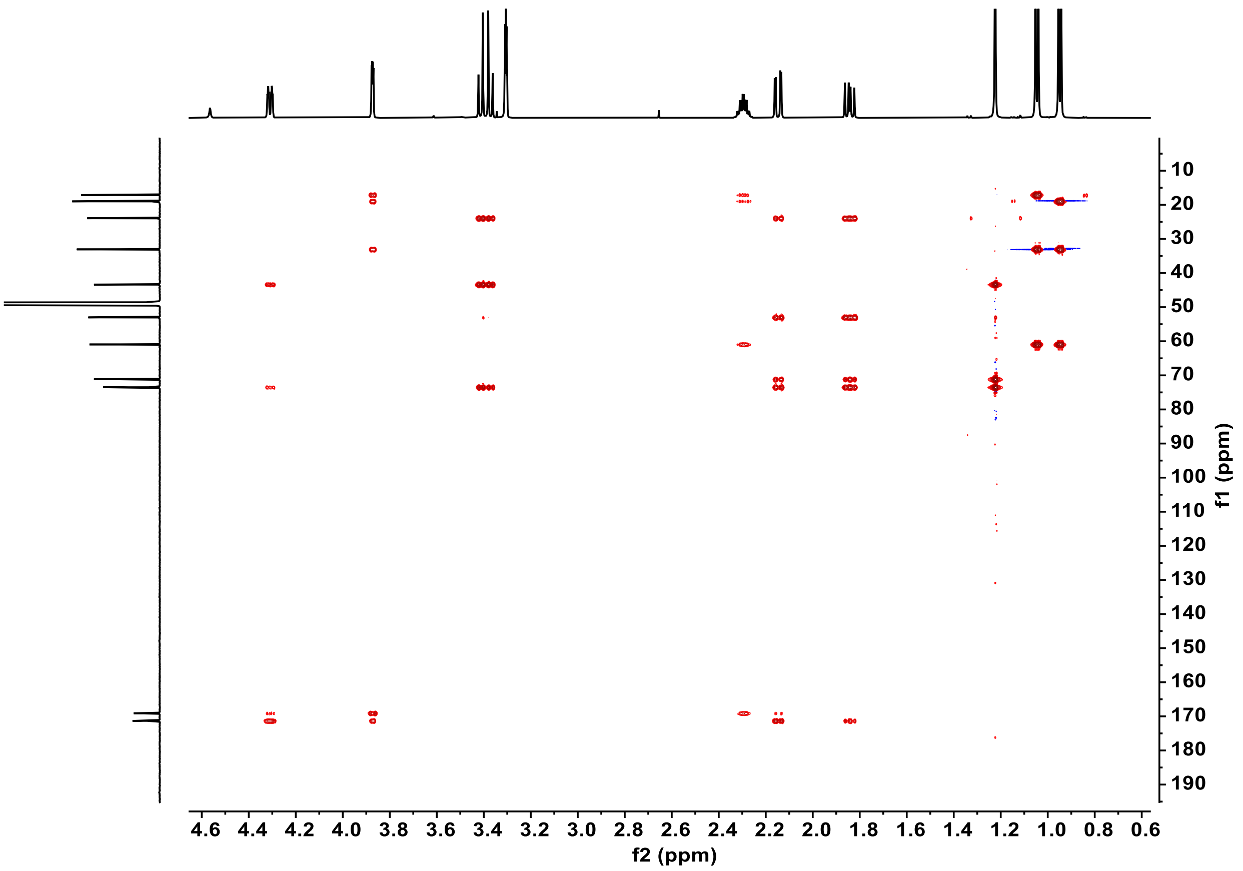

Supplementary Fig. 130. HMBC spectrum of **9b** (600 MHz, CD_3OD).

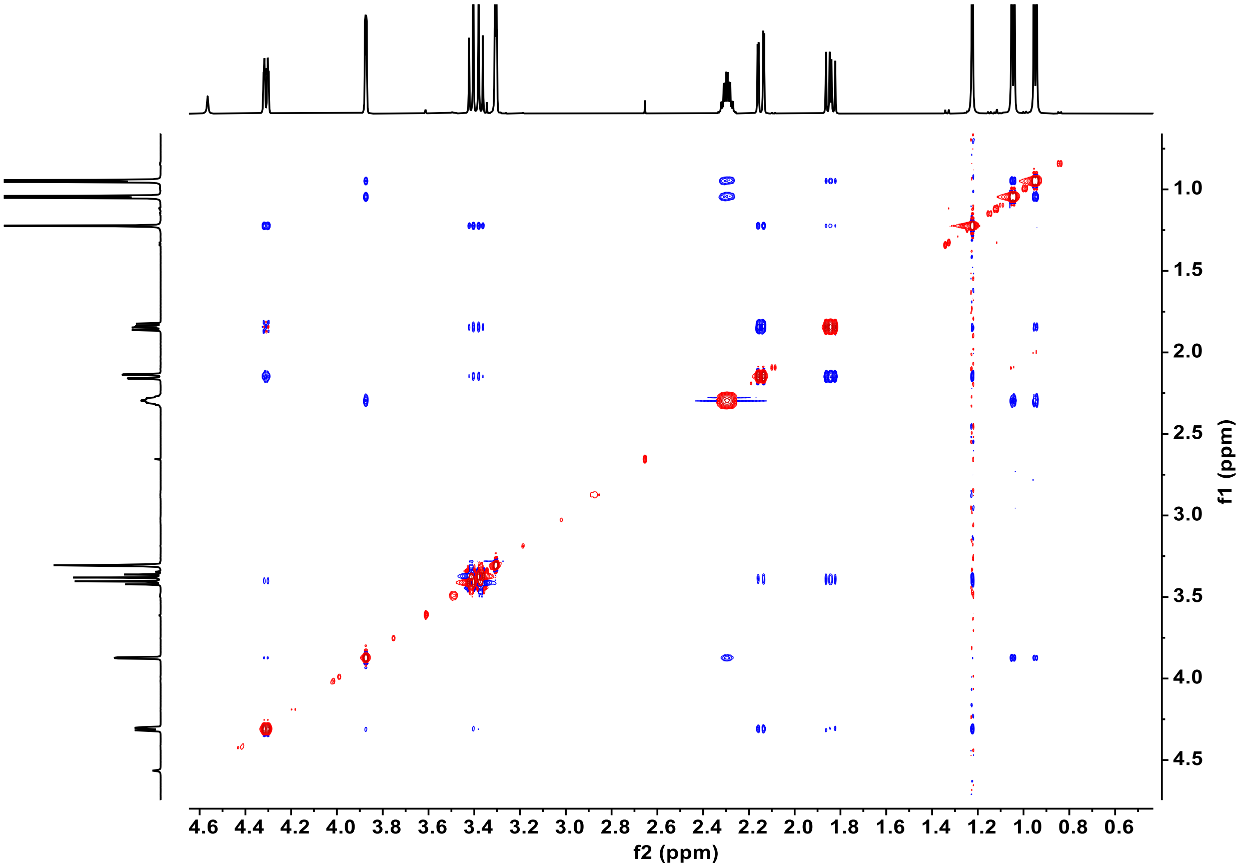

Supplementary Fig. 131. ^{1}H-^{1}H NOESY spectrum of **9b** (600 MHz, CD_3OD).

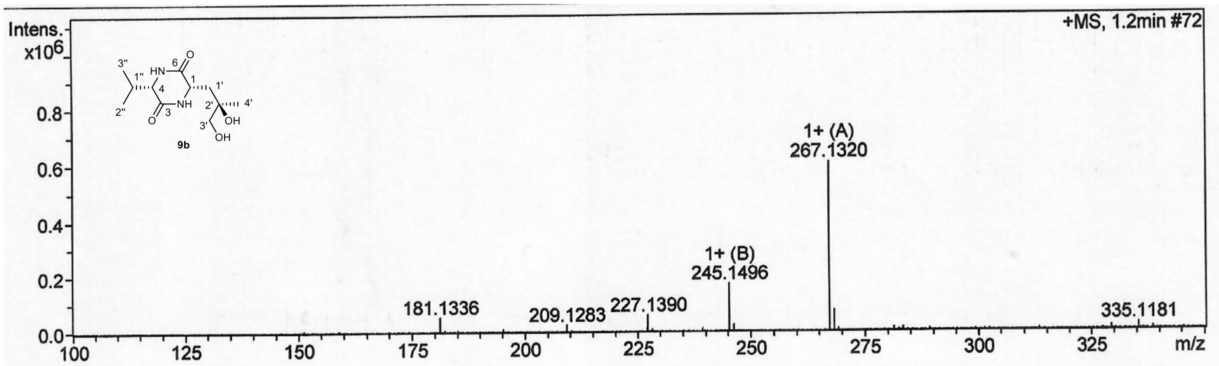

Meas. m/z	#	Formula	Score	m/z	err [ppm]	Mean err [ppm]	mSigma	rdb	e¯ Conf	N-Rule
245.1496	1	C 11 H 21 N 2 O 4	100.00	245.1496	-0.2	-0.4	0.5	2.5	even	ok
267.1320	1	C 11 H 20 N 2 Na O 4	100.00	267.1315	-1.6	-1.6	2.6	2.5	even	ok

Supplementary Fig. 132. HR-ESI-MS (positive) spectrum of **9b**.

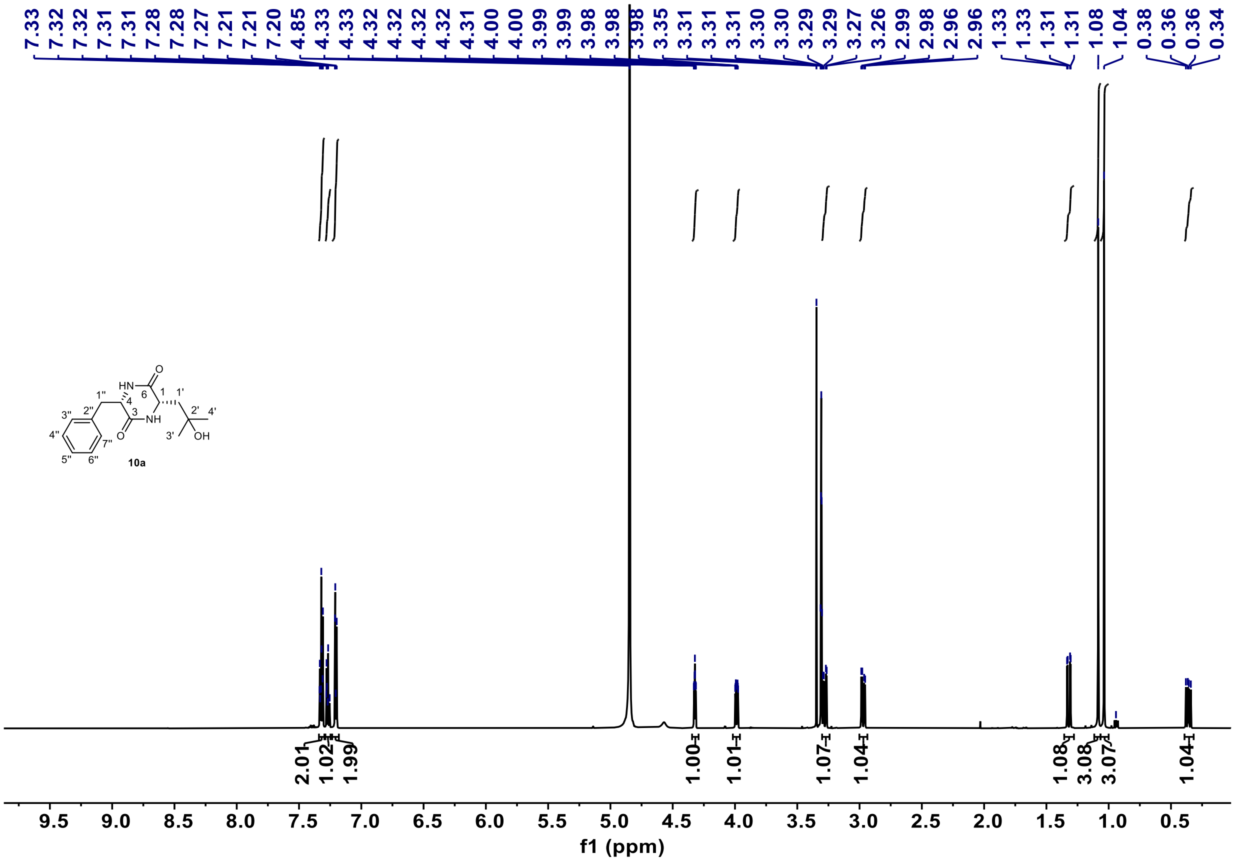

Supplementary Fig. 133. ^{1}H NMR spectrum of **10a** (600 MHz, CD_3OD).

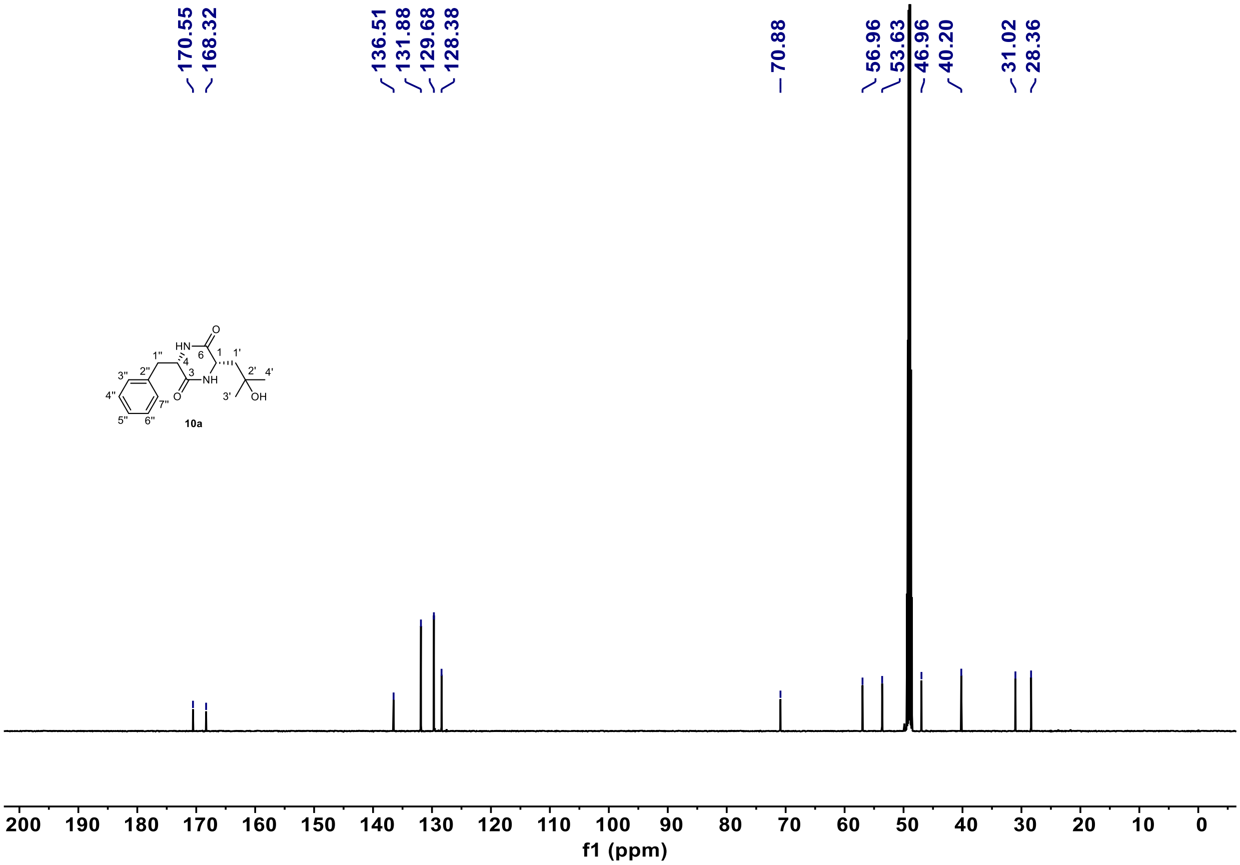

Supplementary Fig. 134. ^{13}C NMR spectrum of **10a** (150 MHz, CD_3OD).

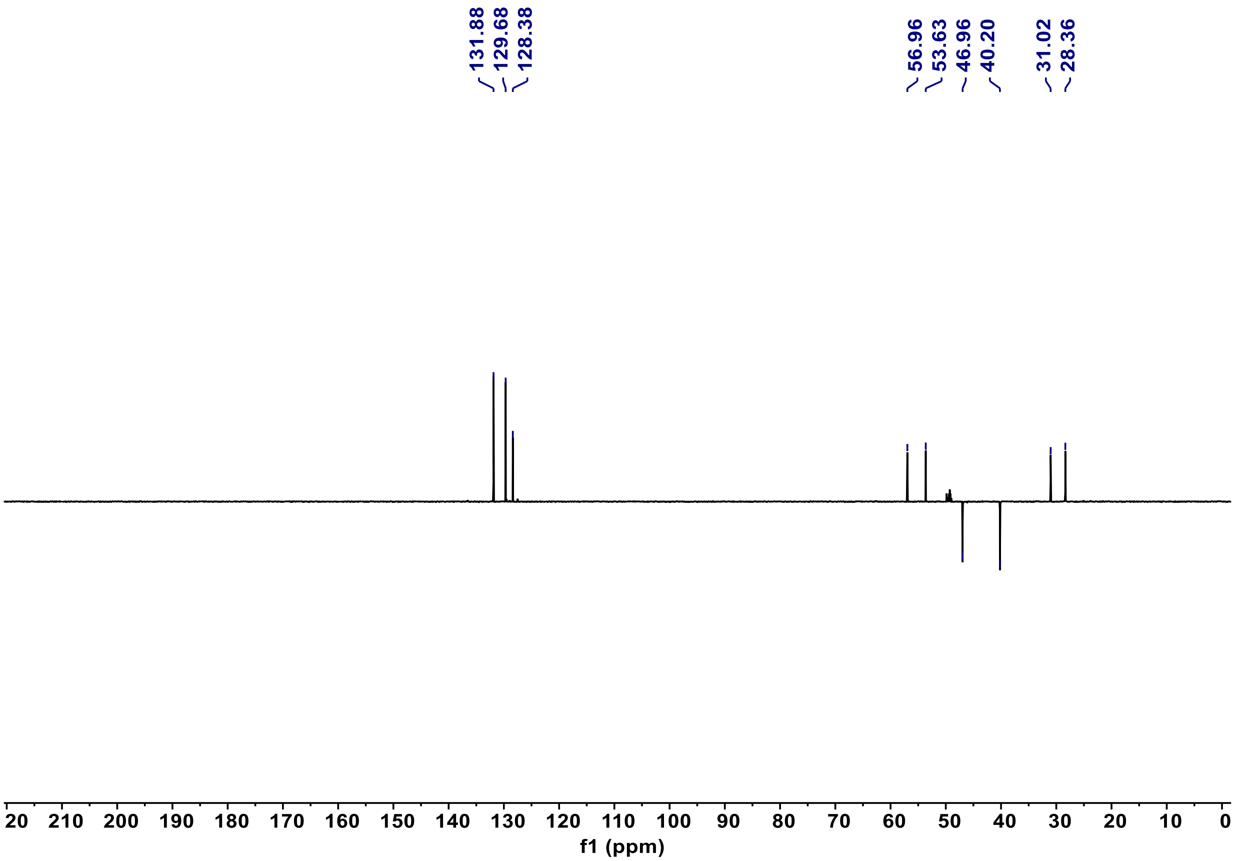

Supplementary Fig. 135. DEPT135 spectrum of **10a** (150 MHz, CD_3OD).

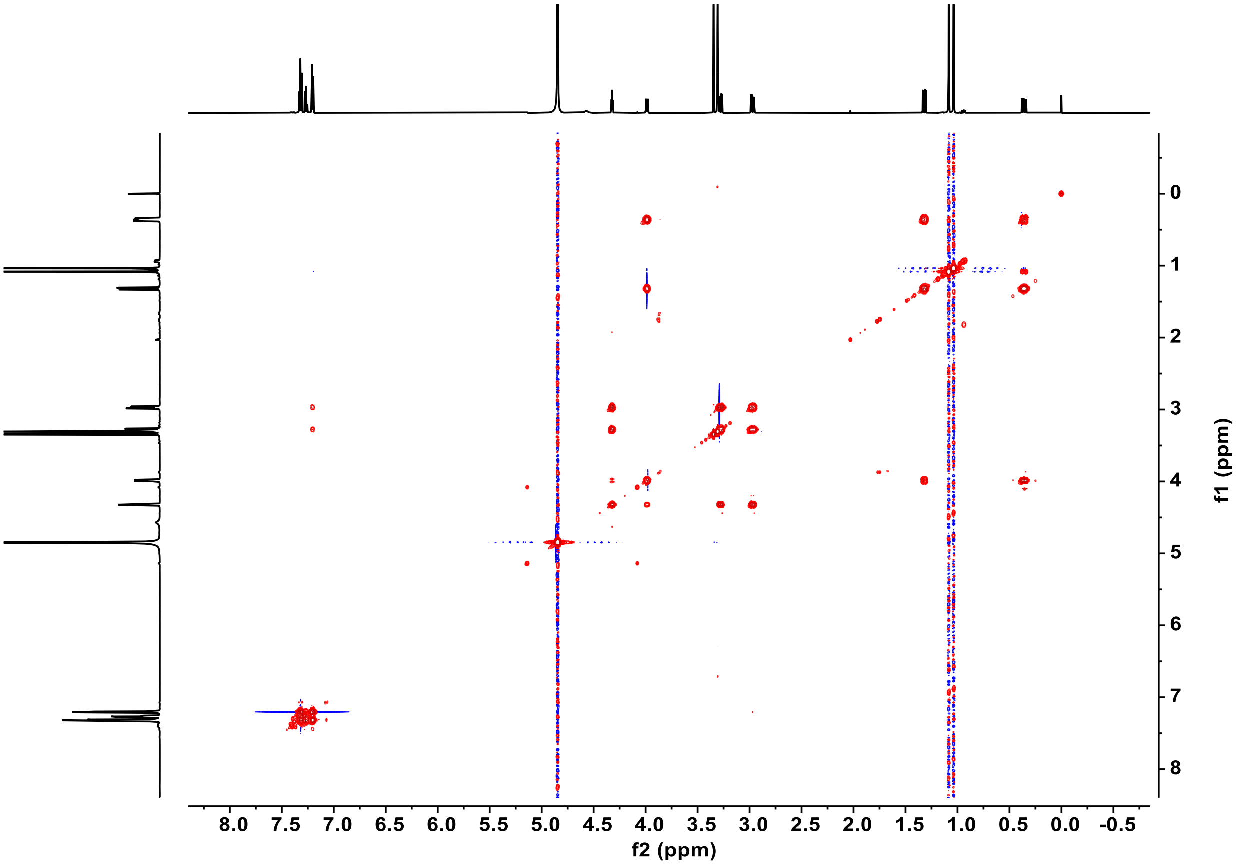

Supplementary Fig. 136. ^{1}H-^{1}H COSY spectrum of **10a** (600 MHz, CD_3OD).

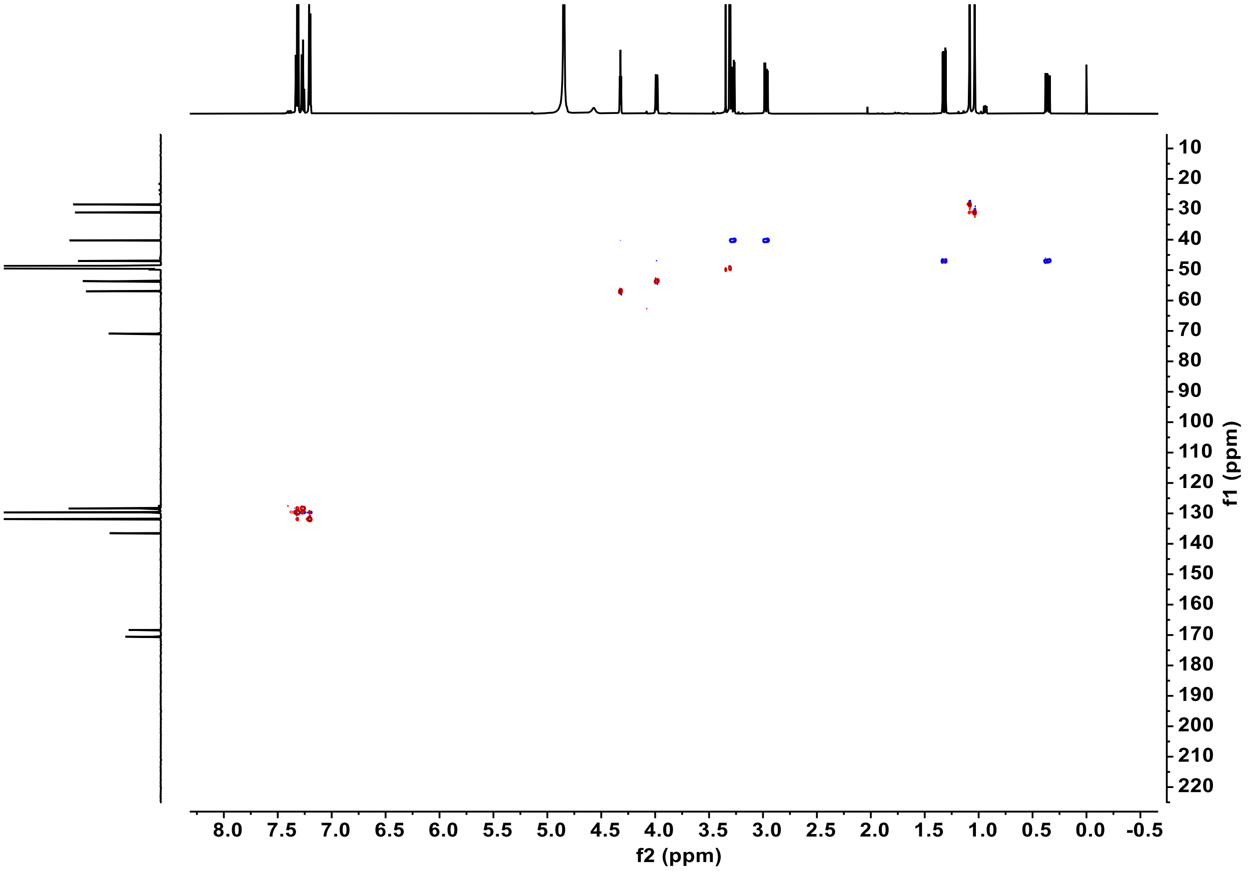

Supplementary Fig. 137. HSQC spectrum of **9b** (600 MHz, CD_3OD).

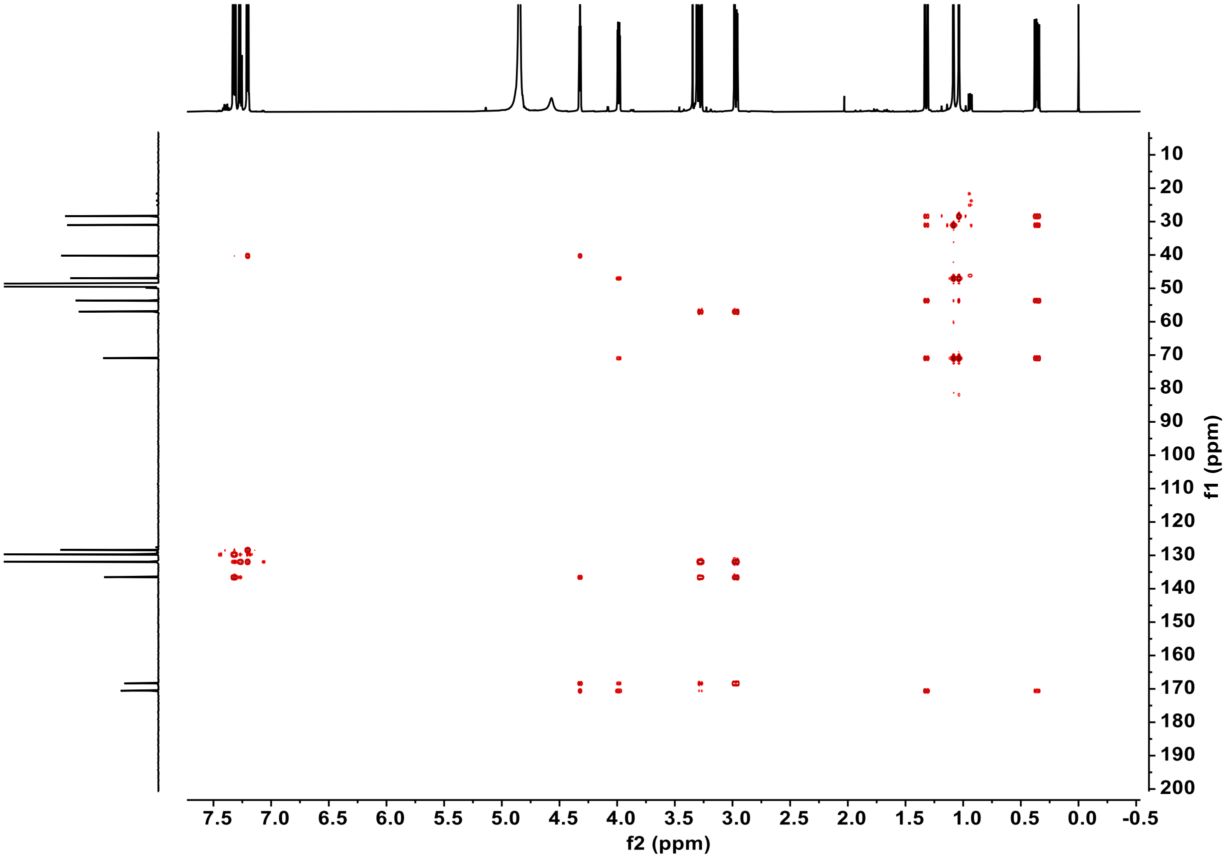

Supplementary Fig. 138. HMBC spectrum of **9b** (600 MHz, CD_3OD).

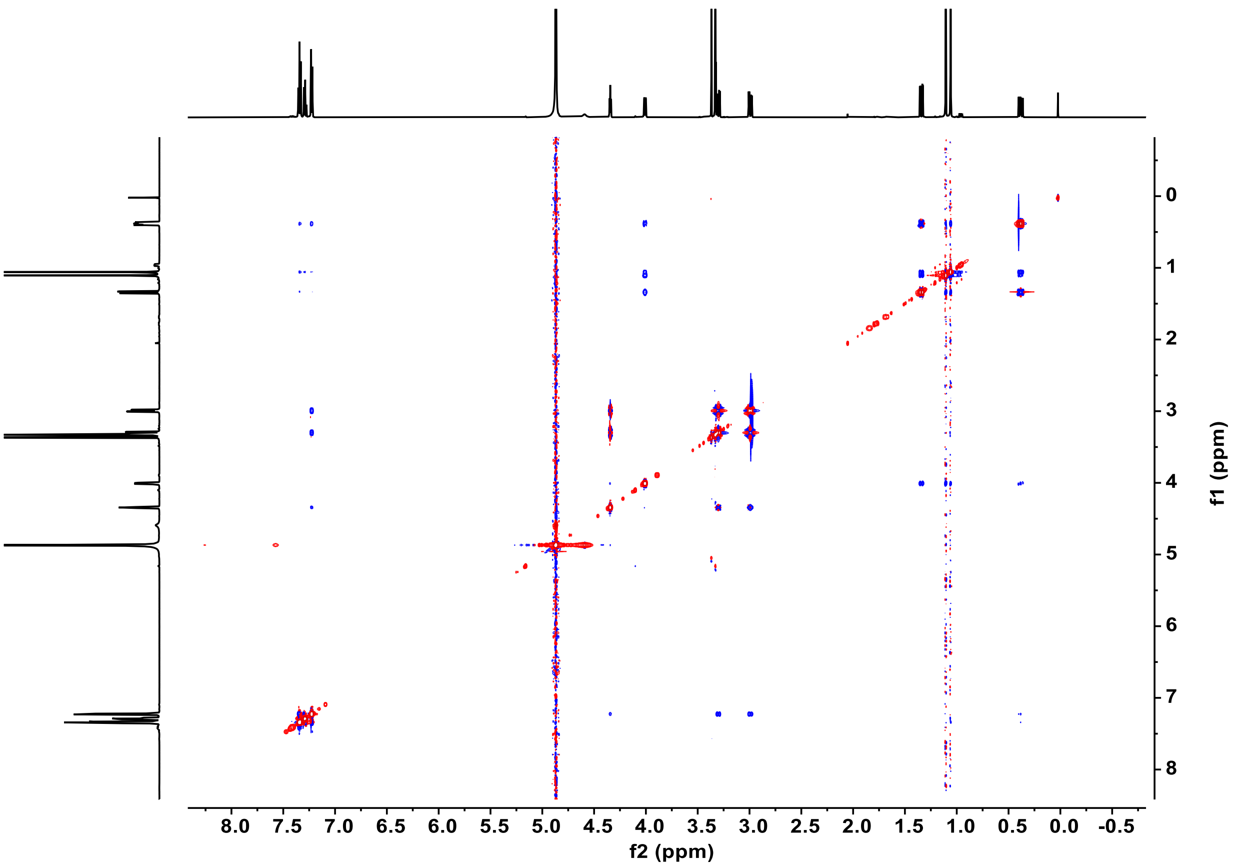

Supplementary Fig. 139. ^{1}H-^{1}H NOESY spectrum of **10a** (600 MHz, CD_3OD).

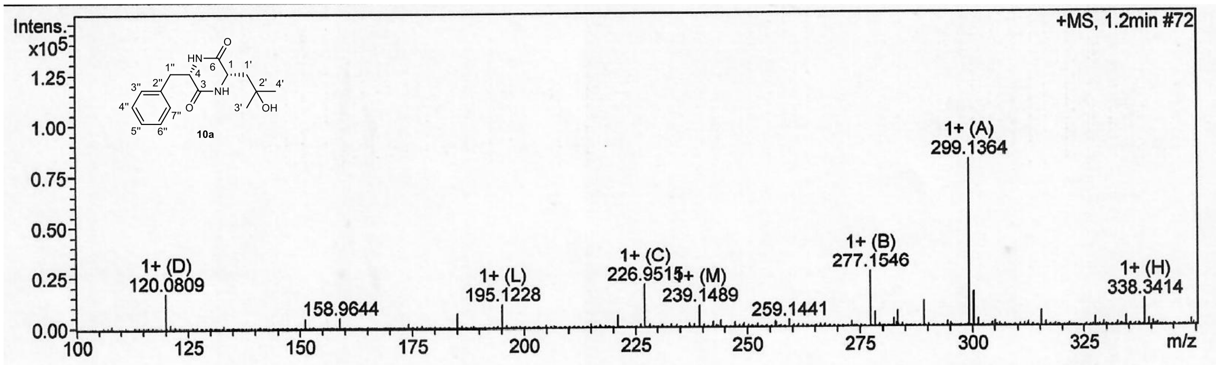

Meas. m/z	#	Formula	Score	m/z	err [ppm]	Mean err [ppm]	mSigma	rdb	e^- Conf	N-Rule
277.1546	1	C 15 H 21 N 2 O 3	100.00	277.1547	0.2	-0.4	42.3	6.5	even	ok
299.1364	1	C 15 H 20 N 2 Na O 3	100.00	299.1366	0.7	0.4	22.2	6.5	even	ok

Supplementary Fig. 140. HR-ESI-MS (positive) spectrum of **10a**.

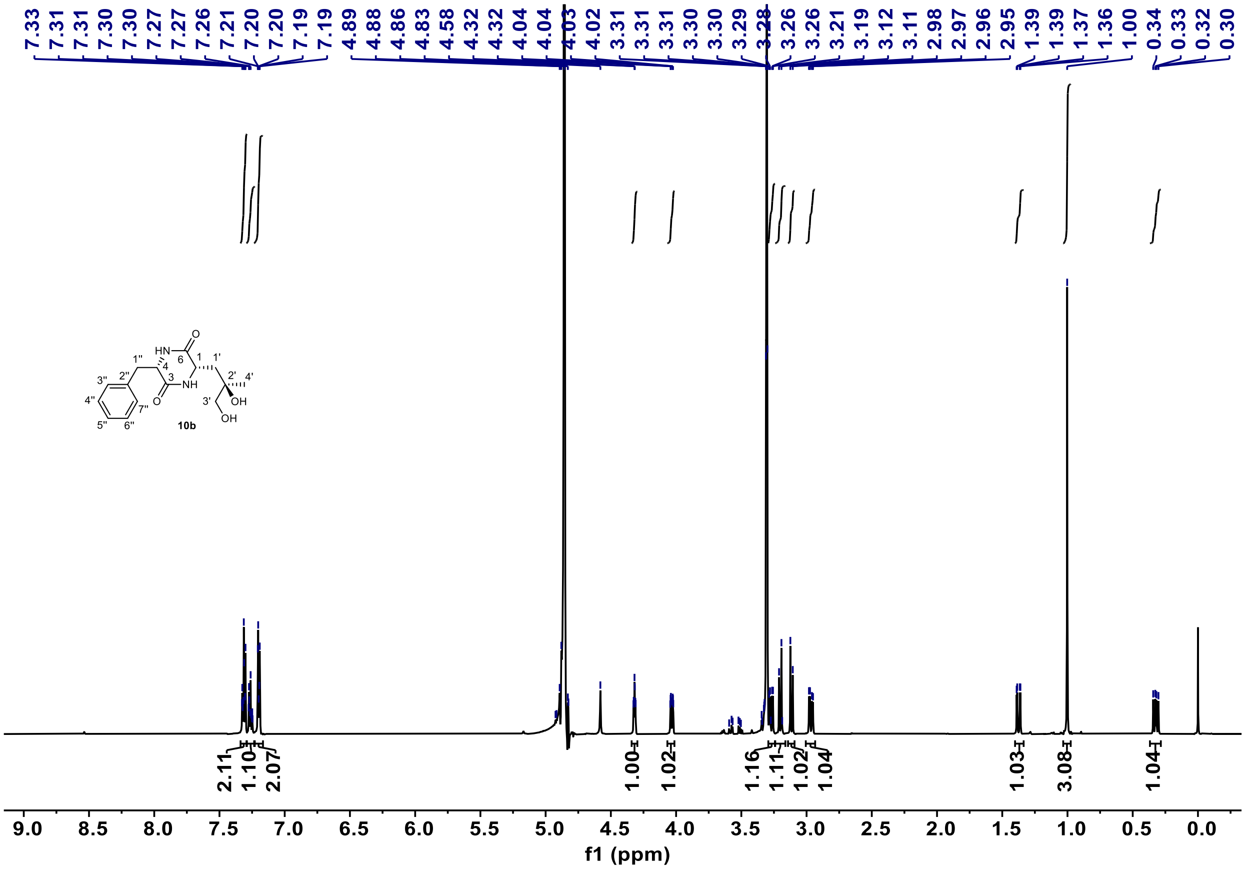

Supplementary Fig. 141. ^{1}H NMR spectrum of **10b** (600 MHz, CD_3OD).

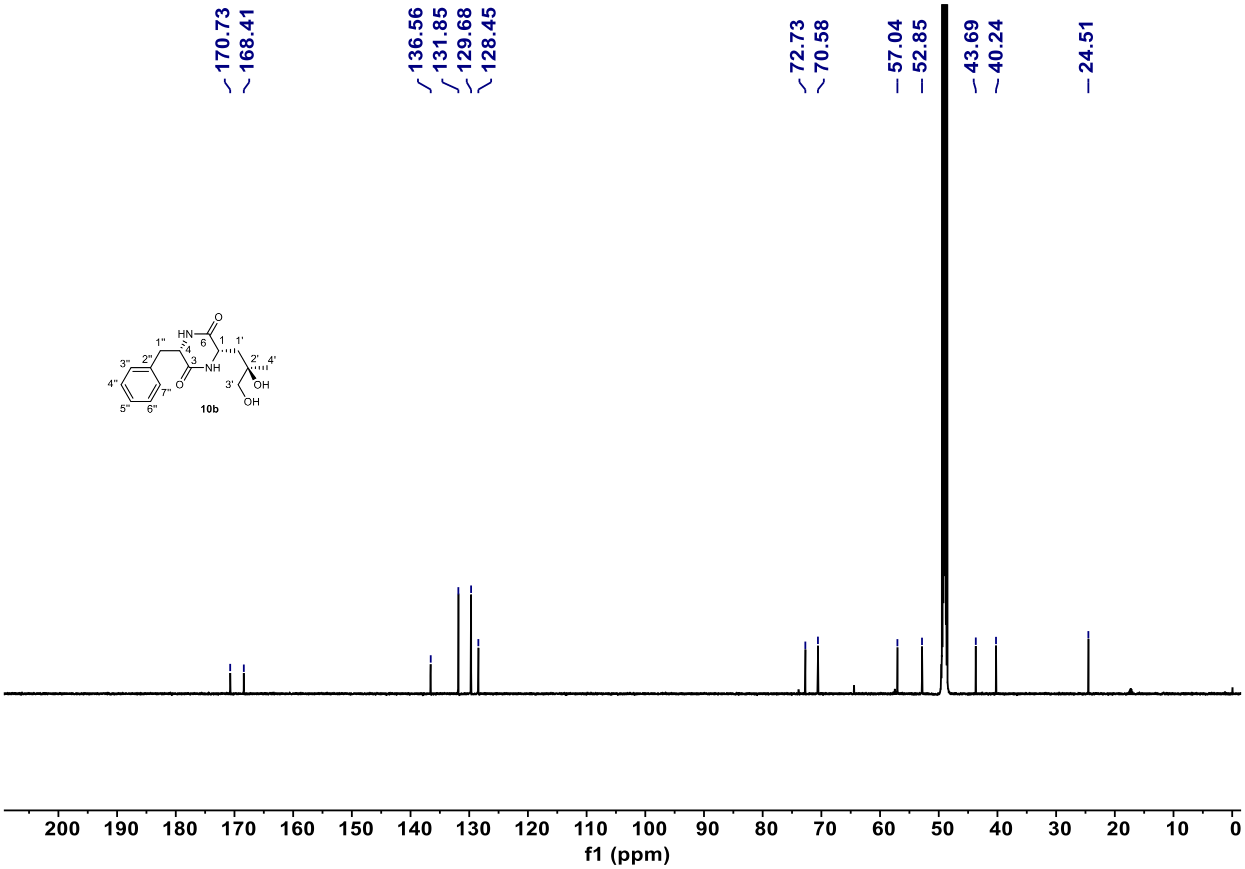

Supplementary Fig. 142. ^{13}C NMR spectrum of **10b** (150 MHz, CD_3OD).

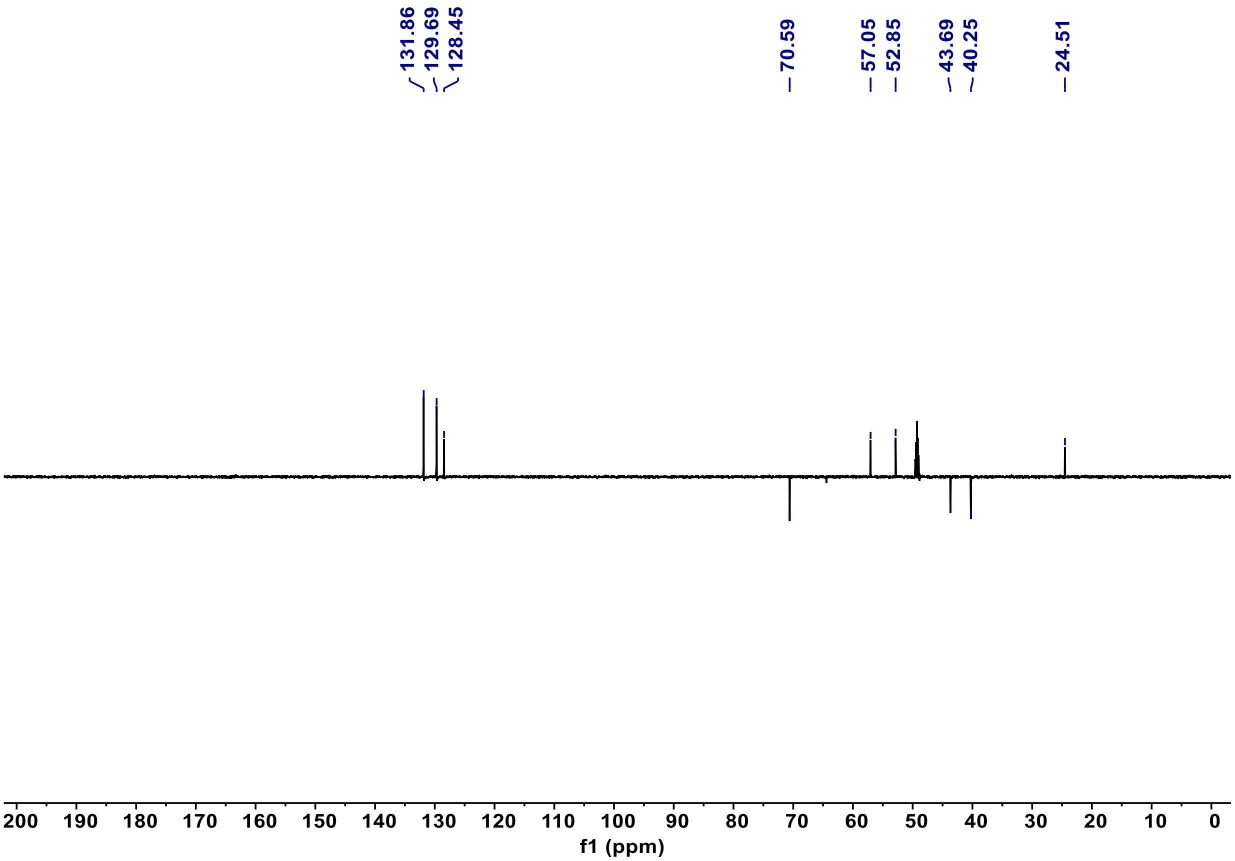

Supplementary Fig. 143. DEPT135 spectrum of **10b** (150 MHz, CD_3OD).

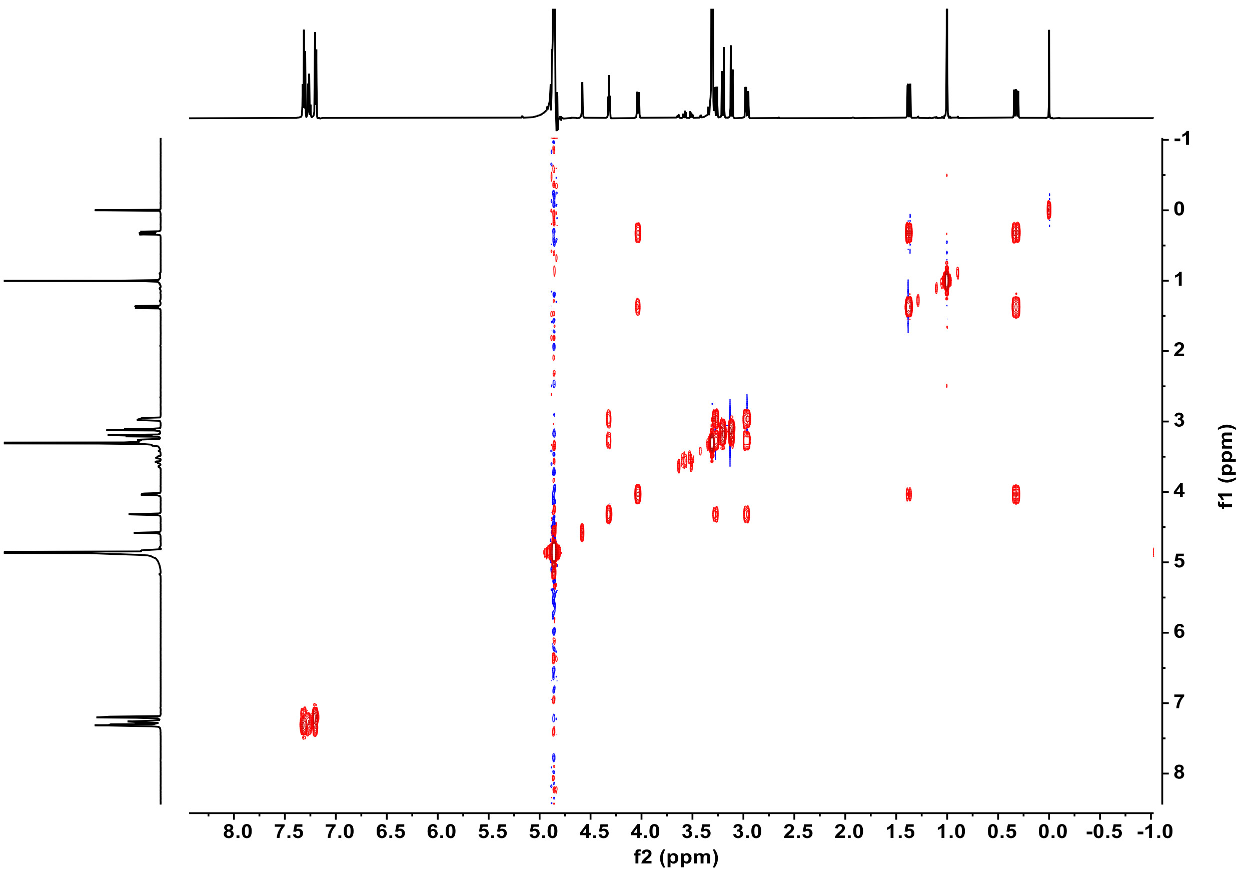

Supplementary Fig. 144. ^{1}H-^{1}H COSY spectrum of **10b** (600 MHz, CD_3OD).

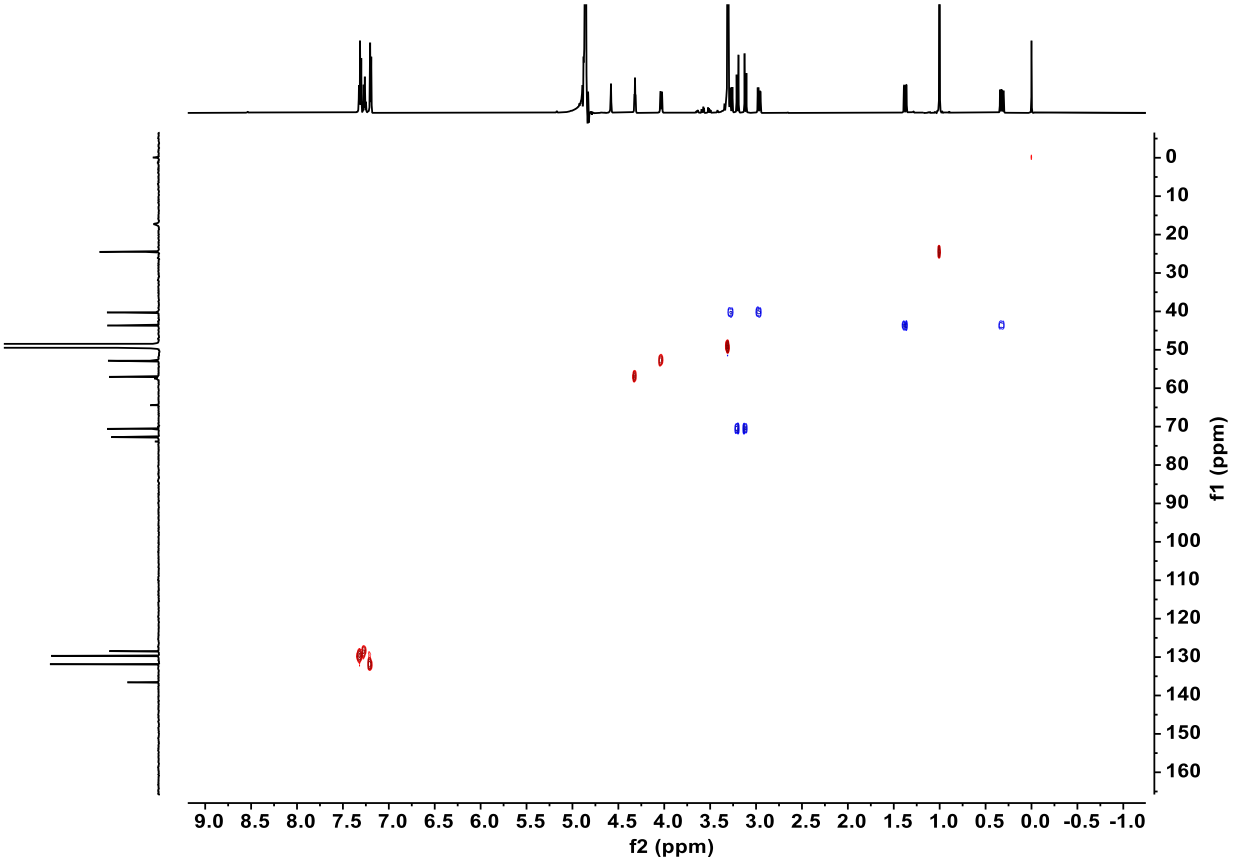

Supplementary Fig. 145. HSQC spectrum of **10b** (600 MHz, CD_3OD).

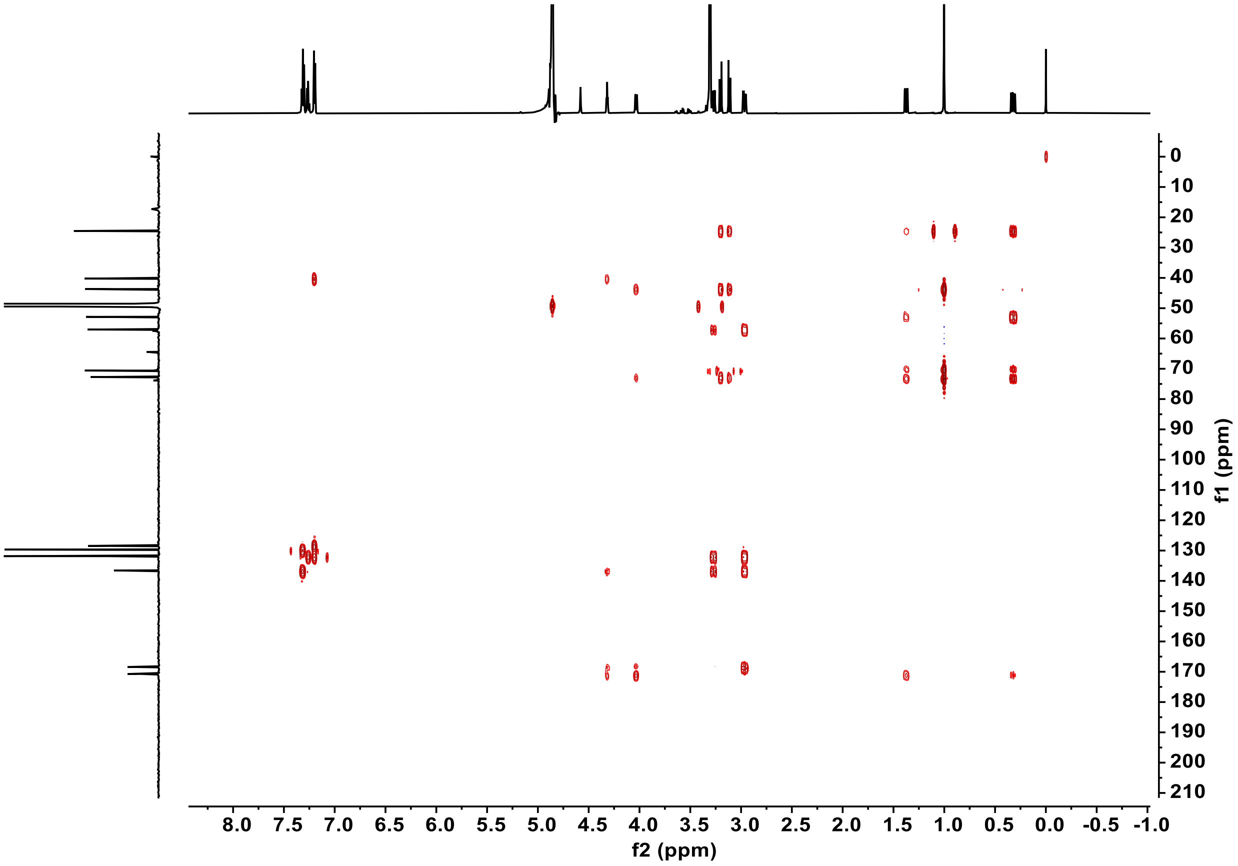

Supplementary Fig. 146. HMBC spectrum of **10b** (600 MHz, CD_3OD).

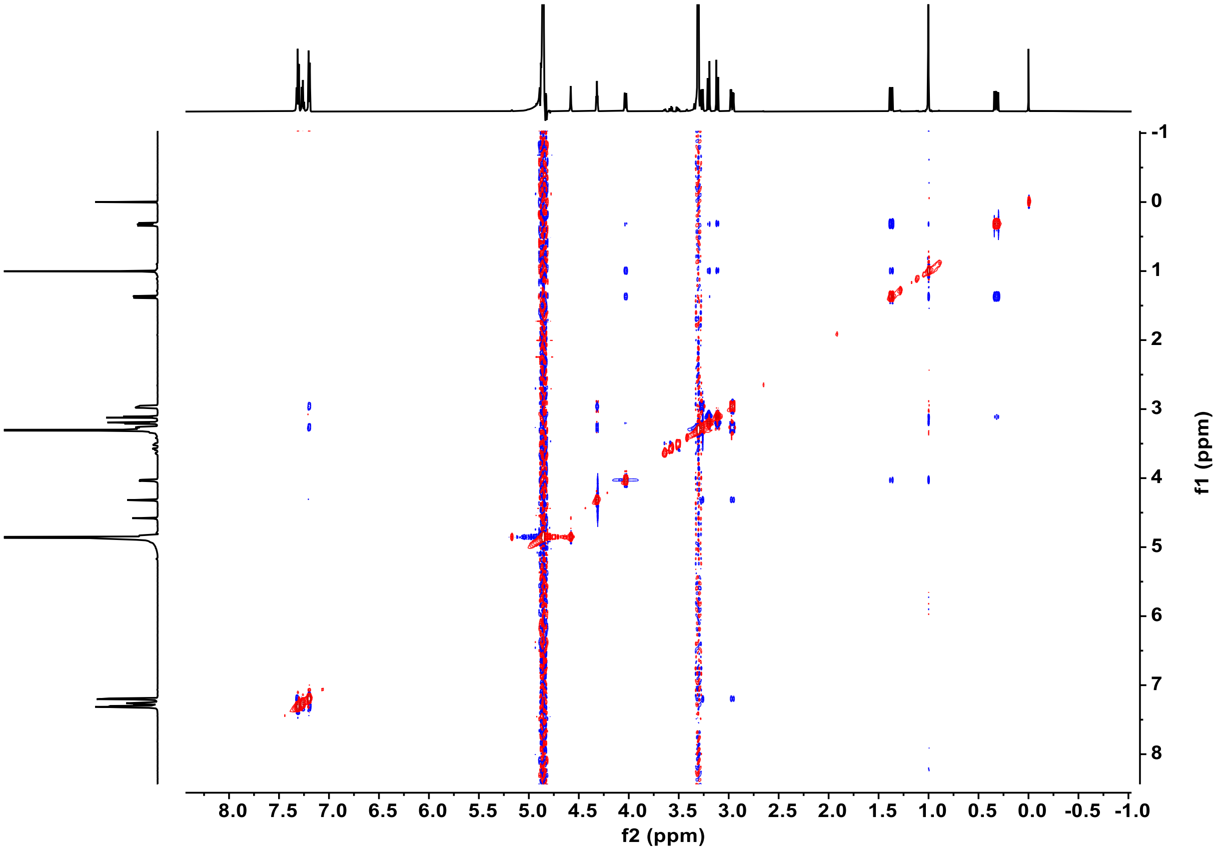

Supplementary Fig. 147. ^{1}H-^{1}H NOESY spectrum of **10b** (600 MHz, CD_3OD).

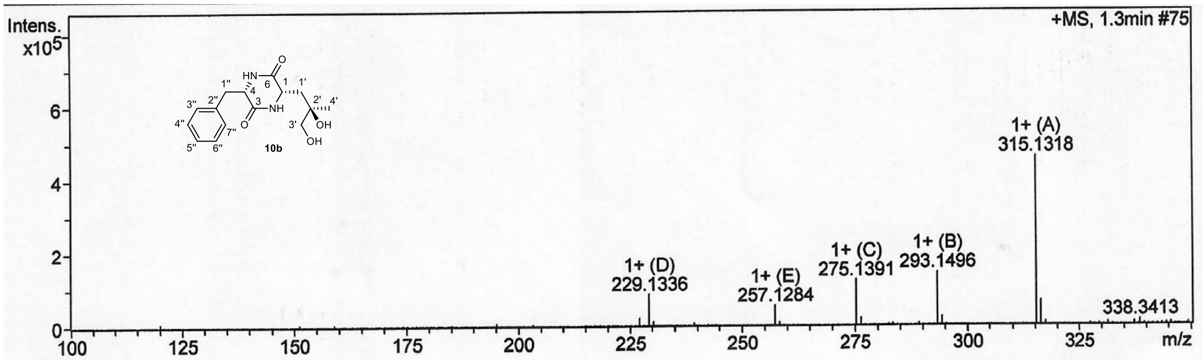

Meas. m/z	#	Formula	Score	m/z	err [ppm]	Mean err [ppm]	mSigma	rdb	e⁻ Conf	N-Rule
293.1496	1	C 15 H 21 N 2 O 4	100.00	293.1496	0.0	0.3	4.2	6.5	even	ok
315.1318	1	C 15 H 20 N 2 Na O 4	100.00	315.1315	-0.8	-0.5	15.0	6.5	even	ok

Supplementary Fig. 148. HR-ESI-MS (positive) spectrum of **10b.**

References

1 Song, X., Lu, J. & Lai, W. Mechanistic insights into dioxygen activation, oxygen atom exchange and substrate epoxidation by AsqJ dioxygenase from quantum mechanical/molecular mechanical calculations. *Phys. Chem. Chem. Phys.* **19**, 20188-20197 (2017).

2 Gaussian 16 Rev. A.03 (Wallingford, CT, 2016).

3 Grimme, S., Antony, J., Ehrlich, S. & Krieg, H. A consistent and accurate ab initio parametrization of density functional dispersion correction (DFT-D) for the 94 elements H-Pu. *J. Chem. Phys.* **132**, 154104 (2010).

4 Hay, P. J. & Wadt, W. R. Ab initio effective core potentials for molecular calculations. Potentials for K to Au including the outermost core orbitals. *J. Chem. Phys.* **82**, 299-310, doi:10.1063/1.448975 (1985).

5 Chaturvedi, S. S. et al. Catalysis by the Non-Heme Iron(II) Histone Demethylase PHF8 Involves Iron Center Rearrangement and Conformational Modulation of Substrate Orientation. *ACS Catal.* **10**, 1195-1209 (2020).

6 Yeh, C. C. G. et al. Cluster Model Study into the Catalytic Mechanism of α-Ketoglutarate Biodegradation by the Ethylene-Forming Enzyme Reveals Structural Differences with Nonheme Iron Hydroxylases. *ACS Catal.* **12**, 3923-3937 (2022).

7 Aboelnga, M. M. & Gauld, J. W. Establishing a substrate-assisted mechanism for the pre-transfer editing in SerRS and IleRS: a QM/QM investigation. *Struct. Chem.* **35**, 519-530 (2024).
