# Supplementary material for: Three distinct strategies lead to programmable aliphatic C−H oxidation in bicyclomycin biosynthesis
Source: Nat Commun. 2025 May 19;16:4651. doi: 10.1038/s41467-025-58997-8 (PMC12089406; doi:10.1038/s41467-025-58997-8)
Supplement: Supplementary file 2 — Reporting Summary [file 41467_2025_58997_MOESM2_ESM.pdf]

Corresponding author(s): Sheng Yang, Yong Liang, Gong-Li Tang, Jiahai Zhou

Last updated by author(s): Apr 8, 2025

Reporting Summary

Nature Portfolio wishes to improve the reproducibility of the work that we publish. This form provides structure for consistency and transparency in reporting. For further information on Nature Portfolio policies, see our Editorial Policies and the Editorial Policy Checklist.

Statistics

For all statistical analyses, confirm that the following items are present in the figure legend, table legend, main text, or Methods section.

n/a	Confirmed	
☐	☒	The exact sample size (*n*) for each experimental group/condition, given as a discrete number and unit of measurement
☐	☒	A statement on whether measurements were taken from distinct samples or whether the same sample was measured repeatedly
☒	☐	The statistical test(s) used AND whether they are one- or two-sided<br>*Only common tests should be described solely by name; describe more complex techniques in the Methods section.*
☒	☐	A description of all covariates tested
☒	☐	A description of any assumptions or corrections, such as tests of normality and adjustment for multiple comparisons
☐	☒	A full description of the statistical parameters including central tendency (e.g. means) or other basic estimates (e.g. regression coefficient) AND variation (e.g. standard deviation) or associated estimates of uncertainty (e.g. confidence intervals)
☒	☐	For null hypothesis testing, the test statistic (e.g. *F*, *t*, *r*) with confidence intervals, effect sizes, degrees of freedom and *P* value noted<br>*Give P values as exact values whenever suitable.*
☒	☐	For Bayesian analysis, information on the choice of priors and Markov chain Monte Carlo settings
☒	☐	For hierarchical and complex designs, identification of the appropriate level for tests and full reporting of outcomes
☒	☐	Estimates of effect sizes (e.g. Cohen's *d*, Pearson's *r*), indicating how they were calculated

Our web collection on statistics for biologists contains articles on many of the points above.

Software and code

Policy information about availability of computer code

Data collection	HPLC data were obtained on an Agilent 1260 instrument. For LC-MS data, a Thermo Fisher LTQ Fleet mass spectrometer featuring an electrospray ionization source was utilized. High-resolution (HR) ESI-MS data were collected using both the Bruker maXis4G instrument and the Agilent 6230B accurate mass TOF LC/MS system. NMR spectra were measured on two spectrometers: the Agilent 500/54 Premium Shielded 500 MHz NMR spectrometer and the Bruker AscendTM 600 MHz spectrometer. The diffraction data of the protein crystals were collected at the Shanghai Synchrotron Radiation Facility (SSRF). For SsBcmE•FeII data were collected at the wavelength of 0.9792 Å in beamline BL17U1. Data of SsBcmET307A•FeII•αKG•1, SoBcmC•FeII•αKG•2, SsBcmC•FeII•αKG and PaBcmG•FeII•αKG were collected at the wavelength of 0.9785 Å in beamline BL19U1. Data of PaBcmG•FeII•αKG•3 were collected at the wavelength of 0.9792 Å in beamline BL18U1. Gaussian 16 (revision A.03) was used for NMR spectra calculations, quantum chemical calculations, structure optimization and PESP partial charge calculations. AutoDock 4.2 (revision 1.213) and AutoDockTools-1.5.6 were used for molecular docking. Amber 16 and AmberTools 16 with SHAKE algorithm were used for molecular dynamics simulations.
Data analysis	LC-MS data were analysed in Thermo Xcalibur 2.2.0. NMR data were analysed in MestReNova-9.0.1. NMR calculation data were analysed in Microsoft Excel 2019. The conversion yield data were analysed in Graphpad Prism 8.0.2 and Microsoft Excel 2019. The data of molecular dynamics simulations were plotted in OriginPro 2016 (OriginLab). All chemical structures were drawn using ChemDraw 22.2 software. Data reduction and integration were achieved with HKL3000 and the XDS software package. The phase of SsBcmE•FeII was solved by molecular replacement in the Phenix-Phaser program using 7V3O as initial searching model. The complex structures SsBcmET307A•FeII•αKG•1, SsBcmC•FeII•αKG and PaBcmG•FeII•αKG were determined by molecular replacement using Phaser with SsBcmE•FeII structure as the initial search model. The quaternary complex structure of SoBcmC and PaBcmG were determined by molecular replacement using Phaser with corresponding substrate free crystal structure as the initial search model. Iterative cycles of model building and refinement were performed in Coot (V0.8.9) and Phenix (V1.11.1) , respectively. Structure Figures were drawn using PyMol 2.4.1 (Schrodinger, LLC). For the crystal structure of apo SsBcmE was not intact with some loops and fragments were missing, we built its structure by AlphaFold2 (AF2). The volume of active

pockets was calculated by online sever (http://altair.sci.hokudai.ac.jp/g6/service/pocasa/).The concentration was corrected using the estimated extinction coefficient from the Prot Param tool on the Expasy server (https://web.expasy.org/protparam/).

For manuscripts utilizing custom algorithms or software that are central to the research but not yet described in published literature, software must be made available to editors and reviewers. We strongly encourage code deposition in a community repository (e.g. GitHub). See the Nature Portfolio guidelines for submitting code & software for further information.

Data

Policy information about availability of data

All manuscripts must include a data availability statement. This statement should provide the following information, where applicable:

- Accession codes, unique identifiers, or web links for publicly available datasets
- A description of any restrictions on data availability
- For clinical datasets or third party data, please ensure that the statement adheres to our policy

Atomic coordinates of SsBcmET307A•FeII•αKG•1, SsBcmC•FeII•αKG, SoBcmC•FeII•αKG•2, PaBcmG•FeII•αKG, PaBcmG•FeII•αKG•3, have been deposited in the Protein Data Bank (PDB) under accession codes 8XHY, 8XHP, 8XHQ, 8XHT, 8XHX (https://www.rcsb.org/). The PDB 7V3O could be download from PDB Database (https://doi.org/10.2210/pdb7V3O/pdb). The DNA sequences of genes SsbcmE, SsbcmC, SsbcmG, SobcmC and PabcmG are downloaded from GenBank with accession numbers MG018995, WP_055518629 and HGP0145857 and the codon-optimized DNA sequences are listed in Supplementary Information. Other data supporting the conclusions of this study are presented in the main text, Supplementary Information and from corresponding author(s) upon request.

Research involving human participants, their data, or biological material

Policy information about studies with human participants or human data. See also policy information about sex, gender (identity/presentation), and sexual orientation and race, ethnicity and racism.

Reporting on sex and gender	*Use the terms sex (biological attribute) and gender (shaped by social and cultural circumstances) carefully in order to avoid confusing both terms. Indicate if findings apply to only one sex or gender; describe whether sex and gender were considered in study design; whether sex and/or gender was determined based on self-reporting or assigned and methods used. Provide in the source data disaggregated sex and gender data, where this information has been collected, and if consent has been obtained for sharing of individual-level data; provide overall numbers in this Reporting Summary. Please state if this information has not been collected. Report sex- and gender-based analyses where performed, justify reasons for lack of sex- and gender-based analysis.*
Reporting on race, ethnicity, or other socially relevant groupings	*Please specify the socially constructed or socially relevant categorization variable(s) used in your manuscript and explain why they were used. Please note that such variables should not be used as proxies for other socially constructed/relevant variables (for example, race or ethnicity should not be used as a proxy for socioeconomic status). Provide clear definitions of the relevant terms used, how they were provided (by the participants/respondents, the researchers, or third parties), and the method(s) used to classify people into the different categories (e.g. self-report, census or administrative data, social media data, etc.) Please provide details about how you controlled for confounding variables in your analyses.*
Population characteristics	*Describe the covariate-relevant population characteristics of the human research participants (e.g. age, genotypic information, past and current diagnosis and treatment categories). If you filled out the behavioural & social sciences study design questions and have nothing to add here, write "See above."*
Recruitment	*Describe how participants were recruited. Outline any potential self-selection bias or other biases that may be present and how these are likely to impact results.*
Ethics oversight	*Identify the organization(s) that approved the study protocol.*

Note that full information on the approval of the study protocol must also be provided in the manuscript.

Field-specific reporting

Please select the one below that is the best fit for your research. If you are not sure, read the appropriate sections before making your selection.

☒ Life sciences ☐ Behavioural & social sciences ☐ Ecological, evolutionary & environmental sciences

For a reference copy of the document with all sections, see nature.com/documents/nr-reporting-summary-flat.pdf

Life sciences study design

All studies must disclose on these points even when the disclosure is negative.

Sample size	For enzymatic assays, a sample size of n >= 3 was selected to test the reproducibility of the experiment. For X-ray diffraction data, the sample size was determined by completeness, redundancy, Rmerge and R factor of decay, which shown in statistic table
Data exclusions	For X-ray diffraction data, resolution cutoffs were determined by examing I/sigma, CC1/2, Rmerge. Some reflections were automatically rejected during X-ray data processing and scaling by software HKL3000 or XDS (version Jan. 31, 2020) using Default rejection criteria. Approximately 5% of unique reflections were randomly excluded for calculating the free R value to monitor the quality of model refinement.

Replication	Apart from each of the crystallographic structure reported in this study being determined using the data obtained from a single crystal, all experiments of replication were successful.
Randomization	For the crystallographic work, the free R value flag for the reflection data was assigned randomly to unique reflections using Phenix (V1.11.1) . The enzymatic activity of the wild type and protein variants was directly determined without randomization.
Blinding	Blinding was not relevant to this study. This is an enzymatic study and appropriate control experiments were included.

Reporting for specific materials, systems and methods

We require information from authors about some types of materials, experimental systems and methods used in many studies. Here, indicate whether each material, system or method listed is relevant to your study. If you are not sure if a list item applies to your research, read the appropriate section before selecting a response.

Materials & experimental systems

n/a	Involved in the study	
☒	☐	Antibodies
☒	☐	Eukaryotic cell lines
☒	☐	Palaeontology and archaeology
☒	☐	Animals and other organisms
☒	☐	Clinical data
☒	☐	Dual use research of concern
☒	☐	Plants

Methods

n/a	Involved in the study	
☒	☐	ChIP-seq
☒	☐	Flow cytometry
☒	☐	MRI-based neuroimaging

Plants

Seed stocks	*Report on the source of all seed stocks or other plant material used. If applicable, state the seed stock centre and catalogue number. If plant specimens were collected from the field, describe the collection location, date and sampling procedures.*
Novel plant genotypes	*Describe the methods by which all novel plant genotypes were produced. This includes those generated by transgenic approaches, gene editing, chemical/radiation-based mutagenesis and hybridization. For transgenic lines, describe the transformation method, the number of independent lines analyzed and the generation upon which experiments were performed. For gene-edited lines, describe the editor used, the endogenous sequence targeted for editing, the targeting guide RNA sequence (if applicable) and how the editor was applied.*
Authentication	*Describe any authentication procedures for each seed stock used or novel genotype generated. Describe any experiments used to assess the effect of a mutation and, where applicable, how potential secondary effects (e.g. second site T-DNA insertions, mosiacism, off-target gene editing) were examined.*
